# Impact of AlphaFold on structure prediction of protein complexes: The CASP15‐CAPRI experiment

**DOI:** 10.1002/prot.26609

**Published:** 2023-10-31

**Authors:** Marc F. Lensink, Guillaume Brysbaert, Nessim Raouraoua, Paul A. Bates, Marco Giulini, Rodrigo V. Honorato, Charlotte van Noort, Joao M. C. Teixeira, Alexandre M. J. J. Bonvin, Ren Kong, Hang Shi, Xufeng Lu, Shan Chang, Jian Liu, Zhiye Guo, Xiao Chen, Alex Morehead, Raj S. Roy, Tianqi Wu, Nabin Giri, Farhan Quadir, Chen Chen, Jianlin Cheng, Carlos A. Del Carpio, Eichiro Ichiishi, Luis A. Rodriguez‐Lumbreras, Juan Fernandez‐Recio, Ameya Harmalkar, Lee‐Shin Chu, Sam Canner, Rituparna Smanta, Jeffrey J. Gray, Hao Li, Peicong Lin, Jiahua He, Huanyu Tao, Sheng‐You Huang, Jorge Roel‐Touris, Brian Jimenez‐Garcia, Charles W. Christoffer, Anika J. Jain, Yuki Kagaya, Harini Kannan, Tsukasa Nakamura, Genki Terashi, Jacob C. Verburgt, Yuanyuan Zhang, Zicong Zhang, Hayato Fujuta, Masakazu Sekijima, Daisuke Kihara, Omeir Khan, Sergei Kotelnikov, Usman Ghani, Dzmitry Padhorny, Dmitri Beglov, Sandor Vajda, Dima Kozakov, Surendra S. Negi, Tiziana Ricciardelli, Didier Barradas‐Bautista, Zhen Cao, Mohit Chawla, Luigi Cavallo, Romina Oliva, Rui Yin, Melyssa Cheung, Johnathan D. Guest, Jessica Lee, Brian G. Pierce, Ben Shor, Tomer Cohen, Matan Halfon, Dina Schneidman‐Duhovny, Shaowen Zhu, Rujie Yin, Yuanfei Sun, Yang Shen, Martyna Maszota‐Zieleniak, Krzysztof K. Bojarski, Emilia A. Lubecka, Mateusz Marcisz, Annemarie Danielsson, Lukasz Dziadek, Margrethe Gaardlos, Artur Gieldon, Adam Liwo, Sergey A. Samsonov, Rafal Slusarz, Karolina Zieba, Adam K. Sieradzan, Cezary Czaplewski, Shinpei Kobayashi, Yuta Miyakawa, Yasuomi Kiyota, Mayuko Takeda‐Shitaka, Kliment Olechnovic, Lukas Valancauskas, Justas Dapkunas, Ceslovas Venclovas, Bjorn Wallner, Lin Yang, Chengyu Hou, Xiaodong He, Shuai Guo, Shenda Jiang, Xiaoliang Ma, Rui Duan, Liming Qui, Xianjin Xu, Xiaoqin Zou, Sameer Velankar, Shoshana J. Wodak

**Affiliations:** ^1^ Univ. Lille, CNRS, UMR8576 – UGSF – Unité de Glycobiologie Structurale et Fonctionnelle Lille France; ^2^ Biomolecular Modeling Laboratory The Francis Crick Institute London UK; ^3^ Bijvoet Center for Biomolecular Research, Faculty of Science – Chemistry Utrecht University Utrecht The Netherlands; ^4^ Institute of Bioinformatics and Medical Engineering, School of Electrical and Information Engineering Jiangsu University of Technology Changzhou China; ^5^ Dept. of Electrical Engineering and Computer Science University of Missouri Columbia Missouri USA; ^6^ Choju‐Medical Institute Fukushimuta Hospital Toyohashi‐City Aichi‐ken Japan; ^7^ International University of Health and Welfare (IUHV Hospital) Nasushiobara‐City Japan; ^8^ Instituto de Ciencias de la Vida y del Vino (ICVV) CSIC ‐ Universidad de La Rioja ‐ Gobierno de La Rioja Logrono Spain; ^9^ Barcelona Supercomputing Center (BSC) Barcelona Spain; ^10^ Dept. of Chemical and Biomolecular Engineering Johns Hopkins University Baltimore Maryland USA; ^11^ Program in Molecular Biophysics Johns Hopkins University Baltimore Maryland USA; ^12^ School of Physics Huazhong University of Science and Technology Wuhan China; ^13^ Protein Design and Modeling Lab, Dept. of Structural Biology Molecular Biology Institute of Barcelona (IBMB‐CSIC) Barcelona Spain; ^14^ Zymvol Biomodeling Barcelona Spain; ^15^ Dept. of Computer Science Purdue University West Lafayette Indiana USA; ^16^ Dept. of Biological Sciences Purdue University West Lafayette Indiana USA; ^17^ Dept. of Biotechnology, Bhupat and Jyoti Mehta School of Biosciences Indian Institute of Technology Madras Chennai India; ^18^ Dept. of Computer Science Tokyo Institute of Technology Yokohama Japan; ^19^ Boston University Boston Massachusetts USA; ^20^ Stony Brook University New York City New York USA; ^21^ Sealy Center for Structural Biology and Molecular Biophysics University of Texas Medical Branch Galveston Texas USA; ^22^ King Abdullah University of Science and Technology (KAUST) Saudi Arabia; ^23^ Department of Chemistry and Biology University of Salerno Fisciano Italy; ^24^ University of Naples “Parthenope” Naples Italy; ^25^ University of Maryland Institute for Bioscience and Biotechnology Research Rockville Maryland USA; ^26^ Dept. of Cell Biology and Molecular Genetics University of Maryland College Park Maryland USA; ^27^ Dept. of Chemistry and Biochemistry University of Maryland College Park Maryland USA; ^28^ School of Computer Science and Engineering The Hebrew University of Jerusalem Jerusalem Israel; ^29^ Department of Electrical and Computer Engineering Texas A&M University College Station Texas USA; ^30^ Department of Computer Science and Engineering Texas A&M University College Station Texas USA; ^31^ Institute of Biosciences and Technology and Department of Translational Medical Sciences Texas A&M University Houston Texas USA; ^32^ University of Gdansk Gdansk Poland; ^33^ Technical University of Gdansk Gdansk Poland; ^34^ School of Pharmacy Kitasato University Minato‐ku Tokyo Japan; ^35^ Institute of Biotechnology, Life Sciences Center Vilnius University Vilnius Lithuania; ^36^ Bioinformatics Division, Department of Physics, Chemistry, and Biology Linkoping University Linköping Sweden; ^37^ National Key Laboratory of Science and Technology on Advanced Composites in Special Environments, Center for Composite Materials and Structures Harbin Institute of Technology Harbin China; ^38^ School of Aerospace, Mechanical and Mechatronic Engineering The University of Sydney New South Wales Australia; ^39^ School of Electronics and Information Engineering Harbin Institute of Technology Harbin China; ^40^ Shenzhen STRONG Advanced Materials Research Institute Col, Ltd Shenzhen People's Republic of China; ^41^ Dalton Cardiovascular Research Center University of Missouri Columbia Missouri USA; ^42^ Dept. of Physics and Astronomy University of Missouri Columbia Missouri USA; ^43^ Dept. of Biochemistry University of Missouri Columbia Missouri USA; ^44^ Institute for Data Science and Informatics University of Missouri Columbia Missouri USA; ^45^ Protein Data Bank in Europe, European Molecular Biology Laboratory European Bioinformatics Institute (EMBL‐EBI) Hinxton Cambridge UK; ^46^ VIB‐VUB Center for Structural Biology Brussels Belgium

**Keywords:** AlphaFold, blind prediction, CAPRI, CASP, deep learning, protein assemblies, protein complexes, protein‐protein interaction

## Abstract

We present the results for CAPRI Round 54, the 5th joint CASP‐CAPRI protein assembly prediction challenge. The Round offered 37 targets, including 14 homodimers, 3 homo‐trimers, 13 heterodimers including 3 antibody–antigen complexes, and 7 large assemblies. On average ~70 CASP and CAPRI predictor groups, including more than 20 automatics servers, submitted models for each target. A total of 21 941 models submitted by these groups and by 15 CAPRI scorer groups were evaluated using the CAPRI model quality measures and the DockQ score consolidating these measures. The prediction performance was quantified by a weighted score based on the number of models of acceptable quality or higher submitted by each group among their five best models. Results show substantial progress achieved across a significant fraction of the 60+ participating groups. High‐quality models were produced for about 40% of the targets compared to 8% two years earlier. This remarkable improvement is due to the wide use of the AlphaFold2 and AlphaFold2‐Multimer software and the confidence metrics they provide. Notably, expanded sampling of candidate solutions by manipulating these deep learning inference engines, enriching multiple sequence alignments, or integration of advanced modeling tools, enabled top performing groups to exceed the performance of a standard AlphaFold2‐Multimer version used as a yard stick. This notwithstanding, performance remained poor for complexes with antibodies and nanobodies, where evolutionary relationships between the binding partners are lacking, and for complexes featuring conformational flexibility, clearly indicating that the prediction of protein complexes remains a challenging problem.

## 1 INTRODUCTION

Protein–protein interactions and multi‐protein assemblies, which often include other macromolecular components such as DNA or RNA, play crucial roles in cellular processes^1^ and their disruption or deregulation often cause disease.^2^, ^3^ Characterizing these interactions and elucidating their functions at the molecular and cellular levels have therefore been important goals in molecular biology and medicine.

Of critical importance to these endeavors are atomic‐resolution 3D structures of these assemblies. These structures are produced by experimental techniques such as x‐ray crystallography, and more recently by cryo‐electron microscopy (cryo‐EM), with the resulting structural models deposited into the worldwide Protein Data Bank (wwPDB).^4^ Unfortunately, however, little or no structural information is available for most of the protein complexes that form in the cell or that can be characterized by modern proteomics and other methods.

The recent spectacular technical advances in single‐molecule cryo‐EM techniques, specifically geared at determining the structure of large macromolecular assemblies at atomic resolution^5^, ^6^ should enable to narrow this gap. An increasingly important role in the efforts to populate the uncharted landscape of protein complexes has also been played by computational methods for predicting the structure of these complexes. The last two decades have witnessed a steady progress of these methods. These methods include increasingly efficient ab‐initio docking algorithms,^7^, ^8^ which build atomic resolution models of a protein complex taking as input the amino acid sequence of the protein components, or template‐based methods that model the structure of a target complex using as template the known structure of a complex between related proteins.^9^, ^10^ Data‐driven docking procedures that integrate data from various sources to guide the docking algorithms have also been popular.^11^ These developments have been greatly facilitated by the continued success of structural biology in enriching the structural repertoire of individual proteins—the building blocks of larger assemblies—and the explosion of the number of available protein sequences.

Further enrichment of this repertoire was enabled by increasingly powerful methods for predicting the 3D structure of single protein chains from sequence information alone, commonly referred to as ab‐initio modeling, which exploit multiple sequence alignments of related proteins to predict residue‐residues contacts crucial to defining the protein fold.^12^, ^13^, ^14^ More recently these methods have greatly benefitted from the incorporation of Artificial Intelligence (AI) Deep Learning (DL) techniques,^15^, ^16^ culminating in the phenomenal success of AlphaFold2 (AF2), the DL algorithm developed by Google DeepMind,^17^ in predicting the atomic structure of single protein chains in the CASP14 blind prediction challenge,^18^ the 14th prediction season of CASP (Critical Assessment of Structure Prediction)^19^; and this to an accuracy comparable with experimental methods. This achievement has been a game changer with immense repercussions across the fields of computational and experimental structural biology.^20^, ^21^ The software of these algorithms was made freely available to the public^22^ (https://github.com/deepmind/alphafold) setting the stage for rapid further developments.^23^ Additionally, DeepMind has partnered with the European Bioinformatics Institute (EBI) to create AlphaFold‐DB,^24^ offering open access to over 200 million protein structures predicted by AlphaFold, providing broad coverage of UniProt.^25^

The vast increase in high accuracy coverage of protein 3D structure space is already having a major impact in many areas of scientific research, including elucidating aspects of evolutionary relationships and protein function,^26^ identifying of potential drug targets^27^ and greatly aiding experimental structure determination.^28^ However, AF2 as designed, and hence also AlphaFold‐DB, provide no information on the dynamic properties of proteins nor on the alternative conformations that proteins sample to carry out their function.^29^ Information is also lacking on functionally important bound small molecule ligands, and of special relevance here, no information is provided on the oligomeric structure of proteins, where two or more proteins form complexes, often including other macromolecular components such as RNA and DNA.

An obvious next frontier for DL‐based protein structure prediction methods is the accurate prediction of complexes and larger protein assemblies.^30^, ^31^ Indeed, during the 18 months period following CASP14, a wave of benchmarking studies suggested that extensions of DL‐based methods to the prediction of protein complexes will provide a major advance over traditional docking methods. For example, AF2 was reported to successfully model the structure of a set of protein complexes of known stoichiometry, albeit not consistently to high accuracy, by feeding it the concatenated sequences of the interacting component proteins.^32^ Better performance, albeit still not reaching the high model accuracy obtained for single chain proteins, was achieved for AlphaFold2‐Multimer (AF2‐M), the more recent inference engine of AlphaFold, directly trained on protein complexes from the PDB.^33^ Approaches have also been proposed to integrate AF2 predictions of complexes with classical docking calculations and using the predicted complexes as templates for AF2 to significantly improve the performance of either method used independently.^34^ Building toward the CASP15 conference held beginning of December of 2022, the key question has been how these game changing developments will impact the modeling of protein complexes. More specifically, the extent to which the power of AlphaFold and other DL‐based prediction methods, such as RoseTTAFold,^35^ will be harnessed by the community to produce a major leap in performance over more classical modeling approaches in the context of blind predictions.

Here we present the evaluation of the results obtained in the CASP15‐CAPRI prediction season, the 5th joint assembly prediction experiment of CASP and CAPRI held in the summer of 2022, representing Round 54 of CAPRI. CAPRI (Critical Assessment of PRedicted Interactions) (https://www.ebi.ac.uk/pdbe/complex-pred/capri/; http://www.capri-docking.org/) is a community‐wide initiative inspired by CASP. Established in 2001, CAPRI has offered computational biologists the opportunity to test their algorithms in blind predictions of unpublished experimentally determined 3D structures of protein complexes, the “targets,” on a rolling basis several times a year. Whereas CASP has been very instrumental in stimulating the field of protein structure prediction, CAPRI has contributed to advancing the field of modeling protein assemblies. Initially focusing on testing procedures for predicting protein–protein complexes, CAPRI is currently also dealing with protein‐peptide, protein‐nucleic acids, and protein‐oligosaccharide complexes. In addition, CAPRI has organized challenges to evaluate computational methods for estimating binding affinity of protein–protein complexes^36^, ^37^ and predicting the positions of water molecules at the interfaces of protein complexes.^38^

Motivated by the growing need of better integration of methods for the prediction of protein 3D structures with those of modeling protein assemblies, closer ties have been established between the CASP and CAPRI communities by running joint CASP‐CAPRI assembly prediction experiments. Four such experiments were conducted in the summers of 2014, 2016, 2018, and 2020, respectively, with results presented at the CASP11, CASP12, CASP13, CASP14 meetings and published in 4 special issues of Proteins.^30^, ^39^, ^40^, ^41^, ^42^

CAPRI Round 54 included the prediction experiments run jointly with CASP as well as scoring experiments, uniquely offered by CAPRI, where participants are invited to identify the correct association modes from an ensemble of anonymized predicted complexes generated during the assembly prediction experiment.^43^, ^44^ This Round offered 37 targets, totaling 38 assessment units (AUs; an assessment unit is the portion of a more complex target for which models were evaluated independently). This is nearly twice the number of targets than in the CASP14‐CAPRI challenge. The 37 targets included 14 homodimers, 3 homo trimers, 13 hetero dimers including 3 antibody–antigen complexes, and 7 large assemblies, 2 of which were split into 2 evaluation units each. Expecting predictors to have ready access to the AF2, AF2‐M, and AlphaFold‐DB, we assumed that many more of the targets offered by CASP would represent tractable modeling problems that depend less on template availability. Nearly all but four of the CASP assembly targets were therefore offered in this Round. The four refused targets were low resolution structures or raised doubts as to their reported stoichiometry.

All submitted models were evaluated using quality measures agreed upon by the CAPRI community.^42^, ^43^, ^44^ A separate evaluation of the CASP15 assembly prediction performance, reported at the CASP15 meeting and in this Special Issue [paper by Özden & Karaca, this issue], was performed by the CASP assembly assessment team in collaboration with the CASP prediction center. As in previous joint prediction experiments, the CASP and CAPRI assessment teams closely collaborated in defining the prediction problem for complex targets, discussing evaluation strategies, and comparing assessment results.

A significantly larger number of CASP groups participated in the CASP15 assembly prediction challenge—between 40 and 58 groups, depending on the target—than in previous challenges, reflecting the keen interest of the community in the prediction of protein complexes. Therefore, to obtain a more complete picture of the impact of deep learning methods on the predictions results across the community, the CAPRI model quality measures were used to evaluate the models submitted by both CASP and CAPRI participants for the Round 54 targets and to rank the prediction performance. This consolidated assessment is presented and discussed.

## 2 THE TARGETS

**FIGURE 1:**
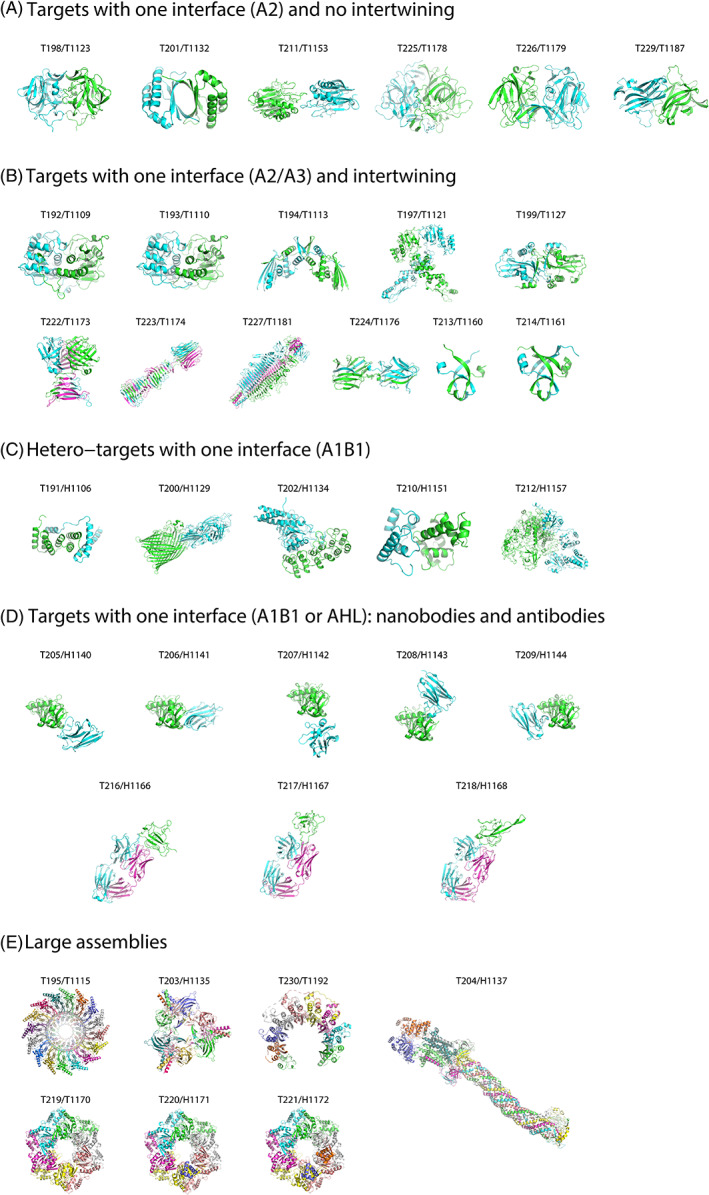
Pictorial representation of the targets of Round 54. The 37 targets of this prediction Round are grouped into five categories: (A) Homodimers without intertwining, (B) homodimers and homotrimers with intertwining, (C) heterodimers of which a special category are complexes with nanobodies (Nb) and antibodies (Ab, consisting of a heavy (H) and light (L) chain) (D), and (E) large homomeric and heteromeric assemblies. The targets are annotated with their CAPRI and CASP ID's. Note that T192/T1109 strictly speaking belongs to group (A), but it is only a point mutation away from T193/T1110, which shows intertwining, and was therefore assigned to group (B).

The 37 targets of Round 54 were grouped into five categories (I‐V) based on the type of their interfaces and association mode (Figure 1). These targets included proteins from bacteria (~34%), Eukaryotes (human, mouse) (~30%), viruses (26%) and plant or fungi (9%). Essential details about the 37 targets, including kingdom, UniProt ID, protein size, interface area, and protein name(s) are listed in Table 1. Further details about the targets can be found in the Table S5.

**TABLE 1:** Target details.

Targets with one interface (A2) and no intertwining						
CAPRI	/ CASP ID	Kingdom^(^ ^a^ ^)^: UniProt		Size	BSA^(^ ^b^ ^)^	Name
T198	/ T1123	V:	B6UYJ1	266	1570	Capsid polyprotein VP90
T201	/ T1132	B:	A0A072ZNL3	102	1125	Antibiotic biosynthesis monooxygenase
T211	/ T1153	E:	Q7L9B9	299	550	Endonuclease/exonuclease/phosphatase family domain‐containing protein 1
T225	/ T1178	V:	Unannotated	306	3715	
T226	/ T1179	V:	Unannotated	261	1830	
T229	/ T1187	E:	Q94EW1	166	935	Nictaba
Targets with one interface (A2/A3) and intertwining						
T192	/ T1109	B:	Q8XYF6(D180A)	227	2100	Putative transcription regulator protein
T193	/ T1110	B:	Q8XYF6	227	2265	Putative transcription regulator protein
T194	/ T1113	V:	A0A4D6BFJ2	193	2750	Uncharacterized protein
T197	/ T1121	B:	A0A0H2ZM47	381	1420	DUF3322 and DUF2220 domain‐containing protein
T199	/ T1127	E:	Q9ZV05	211	3355	L‐ornithine N5‐acetyltransferase NATA1
T213	/ T1160		Designed	48	1080	
T214	/ T1161		Designed	48	1845	
T222	/ T1173	B:	Q6MNC5	204	2015	Cell wall surface anchor family protein
T223	/ T1174	B:	Q6ML84	338	5715	Uncharacterized protein
T224	/ T1176	B:	Unannotated	170	5700	Hypothetical protein
T227	/ T1181	V:	G0XNW6	688	4940	Tail fiber protein
Hetero‐targets with one interface (A1B1)						
T191	/ H1106	B:	P0C2N4,P61417	122 114	1440	Yop proteins translocation protein; Chaperone protein
T200	/ H1129	V:	P06971	747	2040	Ferrichrome outer membrane transporter/phage receptor
			P23207	640		Receptor‐binding protein pb5
T202	/ H1134	B:	A0A0H3CKN4	230	2000	Ankyrin repeat domain‐containing protein
			A0A0M7ENE2	313		Phospholipase
T210	/ H1151	B:	P9WGI1	112	740	RNA polymerase sigma factor SigA
			P9WF37	116		Probably transcriptional regulator WhiB6
T212	/ H1157	E:	G0SCX7,G0SGS2	1029,495	2250	Alpha‐1,2‐mannosidase; Protein disulphide‐isomerase
Targets with one interface (A1B1 or A:HL): nanobodies and antibodies						
		E:	P16330	219		2′,3′‐cyclic‐nucleotide 3′‐phosphodiesterase
T205	/ H1140	E:	Nanobody	132	775	Nb
T206	/ H1141	E:	Nanobody	217	925	Nb7e
T207	/ H1142	E:	Nanobody	128	585	Nb8c
T208	/ H1143	E:	Nanobody	131	770	Nb10e
T209	/ H1144	E:	Nanobody	122	895	Nb8d
		V:	P0DTC9	130		Coronavirus nucleocapsid
T216	/ H1166	E:	Human Antibody	216 231	1690	S24‐188 Fab
T217	/ H1167	E:	Human Antibody	212 218	1600	S24‐188 Fab
T218	/ H1168	E:	Human Antibody	215 222	1820	S24‐188 Fab
Large assemblies						
T195	/ T1115	E:	P27105	288	3350	Stomatin
T203	/ H1135	E:	O94901	195	550–1100	SUN domain‐containing protein 1
			Q12912	25		Inositol 1,4,5‐triphosphate receptor associated 2
T204	/ H1137		Unannotated	266–653	750–6500	
T219	/ T1170	B:	Q5M2B1	318	1900	RuvB
T220	/ T1171	B:	Q5M2B1,P66746	318,48	1900,680	RuvB; RuvA
T221	/ T1172	B:	Q5M2B1,P66746	318,48	1900,640	RuvB; RuvA
T230	/ T1192	E:	P43351	418	2225	DNA repair protein RAD52 homolog

*Note*: The Table shows the 37 targets of Round 54 grouped by target category. Listed are target ID's (CASP and CAPRI), the kingdom of the organism and UniProt ID if available, the size of the target in terms of sequence length (Size) and buried surface area (BSA/Å^2^), and the name of the protein(s). For further details about the targets see Table S5.

^a^ Bacteria, eukaryotes, viruses.

^b^ Buried surface area.

In past Rounds (including CASP‐CAPRI challenges) the target difficulty level could be assessed quite reliably based on the availability and quality of templates. Often, specific properties of the target structure, such as interface size or extent of protein flexibility also provided useful tips. In this Round, ready access by the community to the AlphaFold software and AlphaFold‐DB had a significantly impact on the prediction performance as will be shown in the present evaluation.

In the following, we present the characteristics of the 37 target structures. We describe the considerations that helped assess their difficulty level and mention examples where the wide use of the AF2 tools appeared to impact this assessment.

### 2.1 Category I: Homomeric targets with one interface (A2) and no intertwining: T198, T201, T211, T225, T226, T229

The 6 targets in this category represent symmetric homodimers forming one binding interface each.

As seen from Table 1, the size of the proteins in these targets ranges from 102 (T201/T1132) to 306 (T225/T1178) residues, and the area buried in the interface (BSA, for Buried Surface Area) ranges from 550 Å^2^ (T211/T1153) to 3715 Å^2^ (T225/T1178). These targets comprised 3 viral proteins (T198/T1123, T225/T1178, T226/T1179), a protein from bacteria (T201/T1132), a human protein (T211/T1153), and one plant protein (T229/T1187). Two of the viral proteins, (T225/T1178, T226/T1179) were unannotated.

**FIGURE 2:**
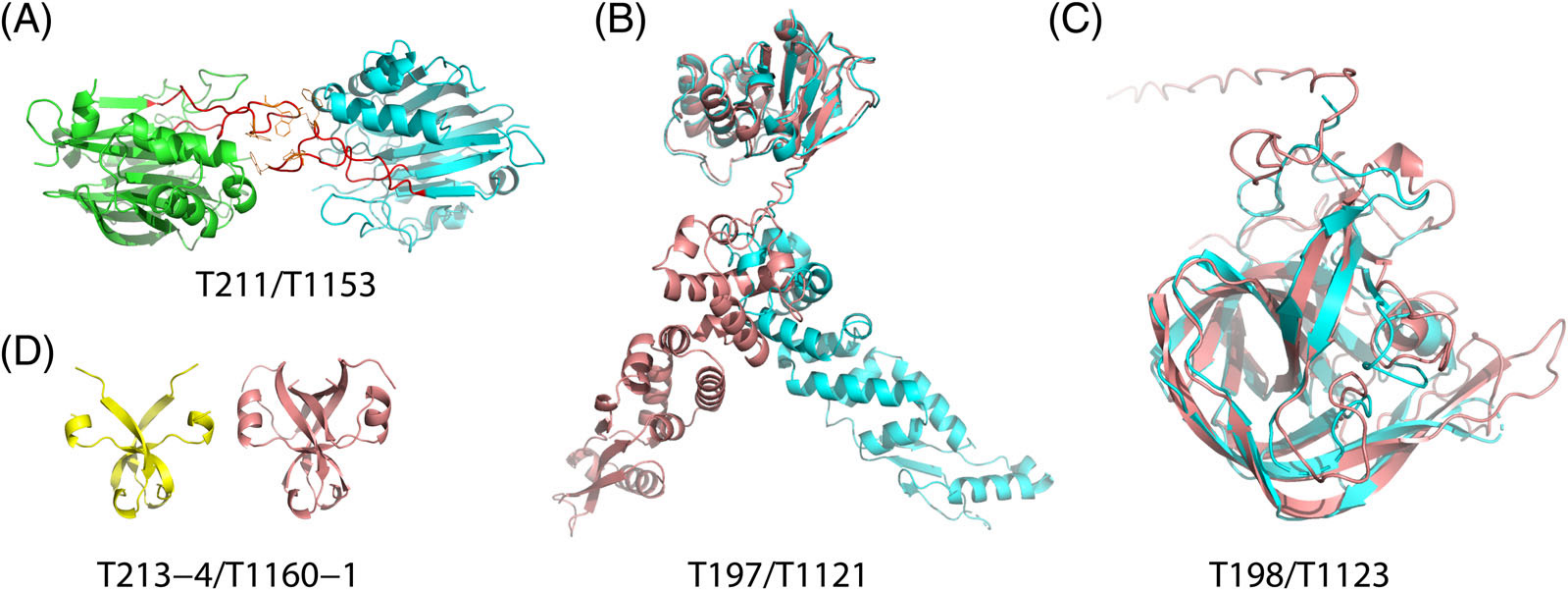
Examples of challenging homomeric targets with and without intertwining. (A) Association mode of T211/T1153, afforded by the Trp (orange)‐rich loops (red). (B) T197/T1121 monomer (cyan) and AF2 model (salmon), illustrating how the flexibility of the loop connecting the two domains affected the AF2‐M prediction results. (C) The T198/T1123 monomer (cyan) and AF2 model (salmon), illustrating how the flexibility of several loops affects the prediction results, here AF2. (D) Dimeric structures of the ancient protein reconstructions of T213/T1160 (left, yellow) and T214/T1161 (right, salmon), illustrating the changes in the interface between the subunits.

Based on template availability alone, T198/T1123, T201/T1132, and T229/T1187 were expected to be easy prediction problems, whereas T211/T1153, T225/T1178, and T226/T1179 were predicted to be difficult targets. For example, only a distantly related template (seq‐id 15% of the structured portion; rmsd 3.85 Å) was available for T211/T1153, the Endonuclease/exonuclease/phosphatase family domain‐containing protein1 dimer. Furthermore, its interface involves two loops forming a small interface (BSA 550 Å^2^) mediating interactions through the association of 2 tryptophane residues (see Figure 2A). Yet this homodimer was rather well predicted (see Section 5), owing mainly to the widespread use of the new DL‐based prediction methods. On the other hand, T198/T1123, the viral capsid polyprotein VP90, features a sizable interface (BSA 1570 Å^2^). It had a template available for the full homodimer (albeit differing from the target dimer by 7.9 Å rmsd). In addition, a good quality model was predicted for the monomer by AF2 (standard version run by the assessment team), equivalent to having an excellent monomeric template (Figure 2B). Nonetheless, the prediction performance for this homodimer was poor.

### 2.2 Category II: Homomeric targets with one interface (A2/A3) and intertwining: T192‐T194, T197, T199, T213, T214, T222‐T224, T227

The 11 targets in this category comprise eight symmetric homodimers and three homotrimers, all featuring one unique interface displaying some degree of intertwining (residues from one subunit reaching out to interact with the neighboring subunit^45^). Proteins in these homodimers range in size from 48 residues (T213/T1160, T214/T1161, two designed proteins) to 381 residues (T197/T1121), and the areas buried in their interfaces range from 1080 Å^2^ (T213/T1160, one of the designed proteins) to 5700 Å2 (T224/T1176). Due to various degrees of intertwining a total of five of the homodimers bury an area of more than 2000 Å^2^ each.

The homodimers comprise 4 bacterial proteins (T192/T1109, T193/T1110, T197/T1121, T224/T1176), one viral protein (T194/T1113), one plant protein, and the two designed proteins mentioned above. The three homotrimers comprise two bacterial proteins (T222/T1173, T223/T1174) and one viral protein (T227/T1181). These proteins range in size from 204 residues (T222/T1173) to 688 residues (T227/T1181) and feature interfaces areas ranging from 2015 Å^2^ (T222/T1173) to 5715 Å^2^ (T223/T1174).

Based on template availability and on the quality of models predicted by AF2 and AF2‐M (standard versions run by the assessment team), four of the homodimer targets (T192/T1109, T193/T1110, T194/T1113, T199/T1127) were expected to be easy targets. This expectation was supported by a good prediction performance across groups (see Section 5).

The other seven targets of this category (T197/T1121, T213/T1160, T214/T1161, T222/T1173, T223/T1174, T224/T1176, T227/T1181) were expected to be difficult targets based mainly on the specific properties of the corresponding structures. The difficulty of T197/T1121, T222/T1173, T223/T1174, and T227/T1181 was inferred from the presence in the proteins of two or more structural domains connected by flexible linkers (see example in Figure 2C). For T224/T1176, the difficulty appeared to reside in the significant intertwining in this complex. Yet, T222/T1173, the bacterial cell wall anchor family protein, and T227/T1181, the tail fiber viral protein, were rather well predicted (see Section 5) and may therefore be classified as easy targets. On the other hand, only incorrect models were produced for the intertwined complex of T224/T1176.

For the two small, designed proteins produced by ancestral reconstruction (T213/T1160, T214/T1161) (Figure 2D), the prediction difficulty was suggested by the presence of an unstructured C‐terminal segment and a small interface, more prominent in T213/T1160 than T214/T1161, in agreement with the overall poor trends in the prediction performance.

### 2.3 Category III: Heteromeric targets with one interface (A1/B1): T191, T200, T202, T210, T212

The 5 targets in this category are heterodimers involving the interaction of proteins whose size ranges from 112 to 1029 residues. The three dimers (T200/H1129, T202/H1134, T212/H1157) of the larger proteins also form sizable interfaces (~2000 Å^2^), whereas the two dimers of the smaller proteins (T191/H1106, T210/H1151) display smaller interfaces (respectively 1440 and 740 Å^2^).

**FIGURE 3:**
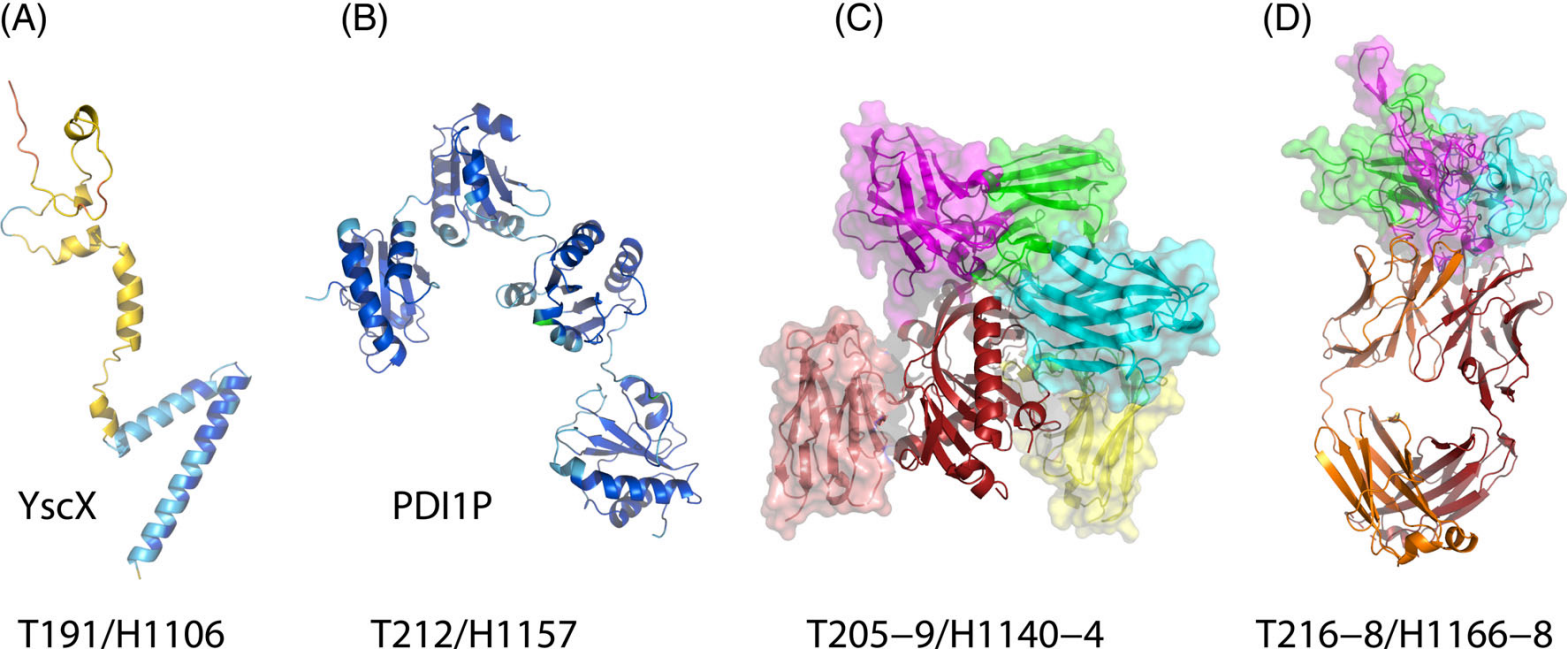
Examples of challenging heteromeric targets. (A) AF2 model of the less well‐structured YscX component of T191/H1106. (B) AF2 model of the PDI1P component of T212/H1157. Color coding of A and B identical to the one used by AlphaFold_DB. (C) The nanobody (Nb) binding modes to the CNPase (red) in targets T205/H1140 (green), T206/H1141 (cyan), T207/H1142 (magenta), T208/H1143 (yellow), and T209/H1144 (salmon). (D) The antibody (Ab, red and orange) binding modes to the SARS‐CoV‐2 nuclear capsid protein of targets T216/H1166 (green), T217/H1167 (cyan), T218/H1168 (magenta).

For T200/H1129 and T210/H1151, respectively composed of proteins from bacteria and a bacterial virus, the availability of excellent quality models for the independent subunits (templates and an AF2 predicted structure) defined these complexes as easy targets, a definition upheld by the good prediction performance (see Section 5). The other two complexes from bacteria (T191/H1106 and T202/H1134), and T212/H1157, the complex from *T. thermophila*, were expected to be difficult targets. T191/H1106, the YscX/YscY complex (of the Yop proteins translocation protein and Chaperone protein), appeared to be difficult because the YscX component was not well structured (Figure 3A). T202/H1134, a complex between an ankyrin repeat domain‐containing protein and phospholipase, had only poor templates available for the component proteins. In T212/H1157, the complex of alpha‐1,2‐mannosidase with the protein disulphide‐isomerase, the prediction problem appeared to be difficult due to the presence of four domains in the larger alpha‐1,2‐mannosidase, with only three of the domains contributing to the binding interface (Figure 3B). Of these three targets, T191/H1106 and T202/H1134 were nevertheless very well predicted by many groups (see Section 5), whereas the prediction results were poorer for T212/H1157.

Thus, examples of targets in categories I‐III suggest that with the wide use of AF2 tools, the lack of adequate templates may no longer be a reliable criterion for evaluating target difficulty, whereas certain specific properties of the target structure, such as the presence of multiple domains separated by flexible linkers, may be an effective signal.

### 2.4 Category IV: Heteromeric Ab and Nb complexes with one interfaces (A1/B1 or A/HL): T205‐T209, T216‐T218

These eight heterodimer targets were grouped in a category of their own because they involve the interactions of a protein epitope (Ag) with an antibody, or with a nanobody (Nb). Nbs are the recombinant variable domains of heavy‐chain‐only antibodies, with many unique properties, that have become an important tool in structural biology as well for the diagnosis and therapy of diseases.^46^ Deep learning methods such as AF2 confer limited advantage for the prediction of these complexes because evolutionary relationships are not expected to prevail between the binding partners, hence limiting the choices to more classical ab‐initio docking methods or template‐based modeling. This was borne out by the prediction results for these targets (see Section 5).

T205‐T209 represent the complexes of 2′,3′‐cyclic‐nucleotide 3′‐phosphodiesterase protein with 5 different Nbs, binding to different regions of the protein (Figure 3C). The protein is of medium size (129 residues) and the bound Nbs are smaller (122‐132 residues). The interaction interfaces formed in the five complexes are rather limited (~600–900 Å^2^). Of these five targets only T208/H1143 was well predicted because a template with the correct binding mode was available (see Section 5).

The prediction results were significantly poorer for T216‐T218, the complexes of the S24‐188 Fab antibodies (~450 residues in the heavy and light chain) bound to somewhat different regions of the SARS‐COV‐2 nucleoprotein (130 residues), although these complexes form respectable size interfaces (~1600–1800 Å^2^; Figure 3D). Mostly acceptable and medium‐quality models were predicted only for T218/H1168, for which a template featuring the correct binding mode was available, whereas only incorrect models were obtained for T216/H1166 and T217/H1167.

### 2.5 Category V: Large assemblies: T195, T203, T204, T219‐T221, T230

These seven targets represented large multi‐subunit homomeric and heteromeric assemblies, often forming multiple interfaces and displaying higher‐order symmetry. Solved by cryo‐EM to high resolution, these large complexes represent challenging prediction problems, even with the help of AF2.

T195/T1115 is a 16‐mer complex of stomatin (248 residues), featuring a large primary interface (3350 Å^2^), and a secondary minor interface (250 Å^2^) which was not evaluated. This target was expected to be of medium difficulty because a reasonable quality template (2.4 Å rmsd) was available for the individual subunit, forming however a larger assembly (24‐mer).

**FIGURE 4:**
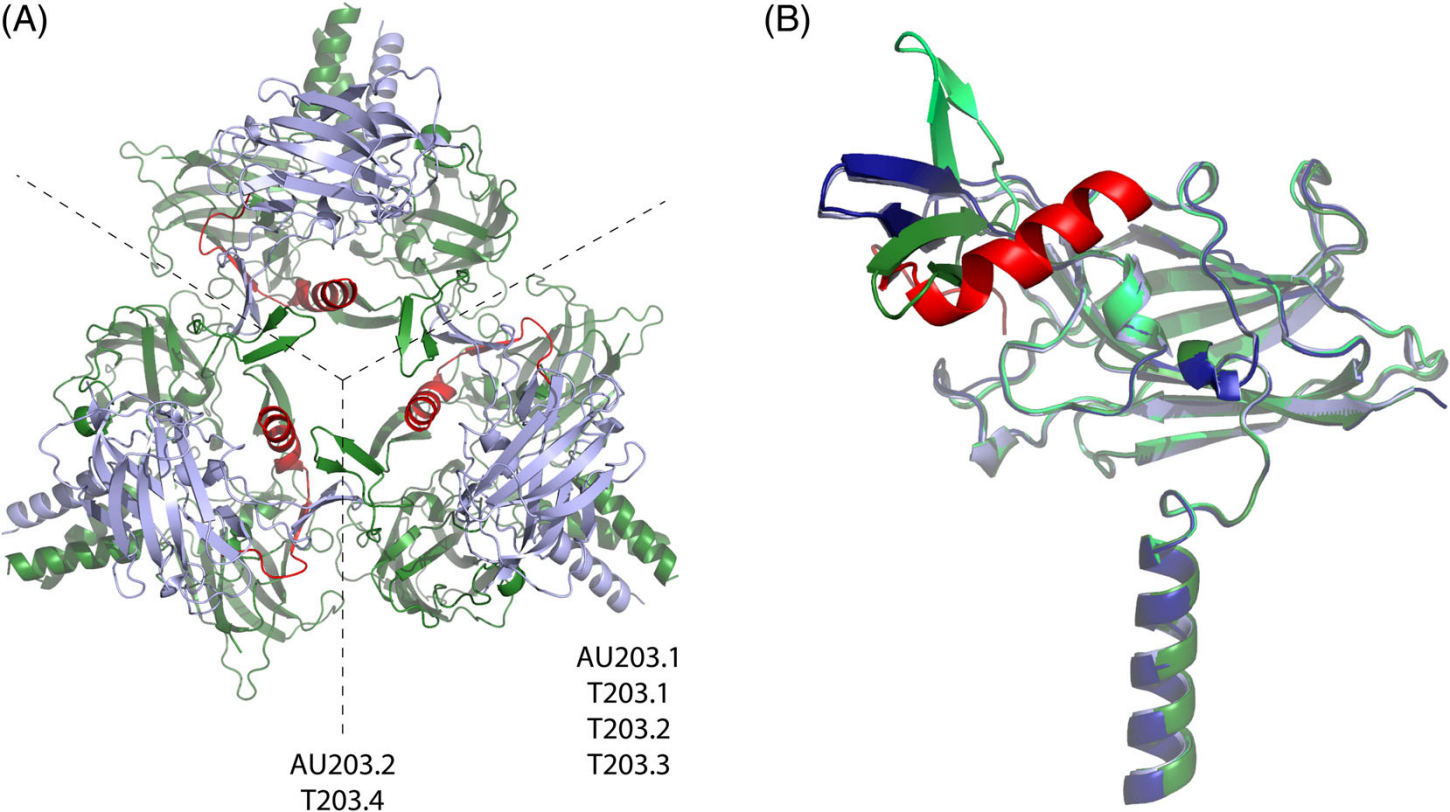
Details of the association mode and assessment units in T203/H1135 large assembly. (A) Shows the association mode of T203/H1135, which assembles into a trimer of trimers. This target was subdivided into 2 assessment units (AUs). AU203.1 is comprised of the minor or inner trimer featuring three interfaces, while AU203.2 represents the major or outer trimer, which consists of a single unique interface. Each minor trimer is formed by three SUN1 monomers that adopt three different conformations, shown in (B), while conserving their internal interface (interface T203.1). In addition, a protein fragment of IRAG2 (red) binds one of the SUN1 monomers (light blue; T203.2; 850 Å^2^), forming a secondary interface (T203.3; 750 Å^2^) to another monomer. The two (green) monomers of the minor trimer form the interface to a neighboring trimer, constituting the interface of AU203.2 (T203.4; 500 Å2), indicated by the dashed lines. (B) Shows the three conformations of the subunits of the minor trimer (dark green, light green, and light blue), plus the conformation found in the template trimer (blue; PDB 6R2I), which was found to overlap with the conformation binding the peptide. However, the alpha‐helical fragment of the peptide was not found in the template and without adopting the other two conformations of SUN1 it could not be accommodated into the minor trimer without clashes.

T203/H1135: This assembly is composed of a trimer of the human SUN1 protein that binds a small peptide (IRAG2) forming an A3B1 complex. This complex forms a larger trimer with stoichiometry (A3B1)3 (Figure 4). A reasonable quality template (3.93 Å rmsd) was available for the A3B1 timer, including the bound peptide (interfaces 1‐3). The corresponding interfaces were therefore expected to represent an easy prediction problem and grouped into one assessment unit (AU) (sub‐complex) (AU203.1). However, prediction performance showed that the A3B1 trimers were difficult to model accurately. To accommodate the interactions with the other copies of the trimer the individual SUN1 subunits adopted three somewhat different conformations, two of which differed from the conformation in the single trimer of the template. Some of these conformational changes were also necessary to accommodate the bound peptide that is somewhat longer than in template. No templates were available for the inter‐trimer weaker unique binding mode (500 Å^2^). The corresponding interface (interface 4) was therefore expected to be difficult to predict and formed the second assessment unit for this target (see Figure 4 for details).

**FIGURE 5:**
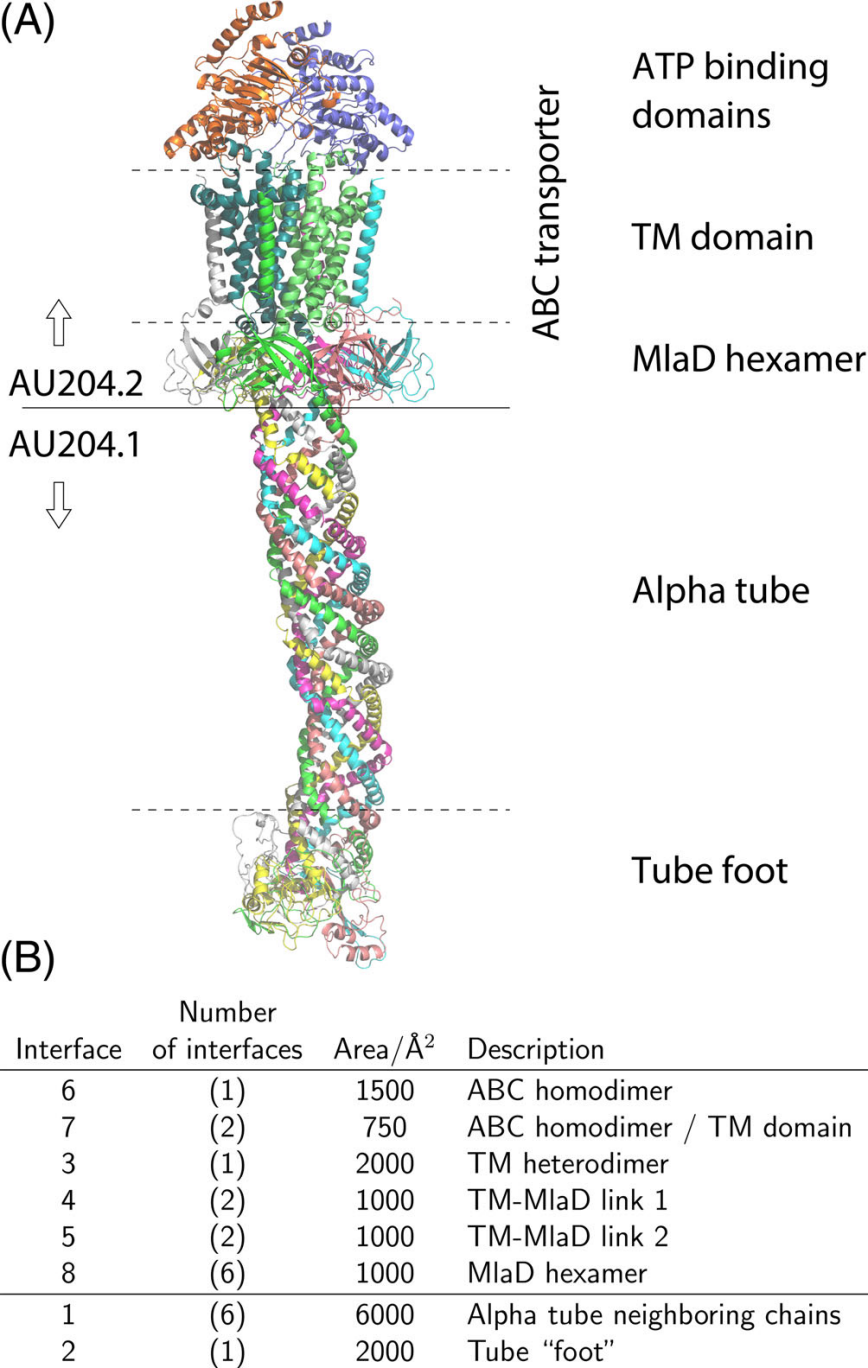
Details of the association mode and assessment units in T204/H1137 large assembly. (A) The assembly of T204/H1137, as resolved by cryo‐EM at 3.10 Å resolution. The bulk of the assembly is made up of six protein chains of different sequences, four of them (white, green, cyan, magenta) starting in the TM domain, all of them containing an MlaD domain and participating in the alpha‐helical tube, and two of them (yellow and salmon; those not in the TM domain) forming a C‐terminal domain annotated here as the tube foot. Whereas all interfaces were assessed, due to their structural similarity many of these interfaces have been grouped together, defining interfaces 1–8 as listed in (B), with the best result for any of the participating interfaces taken as the assessment result for that interface; the original number of interfaces is listed in parentheses. The interfaces are subsequently grouped into two assessment units AU204.1 and AU204.2 (see text for detail).

T204/H1137: This target is a large multi‐component system comprising an ABC transporter and a MlaD hexamer extending into 6 long alpha helices that form a flexible tube‐like structure (see Figure 5). Considering the tube formation and TM domain assembly (ABC transporter and MlaD hexamer) as two distinct modeling problems, this complex was subdivided into two assessment units (AU) for which models were evaluated independently. The first AU (AU204.1) included the interfaces formed by neighboring chains in the tube‐like structure (together taken as interface 1, each burying ~6000 A^2^ surface area), as well as the C‐terminal domain composed of non‐neighboring tube chains (interface 2; 2750 A^2^). Both were expected to be difficult modeling problems due to poor template availability and the flexible nature of the structure. The second AU (AU 204.2) on the other hand, comprising the transmembrane domain formed by an ABC transporter and MlaD hexamer, represented the easier modeling problem, as their internal organization is well known. AU204.2 included the TM heterodimer (interface 3; 2250 A^2^), the ABC homodimer (interface 6; 1500 A^2^), the MlaD hexamer interface (interface 8; 1000 A^2^), and the inter‐domain interfaces between TM/MlaD (interface 4 and 5; 1500 and 750 A^2^) and TM/ABC (interface 7; 750 A^2^).

**FIGURE 6:**
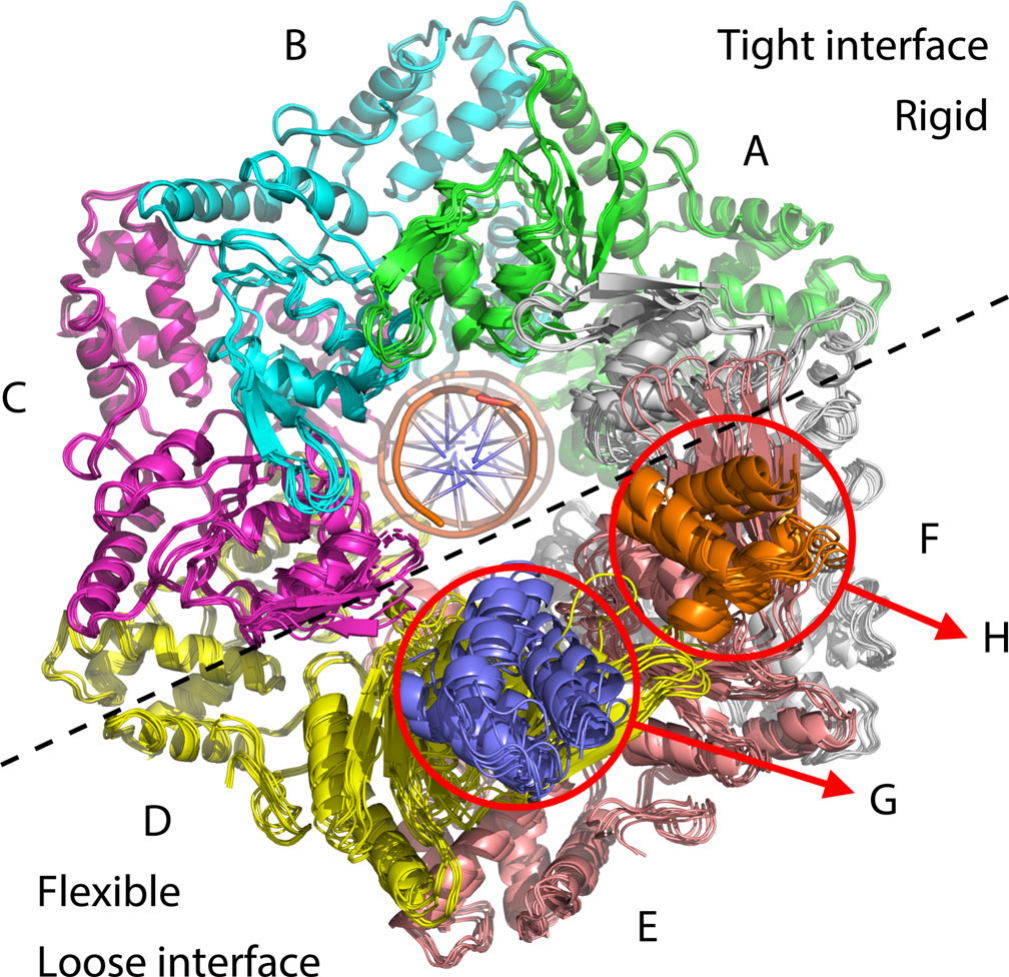
Details of the association mode and assessment units of the large assemblies in T219/T1170, T220/H1171, and T221/H1172. These targets are three different solved structures of the same complex: the RuvB hexamer, bound to a 15 bp dsDNA fragment, which was not included in the modeling problem. The RuvB hexamer accommodates zero (T219/T1170), one (T220/H1171), or two (T221/H1172) RuvA molecules. Shown are all the resolved structures (one for T219/T1170, two for T220/H1171, and four for T221/H1172) superimposed onto the dsDNA segment. Chains A–F of the RuvB hexamer exhibit a tight, rigid interface near the dsDNA and a looser, flexible interface away from it. These interfaces are grouped to a tight “super” interface T219.1 (A:B and B:C), an intermediate “super” interface T219.2 (C:D and A:F) and a loose “super” interface T219.3 (D:E and E:F) (see text). The interface definitions are the same for T220 and T221. These nine interfaces are grouped together in AU219. The binding of a RuvA molecule (chains G and H) to a RuvB monomer form interfaces T220.4 (and T221.4) grouped into AU220.

T219/T1170, T220/H1171, and T221/H1172: These targets are 3 different solved structures of a similar modeling problem. At the basis lies the assembly of 6 RuvB molecules (318 residues) into a hexamer. The hexamer is organized around a 15 bp dsDNA fragment, which was not included in the modeling problem. The RuvB hexamer can accommodate zero (T219/T1170), one (T220/H1171), or two (T221/H1172) RuvA molecules (49 residues) (Figure 6). The hexamer forms a pore loosely accommodating the dsDNA fragment. Neighboring subunits closer to the dsDNA (A, B, C) make tighter contacts, whereas subunits further away from the dsDNA (D, E, F) engage in looser contacts and display higher flexibility. These more distant subunits bind one or two RuvA molecules, thereby losing flexibility at their binding site. These three complexes taken together are subdivided into two distinct AUs: AU219 and AU220. AU219 represents the apo‐hexamer and comprises three unique interfaces, each displaying very similar characteristics in terms of residue‐residue contacts across all three structures. Interface 1 comprises the similar interfaces between subunits AB and BC, interface 2 comprises the interfaces AF and CD, whereas interface 3, comprises interfaces DE and EF (see Figure 6). AU220 represents the RuvB:RuvA complex, and comprises interface 4, which groups the similar RuvB/RuvA interfaces in T220 and T221.

T230/T1192 is a decamer of the human DNA repair protein RAD52 homolog (418 residues) solved by cryo‐EM to high resolution, with only 177 residues resolved in the best monomer unit, and 177/171 residues for the best pair. It featured a unique extensive interface (2225 Å^2^) and had several excellent templates available (rmsd <1 Å, and seq‐id ~44%) and was therefore expected to be an easy target, as also supported by the prediction performance.

Thus, the difficulty level of these large assembly targets was mostly determined by the availability and quality of templates, with good quality templates (for the independent subunits, or even better, for the full complex) making for easier prediction problems. Exceptions occurred for several of the targets displaying higher flexibility of the protein chains, which also led to looser packing of some of the binding interface, or by the requirement to model conformational changes, all of which contributed to a reduced prediction performance and increased target difficulty.

## 3 OVERVIEW OF THE PREDICTION EXPERIMENT

As in previous CASP‐CAPRI challenges and in standard CAPRI Rounds, predictor groups were provided with the amino‐acid sequence or sequences of the target proteins, usually those of the constructs used to determine the structures. For most targets, predictors were also given information (provided by the authors) about the biologically relevant oligomeric state of the protein, the stoichiometry of the complex, and occasionally some additional relevant details about the proteins.

Following the common practice in CAPRI, predictors were invited to submit 100 models for each target, to be used for the scoring challenge (see below). It was stipulated however, that only the 5 top‐ranking models will be evaluated. To continue monitoring the ability of predictors to reliably rank their models, we also report the performance of groups based on their single top‐ranking (top‐1) models.

Scoring experiments were run for all 37 targets (38 AUs) except for the hetero‐complex T191/H1106, for which the time window for prediction was too short. After the predictor submission deadline, all the submitted models (up to 100 per participating group) were shuffled and made available to all the groups participating in the scoring experiment. The “scorer” groups were in turn invited to evaluate the ensemble of uploaded models using the scoring function of their choice and to submit their own 5 top‐ranking ones. Scorer results based on their top‐1 ranking models are also reported. Typical timelines for the prediction and scoring experiments were 3 weeks and 5 days, respectively.

Round 54 participants were invited to submit their models to the CAPRI‐EBI management system. This system generated CASP compliant versions of the 5 top ranking models submitted to CAPRI by predictor and scorer groups. These compliant versions were automatically forwarded to CASP. This procedure afforded a seamless communication between the CASP and CAPRI management systems.

The number of CAPRI and CASP groups (predictors and servers), and CAPRI scorer groups submitting models, and the corresponding number of models for each target assessed here, are listed in the Table S1. A total of ~60–80 predictor and servers groups submitted models for each target. This is nearly twice the number of groups submitting models in previous CASP‐CAPRI challenges. These groups submitted a total of ~170–260 model (CASP) and ~160–1380 models (CAPRI) per target. In general, 15 CAPRI scorer groups submitted a total of 75 models per target.

Altogether, 67 851 models were assessed, of which 21 941 top 5 models were used to evaluate the prediction performance of individual groups.

As already noted, the prediction task was greatly facilitated by the ready access by most participants to the AF2 and AF2‐M software packages, which they used to predict the structure of individual subunits of a complex, or the full complex. As in previous challenges human predictor groups had access to models predicted by participating servers, released by the CASP website after the server submission deadline, although fewer predictor groups appear to have used these models than in previous years (see Supplementary Material; Individual Group Summaries).

## 4 ASSESSMENT METRICS AND PROCEDURES

For ready comparison with the results obtained in previous CAPRI Rounds and previous CASP‐CAPRI experiments,^39^, ^40^ models were evaluated using the standard CAPRI assessment protocol. This protocol was complemented with the DockQ score,^47^, ^48^ a continuous quality metric that integrates the main quality measures of the standard CAPRI protocol (see details below).

The ranking of predictor performance was based on the CAPRI score derived from the parameters evaluated by the standard CAPRI protocol. Alternative ranking based on the DockQ score and on the DockQ Z‐score are also presented and discussed.

### 4.1 The CAPRI assessment and ranking protocols

The standard CAPRI assessment protocol^43^, ^44^ was used to evaluate the quality of the predicted homo‐ and hetero‐complexes. This protocol uses three main parameters, *f*(nat), *L_*rms, and *i_*rms, to measure the quality of a predicted model. *f*(nat) is the fraction of native contacts in the target that is recalled in the model. Atomic contacts below 3 Å are considered clashes and predictions with too many clashes are disqualified (for the definition of native contacts, and the threshold for clashes see reference^43^). *L_*rms is the backbone rmsd (root mean square deviation) over the common set of residues (across all submitted models) of the ligand‐protein after the receptor protein has been superimposed. *i_*rms represents the backbone rmsd calculated over the common set of interface residues after these residues have been structurally superimposed. An interface residue is defined as such when any of its atoms (hydrogen atoms excluded) are located within 10 Å of any of the atoms of the binding partner. Based on the values of these three parameters, models are ranked into four categories: high quality, medium quality, acceptable quality, and incorrect, as previously described.^39^

For targets representing higher‐order oligomers featuring multiple distinct interfaces, submitted models were evaluated by comparing each pair of interacting subunits in the model to each of the relevant pairs of interacting subunits in the target.^39^ When such oligomers represent complex arrangements, the target is subdivided into two or more assessment units (AU), with each AU including a subset of the oligomer distinct interfaces. The quality score for each AUs, *Score*_*AU*_ is computed as a weighted average as follows:

(1) $${\text{Score}}_{\mathrm{AU}}=\left(\omega_{1}n_{\mathrm{ACC}}+\omega_{2}n_{\mathrm{MED}}+\omega_{3}n_{\text{HIGH}}\right)$$

where *n*_ACC_, *n*_MED,_ and *n*_HIGH_ are the number of distinct interfaces of the AU for which at least 1 acceptable‐, medium‐, and high‐ quality model respectively, was submitted among the top 5 ranking models. The values of the weights “*ω*” were taken as *ω*_*1*_ = 1, *ω*_*2*_ = 2, and *ω*_*3*_ = 3. For ranking the performance of individual groups across all targets we used the normalized version of Equation (1): $<{\text{Score}}_{\mathrm{AU}}>=\frac{1}{K}\;\left({\text{Score}}_{\mathrm{AU}}\right)$, where *K* is the number of evaluated interfaces. However, in cases of higher‐order symmetric oligomers displaying identical or closely similar interfaces, the quality of the best model was taken to represent the model quality of the corresponding AU. These strategies were implemented to avoid large assemblies with multiple interfaces weighing too heavily on the global score of individual groups (Score_G_ of Equation (4) below).

In addition, interfaces were evaluated using the DockQ score as follows^47^:

(2) $$\text{DockQ}=\left[f\left(\mathrm{nat}\right)+{\mathrm{rms}}_{\text{scaled}}\left(L_{\mathrm{rms}},d_{1}\right)+{\mathrm{rms}}_{\text{scaled}}\left(i_{\mathrm{rms}},d_{2}\right)\right]/3$$

With

(3) $${\mathrm{rms}}_{\text{scaled}}=1/\left[1+{\left(\frac{\mathrm{rms}}{d_{i}}\right)}^{2}\right]$$

where *f*(nat), *i_*rms, and *L_*rms are as defined above. The rms_scaled_ represents the scaled rms deviations corresponding to either *L_*rms or *i_*rms and *d*_*i*_ is a scaling factor, *d*_*1*_ for *L_*rms and *d*_*2*_ for *i_*rms, which was optimized to fit the CAPRI model quality criteria, yielding *d*_*1*_ = 8.5 Å and *d*_*2*_ = 1.5 Å (see reference^47^).

For targets representing higher order oligomers featuring multiple distinct interfaces the quality score for each AU was computed as the average of the DockQ scores of the distinct interfaces that are part of the AU. For higher‐order oligomers, displaying identical or closely similar interfaces, the best DockQ score was taken to represent the model quality of the corresponding AU.

There is no strict correspondence between the DockQ values of predicted interfaces and the four CAPRI model quality categories, because the DockQ score employs a somewhat different algorithm to combine the three quality‐metrics (*f*(nat), *L_*rms, and *i_*rms) into a single continuous score than CAPRI uses to define its discrete model quality categories.

To further evaluate the accuracy of the modeled protein–protein interface we also computed the root mean square deviation of sidechain atoms (*S‐*rms) of residues at the binding interface, which was however not used to rank performance. This measure uses the backbone rms fit of the *i_*rms calculation, to compute rms values over side‐chain atoms only. It is not used in the classification of models.

The performance of predictor and scorer groups and servers was ranked based on their best‐quality model in the 5‐model submission for each target. The final score assigned to a group or a server was expressed as a weighted sum, analogous to that of Equation (1), but considering the performance for individual targets, expressed in each of the three categories (acceptable, medium, and high), achieved by that group or server over all targets:

(4) $${\text{Score}}_{G}=\omega_{1}N_{\mathrm{ACC}}+\omega_{2}N_{\mathrm{MED}}+\omega_{3}N_{\text{HIGH}}$$

where *N*_ACC_, *N*_MED_, and *N*_HIGH_ are the number of targets/AUs of acceptable‐, medium‐, and high‐ quality, respectively, and the values of weights “*ω*” were taken as *ω*_*1*_ = 1, *ω*_*2*_ = 2, and *ω*_*3*_ = 3.

This ranking method was already used in the two previous CASP‐CAPRI challenge^30^, ^42^ and previous CAPRI assessments.^49^ It considers all models of acceptable quality or higher submitted by a given group. For larger assemblies with more than 1 AU, it considers the model quality as defined by the value of <Score_AU_ > for the different AUs defined above.

Groups were also ranked using the sum of the DockQ values computed for all their best‐ranking models for each target or AU. Furthermore, rankings based on the Z‐score values across predictor groups of the CAPRI‐scores, and DockQ‐based scores were computed, and the different scoring schemes were compared to evaluate the robustness of the ranking procedure.

The CAPRI ranking methods, whether based on the CAPRI score or on DockQ, rank group performance by the overall quality of the modeled interfaces that each predictor group produces for each target/AU. This quality is evaluated by measuring the extent to which individual models submitted by each predictor group reproduce the target binding mode, based on a combination of three key quality metrics (*f*(nat), *i‐*rms, *L‐*rms) taken together. This approach differs from the CASP raking method. The latter is based on evaluating four quality scores for each model (Interface Contact Score (ICS), Interface Patch Score (IPC), TM score, oligomeric IDDT) [Ozden & Karaca, this issue]. These scores are not used to define the overall quality of each submitted model and rank groups according to this quality. Instead a Z‐score is computed independently for each of the four quality scores across all groups submitting models for a given target. These Z‐scores (limited to positive values) are summed across targets for each predictor group, and a weighted average of this sum is used to produce the final rank. This ranking method has been shown to produce only minor differences relative to the CAPRI ranking, especially for top performers, as also confirmed in the present challenge [Özden & Karaca, this issue] and is therefore a valid alternative. However, one consequential difference is that the performance of a given group does not necessarily reflect the overall quality of the models it produces. Accordingly, there currently is no overall CASP quality‐score for a predicted assembly that can be used to benchmark prediction methods.

## 5 RESULTS AND DISCUSSION

This section is divided into four main parts. The first part presents the results of human predictors, prediction servers, and CAPRI scorer groups (human and servers) for the individual 37 targets (38 AUs) of the 5 target categories of CAPRI Round 54, for which the prediction and scoring experiments were conducted. In the second part we present the rankings of the same groups established based on their performance across all targets. The third part analyses the prediction results across all the targets (AUs) of this Round, and the fourth part evaluates and discusses the progress that has been achieved.

### 5.1 Predictor server and scorer results for individual targets

The detailed prediction results per target obtained by CASP and CAPRI groups (predictors and servers) and CAPRI scorer groups can be found in Tables S2 and S3 of the Supplementary Material. An independent evaluation of the performance of predictor and server groups that submitted models only to CASP is presented in a separate publication [Ozden & Karaca, this issue]. Values of all the CAPRI quality assessment measures for individual models submitted by CAPRI participants for the 37 targets (38 AUs) of CAPRI Round 54 have been communicated to the participants and posted on the CAPRI website (URL: capri-docking.org). Additional information on the performance of individual CAPRI groups can be found in the Supplementary Material (Individual Group Summaries).

Given the large number of targets as well as the higher overall prediction performance in this prediction Round compared to previous Rounds, we present a brief account of the prediction results for the targets that were in general well predicted and discuss in further detail the performance of groups for the less well‐predicted complexes. This approach is applied to targets in the five target categories presented in the Targets section. To enable comparison with the models predicted by AF2 we also evaluated the quality of the best model predicted for each target by the AF2‐M software (run using standard parameters) and generously offered to this challenge by the group of Elofsson.

### 5.2 Category I: Homomeric targets with one interface (A2) and no intertwining: T198, T201, T211, T225, T226, T229

Of the 6 targets in this category, the best prediction performance across groups was obtained for T201/T1132, the bacterial antibiotic biosynthesis monooxygenase for which an excellent template was available. For this target, 54 groups (predictors and servers) produced acceptable models or better, with 52 of these groups submitting high quality models. Next in line is T211/H1153. For this target, assumed to be difficult because of its loopy interface and the availability of only a distant template, 48 groups submitted acceptable models or better, with 25 of these groups submitting high quality models and 21 groups submitting medium quality ones.

For T225/T1178 and T226/T1179, two related complexes displaying different binding modes, better performance was obtained for T225/T1178, with 57 groups producing models of acceptable quality or better. Of these, medium quality models were produced by 45 groups and high‐quality ones by only 9 groups. For T226/T1179 on the other hand, 48 groups produced acceptable models or better; of these only 6 and 2 groups produced medium and high‐quality models, respectively. The two best‐performing predictor groups for T226/T1179 were Wei Zheng and Toshiyuki Oda‐PEZYFoldings. Servers produced lower quality models for this target, with only 2 servers (DFOLDING‐SERVER, DEFOLDING‐REFINE) submitting medium quality models and 16 servers submitting acceptable models. CAPRI scorers (11 groups out of the total of 15) produced only acceptable models for this target.

The two most poorly predicted targets of this category were T198/T1123, the viral Capsid polyprotein VP90, and T229/T1187, the nictaba plant protein. The worst prediction results were obtained for T229/T1187. Only 16 groups and servers produced acceptable quality models or better. Fourteen of these groups produced high‐quality models, whereas only one group submitted a medium‐quality model. The best performing group for this target were Xinqi Gong‐BeijingAIProtein and Qiwei Ye‐UltraFold, with an additional 7 predictor groups producing high quality models, whereas only 4 servers performed on par with these groups (Table S2). For T198/T1123, despite the good model predicted by AF2 and a distant template for the full complex, only 30 groups produced acceptable quality models or better; of these, 19 groups submitted medium quality models, and none of the groups produced high‐quality models. The 4 best performing groups for this target were J Cheng, Wei Zheng, Jianyi Yang, and Baker. Five servers, including three versions of the MULTICOM server, and two of the DFOLDING server, respectively by the groups of J Cheng and Paek, performed on par with the best predictor groups. This was also the case for the CAPRI scoring server LZERD (Kihara group).

### 5.3 Category II: Homomeric targets with one interface (A2/A3) and intertwining: T194, T197, T199, T213, T214, T222‐T224, T227, T192, T193

For the 11 targets of this category, the best prediction performance was obtained for the dimers T192/T1109 and T193/T1110, the mutant and *wt* forms of the bacterial putative transcription regulator protein, and for the T199/T1127 dimer of the plant acetyltransferase for which multiple dimeric templates were available. For T192/T1109 and T193/T1110, respectively 54 and 56 groups (including predictors and prediction servers) produced models of acceptable quality or better, including respectively, 45 and 53 groups that produced high quality models. Even better performance was observed for T199/T1127, with 57 groups out of 65 submitting high quality models for this target. For all three targets, predictor and prediction server groups performed on par with AF2‐M (Table S2). The performance of scorers and scoring servers was excellent too but trailing behind that of the predictor and server groups for these three targets.

For the T194/T1113 dimer, only medium quality models were produced by 54 out of the 57 predictor/server groups that submitted correct models for this target, and by AF2‐M, whereas one predictor group (Jianyi Yang) produced a high‐quality model (*i‐*rms <1.0 Å). As expected, only medium quality models were submitted by the 12 scorers and scoring servers that submitted correct models for this target. Significantly lower quality models were produced by predictors and CAPRI scorers for the remaining four dimers: T197/T1121, T213/T1160, T214/T1161, and T224/T1176. Only incorrect models were submitted for T224/T1176, the domain‐swapped dimer from Clostridioides difficile. For T197/T1121, the cryo‐EM structure of the protein from pseudomonas containing DUF3322 and DUF2220 connected by a flexible linker, five predictor groups, two prediction servers, and one scorer group managed to produce acceptable models, whereas the model produced by AF2‐M was incorrect. Of the two small proteins T213/T1160 and T214/T1161, designed using ancient protein reconstruction, only two predictors (S_Huang, and Toshiyuki Oda‐PEZYFoldings and one server (HDOCK) produced one medium‐quality structure for T213/T1160, the version with the very small interface, whereas scorers produced only incorrect models. The T214/T1161, the variant containing mutations that increased the size of the subunit interface, seven predictor groups and three prediction servers produced acceptable models, with three of the predictor groups (Kozakov, Kozakov‐Vajda, Wallner), one server (CLUSPRO), and one scorer group (Venclovas) producing a high‐quality model. The model produced by AF2‐M was incorrect (not included in Table S2 for this target).

Of the 3 trimers (T222/T1173, T223/T1174, T227/T1181), the lowest quality models were submitted for T223/T1174, the intertwined bacterial trimer, another example of a protein containing several domains (here, 3 or 4) linked by flexible chain segments. For this target, 48 predictor groups (including servers) produced acceptable models. Of these, only one predictor (Kihara) and one server (DFOLDING‐SERVER), produced a medium‐quality model, which none of the scorer groups, including Kihara, were able to identify. Much better results were obtained for T222/T1173, and T227/T1181. As many as 56 groups submitted acceptable models for T222/T1173, the cell wall surface anchor family protein featuring 2 domains, with 21 and 25 of these groups submitting high‐ and medium‐quality models, respectively, and 12 scorer groups also submitting such models. Predictors groups likewise performed adequately for T227/T1181, the multi‐domain tail fiber protein viral protein. Of the 53 predictor and server groups submitting acceptable models or better, 31 submitted medium‐quality models and only three groups (Wei_Zheng, Kihara, Toshiyuki Oda‐PEZYFoldings) managed to produce a high‐quality model. Not too surprisingly, only medium quality models were produced by 10 out of the 13 CAPRI scorer groups who produced correct models.

Thus, the main difficulty in this target category arose in cases were the complexes displayed a significant degree of intertwining, sometimes compounded by the presence of multiple structural domains linked by flexible fragments. The presence of several disordered highly flexible regions, or small binding interface were likewise adversely affecting prediction results.

### 5.4 Category III: Heteromeric targets with one interface (A1/B1): T191, T200, T202, T210, T212

The best prediction results for the 5 heterodimers in this category were obtained for T191/H1106, T202/H1134, and T210/H1151. For T191/H1106, the YscX and YscY proteins of the Yop protein translocation/chaperone protein complex, 70 predictor and server groups submitted acceptable models or better, with 48 and 21 of these producing high‐ and medium‐quality models, respectively. Considering the significantly disordered character of the YscX component (Figure 3A), these were unexpectedly good prediction results, likely due to the wide use of AF2‐M, which also produced a high‐quality model for this target (Table S2). Excellent performance was also achieved for T210/H1151, the bacterial SigA/WhiB6 complex, for which an excellent quality template for the full complex was available, enabling a record number of predictors and servers (63), and 8 scorer groups, to submit high‐quality models. Adequate prediction results were also obtained for T202/H1134, the Ankyrin repeat protein/phospholipase complex, with only 22 predictor and servers submitting high quality models, but 38 additional groups including AF2‐M producing medium‐quality ones. Of the 13 scorer groups submitting correct models for T202/H1134, 9 and 4 scorer groups submitted medium‐ and high‐quality models respectively.

Prediction results were relatively poorer for T212/H1157, and very poor for T200/H1129. For T212/H1157, the Alpha‐1,2‐mannosidase/protein disulphide‐isomerase complex, 63 predictors and servers produced correct models, of which 61 produced medium‐quality models. Such models were also produced by the 14 scorer groups producing correct models for this target. For T200/H1129, the FhuA/pb5 phage host complex, only 20 predictor and server groups managed to produce correct models, with only five of these, three predictor groups and two prediction servers, producing high‐quality models. The three best performing predictors were Jainyi_Yang, Venclovas and Wallner, and the two servers were YANG_MULTIMER, and TS317. Surprisingly, 12 CAPRI scorers and scoring servers produced models of acceptable quality or better. As many as seven of these groups (five human scorers Kihara, Venclovas, Zou, S_Huang, and Bonvin), and two servers (MDOCKPP, HDOCK) submitting high quality models.

### 5.5 Category IV: Heteromeric Ab and Nb complexes with one interface (A1/B1 or A/HL): T205‐T209, T216‐T218

The eight targets in this category include five complexes of the mouse 2′,3′‐cyclic‐nucleotide 3′‐phosphodiesterase bound respectively to five different Nb (T205‐T209/H1140‐H1144) (Figure 3C), and three complexes of the S24‐188 Fab antibodies binding to somewhat distinct epitopes of the SARS‐COV‐2 nucleoprotein (T216‐T218/H1166‐H1168) (Figure 3D). With few exceptions, prediction results were rather poor for all 8 targets.

For the Nb complexes good results were achieved for T208/H1143, with 57 predictor groups producing models of acceptable quality or better, and 47 of these including servers submitting high‐quality models. Significantly poorer results were obtained for T206/H1141 and T209/H1144, for which respectively only 12 and 14 groups produced acceptable models or better. Of these, only four human predictors produced high‐quality models for T206/H1141. For T209/H1144, high quality models were produced by five human predictors and two prediction servers, outperforming most of their peers, as well as AF2‐M. The best performing groups for T206/H1141 were Venclovas, Wallner, Toshiyuki Oda‐PEZYFoldings and David_Jones‐DM. Venclovas was the only scorer group producing a correct model (of high quality) for this target. For T209/H1144, the best performers were Wei Zheng, Toshiyuki Oda‐PEZYFoldings, Suwen Zhao, Wallner, Jianyi Yang, and the YANG‐MULTIMER server, while AF2‐M produced an incorrect model. The worst results were obtained for the Nb complexes in T205/H1140 and T207/H1142. For T205/H1140, only seven predictor groups produced models of acceptable quality with two of these groups (Wei_Zheng and Wallner) submitting medium‐quality models. Of the scorer groups, only the group of Oliva managed to submit one medium‐quality model. This is interesting, because only CAPRI groups contributed models to the uploaded set from which scorers single out the best models. But none of the CAPRI predictor groups submitted models of acceptable quality or better among their five top‐ranking solutions for this target. Hence Oliva managed to single out a lower ranking medium‐quality model in the uploaded set that none of the CAPRI predictors identified. For T207/H1142, no correct models were submitted.

Of the 3 Ab complexes, adequate prediction results were obtained only for T218/H1168, the only complex for which a template with the correct binding mode was available. Sixty‐three predictor groups produced models of acceptable quality or better for this target, of which 53 groups and AF2‐M, submitted medium quality models, and only two groups, one human predictor (Junlin Wang‐MUFold_H) and the DFOLDING‐SERVER produced high‐quality models. In total 11 CAPRI scorer groups produced medium quality models. No correct models were submitted for T216/H1166 and T217/H1167.

Overall, we see that for this type of complexes, where one of the components is a Nb or an Ab, prediction results tend to be rather poor except for cases where template(s) with the same of similar binding models are available. As already noted previously,^34^ AF2‐M preforms particularly poorly with these complexes as well, unless help from templates is available. From the results obtained here, complexes with Nbs seem to be easier to predict than those with Abs, but this needs to be further confirmed on a larger number of complexes of both types.

### 5.6 Category V: Large assemblies: T195, T203, T204, T119, T220, T221, T230

Targets in this category comprised multi‐subunit homo and hetero higher‐order oligomers.

For these targets the prediction quality of all distinct interfaces was evaluated. In complexes featuring multiple distinct interfaces, the full complex was subdivided into two or more assessment units (AUs), with each AU containing one or more distinct interfaces. The CAPRI score for each AU was computed using the Score_AU_ expression of Equation (1). The results of predictor server and scorer groups for individual interfaces of each assembly target can be found in Table S2 of the supplementary material. The performance of these groups for individual AUs of each of target is provided in Table S3. For the assemblies featuring multiple distinct interfaces we will discuss the prediction results only for individual AUs.

Very different results were obtained for the two higher order homo‐oligomer targets T195/T1115 and T230/T1192 featuring a single unique interface. For T195/T1115, the 16‐mer stomatin complex, featuring a large distinct primary interface, and for which a good‐quality template was available for the individual subunits (2.4 Å rmsd), prediction results were disappointing. Only 24 predictor and server groups managed to produce a model of acceptable quality or better and of these, 11 groups (including AF2‐M) submitted a medium‐quality model. In comparison, the performance of scorer groups was better, with a 13 CAPRI scorers (virtually everybody) submitting acceptable models and five of these groups producing medium‐quality ones. The problem with this target was likely due to the available template for the stomatin subunit being part of a 24‐mer assembly and predictors failing to model the conformational adjustments that were required to fit the subunit into a smaller oligomer. On the other hand, rather good results were obtained for T230/T1192, the decamer featuring the human RAD52 homolog solved by cryo‐EM, for which an excellent template was available. For this target, 45 groups produced at least acceptable models, with 40 of these submitting high‐quality models. CAPRI scorers likewise performed well on this target.

Target T203/H1135, the trimer of trimers of the human SUN1 protein that binds a small peptide, was subdivided into 2 AUs, AU203.1 and AU203.2 (Figure 4): AU203.1 groups the three distinct interfaces (1–3) of the peptide‐bound trimer. AU203.2 includes the weaker distinct inter‐trimer interface (interface T203.4). For AU203.1 excellent prediction results were obtained for the major interface (interface 1), whereas poorer quality models were submitted for interfaces 2 and 3 (Table S2), resulting in most predictor groups submitting acceptable models for AU203.1 and only one group (Fang_Bai) submitting a medium‐quality model for this assessment unit (Table S3). Prediction servers and CAPRI scoring groups likewise managed to produce only acceptable models for AU203.1. Somewhat better results were obtained for AU203.2 (the smaller inter‐trimer interface 4). Of the 16 predictor groups (including AF2‐M) submitting at least acceptable models for this AU, four groups (S_Chang, Shan Chang‐CoDock, Venclovas, Kazuki Yamamoto‐ddquest) produced medium‐quality models. Of the eight CAPRI scorer groups producing at least acceptable models, only S_Chang produced a medium‐quality one. Thus, as for the T195/T1115 assembly, prediction methods including AF2‐M failed to adequately tackle the conformational adjustments in AU203.1, also affecting their ability to correctly model the inter‐trimer interface of AU203.2.

Target T204/H1137, the large multi‐component assembly composed of an ABC transporter and a MlaD hexamer that extends into 6 long helices forming a flexible tube‐like structure, was divided into two assessment units, AU204.1 and AU204.2, representing two distinct modeling problems (see Figure 5). AU204.1, which includes the interfaces between neighboring chains in the tube‐like structure (together taken as interface T204.1) and the C‐terminal domain, represents the more difficult modeling problem due to the flexible nature of the protein chains and the availability of only poor templates. For this AU, prediction performance was evaluated by the best predicted interface among the interfaces of the helical tube, and the interface of the C‐terminal domain. Only 22 predictor and server groups submitted models of acceptable quality or better for AU204.1, with only three predictors (Wei_Zheng, Xuyang Liu‐Manifold, and Kozakov) and two servers (CLUSPRO, MANIFOLD‐E) producing medium‐quality models. Only two CAPRI scorer groups (S_Chang and J_Cheng) submitted medium‐quality models out of the 14 CAPRI scorer groups producing acceptable models or better.

Better results were obtained for AU204.2, which included the transmembrane domain formed by an ABC transporter and the MlaD hexamer for which the organization was well known. This AU included the remaining six interfaces of the assembly, which were grouped into a total of eight ‘super’ interfaces (Figure 5). A total of 38 predictor and server groups produced at least acceptable models, including 14 groups submitting medium quality models for this AU (Table S3). Most of the 15 CAPRI scorer groups identified one of the medium quality models in the set uploaded by CAPRI predictors.

The remaining three large assembly targets, T219/T1170, T220/H1171, and T221/H1172, representing 3 closely related complexes, were evaluated together, and grouped into 2 distinct AUs: AU219, and AU220 (see Targets Section and Figure 6). AU219 groups the RuvB apo‐hexamer from all three complexes and comprises three unique “super” interfaces (interfaces 1‐3) featuring tighter to looser packing. These three “super” interfaces were evaluated separately, and the best model of all three submitted by each group was taken to represent the prediction result for this AU. AU220 represents the RuvB:RuvA complex and comprises interface 4, which groups the closely similar interfaces in T220/H1171 and T221/H1172 of the more loosely packed RuvB subunits with RuvA. Here too, the best models for this interface in the two complexes (T220/H1171 with one bound RuvA molecule and T221/H1172 with two bound RuvA molecules) were taken to represent the prediction results for AU220.

Despite the availability of good templates for the RuvB hexamer, only medium quality models were produced by all groups for the super interfaces 1‐2): 67 predictors, servers, and 14 CAPRI scorer and scoring servers (Table S3). Poorer prediction results were obtained by most groups for the super interface 3, the interface between the more loosely packed RuvB subunits (Table S3). Better prediction results were achieved for AU220. Fifty‐four predictor and server groups produced models of acceptable quality or better for this AU, with 24 of these groups producing medium‐quality models and 18 groups submitting high‐quality models.

Interestingly, higher quality models were submitted for the RuvB/RuvA interfaces in T221/H1172, which binds two RuvA molecules, than in T220/H1171, which binds only one (Table S3). This was likewise the case for the CAPRI scorer groups.

### 5.7 Performance of predictors, servers, and scorers across targets

Groups (predictors, servers, and scorers) were ranked according to their prediction performance for the 37 targets (38 AUs) of Round 54, using both the CAPRI score and the DockQ score. All the rankings presented here consider, as usual, the best model submitted by each group among the 5 top ranking models for each evaluated target or AU. The groups CAPRI‐score was computed using Equation (4) (see Assessment Metrics and Procedures). For the group DockQ‐based score, the DockQ scores of individual targets or AUs was summed.

**FIGURE 7:**
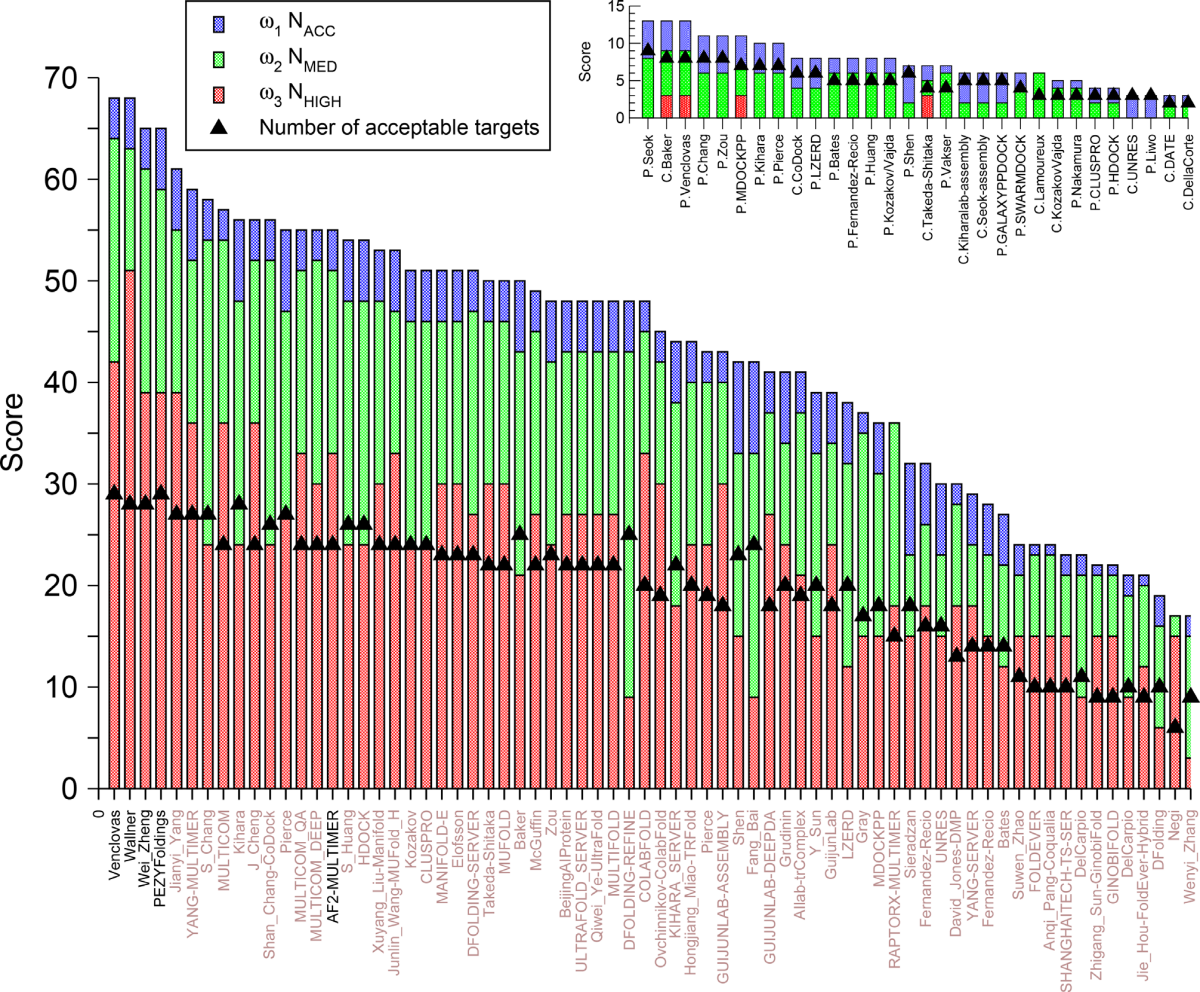
Performance of CASP and CAPRI predictor groups and prediction servers. The main graph shows the CAPRI performance scores of the top‐ranking CASP and CAPRI predictor and server groups in the CASP15‐CAPRI challenge evaluated here, broken off at the 70th group in the rank; server groups are listed in capital letters. The smaller graph shows the same plot for CASP and CAPRI predictor and server group 2 years earlier in the CASP14‐CAPRI assembly prediction challenge, broken off at the 23rd group. For both graphs, the height of each colored bar corresponds to the CAPRI score contributions of high, medium, or acceptable‐quality models. The total number of assessment units (AUs) for which at least an acceptable quality model was produced is indicated in the graph by a black triangle. CASP15‐CAPRI counted 38 AUs for 37 targets, CASP14‐CAPRI counted 16 AUs for 12 targets.

**FIGURE 8:**
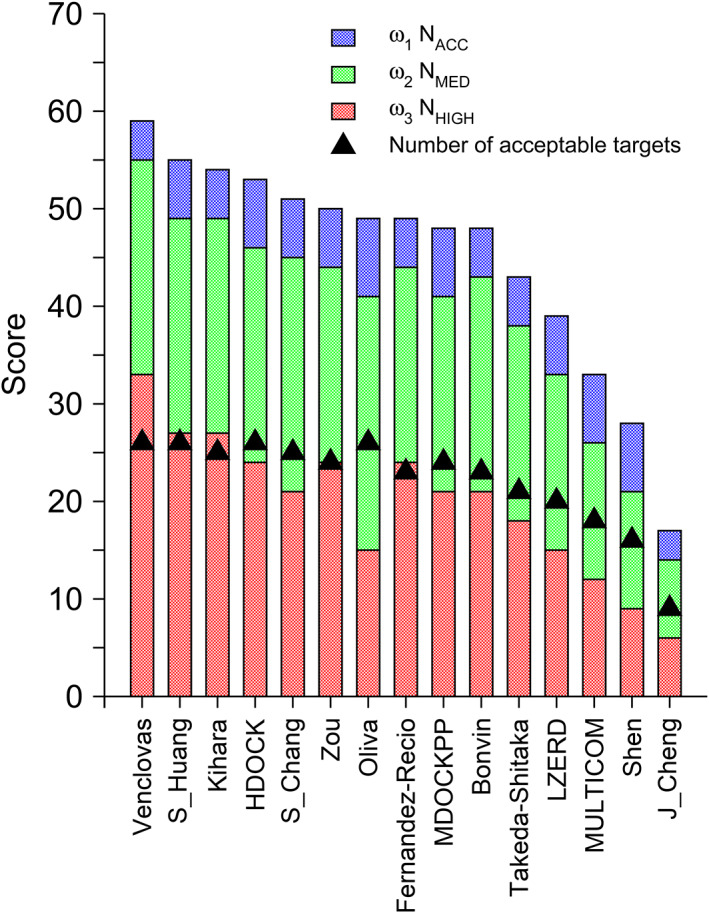
Performance of CAPRI scorer groups and scoring servers. CAPRI performance scores for all scorer groups, with scoring servers listed in capital letters. Legend as in Figure 7.

The ranking based on the CAPRI score of CASP and CAPRI (predictors and server) groups is shown in Figure 7, and the corresponding ranking of CAPRI scorers and scoring servers is displayed in Figure 8. The full ranked list of the different categories of participants, together with the corresponding CAPRI scores and DockQ scores is provided in the Table S4.

#### 5.7.1 Predictor and server performance

The ranking of the 96 participants of CASP15‐CAPRI shows several interesting trends (Figure 7). We see that the CAPRI scores of individual groups decrease rather monotonically as one moves down the rank, with only a small gap separating the slightly higher scores of the 4 best performers: Venclovas, Wallner, Wei Zheng, and PEZY‐Foldings. This is in stark contrast with the situation in CASP14, where one participant (AF2) clearly outperformed its peers in the 3D structure prediction of single‐chain proteins and indicates that an analogous game‐changing performance has not been achieved in CASP15 for the assembly prediction problem.

This notwithstanding, the ranking plot clearly indicates that an incremental, yet notable progress has been achieved over the standard performance of AF2‐M, which appears in the 15th position of the ranked list of Figure 7. As many as 14 groups of predictors and servers submitted higher or similar quality models than those produced by AF2‐M, as already noted in the section of the *Results for Individual Targets*. These groups include 6 CASP groups, including top‐performers Wei_Zheng, PEZYFoldings and Yang, and several veteran CAPRI groups (Venclovas, Chang, Kihara, Cheng, Pierce). It is furthermore noteworthy that 4 prediction servers from two groups (Jianyi Yang: YANG‐MULTIMER, J_Cheng: MULTICOM, MULTICOM_QA, and MULTICOM_DEEP) are among the servers outperforming AF2‐M (see also Table S4).

Another striking observation is the large fraction of respectively high‐ and medium‐ quality models contributing to the computed scores of most participants, with models of acceptable quality contributing only marginally. This trend confirms that the quality of the structures predicted in the present challenge is in general quite high, and clearly much higher than the models submitted only—2 years earlier in the CASP14‐CAPRI challenge (inset, top of Figure 7), where high‐quality models were submitted by 4 out of the total of 23 predictor and server groups, and even for these group such models were submitted for only a small fraction of the targets.

Any given ranking method inevitably suffers from inherent biases. To evaluate the extent to which such biases may affect the global ranking presented here we examine 2 alternative ranking methods based on the DockQ scores. One ranks groups using the sum of the DockQ scores (∑DockQ), computed over the best models submitted for the 38 AUs by predictors and servers (Figure S1). The second ranks groups using the ∑DockQ Z‐score computed across group, considering only positive Z‐scores (Figure S1).

The ∑DockQ scores of Figure S1 display analogous trends to those of the CAPRI ranking. The values decrease smoothly as one moves down the rank, with a small gap separating the slightly higher scores of the 4 best performers from those of their peers. These 4 top groups are the same as those of the CAPRI ranking, albeit in a slightly different order, whereas the order of groups further down the rank changes more significantly but is of minor importance given the very smooth decrease in the score values. The most salient difference with the CAPRI ranking is the rank of AF2‐M. The latter moves further down the rank to position 24 (instead of position 15 in the CAPRI rank), and hence a larger number of groups appear to outperform it. This is due to the fact the group score also includes contributions from incorrect models (roughly corresponding to DockQ values below 0.23), which the CAPRI score ignores, thereby boosting the performance or groups submitting incorrect models. Indeed, the ranking plot of Figure 7 also displays the number of AUs for which a given group submitted correct models (acceptable quality or better), showing that the number of correctly predicted targets decreases significantly from 30 to about 10 along the ranked list, corresponding to an increase in incorrect models from 8 to ~28. A similar behavior is displayed by the ∑DockQ Z‐score plot (Figure S1), which ranks a group relative to its peers. Again, the same 4 groups rank on top, with more significant changes further down the rank relative to both the CAPRI and ∑DockQ rankings, and the position of the AF2‐M performance moving much further down the rank to position 44. Hence, including a non‐zero contribution from incorrect models in the DockQ‐based evaluation introduces a misleading bias and should be avoided.

#### 5.7.2 Scorer performance

The ranking of the CAPRI scorer and server groups (15 in total) based on the CAPRI score (Figure 8), shows similar trends to those of the predictor performance, with a clear gap separating the score of the best performers (here the group of Venclovas) from those of other groups/servers. Scores of the following nine groups decrease very gradually thereafter but drop more swiftly for the five remaining scorer groups. Here too, high‐ and medium‐quality models make a major contribution to the computed scores of most participants, a clear consequence of the larger fraction of such models in the set of models uploaded by CAPRI predictor groups. Of the 4 participating scoring servers HDOCK performed best, followed by MDOCKPP, whereas LZERD and MULTICOM ranked last.

Lastly, it is noteworthy, that the data on the global group ranking (Tables S4) indicate that most predictor groups have improved their ability to rank models. The number of targets for which these groups have a model of acceptable quality or higher ranked on top (top‐1) is often only slightly lower than when their top‐5 ranking models are considered. It is noteworthy that this is also the case here for prediction servers, whereas scorers and scoring servers are less consistently successful in having their best quality models ranked on top.

### 5.8 Global overview of the quality of predicted models

**FIGURE 9:**
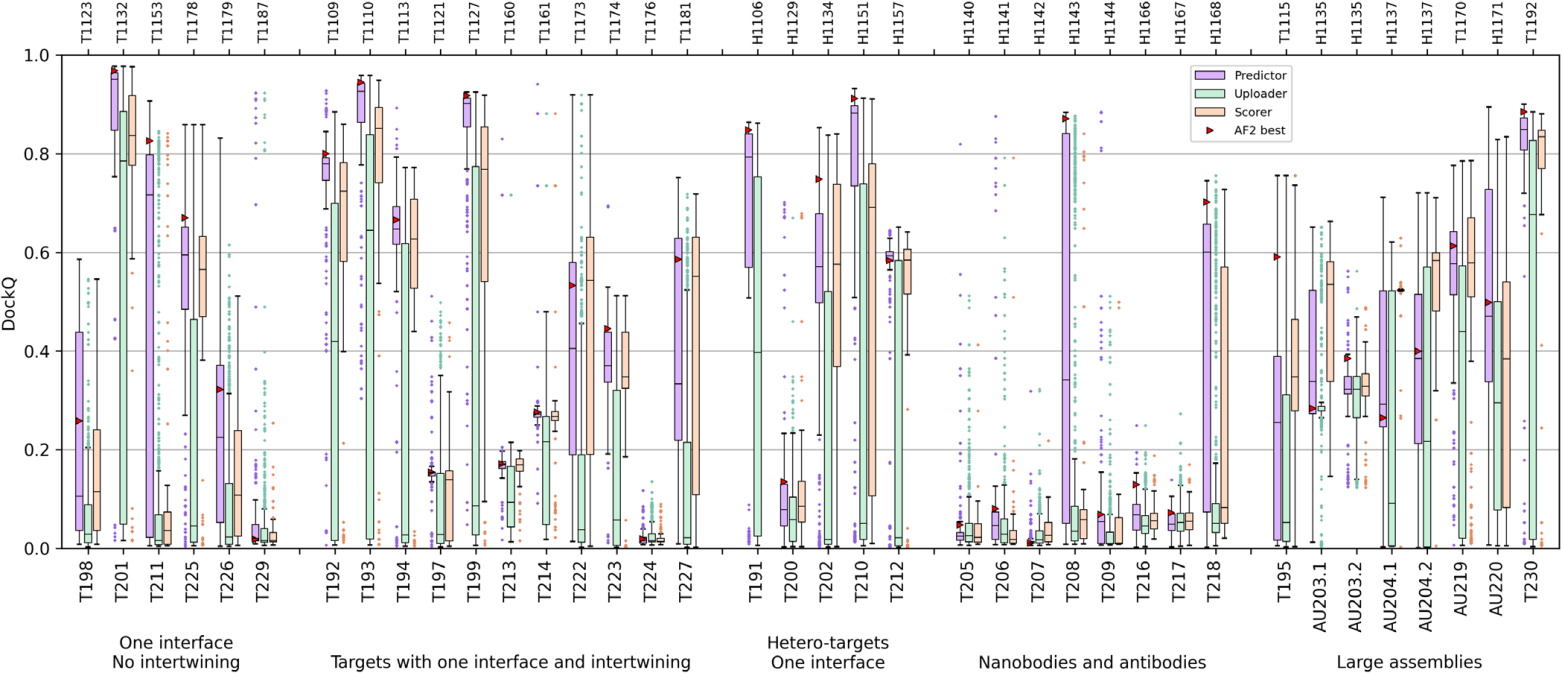
Global overview of performance for the 38 AUs (37 targets) of Round 54. Distribution of DockQ values of all the submissions of the Predictor ‐human and server groups‐ (purple; CASP and CAPRI; 5 models max), CAPRI Uploader groups (green; 100 models max), and Scorer groups (salmon; 10 models max). The best AF2‐M submission was taken as the standard for off‐the‐bench tools and is indicated by a red triangle. The box plots follow the matplotlib 3.1.2 defaults, with boxes from the first to the third quartile and a line at the median; whiskers are located at the upper resp. lower quartile plus or minus 1.5 the interquartile range and flier points are those outside the whiskers. The lower horizontal axis indicates the CAPRI ID and the upper horizontal axis the target CASP ID.

A global overview of the quality of models submitted (the best model of the five submitted models by each group) for the targets in the five categories is presented in Figure 9. This Figure shows box plots of the DockQ value distributions of models submitted by CASP and CAPRI human predictor and prediction servers, those submitted and CAPRI scorer groups (including servers), as well as model uploaded by CAPRI predictors (100 models per target). To enable comparison with the models predicted by AlphaFold we also plot for each target the DockQ values for the best model predicted by the AF2‐M used here as the baseline to gauge overall performance relative to this impactful deep learning package. These data illustrate the performance‐based target difficulty levels experienced in practice.

For example, for dimers with no intertwining of Category I, the highest quality models were produced for the easy target T201/T1132 by CASP and CAPRI predictor groups (human and servers). These models feature a narrow distribution around high DockQ values (μDockQ >0.9, where μDockQ is the median of the DockQ distribution). A high‐quality model was also predicted by AF2‐M, whereas the models submitted by scorers tended to be of somewhat lower quality (a wider distribution of DockQ values around ~0.83). Medium to high quality models were obtained by predictors groups for T211/T1153 and T225/T1178, but much poorer quality models were produced by CAPRI scorer croups for the former target due to the lower quality models uploaded by the smaller number of CAPRI predictor groups for that target. The poorest quality models for targets in Category I were submitted for T229/T1187, closely followed by those of T198/T1123 and T226/T1179. These three targets hence stand out as the most difficult dimers of this category.

An analogous analysis can be carried out for targets in the other categories. Category IV, grouping the 5 Caspase/Nb complexes (T205‐T209/H1140‐H1144) and the three SARS‐Cov2 nucleoprotein/Ab complexes (T216‐T218/H1166‐H1168) stands out by the poor quality of the submitted models. The box plots of Figure 9 clearly illustrate the general poor quality of the models (μDockQ ~0.0) produced for the Nb complexes, except for the Nb complex of T208/H1143 for which a template was available. Of the 3 Ab complexes, better quality models were obtained for T218/H1168, for which also a template was available, whereas only incorrect models (μDockQ ~0.0) were produced for T216/H1166 and T217/H1167. Taken together these results underscore the challenge of predicting the structure of antibody and nanobody complexes.

The quality of the models produced for targets in the four remaining categories was more variable. For the dimers and trimers with intertwining (category II) low quality models (μDockQ <0.3) were in general produced by predictors and CAPRI scorers for T197/T1121, T213/T1160, T214/T1161, T223/T1174, and T224/T1176, with only incorrect models submitted for T224/T1176, a domain swapped bacterial dimer. The prediction performance thus singles out these four targets as the most difficult prediction problems in this category, in clear contrast with the dimers of T192/T1109, T193/T1110, and T199/T1127, for which the performance was much better overall.

Among the hetero‐dimers (category III), T200/H1129, the phage Ferrichrome/receptor binding protein pb5 complex solved by cryo‐EM stands out as the target with the lowest quality models overall, including the one by AF2‐M, and hence the most difficult targets in this category.

Lastly, for the 8 AUs making up the large assemblies solved by cryo‐EM of category V, the highest quality models obtained by all groups were for T230/T1192, the DNA repair protein RAD52 homolog decamer. Whereas the quality of models submitted for the remaining AUs spanned a wider range, mostly without reaching high quality for none of the AUs, except for AU220 that combines the RuvB/RuvA binding interfaces of targets T220/H1172 and T221/H1172. Thus, large multi component assemblies represent difficult targets for a variety of reasons (see Section 1, Category V).

**FIGURE 10:**
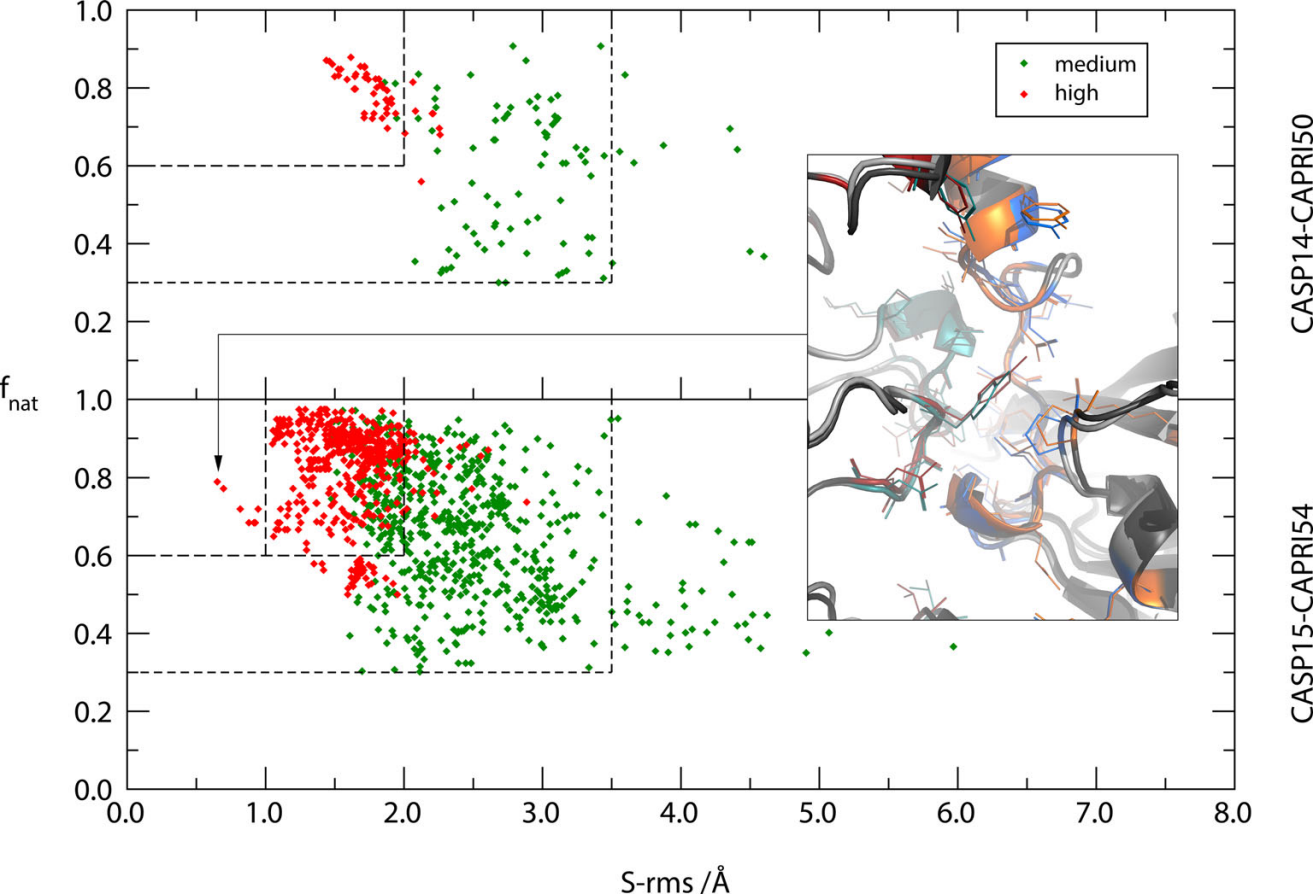
*f*nat (interface recall) as a function of *S‐*rms (side‐chain rms). Each point in the Figure represents the best model of a predictor group for an individual interface of a specific target, color‐coded according to model quality; incorrect and acceptable models are excluded. The top panel shows the data for targets of CASP14/CAPRI Round 50, the bottom panel for CASP15/CAPRI Round 54. The regions outlined by the short‐dashed and long‐dashed lines feature the best models, with medium‐quality models showing an *S‐*rms below 3.5 Å and *f*nat above 30%, and high‐quality models showing an *S‐*rms below 2.0 Å and an *f*nat above 60%. Some interfaces feature *S‐*rms values below 1.0 Å; those all belong to T203.1 (interface 1 of targets T203). The inset shows the interface of the model with the lowest S‐rms, produced by GuijunLab (target chains in red and orange, prediction chains in teal blue and marine blue; non‐interface residues in dark gray and light gray for target and prediction, respectively); interface residues—those that have any atom within 5 Å of the other chain—are shown in wireframe.

An important measure of the quality of the predicted structure of a binding interface, which determines the type of downstream studies and applications that it enables, is the accuracy with which the conformations of interface sidechains are modeled. Since many more binding interfaces were predicted to high accuracy in this Round than in previous years it was reasonable to expect that the sidechain conformation in many of these interfaces would also be predicted more accurately. This is borne out by the scatter plot of *f*(nat), the residue‐residue contacts of the targets recalled in the structure of medium and high‐quality models of this prediction Round, versus *S‐*rms, the root mean square deviation of interface sidechain atoms (Figure 10). These plots show that most of the high‐quality models and a small fraction of medium‐quality models feature low *S‐*rms values between 1 and 2 Å, and that 6 models have *S‐*rms values lower than 1 Å, of which two models submitted for interface T203.1 by Xianming_Pan and GuijunLab display *S‐*rms values as low as ~0.65. The interface of the better of both models is illustrated in the inset of Figure 10.

### 5.9 The impact of AlphaFold: progress and remaining challenges

This assessment shows that significant progress has been achieved in the prediction of protein complexes and assemblies. This progress is not due to the substantially better performance of one group relative to other participants, as by AF2 for the prediction of single protein chains in CASP14 2 years earlier. This time it was achieved by a significant fraction of methods developers (>60), including groups that have never previously participated in any assembly prediction challenges. These groups produced high‐quality models for about 40% of the targets with which they were challenged, many more than in previous years (Figures 7 and 9). Furthermore, this performance appears to be robust enough since it was achieved on nearly 40 distinct modeling problems (38 AUs), a much larger number of complexes than offered as targets in previous years.

As reported at the CASP15 conference in December 2022, as well as in the extended Abstracts of individual groups co‐authors of this report (see Supplementary Material), this sizable improvement in performance across the community is undeniably due to the wide use of the AF2 and AF2‐M software and similar DL‐based methods. These methods were widely used to predict the 3D structure of individual components of the complex, and whenever possible, the structure of the full complex.

An important driver of this improvement has been the creative employment of these deep learning‐based inference engines, modifying them to sample a much larger number of models and/or using as input multiple sequence alignments augmented with sequences from various sources. Such strategies were most successfully implemented by several of the top performing groups that outperformed off‐the‐shelf versions of AF2‐M, used as a yardstick with which the performance of predictor groups was compared (Figures 7 and S1 and S2). For example, Wallner—one of the three top performers – stochastically perturbed the neural network of AF2‐M by enabling dropouts at the inference stage combined with massive sampling, generating up to 6000 models per target compared to the 25 models AF2‐M generates by default. This was done using different versions of the network models, with and without templates, and increasing the number of recycles within the network. The group of Wei_Zheng, another of the three top performers, used a set of procedures to generate MSAs for each component of a complex, and further augmented them with a vast number of sequences from various metagenome sequence databases. A subset of these MSAs was then used to build the paired alignments fed into AF2‐M. For hetero‐complexes the paired MSA were built by combinatorically combining the MSAs of individual components and using AF2‐M to sample a corresponding large number of models from which the top five models for each complex were selected. The performance of both approaches heavily relied on the AF2/AF2‐M confidence metrics (pLDDT, iPTM, pTM)^17^, ^33^ to prioritize and rank models, a practice backed by recent findings that these metrics closely estimate the true quality of the candidate structures, outperforming other state‐of‐the‐art model accuracy estimates.^50^ The group of Venclovas—another top performer—used both the AF2 pipeline and ColabFold,^23^ tweaking a variety of parameters and settings to achieve more extensive sampling of models for each target. While AF2 confidence metrics were also employed to evaluate models, the final model ranking and selection was performed using their powerful Voronoi tessellation‐based scoring procedures, shown to perform extremely well in singling out physiological dimers from the non‐physiological well in a recent community‐wide benchmark study.^51^ Interestingly, Wallner produced more high‐quality models than Wei_Zheng and Venclovas, but Venclovas submitted models for three more targets than Wallner, of which two were high‐quality and one medium‐quality (See Figure S2).

Interestingly, while wide use was made of AF2 and AF2‐M and their confidence metrics, state of the art docking algorithms and objective methods for scoring and ranking predicted interfaces, developed notably by members of the CAPRI community, also conferred an advantage. Docking was particularly helpful for modeling complexes with nanobodies and antibodies, compensating for the poor performance of AF2‐M for such complexes. For example, it helped the group of Pierce produce acceptable models for the Nb complexes of T205/H1140 and T206/H1141, for which AF2‐M totally failed. Docking also helped the Venclovas group build fairly accurate full models of some of the large assemblies of T195/T1115 and T203/H1135 from only partial models produced by AF2‐M.^52^ For smaller targets, it was not uncommon to use docking calculations in parallel to AF2‐M, to evaluate consistency between the models produced by the two methods (Venclovas), and in the case of inconsistent models to submit a mix of models produced by both approaches (Fernandez‐Recio) (see Supplementary Material; Individual Group Summaries). Not unexpectedly, the results on individual targets and the reports by individual groups confirm that the availability of good quality templates played an undeniable role.

This notwithstanding, highly accurate models were produced for only ~40% of the assembly targets, indicating that the accurate prediction of protein complexes and assemblies remains a challenge. A specific category of targets that prediction methods struggled with most were the complexes with Ab's and Nb's for which evolutionary signals cannot be extracted from multiple sequence alignments and classical docking algorithms also often perform sub‐optimally. Improving the prediction performance for this important category of complexes is a very active research area, involving the development of new data resources on antigen–antibody interactions^53^ and new deep learning methods exploiting structural and sequence data on experimentally determined protein binding sites.^54^

A long‐standing limitation of classical methods for modeling protein complexes is their inability to model the conformational flexibility of proteins.^30^, ^55^ The same problem plagues deep learning‐based structure prediction methods such as AF2^29^ and AF2‐M.^31^ It is therefore not surprising, that the prediction methods evaluated here in general performed poorly when complexes contained subunits with multiple globular domains linked by flexible segments (targets T197/T1121, T222/T1173, T223/T1174, T227/T1181) or when the binding interface included disordered segments (targets T192/T1109, T224/T1176). Accurately modeling the conformational adjustments of individual subunits necessary to correctly assemble higher‐order homo‐ or hetero‐oligomers, was likewise problematic (target T203/H1135). Recognized as a major unsolved problem, modeling protein flexibility and more generally, sampling the different conformational states that proteins adopt to carry out their function, is currently the focus of intense method development efforts. These efforts involve the design of promising novel deep learning approaches^56^ and methods that combine deep learning with physics‐based simulation methods.^57^ But these approaches deal with single protein chains. Extending them to consider the association process and the formation of complexes would be a worthwhile but challenging future undertaking.

## 6 CONCLUDING REMARKS

This assessment of the CASP15‐CAPRI assembly prediction challenge reports the results for 37 targets (38 AUs) spanning a range in target types and difficulty levels. These results were produced by over 60 CASP and CAPRI predictor groups including more than 20 automatic servers. The numbers of both assembly targets and groups were much greater than in previous challenges allowing for a robust evaluation of the prediction performance across the community.

Analysis of these results revealed substantial progress achieved across a significant fraction of the participating groups, with high‐quality models as judged by the CAPRI criteria produced for about 40% of the targets compared to 8% two years earlier. As highlighted throughout the analysis, this jump in progress may be attributed to the wide use of AF2 and AF2‐M. These deep learning tools were used in a variety of ways. They were used to predict the structure of individual components of a complex (AF2), the entire complex, or a portion thereof (AF2‐M), employing off the shelf or customized versions, with the latter often enabling much wider sampling of candidate conformations. Integrating predictions performed by AF2‐M with classical docking and scoring procedures was also not uncommon.

This wide adoption of the deep‐learning tools by the community and its very significant influence on the prediction performance had an undeniable impact on the factors that define the target difficulty level. While the absence of templates no longer systematically hampered accurate prediction, the availability of good quality templates still mostly facilitated prediction. As already mentioned, complexes with antibodies and nanobodies remain an important category of targets that current prediction methods struggle with. This was also the case of targets where structural features of the templates (subunit contacts and/or conformation) differed from those of the target, and where the target included proteins with flexible segments. That targets displaying a significant intertwining as in the homodimer of T224/T1176 also turned out to be poorly predicted is somewhat surprising since one would expect folding and binding to be strongly coupled in such complexes^58^ and therefore be more readily well predicted by the AF2 or AF2‐M, which model the conformations of both chains simultaneously.

Thus, the accurate prediction of protein complexes remains an important open problem, with considerable room for improvements. To better evaluate where improvements are necessary one also needs to objectively compare the errors in the high‐quality models produced by current methods to the uncertainties of experimentally determined structures, a difficult task so far attempted only for AF2 predicted structures of single protein chains.^59^

## AUTHOR CONTRIBUTIONS

**Marc F. Lensink:** Writing – review and editing; conceptualization; methodology; software; supervision; visualization; project administration; resources; writing – original draft; investigation; validation; data curation. **Guillaume Brysbaert:** Writing – review and editing; software; methodology; resources; supervision; investigation. **Nessim Raouraoua:** Writing – review and editing; software. **Paul A. Bates:** Writing – review and editing. **Marco Giulini:** Writing – review and editing. **Rodrigo V. Honorato:** Writing – review and editing. **Charlotte van Noort:** Writing – review and editing. **Joao M. C. Teixeira:** Writing – review and editing. **Alexandre M. J. J. Bonvin:** Writing – review and editing. **Ren Kong:** Writing – review and editing. **Hang Shi:** Writing – review and editing. **Xufeng Lu:** Writing – review and editing. **Shan Chang:** Writing – review and editing. **Jian Liu:** Writing – review and editing. **Zhiye Guo:** Writing – review and editing. **Xiao Chen:** Writing – review and editing. **Alex Morehead:** Writing – review and editing. **Raj S. Roy:** Writing – review and editing. **Tianqi Wu:** Writing – review and editing. **Nabin Giri:** Writing – review and editing. **Farhan Quadir:** Writing – review and editing. **Chen Chen:** Writing – review and editing. **Jianlin Cheng:** Writing – review and editing. **Carlos A. Del Carpio:** Writing – review and editing. **Eichiro Ichiishi:** Writing – review and editing. **Luis A. Rodriguez‐Lumbreras:** Writing – review and editing. **Juan Fernandez‐Recio:** Writing – review and editing. **Ameya Harmalkar:** Writing – review and editing. **Lee‐Shin Chu:** Writing – review and editing. **Sam Canner:** Writing – review and editing. **Rituparna Smanta:** Writing – review and editing. **Jeffrey J. Gray:** Writing – review and editing. **Hao Li:** Writing – review and editing. **Peicong Lin:** Writing – review and editing. **Jiahua He:** Writing – review and editing. **Huanyu Tao:** Writing – review and editing. **Sheng‐You Huang:** Writing – review and editing. **Jorge Roel‐Touris:** Writing – review and editing. **Brian Jimenez‐Garcia:** Writing – review and editing. **Charles W. Christoffer:** Writing – review and editing. **Anika J. Jain:** Writing – review and editing. **Yuki Kagaya:** Writing – review and editing. **Harini Kannan:** Writing – review and editing. **Tsukasa Nakamura:** Writing – review and editing. **Genki Terashi:** Writing – review and editing. **Jacob C. Verburgt:** Writing – review and editing. **Yuanyuan Zhang:** Writing – review and editing. **Zicong Zhang:** Writing – review and editing. **Hayato Fujuta:** Writing – review and editing. **Masakazu Sekijima:** Writing – review and editing. **Daisuke Kihara:** Writing – review and editing. **Omeir Khan:** Writing – review and editing. **Sergei Kotelnikov:** Writing – review and editing. **Usman Ghani:** Writing – review and editing. **Dzmitry Padhorny:** Writing – review and editing. **Dmitri Beglov:** Writing – review and editing. **Sandor Vajda:** Writing – review and editing. **Dima Kozakov:** Writing – review and editing. **Surendra S. Negi:** Writing – review and editing. **Tiziana Ricciardelli:** Writing – review and editing. **Didier Barradas‐Bautista:** Writing – review and editing. **Zhen Cao:** Writing – review and editing. **Mohit Chawla:** Writing – review and editing. **Luigi Cavallo:** Writing – review and editing. **Romina Oliva:** Writing – review and editing. **Rui Yin:** Writing – review and editing. **Melyssa Cheung:** Writing – review and editing. **Johnathan D. Guest:** Writing – review and editing. **Jessica Lee:** Writing – review and editing. **Brian G. Pierce:** Writing – review and editing. **Ben Shor:** Writing – review and editing. **Tomer Cohen:** Writing – review and editing. **Matan Halfon:** Writing – review and editing. **Dina Schneidman‐Duhovny:** Writing – review and editing. **Shaowen Zhu:** Writing – review and editing. **Rujie Yin:** Writing – review and editing. **Yuanfei Sun:** Writing – review and editing. **Yang Shen:** Writing – review and editing. **Martyna Maszota‐Zieleniak:** Writing – review and editing. **Krzysztof K. Bojarski:** Writing – review and editing. **Emilia A. Lubecka:** Writing – review and editing. **Mateusz Marcisz:** Writing – review and editing. **Annemarie Danielsson:** Writing – review and editing. **Lukasz Dziadek:** Writing – review and editing. **Margrethe Gaardlos:** Writing – review and editing. **Artur Gieldon:** Writing – review and editing. **Adam Liwo:** Writing – review and editing. **Sergey A. Samsonov:** Writing – review and editing. **Rafal Slusarz:** Writing – review and editing. **Karolina Zieba:** Writing – review and editing. **Adam K. Sieradzan:** Writing – review and editing. **Cezary Czaplewski:** Writing – review and editing. **Shinpei Kobayashi:** Writing – review and editing. **Yuta Miyakawa:** Writing – review and editing. **Yasuomi Kiyota:** Writing – review and editing. **Mayuko Takeda‐Shitaka:** Writing – review and editing. **Kliment Olechnovic:** Writing – review and editing. **Lukas Valancauskas:** Writing – review and editing. **Justas Dapkunas:** Writing – review and editing. **Ceslovas Venclovas:** Writing – review and editing. **Bjorn Wallner:** Writing – review and editing. **Lin Yang:** Writing – review and editing. **Chengyu Hou:** Writing – review and editing. **Xiaodong He:** Writing – review and editing. **Shuai Guo:** Writing – review and editing. **Shenda Jiang:** Writing – review and editing. **Xiaoliang Ma:** Writing – review and editing. **Rui Duan:** Writing – review and editing. **Liming Qui:** Writing – review and editing. **Xianjin Xu:** Writing – review and editing. **Xiaoqin Zou:** Writing – review and editing. **Sameer Velankar:** Resources; writing – review and editing. **Shoshana J. Wodak:** Conceptualization; methodology; supervision; project administration; writing – original draft; writing – review and editing; validation; investigation.

## CONFLICT OF INTEREST STATEMENT

The authors declare no conflicts of interest.

### PEER REVIEW

The peer review history for this article is available at https://www.webofscience.com/api/gateway/wos/peer-review/10.1002/prot.26609.

## Supporting information


**Data S1:** Supporting Information.

Table S5:

## Data Availability

The data that supports the findings of this study are available in the supplementary material of this article.
