# Supplementary material for: Impact of AlphaFold on structure prediction of protein complexes: The CASP15‐CAPRI experiment
Source: Proteins. 2023 Oct 31;91(12):1658–83. doi: 10.1002/prot.26609 (PMC10841881; doi:10.1002/prot.26609)
Supplement: Supplementary file 1 — Data S1: Supporting Information. [file PROT-91-1658-s002.pdf]

Impact of AlphaFold on Structure Prediction of Protein Complexes: The CASP15-CAPRI Experiment

Marc F. Lensink, Guillaume Brysbaert, Nessim Raouraoua,
all CAPRI participants,
Sameer Velankar and **Shoshana J. Wodak**

Supplementary Material

– Funding information **1**

– Table S1 – Participation statistics **3**
The Table lists for all targets – grouped together by target type – the number of Predictor and Scorer groups, with 'CASP' indicating CASP-only registrees. The number of submitted models is listed for CASP groups (limited to 5 per group) and CAPRI groups (limited to 100 per group). Scorer groups only had access to the CAPRI-submitted models. The table also shows the stoichiometry of the target and the number of interfaces in the assessment.

– Table S2 – Prediction results **4**
The Table lists for every interface the performance of human and server Predictor groups, and of Scorer groups (human and servers together). In the Table, server groups are listed in capitals. Results are listed as the number of submitted models of acceptable quality or better, with the number of higher than acceptable quality models listed after a slash, *e.g.* '5/4**' indicates that 5 models of acceptable quality or better were submitted of which 4 are of medium quality. Only the first five models of a submission were considered. Incorrect models are not listed.

  – Targets with one interface (A2) and no intertwining
  T198: **4**; T201: **5**; T211: **7**; T225: **8**; T226: **10**; T229: **11**

  – Targets with one interface (A2/A3) and intertwining
  T192: **12**; T193: **14**; T194: **16**; T197: **18**; T199: **20**; T213: **20**; T214: **21**; T222: **22**; T223: **24**; T224: **25**; T227: **25**

  – Hetero-targets with one interface (A1B1)
  T191: **27**; T200: **29**; T202: **30**; T210: **32**; T212: **34**

  – Targets with one interface (A1B1 or A:HL): nanobodies and antibodies
  T205: **36**; T206: **36**; T207: **36**; T208: **37**; T209: **38**; T216: **38**; T217: **38**; T218: **39**

  – Large assemblies
  T195: **41**; T203: **42**; T204: **47**; T219: **55**; T220: **61**; T221: **69**; T230: **77**

– Table S3 – Assessment Unit prediction results **78**
The Table lists for every assessment unit the quality of the best model for each of the interfaces. Results are listed for the groups as in Table S2. The last column shows the prediction quality for the assessment unit as a whole, using either the average or the best of the listed interfaces, this being target-dependent.

– Table S4 – Complete participant performance **87**
The Table lists the overall participant performance for the groups as in Tables S2 and S3. The columns 'Top-1' and 'Top-5' consider only the first, or first five submitted models in the calculation, showing the total number of assessment units for which a model of at least acceptable quality was submitted, indicating after the slash how many of these were of high or medium quality. The score accumates the 'Top-5' score following the formula as described in the main text (attributing a 3, 2 or 1 for a high, medium or acceptable quality model). The right-hand columns calculate alternate ranking possibilities, using either the cumulative DockQ scores, or the sum of positive Z-scores of the distribution of DockQ values. The alternative rankings are visualized in Supplementary Figure S1A and B.

– Table S5 – CASP15/CAPRI target master table **89**

– Figure S1 – Alternative ranking using DockQ or Z-scores. **90**

– Figure S2 – Relative performance of the four top-performing groups. **91**

– Individual Group Summaries - Methods employed by CAPRI participants **92**
Bates – **92**; Bonvin – **95**; Chang – **99**; Cheng – **101**; Del Carpio – **107**; Fernandez-Recio – **109**; Gray – **112**; Huang – **119**; Jimenez-Garcia – **121**; Kihara – **123**; Kozakov/Vajda – **126**; Negi – **129**; Oliva – **130**; Pierce – **132**; Schneidman – **135**; Shen – **138**; Sieradzan – **140**; Takeda-Shitaka – **142**; Venclovas – **145**; Wallner – **148**; Yang – **149**; Zou – **151**

Funding information

Bates — This work was supported by the Francis Crick Institute, which receives its core funding from Cancer Research UK (FC0001003), the UK Medical Research Council (FC001003), and the Wellcome Trust (FC001003).

Bonvin — Financial support from the European Union Horizon 2020, project BioExcel (823830) and from the Netherlands e-Science Center, project IVRESSE (027.020.G13) is acknowledged.

Cheng — This work was partially supported by US National Science Foundation (grant #: DBI1759934 and IIS1763246), and US National Institutes of Health (grant #: R01GM093123 and R01GM146340).

Fernandez-Recio — Spanish Ministry of Science (grant PID2019-110167RB-I00 / AEI /10.13039/501100011033).

Gray — This work was supported by National Institute of Health grant R35-GM141881. Computational resources were provided by the Advanced Research Computing at Hopkins (ARCH) core facility, supported by the National Science Foundation (NSF) grant number OAC1920103.

Huang — This work was supported by the National Natural Science Foundation of China to S.H. (Grant No: 62072199 and 32161133002).

Jimenez-Garcia — J.R-T. acknowledges funding from the European Molecular Biology Organization (EMBO) [ALTF 145-2021]; BJ-G. is employed by Zymvol Biomodeling on a project which received funding from the European Union's Horizon 2020 research and innovation programme under Marie Skłodowska-Curie grant agreement No. 801342 (Tecniospring INDUSTRY) and the Government of Catalonia's Agency for Business Competitiveness (ACCIÓ)

Kihara — We are grateful for ITaP Research Computing at Purdue University for providing us additional computational resources for this project. This work is partially supported by the National Institutes of Health (R01GM133840, R01GM123055), the National Science Foundation (CMMI1825941, MCB1925643, DBI2146026, IIS2211598, and DBI2003635). C.C. and J.V. were supported by a National Institute of General Medical Sciences -funded predoctoral fellowship to C.C. and J.V. (T32 GM132024).

Kozakov/Vajda — DMS 2054251 from the National Science Foundation, and R01GM140098, R35GM118078, RM1135136 from the National Institute of Health.

Oliva — This research was funded by the KAUST baseline research funding (to LC) and by MIUR-FFABR "Fondo per il Finanziamento Attività Base di Ricerca" (to RO).

Pierce — This work was supported by NIH R35 GM144083 (to BGP).

Shen — The study was in part supported by NIH/NIGMS (R35GM124952 to YS) and NSF (CCF- 1943008 to YS). Portions of this research were conducted with the advanced computing resources provided by Texas A&M High Performance Research Computing.

Sieradzan — Supported by National Science Center of Poland (Narodowe Centrum Nauki) (NCN), grants UMO-2017/25/B/ST4/01026, UMO-2017/26/M/ST4/00044, and UMO-2017/27/B/ST4/00926. Computational resources were provided by (a) the Interdisciplinary Centre of Mathematical and Computational Modelling (ICM) at the University of Warsaw (b) the Centre of Informatics – Tricity Academic Superkomputer & NetworK (CI TASK) in Gdańsk, (c) the Polish Grid Infrastructure (PL-GRID), and (d) our Beowulf cluster at the Faculty of Chemistry, University of Gdańsk.

Venclovas — Research Council of Lithuania (grant S-MIP-21-25).

Wallner — Wallenberg AI, Autonomous System and Software Program (WASP) and Berzelius from Knut and Alice Wallenberg Foundation (KAW), Swedish Research Council, 2020-03352, The Swedish e-Science Research Center, and Carl Tryggers stiftelse för Vetenskaplig Forskning, 20:453.

Yang — The authors acknowledge the financial support from the Science Foundation of the National Key Laboratory of Science and Technology on Advanced Composites in Special Environments, the Fundamental Research Funds for the Central Universities of China.

Zou — This work is supported by NIH/NIGMS R35GM136409 to XZ.

Table S1 – Participation statistics

Targets with one interface (A2) and no intertwining

CAPRI ID	CASP ID	Stoichiometry	#interfaces	Predictors CASP	Predictors All	Scorers CAPRI	#models CASP	#models CAPRI
T198	T1123	A2	1	40	60	15	192	1110
T201	T1132	A2	1	36	58	15	118	992
T211	T1153	A2	1	37	58	15	180	1111
T225	T1178	A2	1	45	66	15	214	1106
T226	T1179	A2	1	45	66	15	217	1070
T229	T1187	A2	1	51	71	15	244	1151

Targets with one interface (A2/A3) and intertwining

CAPRI ID	CASP ID	Stoichiometry	#interfaces	Predictors CASP	Predictors All	Scorers CAPRI	#models CASP	#models CAPRI
T192	T1109	A2	1	40	60	15	188	1009
T193	T1110	A2	1	40	60	15	186	1007
T194	T1113	A2	1	40	62	15	188	995
T197	T1121	A2	1	38	58	15	181	1040
T199	T1127	A2	1	47	69	15	224	1088
T213	T1160	A2	1	43	64	15	211	1023
T214	T1161	A2	1	44	64	15	216	1024
T222	T1173	A3	1	50	71	15	229	1028
T223	T1174	A3	1	49	69	15	217	786
T224	T1176	A2	1	44	63	15	171	969
T227	T1181	A3	1	50	70	15	233	967

Hetero-targets with one interface (A1B1)

CAPRI ID	CASP ID	Stoichiometry	#interfaces	Predictors CASP	Predictors All	Scorers CAPRI	#models CASP	#models CAPRI
T191	H1106	AB	1	57	79	–	258	984
T200	H1129	AB	1	50	71	14	238	1189
T202	H1134	AB	1	55	76	15	266	1059
T210	H1151	AB	1	57	77	15	265	1105
T212	H1157	AB	1	52	73	15	244	1089

Targets with one interface (A1B1 or A:HL): nanobodies and antibodies

CAPRI ID	CASP ID	Stoichiometry	#interfaces	Predictors CASP	Predictors All	Scorers CAPRI	#models CASP	#models CAPRI
T205	H1140	AB	1	58	80	15	262	1379
T206	H1141	AB	1	56	78	15	257	1322
T207	H1142	AB	1	57	77	15	258	1270
T208	H1143	AB	1	57	76	15	261	1072
T209	H1144	AB	1	57	77	15	258	1185
T216	H1166	AHL	1	52	72	15	241	1228
T217	H1167	AHL	1	55	76	15	256	1247
T218	H1168	AHL	1	55	76	15	253	1254

Large assemblies

CAPRI ID	CASP ID	Stoichiometry	#interfaces	Predictors CASP	Predictors All	Scorers CAPRI	#models CASP	#models CAPRI
T195	T1115	A16	1	34	54	15	111	799
T203	H1135	A9B3	4	56	75	15	242	156
T204	H1137	ABCDEFG2IJ	8	46	62	15	169	434
T219	T1170	A6	3	50	75	15	216	882
T220	H1171	A6B1	4	51	63	15	227	850
T221	H1172	A6B2	4	49	63	15	223	850
T230	T1192	A10	1	40	58	15	181	662

Table S2 – Prediction results for Target/Interface T198.1

**Predictors**	Top-1	Top-5
J_Cheng	1**	5**
Wei_Zheng	1**	5/4**
Jianyi_Yang	1	5/3**
Baker	1**	5/3**
Hongjiang_Miao-TRFold	0	4/3**
AIlab-trComplex	0	4/3**
Xinqi_Gong-BeijingAIProtein	1**	3**
Qiwei_Ye-UltraFold	1**	3**
Venclovas	1	4/2**
Wallner	1**	4/1**
Xuyang_Liu-Manifold	0	3/1**
Toshiyuki_Oda-PEZYFoldings	1**	2/1**
Shen	0	1
Kihara	0	1
Gray	0	1
Y_Sun	0	1
Grudinin	1	1
Fang_Bai	0	1

**Servers**	Top-1	Top-5
MULTICOM	1**	5/4**
MULTICOM_QA	1**	5/4**
MULTICOM_DEEP	1**	5/4**
DFOLDING-SERVER	1**	5/4**
DFOLDING-REFINE	1**	5/4**
ULTRAFOLD_SERVER	1**	3**
RAPTORX-MULTIMER	1**	3/1**
YANG-MULTIMER	1	2
LZERD	1	1
MANIFOLD-E	1	1
KIHARA_SERVER	1	1
AF2-MULTIMER	0	1

**Scorers and Scoring Servers**	Top-1	Top-5
LZERD	1**	5/4**
Kihara	1**	5/4**
Venclovas	1**	4/3**
MULTICOM	1**	3**
Oliva	0	1**
S_Chang	1	2

Table S2 – Prediction results for Target/Interface T201.1

**Predictors**	Top-1	Top-5
Sieradzan	1***	5***
Pierce	1***	5***
Kozakov	1***	5***
J_Cheng	1***	5***
Gray	1***	5***
Ovchinnikov-ColabFold	1***	5***
Fang_Bai	1***	5***
DelCarpio	1***	5/4***/1**
Negi	1***	4***
Xuyang_Liu-Manifold	1***	4***
Qiwei_Ye-UltraFold	1***	4***
Hongjiang_Miao-TRFold	1***	4***
Elofsson	1***	4***
Baker	0	4***
S_Chang	1***	5/3***/2**
Kihara	1**	5/3***/2**
Shan_Chang-CoDock	1***	5/3***/2**
Venclovas	1***	4/3***/1**
AIlab-trComplex	0	4/3***/1**
Fernandez-Recio	1***	3***
Xinqi_Gong-BeijingAIProtein	0	3***
Wei_Zheng	0	3***
Grudinin	1***	3***
Shen	1	5/2***/1**
Y_Sun	1	5/2***/1**
Zou	1**	5/1***/4**
Wallner	1***	3/2***/1**
Bates	0	2***
Takeda-Shitaka	1***	2***
Junlin_Wang-MUFold_H	1***	2***
Toshiyuki_Oda-PEZYFoldings	1***	3/1***/2**
S_Huang	1***	1***
McGuffin	0	1***
GuijunLab	0	1***
Gwang_So-FTBiot0119	0	2

Table S2 – Prediction results for Target/Interface T201.1

**Servers**	Top-1	Top-5
MULTICOM	1***	5***
CLUSPRO	1***	5***
YANG-MULTIMER	1***	5***
MANIFOLD-E	1***	5***
RAPTORX-MULTIMER	1***	4***
MULTICOM_DEEP	1***	4***
ULTRAFOLD_SERVER	1***	3***
MULTIFOLD	1***	3***
MUFOLD	0	3***
GUIJUNLAB-DEEPDA	0	3***
GUIJUNLAB-ASSEMBLY	1***	3***
AF2-MULTIMER	1***	3***
LZERD	1**	5/1***/4**
MULTICOM_QA	1***	2***
KIHARA_SERVER	0	4/1***/3**
COLABFOLD	0	2***
HDOCK	1***	1***
MDOCKPP	1**	1**

**Scorers and Scoring Servers**	Top-1	Top-5
Oliva	1***	5***
Bonvin	0	4***
MULTICOM	0	4***
S_Chang	1***	5/3***/2**
Kihara	0	4/3***/1**
Fernandez-Recio	1	4/3***
LZERD	0	3***
S_Huang	1**	5/2***/2**
Takeda-Shitaka	1***	3/2***/1**
Shen	1***	2***
Venclovas	1**	3/1***/2**
Zou	1**	4/3**
MDOCKPP	1**	4/3**
HDOCK	0	1

Table S2 – Prediction results for Target/Interface T211.1

Predictors	Top-1	Top-5
Elofsson	1***	5***
Grudinin	1***	5/4***/1**
J_Cheng	1**	5/3***/2**
Takeda-Shitaka	1***	5/3***/1**
Shen	1***	4/3***
Y_Sun	1***	4/3***
Xinqi_Gong-BeijingAIProtein	0	3***
Qiwei_Ye-UltraFold	0	3***
Junlin_Wang-MUFold_H	1***	5/2***/3**
Wei_Zheng	1**	5/1***/4**
Ovchinnikov-ColabFold	1**	5/1***/4**
McGuffin	1**	5**
Jianyi_Yang	1**	5**
Venclovas	1***	2/1***/1**
Baker	1**	4**
Fang_Bai	1**	5/3**
Zou	1***	2/1***
Kihara	1**	4/3**
Wallner	1***	2/1***
Pierce	0	1***
S_Chang	1**	2**
Toshiyuki_Oda-PEZYFoldings	0	2**
Shan_Chang-CoDock	1**	2**
Fernandez-Recio	1**	3/1**
S_Huang	1**	1**
DFolding	0	1**
David_Jones-DMP	1**	1**
Sieradzan	0	2

Servers	Top-1	Top-5
RAPTORX-MULTIMER	1***	5***
MUFOLD	1***	5***
AF2-MULTIMER	1***	5***
ULTRAFOLD_SERVER	0	3***
GUIJUNLAB-META	1**	5/2***/3**
GUIJUNLAB-DEEPDA	1**	5/2***/3**
GUIJUNLAB-ASSEMBLY	1**	5/2***/3**
MULTICOM	1**	5/1***/4**
MULTICOM_QA	1**	5/1***/4**
COLABFOLD	1**	5/1***/4**
YANG-MULTIMER	1**	5**
MULTIFOLD	1**	5**
MULTICOM_DEEP	1**	5**
LZERD	1**	4**
KIHARA_SERVER	1**	4**
MANIFOLD-E	0	3/1**
HDOCK	1**	1**
DFOLDING-SERVER	1**	1**
DFOLDING-REFINE	1**	1**

Scorers and Scoring Servers	Top-1	Top-5
Oliva	1**	2/1***/1**
Zou	0	2/1***
Venclovas	1**	3**
Kihara	1**	3/2**
HDOCK	0	1**
S_Huang	0	1**
S_Chang	0	1**
Fernandez-Recio	0	1
Shen	0	1

Table S2 – Prediction results for Target/Interface T225.1

**Predictors**	Top-1	Top-5
Kihara	1***	5/2***/3**
Hongjiang_Miao-TRFold	1***	5/2***/2**
Toshiyuki_Oda-PEZYFoldings	1***	5/1***/4**
Jianyi_Yang	1***	5/1***/4**
Zou	1**	5**
Shen	1**	5**
Venclovas	1**	5**
S_Chang	1**	5**
Pierce	1**	5**
Kozakov	1**	5**
J_Cheng	1**	5**
Xuyang_Liu-Manifold	1**	5**
Xinqi_Gong-BeijingAIProtein	1**	5**
Wei_Zheng	1**	5**
Takeda-Shitaka	1**	5**
Y_Sun	1**	5**
Shan_Chang-CoDock	1**	5**
Qiwei_Ye-UltraFold	1**	5**
KozakovVajda	1**	5**
Junlin_Wang-MUFold_H	1**	5**
Fang_Bai	1**	5**
Elofsson	1**	5**
Baker	1**	5**
AIlab-trComplex	1***	3/1***/1**
Wallner	1***	2/1***/1**
McGuffin	1**	4**
Grudinin	1**	4**
GuijunLab	1**	3**
Gray	1**	5/1**
DelCarpio	1	5/1**
Suwen_Zhao	0	2**
Bates	1	5
S_Huang	1**	2/1**
Sachin_Kadyan-OpenFold-SingleSeq	0	2/1**
Sachin_Kadyan-OpenFold	0	2/1**
Jie_Hou-FoldEver-Hybrid	1**	1**
Sieradzan	0	1

Table S2 – Prediction results for Target/Interface T225.1

**Servers**	Top-1	Top-5
DFOLDING-SERVER	1***	5/3***/2**
YANG-SERVER	1***	5/1***/4**
YANG-MULTIMER	1***	5/1***/4**
MULTICOM	1**	5**
LZERD	1**	5**
CLUSPRO	1**	5**
ULTRAFOLD_SERVER	1**	5**
MULTICOM_DEEP	1**	5**
MUFOLD	1**	5**
MANIFOLD-E	1**	5**
KIHARA_SERVER	1**	5**
GUIJUNLAB-DEEPDA	1**	5**
FOLDEVER	1**	5**
AF2-MULTIMER	1**	5**
MULTICOM_QA	1**	4**
MDOCKPP	1**	4/3**
GUIJUNLAB-ASSEMBLY	1**	3**
DFOLDING-REFINE	0	3**
HDOCK	1**	2/1**

**Scorers and Scoring Servers**	Top-1	Top-5
Kihara	1***	5/2***/3**
HDOCK	1**	5/1***/4**
S_Huang	1**	5/1***/4**
Venclovas	1**	4/1***/3**
MULTICOM	1**	5**
Zou	1**	5**
MDOCKPP	1**	5**
LZERD	1**	5**
S_Chang	1**	5**
Fernandez-Recio	1**	5**
Oliva	1	4/3**
Bonvin	0	2/1**
Shen	1**	1**

Table S2 – Prediction results for Target/Interface T226.1

**Predictors**	Top-1	Top-5
Wei_Zheng	1***	2/1***/1**
Toshiyuki_Oda-PEZYFoldings	1***	3/1***
Jianyi_Yang	1**	5/1**
Baker	1**	5/1**
Kihara	1	4/1**
J_Cheng	1	5
Xinqi_Gong-BeijingAIProtein	1	5
Qiwei_Ye-UltraFold	1	5
Junlin_Wang-MUFold_H	1	4
Zou	0	3
Wallner	1	3
DFolding	1**	1**
Shen	0	2
Venclovas	0	2
Sieradzan	0	2
Pierce	1	2
Kozakov	1	2
Takeda-Shitaka	0	2
Y_Sun	0	2
KozakovVajda	1	2
Hongjiang_Miao-TRFold	0	2
Fang_Bai	0	2
AIlab-trComplex	0	2
S_Huang	1	1
Bates	1	1
Xuyang_Liu-Manifold	0	1
GuijunLab	0	1
Grudinin	0	1
Elofsson	1	1

**Servers**	Top-1	Top-5
DFOLDING-SERVER	1**	3/2**
ULTRAFOLD_SERVER	1	5
MULTICOM_DEEP	1	5
MULTICOM	1	4
MULTICOM_QA	1	4
MUFOLD	1	4
DFOLDING-REFINE	1**	2/1**
LZERD	0	2
CLUSPRO	1	2
YANG-SERVER	1	2
YANG-MULTIMER	1	2
KIHARA_SERVER	0	2
GUIJUNLAB-DEEPDA	0	2
AF2-MULTIMER	0	2
MDOCKPP	1	1
HDOCK	1	1
MANIFOLD-E	0	1
GUIJUNLAB-ASSEMBLY	0	1

**Scorers and Scoring Servers**	Top-1	Top-5
Kihara	1	5
Fernandez-Recio	1	4
LZERD	1	3
Venclovas	1	2
Zou	0	2
MULTICOM	1	2
Oliva	1	2
HDOCK	0	2
S_Huang	0	2
Bonvin	0	1
MDOCKPP	0	1
Shen	0	1

Table S2 – Prediction results for Target/Interface T229.1

**Predictors**	Top-1	Top-5
Xinqi_Gong-BeijingAIProtein	1**	3/1***/2**
Qiwei_Ye-UltraFold	1**	3/1***/2**
Venclovas	0	1***
S_Chang	0	1***
J_Cheng	0	1***
Bates	0	1***
Wallner	1***	1***
Shan_Chang-CoDock	0	1***
McGuffin	1***	1***
DelCarpio	1**	2/1**
Baker	0	1

**Servers**	Top-1	Top-5
ULTRAFOLD_SERVER	1**	3/1***/2**
MULTICOM	0	1***
MULTIFOLD	1***	1***
MULTICOM_QA	0	1***
MULTICOM_DEEP	0	1***

**Scorers and Scoring Servers**	Top-1	Top-5
Bonvin	0	1

Table S2 – Prediction results for Target/Interface T192.1

Predictors	Top-1	Top-5
Sieradzan	1***	5***
S_Huang	1***	5***
Negi	1***	5***
Kozakov	1***	5***
Kihara	1***	5***
DelCarpio	1***	5***
Wei_Zheng	1***	5***
Wallner	1***	5***
Takeda-Shitaka	1***	5***
McGuffin	1***	5***
KozakovVajda	1***	5***
Junlin_Wang-MUFold_H	1***	5***
Jianyi_Yang	1***	5***
Elofsson	1***	5***
S_Chang	1***	5/4***/1**
Pierce	1***	5/4***/1**
Bates	1***	5/4***/1**
Xinqi_Gong-BeijingAIProtein	1**	5/4***/1**
Shan_Chang-CoDock	1***	5/4***/1**
Qiwei_Ye-UltraFold	1**	5/4***/1**
GuijunLab	1***	5/3***/2**
Zou	1***	5/3***/1**
J_Cheng	1**	5/2***/3**
Ovchinnikov-ColabFold	1***	3***
Xuyang_Liu-Manifold	1**	4/2***/2**
Venclovas	1***	4/2***/1**
Toshiyuki_Oda-PEZYFoldings	1***	3/2***/1**
Shen	1**	5**
Fernandez-Recio	1**	5**
Y_Sun	1**	5**
Baker	1**	5**
David_Jones-DMP	1***	1***
Gray	1	5/1**
Fang_Bai	1**	2**

Table S2 – Prediction results for Target/Interface T192.1

**Servers**	Top-1	Top-5
HDOCK	1***	5***
CLUSPRO	1***	5***
YANG-MULTIMER	1***	5***
ULTRAFOLD_SERVER	1***	5***
MULTIFOLD	1***	5***
MUFOLD	1***	5***
KIHARA_SERVER	1***	5***
DFOLDING-SERVER	1***	5***
AF2-MULTIMER	1***	5***
MANIFOLD-E	1***	4***
GUIJUNLAB-DEEPDA	1**	5/3***/2**
MULTICOM_DEEP	1**	5/2***/3**
GUIJUNLAB-ASSEMBLY	1***	3***
COLABFOLD	1***	3***
MULTICOM	1**	5**
MULTICOM_QA	1**	5**
MDOCKPP	1**	5/4**
DFOLDING-REFINE	1**	5/4**
RAPTORX-MULTIMER	0	1***

**Scorers and Scoring Servers**	Top-1	Top-5
MULTICOM	1***	5***
Fernandez-Recio	1***	5***
Zou	1***	5***
HDOCK	1***	5***
S_Huang	1***	5***
S_Chang	1***	5/4***/1**
MDOCKPP	1***	5/4***/1**
Bonvin	1***	5/4***/1**
LZERD	1***	5/3***/2**
Venclovas	1***	3***
Takeda-Shitaka	1***	5/1***/3**
J_Cheng	0	2***
Kihara	1***	4/1***/2**
Oliva	1**	4**
Shen	1**	1**

Table S2 – Prediction results for Target/Interface T193.1

Predictors	Top-1	Top-5
Sieradzan	1***	5***
S_Chang	1***	5***
Pierce	1***	5***
Negi	1***	5***
Kozakov	1***	5***
Kihara	1***	5***
J_Cheng	1***	5***
DelCarpio	1***	5***
Bates	1***	5***
Xinqi_Gong-BeijingAIProtein	1***	5***
Wei_Zheng	1***	5***
Takeda-Shitaka	1***	5***
Shan_Chang-CoDock	1***	5***
Qiwei_Ye-UltraFold	1***	5***
Ovchinnikov-ColabFold	1***	5***
McGuffin	1***	5***
KozakovVajda	1***	5***
Junlin_Wang-MUFold_H	1***	5***
Hongjiang_Miao-TRFold	1***	5***
GuijunLab	1***	5***
Elofsson	1***	5***
Baker	1***	5***
AIlab-trComplex	1***	5***
Zou	1***	5/3***/2**
Fernandez-Recio	1***	5/3***/2**
Venclovas	1***	5/3***/1**
Jianyi_Yang	1***	5/3***
Shen	1**	5/2***/3**
Wallner	1***	3***
Y_Sun	1**	5/2***/3**
Toshiyuki_Oda-PEZYFoldings	1***	5/1***/2**
Xuyang_Liu-Manifold	1***	5/1***/1**
Gray	1**	5/4**
S_Huang	1***	1***
David_Jones-DMP	1***	1***
Fang_Bai	1	5/1**

Table S2 – Prediction results for Target/Interface T193.1

**Servers**	Top-1	Top-5
MULTICOM	1***	5***
CLUSPRO	1***	5***
YANG-MULTIMER	1***	5***
ULTRAFOLD_SERVER	1***	5***
MULTIFOLD	1***	5***
MULTICOM_QA	1***	5***
MULTICOM_DEEP	1***	5***
MUFOLD	1***	5***
KIHARA_SERVER	1***	5***
GUIJUNLAB-DEEPDA	1***	5***
DFOLDING-SERVER	1***	5***
AF2-MULTIMER	1***	5***
COLABFOLD	1***	4***
MDOCKPP	1***	5/2***/3**
RAPTORX-MULTIMER	1***	3***
GUIJUNLAB-ASSEMBLY	1***	3***
MANIFOLD-E	1***	5/1***/1**
DFOLDING-REFINE	1**	5**
HDOCK	1***	1***

**Scorers and Scoring Servers**	Top-1	Top-5
MULTICOM	1***	5***
Zou	1***	5***
Oliva	1***	5***
S_Chang	1***	5***
Fernandez-Recio	1***	5***
MDOCKPP	1**	5/4***/1**
LZERD	1	5/4***
Bonvin	1***	5/3***/1**
Venclovas	1***	4/3***
Takeda-Shitaka	1**	5/2***/3**
J_Cheng	0	2***
Kihara	1	4/1***/2**
Shen	1**	3/1***/2**
HDOCK	1***	1***
S_Huang	1***	1***

Table S2 – Prediction results for Target/Interface T194.1

Predictors	Top-1	Top-5
Jianyi_Yang	1**	5/1***/4**
Negi	1**	5**
Kozakov	1**	5**
Kihara	1**	5**
J_Cheng	1**	5**
Gray	1**	5**
Xinqi_Gong-BeijingAIProtein	1**	5**
Toshiyuki_Oda-PEZYFoldings	1**	5**
Takeda-Shitaka	1**	5**
Qiwei_Ye-UltraFold	1**	5**
Ovchinnikov-ColabFold	1**	5**
Junlin_Wang-MUFold_H	1**	5**
Grudinin	1**	5**
Fang_Bai	1**	5**
Elofsson	1**	5**
S_Chang	1	5/4**
Bates	1	5/4**
Wei_Zheng	1**	5/4**
Shan_Chang-CoDock	1	5/4**
GuijunLab	1	5/4**
Pierce	1**	4**
Sieradzan	1**	5/3**
Fernandez-Recio	1**	5/3**
Hongjiang_Miao-TRFold	1	5/3**
AIlab-trComplex	1	5/3**
Shen	1**	4/3**
Y_Sun	1**	4/3**
McGuffin	1	5/2**
Zou	1**	3/2**
Wallner	1**	3/2**
Venclovas	1**	2**
Baker	1	5
Xuyang_Liu-Manifold	0	2/1**
S_Huang	1**	1**
DelCarpio	0	1**
David_Jones-DMP	1**	1**

Table S2 – Prediction results for Target/Interface T194.1

**Servers**	Top-1	Top-5
MULTICOM	1**	5**
LZERD	1**	5**
CLUSPRO	1**	5**
YANG-MULTIMER	1**	5**
ULTRAFOLD_SERVER	1**	5**
RAPTORX-MULTIMER	1**	5**
MULTIFOLD	1**	5**
MULTICOM_QA	1**	5**
MULTICOM_DEEP	1**	5**
MUFOLD	1**	5**
KIHARA_SERVER	1**	5**
GUIJUNLAB-DEEPDA	1**	5**
COLABFOLD	1**	5**
AF2-MULTIMER	1**	5**
DFOLDING-SERVER	1**	5/4**
DFOLDING-REFINE	1**	5/4**
GUIJUNLAB-ASSEMBLY	1**	3**
HDOCK	1**	1**
MANIFOLD-E	1**	1**
MDOCKPP	1	1

**Scorers and Scoring Servers**	Top-1	Top-5
MULTICOM	1**	5**
Venclovas	1**	5**
Oliva	1**	5**
HDOCK	1**	5**
S_Huang	1**	5**
Fernandez-Recio	1**	5/4**
S_Chang	1**	5/4**
LZERD	1**	5/4**
Kihara	1**	5/4**
MDOCKPP	1**	5/3**
Takeda-Shitaka	1	4/2**
Bonvin	1**	2**
Zou	1**	2/1**

Table S2 – Prediction results for Target/Interface T197.1

**Predictors**	Top-1	Top-5
DelCarpio	1	4
S_Chang	1	2
Shan_Chang-CoDock	1	2
Wallner	0	1
Ovchinnikov-ColabFold	0	1

**Servers**	Top-1	Top-5
DFOLDING-SERVER	0	1
COLABFOLD	0	1

**Scorers and Scoring Servers**	Top-1	Top-5
S_Chang	0	2

Table S2 – Prediction results for Target/Interface T199.1

**Predictors**	Top-1	Top-5
Sieradzan	1***	5***
S_Chang	1***	5***
Pierce	1***	5***
Negi	1***	5***
Kozakov	1***	5***
J_Cheng	1***	5***
Gray	1***	5***
Zechen_Wang-AIchemy_LIG2	1***	5***
Xinqi_Gong-BeijingAIProtein	1***	5***
Xiao_Jia-AIchemy_LIG3	1***	5***
Wei_Zheng	1***	5***
Takeda-Shitaka	1***	5***
Shan_Chang-CoDock	1***	5***
Qiwei_Ye-UltraFold	1***	5***
Ovchinnikov-ColabFold	1***	5***
Ness	1***	5***
McGuffin	1***	5***
Liangzhen_Zheng-AIchemy_LIG	1***	5***
KozakovVajda	1***	5***
Junlin_Wang-MUFold_H	1***	5***
Jianyi_Yang	1***	5***
Hongjiang_Miao-TRFold	1***	5***
GuijunLab	1***	5***
Grudinin	1***	5***
Elofsson	1***	5***
AIlab-trComplex	1***	5***
Baker	1**	5/4***/1**
Fernandez-Recio	1***	5/3***/2**
Zou	1***	5/2***/3**
Xuyang_Liu-Manifold	1***	3***
Junhan_Chang-Manifold-X	1***	3***
Venclovas	1***	4/2***
Toshiyuki_Oda-PEZYFoldings	1***	4/2***
Fang_Bai	1**	5/1***/3**
DelCarpio	1**	5**
Bates	1**	5**
Kihara	1**	5/3**
Shen	1	5/2**
S_Huang	1***	1***
Wallner	1***	1***
Y_Sun	1	5/2**
Kazuki_Yamamoto-ddquest	1***	1***

Table S2 – Prediction results for Target/Interface T199.1

Servers	Top-1	Top-5
MULTICOM	1***	5***
CLUSPRO	1***	5***
YANG-SERVER	1***	5***
YANG-MULTIMER	1***	5***
ULTRAFOLD_SERVER	1***	5***
RAPTORX-MULTIMER	1***	5***
MULTIFOLD	1***	5***
MULTICOM_QA	1***	5***
MULTICOM_DEEP	1***	5***
MUFOLD	1***	5***
GUIJUNLAB-DEEPDA	1***	5***
GUIJUNLAB-ASSEMBLY	1***	5***
FOLDEVER	1***	5***
COLABFOLD	1***	5***
AF2-MULTIMER	1***	5***
MANIFOLD-LC-E	1***	4***
MANIFOLD-E	1***	4***
MDOCKPP	1**	5/1***/4**
LZERD	1**	5**
KIHARA_SERVER	1**	5**
DFOLDING-REFINE	1**	5**
HDOCK	1***	1***

Scorers and Scoring Servers	Top-1	Top-5
Takeda-Shitaka	1***	5/4***/1**
LZERD	1***	5/4***
S_Chang	1***	5/3***/2**
Fernandez-Recio	1**	5/3***/2**
Zou	1**	5/1***/4**
MDOCKPP	1**	5/1***/4**
Kihara	1	5/2***
Venclovas	1***	5/1***/4**
Oliva	1**	5**
HDOCK	1***	3/1***/2**
S_Huang	1***	3/1***/2**
Bonvin	1***	2/1***
Shen	0	1***
MULTICOM	1	4/2**

Table S2 – Prediction results for Target/Interface T213.1

Predictors	Top-1	Top-5
S_Huang	0	1**
Toshiyuki_Oda-PEZYFoldings	0	1**

Servers	Top-1	Top-5
HDOCK	0	1**

Scorers and Scoring Servers	Top-1	Top-5

no acceptable models

Table S2 – Prediction results for Target/Interface T214.1

**Predictors**	Top-1	Top-5
Kozakov	0	2/1***
KozakovVajda	0	2/1***
Wallner	0	1***
Pierce	1**	2/1**
S_Huang	0	1**
Xuyang_Liu-Manifold	0	1
Toshiyuki_Oda-PEZYFoldings	0	1

**Servers**	Top-1	Top-5
CLUSPRO	0	2/1***
HDOCK	0	1**
DFOLDING-REFINE	1	1

**Scorers and Scoring Servers**	Top-1	Top-5
Venclovas	0	1***
Takeda-Shitaka	1**	1**
HDOCK	0	1**
S_Huang	0	1**
Fernandez-Recio	1	1

Table S2 – Prediction results for Target/Interface T222.1

**Predictors**	Top-1	Top-5
Jianyi_Yang	1***	5***
Wei_Zheng	1***	4/3***/1**
J_Cheng	1***	4/2***/1**
GuijunLab	1	5/2***
S_Huang	1***	4/1***/3**
Zhigang_Sun-GinobiFold	1***	3/1***/2**
Xuyang_Liu-Manifold	1***	3/1***/1**
Kihara	1**	4**
Wallner	1***	2/1***/1**
Anqi_Pang-Coqualia	1***	2/1***/1**
Takeda-Shitaka	1	5/3**
Toshiyuki_Oda-PEZYFoldings	1**	4/3**
Elofsson	1**	4/3**
Zou	1**	3**
Venclovas	1**	5/2**
S_Chang	1	5/2**
Shan_Chang-CoDock	1	5/2**
Baker	1**	3**
Xinqi_Gong-BeijingAIProtein	1**	3/2**
Qiwei_Ye-UltraFold	1**	3/2**
AIlab-trComplex	1**	3/2**
Kozakov	0	2**
KozakovVajda	0	2**
Junlin_Wang-MUFold_H	1**	2**
Pierce	1**	3/1**
Hongjiang_Miao-TRFold	1**	3/1**
Ovchinnikov-ColabFold	1**	2/1**
Jie_Hou-FoldEver-Hybrid	1**	1**
Shen	1	2
Y_Sun	1	2
McGuffin	0	2
Fernandez-Recio	0	1
Grudinin	0	1
Fang_Bai	0	1
DFolding	0	1

Table S2 – Prediction results for Target/Interface T222.1

Servers	Top-1	Top-5
YANG-SERVER	1***	5/4***/1**
YANG-MULTIMER	1***	5/4***/1**
MULTICOM	1***	2***
HDOCK	1***	4/1***/3**
MULTICOM_QA	1***	2***
MULTICOM_DEEP	0	2***
MDOCKPP	1**	4/1***/2**
SHANGHAITECH-TS-SER	1***	3/1***/2**
GUIJUNLAB-ASSEMBLY	0	3/1***/2**
MANIFOLD-E	1***	3/1***/1**
GINOBIFOLD	1***	2/1***/1**
GUIJUNLAB-DEEPDA	1***	3/1***
MUFOLD	0	3**
ULTRAFOLD_SERVER	1**	3/2**
CLUSPRO	0	2**
COLABFOLD	1**	2/1**
AF2-MULTIMER	1**	2/1**
FOLDEVER	1**	1**
MULTIFOLD	0	2
DFOLDING-SERVER	0	1
DFOLDING-REFINE	0	1

Scorers and Scoring Servers	Top-1	Top-5
Takeda-Shitaka	1**	5/4***/1**
MULTICOM	1***	5/2***/3**
LZERD	1	5/2***/2**
Kihara	1	5/1***/3**
HDOCK	0	4/1***/2**
S_Huang	0	4/1***/2**
Venclovas	1**	5/4**
S_Chang	1	5/3**
Zou	1**	4/3**
MDOCKPP	0	3/2**
Fernandez-Recio	1	4/1**
Bonvin	1	3/1**
Oliva	0	1

Table S2 – Prediction results for Target/Interface T223.1

**Predictors**	Top-1	Top-5
Kihara	1	5
Ovchinnikov-ColabFold	1	5
DFolding	1**	3/1**
Baker	1	4
Zou	0	3
Kozakov	1	3
Takeda-Shitaka	1	3
KozakovVajda	1	3
Jianyi_Yang	1	3
Elofsson	1	3
Venclovas	1	2
Pierce	0	2
Zhigang_Sun-GinobiFold	0	2
Wallner	1	2
Toshiyuki_Oda-PEZYFoldings	0	2
Junlin_Wang-MUFold_H	0	2
Hongjiang_Miao-TRFold	0	2
Fang_Bai	1	2
Shen	0	1
S_Huang	1	1
J_Cheng	0	1
Xuyang_Liu-Manifold	0	1
Xinqi_Gong-BeijingAIProtein	1	1
Suwen_Zhao	0	1
Y_Sun	0	1
Qiwei_Ye-UltraFold	1	1
GuijunLab	1	1
Grudinin	0	1
David_Jones-DMP	1	1
AIlab-trComplex	0	1

**Servers**	Top-1	Top-5
DFOLDING-SERVER	1**	3/1**
COLABFOLD	1	5
CLUSPRO	1	3
YANG-SERVER	1	3
YANG-MULTIMER	1	3
MULTIFOLD	1	3
MULTICOM	0	2
MDOCKPP	1	2
SHANGHAITECH-TS-SER	0	2
MULTICOM_QA	0	2
MUFOLD	0	2
DFOLDING-REFINE	1	2
HDOCK	1	1
ULTRAFOLD_SERVER	1	1
MULTICOM_DEEP	0	1
MANIFOLD-E	1	1
GUIJUNLAB-ASSEMBLY	1	1
AF2-MULTIMER	1	1

**Scorers and Scoring Servers**	Top-1	Top-5
Kihara	1	5
Takeda-Shitaka	1	3
LZERD	1	2
HDOCK	0	2
S_Huang	0	2
MULTICOM	1	1
Zou	0	1
MDOCKPP	0	1
Venclovas	0	1
Bonvin	0	1
Oliva	0	1

Table S2 – Prediction results for Target/Interface T224.1

**Predictors**	Top-1	Top-5
no acceptable models		

**Servers**	Top-1	Top-5
no acceptable models		

**Scorers and Scoring Servers**	Top-1	Top-5
no acceptable models		

Table S2 – Prediction results for Target/Interface T227.1

**Predictors**	Top-1	Top-5
Wei_Zheng	1***	5/1***/4**
Toshiyuki_Oda-PEZYFoldings	1***	5/1***/4**
Kihara	1**	3/1***/2**
J_Cheng	1**	5**
Ovchinnikov-ColabFold	1**	5**
Ness	1**	5**
Jianyi_Yang	1**	5/4**
Baker	1**	5/4**
Venclovas	1**	4**
Pierce	1**	5/3**
Kozakov	1**	4/3**
Takeda-Shitaka	1**	4/3**
KozakovVajda	1**	4/3**
Shen	1**	3**
Y_Sun	1**	3**
McGuffin	1**	5/1**
Junlin_Wang-MUFold_H	1**	3/2**
Elofsson	1**	3/2**
Wallner	1**	4/1**
Xuyang_Liu-Manifold	1	5
Junhan_Chang-Manifold-X	1	5
Xinqi_Gong-BeijingAIProtein	1	4
Qiwei_Ye-UltraFold	1	4
Fang_Bai	0	2/1**
Zou	1	3
S_Chang	0	1**
Shan_Chang-CoDock	0	1**
Grudinin	0	3
S_Huang	1	1
Jie_Hou-FoldEver-Hybrid	1	1
Hongjiang_Miao-TRFold	0	1
DFolding	1	1
AIlab-trComplex	0	1

Table S2 – Prediction results for Target/Interface T227.1

**Servers**	Top-1	Top-5
MULTICOM	1**	5**
MULTICOM_QA	1**	5**
MULTICOM_DEEP	1**	5**
COLABFOLD	1**	5**
YANG-SERVER	1**	5/4**
YANG-MULTIMER	1**	5/4**
AF2-MULTIMER	1**	5/3**
CLUSPRO	1**	4/3**
MULTIFOLD	1**	3**
LZERD	1**	5/1**
KIHARA_SERVER	1**	5/1**
DFOLDING-REFINE	1**	5/1**
MANIFOLD-LC-E	1	5
MANIFOLD-E	1	5
FOLDEVER	1	5
ULTRAFOLD_SERVER	1	4
GUIJUNLAB-DEEPDA	0	2
HDOCK	1	1
MUFOLD	0	1
DFOLDING-SERVER	1	1

**Scorers and Scoring Servers**	Top-1	Top-5
Takeda-Shitaka	1**	5**
Venclovas	1**	5**
Zou	1**	4**
MDOCKPP	1**	4**
Bonvin	1**	4/3**
Kihara	1**	3**
S_Chang	1**	3**
Fernandez-Recio	0	3**
HDOCK	1**	3/2**
S_Huang	1**	3/2**
LZERD	0	1**
Shen	0	2
Oliva	0	1

Table S2 – Prediction results for Target/Interface T191.1

**Predictors**	Top-1	Top-5
Kihara	1***	5***
J_Cheng	1***	5***
Wei_Zheng	1***	5***
Y_Sun	1***	5***
Sachin_Kadyan-OpenFold-SingleSeq	1***	5***
Sachin_Kadyan-OpenFold	1***	5***
Grudinin	1***	5***
Elofsson	1***	5***
S_Chang	1***	5/4***/1**
Xuyang_Liu-Manifold	1***	5/4***/1**
Wallner	1***	5/4***/1**
Shan_Chang-CoDock	1***	5/4***/1**
Baker	1***	5/4***/1**
Shen	1***	5/3***/2**
Xinqi_Gong-BeijingAIProtein	1***	5/3***/2**
Takeda-Shitaka	1***	5/3***/2**
Qiwei_Ye-UltraFold	1***	5/3***/2**
Hongjiang_Miao-TRFold	1**	5/3***/2**
GuijunLab	1***	5/3***/2**
AIlab-trComplex	1**	5/3***/2**
Venclovas	1**	5/2***/3**
Toshiyuki_Oda-PEZYFoldings	1***	5/2***/3**
Jianyi_Yang	1***	5/2***/3**
Sieradzan	1**	5/1***/4**
Negi	1**	5/1***/4**
Ovchinnikov-ColabFold	1**	5/1***/4**
Junlin_Wang-MUFold_H	1***	5/1***/4**
Suwen_Zhao	1**	5/1***/3**
Zou	1**	5**
Kozakov	1**	5**
Gray	1**	5**
Bates	1**	5**
McGuffin	1**	5**
KozakovVajda	1**	5**
Anqi_Pang-Coqualia	1**	5**
Jie_Hou-FoldEver-Hybrid	1**	5/4**
Fang_Bai	1**	5/4**
S_Huang	1***	2/1***/1**
Pierce	1***	2/1***/1**
Gwang_So-FTBiot0119	1***	1***
YiTing_Chen	1**	1**
Wenyi_Zhang	1**	1**
Ruihan_Guo	1**	1**
David_Jones-DMP	1**	1**
Fernandez-Recio	0	1

Table S2 – Prediction results for Target/Interface T191.1

**Servers**	Top-1	Top-5
MULTICOM	1***	5***
LZERD	1***	5***
MULTICOM_QA	1***	5***
MULTICOM_DEEP	1***	5***
MUFOLD	1***	5***
KIHARA_SERVER	1***	5***
AF2-MULTIMER	1***	5***
DFOLDING-SERVER	1***	5/4***/1**
YANG-SERVER	1**	5/2***/3**
YANG-MULTIMER	1**	5/2***/3**
MANIFOLD-E	1**	5/2***/3**
GUIJUNLAB-DEEPDA	1**	5/1***/4**
GUIJUNLAB-ASSEMBLY	1***	3/2***/1**
COLABFOLD	1**	5/1***/4**
CLUSPRO	1**	5**
ULTRAFOLD_SERVER	1**	3/1***/2**
RAPTORX-MULTIMER	1**	5**
MULTIFOLD	1**	5**
FOLDEVER	1**	5/4**
DFOLDING-REFINE	1**	5/4**
HDOCK	1***	2/1***/1**
MDOCKPP	1**	2**
XRC_VU	1**	1**

**Scorers and Scoring Servers**	Top-1	Top-5
no scoring for this target		

Table S2 – Prediction results for Target/Interface T200.1

Predictors	Top-1	Top-5
Jianyi_Yang	1***	5***
Venclovas	1***	1***
Wallner	1***	1***
S_Chang	1	4
Shan_Chang-CoDock	1	4
Bates	1	3
Fang_Bai	1	2
Zou	1	1
Kihara	1	1
Fernandez-Recio	0	1
Wei_Zheng	0	1
Ovchinnikov-ColabFold	0	1
Anqi_Pang-Coqualia	0	1

Servers	Top-1	Top-5
YANG-MULTIMER	0	4/1***/3**
TS317	1***	1***
LZERD	0	1
SHANGHAITECH-TS-SER	0	1
KIHARA_SERVER	0	1
GINOBIFOLD	0	1
COLABFOLD	0	1

Scorers and Scoring Servers	Top-1	Top-5
Kihara	1**	4/1***/1**
Venclovas	1***	2/1***
Zou	0	1***
MDOCKPP	0	1***
HDOCK	0	1***
S_Huang	0	1***
Bonvin	0	1***
MULTICOM	1	4
Oliva	0	2
S_Chang	1	2

Table S2 – Prediction results for Target/Interface T202.1

**Predictors**	Top-1	Top-5
Ovchinnikov-ColabFold	1***	5/3***/2**
McGuffin	1**	5/2***/3**
Pierce	1***	5/1***/4**
Xuyang_Liu-Manifold	1***	5/1***/4**
Takeda-Shitaka	1**	5/1***/4**
Suwen_Zhao	1**	5/1***/4**
Junhan_Chang-Manifold-X	1***	5/1***/4**
Grudinin	1**	5/1***/4**
Elofsson	1**	5/1***/4**
Jianyi_Yang	1***	5/1***/3**
Zou	1**	5**
Kozakov	1**	5**
Kihara	1**	5**
J_Cheng	1**	5**
Gray	1**	5**
Bates	1**	5**
Zhigang_Sun-GinobiFold	1**	5**
Xinqi_Gong-BeijingAIProtein	1**	5**
Wenyi_Zhang	1**	5**
Qiwei_Ye-UltraFold	1**	5**
KozakovVajda	1**	5**
Junlin_Wang-MUFold_H	1**	5**
Jie_Hou-FoldEver-Hybrid	1***	3/1***/2**
Fang_Bai	1**	5**
Anqi_Pang-Coqualia	1**	5**
Shen	1**	5/4**
Y_Sun	1**	5/4**
S_Chang	1**	4**
Shan_Chang-CoDock	1**	4**
Wallner	1***	2/1***
Venclovas	1***	1***
Wei_Zheng	1**	3**
Ruihan_Guo	1***	1***
Hongjiang_Miao-TRFold	0	3**
AIlab-trComplex	0	3**
GuijunLab	1	5/1**
Baker	1	5
S_Huang	1**	1**
Toshiyuki_Oda-PEZYFoldings	1**	1**
Fernandez-Recio	1	2

Table S2 – Prediction results for Target/Interface T202.1

**Servers**	Top-1	Top-5
COLABFOLD	1***	5/3***/2**
MULTICOM	1**	5/1***/4**
MULTIFOLD	1**	5/1***/4**
MULTICOM_QA	1**	5/1***/4**
MANIFOLD-E	1***	5/1***/4**
AF2-MULTIMER	1**	5/1***/4**
YANG-SERVER	1**	5/1***/3**
LZERD	1**	5**
CLUSPRO	1**	5**
ULTRAFOLD_SERVER	1**	5**
SHANGHAITECH-TS-SER	1**	5**
MULTICOM_DEEP	1**	5**
MUFOLD	1**	5**
KIHARA_SERVER	1**	5**
GINOBIFOLD	1**	5**
FOLDEVER	1**	3/1***/2**
GUIJUNLAB-DEEPDA	1**	5/4**
RAPTORX-MULTIMER	0	4**
GUIJUNLAB-ASSEMBLY	1**	4**
DFOLDING-REFINE	1**	4**
YANG-MULTIMER	1**	5/3**
MDOCKPP	1**	5/2**
HDOCK	1**	1**

**Scorers and Scoring Servers**	Top-1	Top-5
S_Chang	1**	5/1***/4**
Venclovas	1***	5/1***/4**
Bonvin	1**	5/1***/4**
Zou	1**	5/1***/4**
MDOCKPP	1**	5/1***/4**
Takeda-Shitaka	1**	5**
Oliva	1**	5**
MULTICOM	1**	5**
Fernandez-Recio	1**	4**
Kihara	1**	3**
HDOCK	1**	3**
S_Huang	1**	3**
Shen	0	2**
J_Cheng	0	1**

Table S2 – Prediction results for Target/Interface T210.1

**Predictors**	Top-1	Top-5
Kozakov	1***	5***
J_Cheng	1***	5***
Gray	1***	5***
Zhigang_Sun-GinobiFold	1***	5***
Xuyang_Liu-Manifold	1***	5***
Xinqi_Gong-BeijingAIProtein	1***	5***
Wei_Zheng	1***	5***
Sachin_Kadyan-OpenFold-SingleSeq	1***	5***
Sachin_Kadyan-OpenFold	1***	5***
Qiwei_Ye-UltraFold	1***	5***
Ovchinnikov-ColabFold	1***	5***
McGuffin	1***	5***
KozakovVajda	1***	5***
Junlin_Wang-MUFold_H	1***	5***
Jianyi_Yang	1***	5***
GuijunLab	1***	5***
Grudinin	1***	5***
Elofsson	1***	5***
DFolding	1***	5***
Baker	1***	5***
Anqi_Pang-Coqualia	1***	5***
Zou	1***	5/4***/1**
Shen	1***	5/4***/1**
Takeda-Shitaka	1***	5/4***/1**
Y_Sun	1***	5/4***/1**
Kihara	1***	4***
Suwen_Zhao	1***	4***
S_Chang	1***	5/3***/2**
Wallner	1***	5/3***/2**
Shan_Chang-CoDock	1***	5/3***/2**
Venclovas	1***	4/3***/1**
YiTing_Chen	1***	4/3***/1**
Fernandez-Recio	1***	3/2***/1**
S_Huang	1***	2***
Hongjiang_Miao-TRFold	0	2***
AIlab-trComplex	0	2***
Toshiyuki_Oda-PEZYFoldings	1***	4/1***/2**
DelCarpio	1**	5**
Fang_Bai	1**	5**
Pierce	1	2/1***
Ruihan_Guo	1***	1***
Jie_Hou-FoldEver-Hybrid	1***	1***
David_Jones-DMP	1***	1***
Wenyi_Zhang	1	5/1**
Sieradzan	1**	2/1**

Table S2 – Prediction results for Target/Interface T210.1

**Servers**	Top-1	Top-5
MULTICOM	1***	5***
CLUSPRO	1***	5***
YANG-MULTIMER	1***	5***
ULTRAFOLD_SERVER	1***	5***
SHANGHAITECH-TS-SER	1***	5***
RAPTORX-MULTIMER	1***	5***
MULTIFOLD	1***	5***
MULTICOM_QA	1***	5***
MULTICOM_DEEP	1***	5***
MUFOLD	1***	5***
MANIFOLD-E	1***	5***
GUIJUNLAB-ASSEMBLY	1***	5***
GINOBIFOLD	1***	5***
DFOLDING-SERVER	1***	5***
COLABFOLD	1***	5***
AF2-MULTIMER	1***	5***
GUIJUNLAB-DEEPDA	1**	5/4***/1**
MDOCKPP	1***	4/3***/1**
XRC_VU	1	4/3***
DFOLDING-REFINE	1**	5/2***/3**
HDOCK	1***	2***
FOLDEVER	1***	1***

**Scorers and Scoring Servers**	Top-1	Top-5
Zou	1***	5/3***/2**
Oliva	1**	5/3***/2**
Fernandez-Recio	1***	3***
MDOCKPP	1**	5/1***/4**
S_Chang	1***	5/1***/3**
Kihara	1***	2***
HDOCK	1**	4/1***/2**
S_Huang	1**	4/1***/2**
Takeda-Shitaka	1**	3**
Venclovas	1	4/2**
Bonvin	1**	3/2**
Shen	0	1**

Table S2 – Prediction results for Target/Interface T212.1

**Predictors**	Top-1	Top-5
Shen	1**	5**
S_Chang	1**	5**
Pierce	1**	5**
Kozakov	1**	5**
J_Cheng	1**	5**
Gray	1**	5**
Zhigang_Sun-GinobiFold	1**	5**
Xinqi_Gong-BeijingAIProtein	1**	5**
Takeda-Shitaka	1**	5**
Y_Sun	1**	5**
Shan_Chang-CoDock	1**	5**
Qiwei_Ye-UltraFold	1**	5**
Ovchinnikov-ColabFold	1**	5**
KozakovVajda	1**	5**
Junlin_Wang-MUFold_H	1**	5**
Hongjiang_Miao-TRFold	1**	5**
GuijunLab	1**	5**
Grudinin	1**	5**
Elofsson	1**	5**
Baker	1**	5**
Anqi_Pang-Coqualia	1**	5**
AIlab-trComplex	1**	5**
Jianyi_Yang	1**	5/4**
DFolding	1**	5/4**
Venclovas	1**	5/3**
Kihara	1**	5/3**
Suwen_Zhao	1**	4/3**
Xuyang_Liu-Manifold	1	5/2**
Zou	1**	4/2**
Wei_Zheng	1**	5/1**
Sachin_Kadyan-OpenFold-SingleSeq	0	2**
Sachin_Kadyan-OpenFold	0	2**
McGuffin	1**	2**
Wenyi_Zhang	1	5
S_Huang	1**	1**
YiTing_Chen	0	1**
Toshiyuki_Oda-PEZYFoldings	1**	1**
Ruihan_Guo	1**	1**
Fang_Bai	0	3
David_Jones-DMP	1**	1**

Table S2 – Prediction results for Target/Interface T212.1

Servers	Top-1	Top-5
MULTICOM	1**	5**
CLUSPRO	1**	5**
YANG-SERVER	1**	5**
ULTRAFOLD_SERVER	1**	5**
SHANGHAITECH-TS-SER	1**	5**
RAPTORX-MULTIMER	1**	5**
MULTICOM_QA	1**	5**
MULTICOM_DEEP	1**	5**
MUFOLD	1**	5**
GUIJUNLAB-DEEPDA	1**	5**
GUIJUNLAB-ASSEMBLY	1**	5**
GINOBIFOLD	1**	5**
COLABFOLD	1**	5**
AF2-MULTIMER	1**	5**
DFOLDING-SERVER	1**	5/4**
YANG-MULTIMER	1**	4**
LZERD	1	5/2**
MANIFOLD-E	1	5/2**
KIHARA_SERVER	1	5/2**
MULTIFOLD	1**	2**
DFOLDING-REFINE	1	4/1**
MDOCKPP	1**	1**
HDOCK	1**	1**

Scorers and Scoring Servers	Top-1	Top-5
Oliva	1**	5**
LZERD	1**	5**
Venclovas	1**	5**
Kihara	1**	5/4**
Takeda-Shitaka	1**	5/4**
S_Chang	1**	4**
Zou	1**	5/3**
MDOCKPP	1**	5/3**
HDOCK	1**	4/3**
S_Huang	1**	4/3**
J_Cheng	0	3**
Fernandez-Recio	1**	3**
Bonvin	1**	2/1**
Shen	0	1**

Table S2 – Prediction results for Target/Interface T205.1

Predictors	Top-1	Top-5
Wei_Zheng	1**	3/1**
Wallner	1**	1**
Pierce	1	1
Kihara	1	1
Toshiyuki_Oda-PEZYFoldings	0	1

Servers	Top-1	Top-5
LZERD	1	1
KIHARA_SERVER	1	1

Scorers and Scoring Servers	Top-1	Top-5
Oliva	0	1**

Table S2 – Prediction results for Target/Interface T206.1

Predictors	Top-1	Top-5
Venclovas	1***	2/1***
Wallner	1***	1***
Toshiyuki_Oda-PEZYFoldings	0	1***
David_Jones-DMP	1***	1***
Wei_Zheng	1**	2**
S_Huang	0	1**
Xuyang_Liu-Manifold	1**	1**
Pierce	0	1

Servers	Top-1	Top-5
HDOCK	0	1**
MANIFOLD-E	1**	1**
DFOLDING-SERVER	1**	1**
DFOLDING-REFINE	1**	1**

Scorers and Scoring Servers	Top-1	Top-5
Venclovas	1***	2/1***

Table S2 – Prediction results for Target/Interface T207.1

Predictors	Top-1	Top-5
Kihara	0	1

Servers	Top-1	Top-5
no acceptable models		

Scorers and Scoring Servers	Top-1	Top-5
no acceptable models		

Table S2 – Prediction results for Target/Interface T208.1

Predictors	Top-1	Top-5
Zhigang_Sun-GinobiFold	1***	5***
Takeda-Shitaka	1***	5***
McGuffin	1***	5***
Elofsson	1***	5/4***/1**
Wei_Zheng	1***	4***
Anqi_Pang-Coqualia	1***	4***
J_Cheng	1***	5/3***/2**
GuijunLab	1**	5/3***/2**
Jianyi_Yang	1***	4/3***
Gray	1***	5/2***/3**
Fernandez-Recio	1***	3/2***/1**
Ovchinnikov-ColabFold	1***	5/2***
Junlin_Wang-MUFold_H	1**	5/1***/4**
Hongjiang_Miao-TRFold	1***	4/2***
AIlab-trComplex	1***	4/2***
Venclovas	1***	3/2***
Suwen_Zhao	1***	2***
Xuyang_Liu-Manifold	1***	3/1***/1**
S_Chang	0	2/1***/1**
Shan_Chang-CoDock	0	2/1***/1**
Wenyi_Zhang	1**	5/3**
Jie_Hou-FoldEver-Hybrid	1	3/1***
Grudinin	1***	3/1***
Kozakov	1***	2/1***
Xinqi_Gong-BeijingAIProtein	1	2/1***
Toshiyuki_Oda-PEZYFoldings	1***	2/1***
Qiwei_Ye-UltraFold	1	2/1***
Zou	0	1***
Pierce	1***	1***
Wallner	1***	1***
David_Jones-DMP	1***	1***
Sieradzan	1	4
S_Huang	1**	2/1**
Sachin_Kadyan-OpenFold-SingleSeq	0	1
Sachin_Kadyan-OpenFold	0	1

Servers	Top-1	Top-5
MULTICOM	1***	5***
MULTICOM_QA	1***	5***
MULTICOM_DEEP	1***	5***
MUFOLD	1***	5***
GUIJUNLAB-DEEPDA	1***	5/4***/1**
GUIJUNLAB-ASSEMBLY	1***	5/4***/1**
AF2-MULTIMER	1***	5/4***/1**
GINOBIFOLD	1***	4***
YANG-MULTIMER	1***	4/3***
COLABFOLD	1***	5/2***
SHANGHAITECH-TS-SER	1**	4/1***/2**
MULTIFOLD	1**	5**
RAPTORX-MULTIMER	1**	5/4**
MANIFOLD-E	1***	3/1***/1**
FOLDEVER	1	3/1***
DFOLDING-SERVER	1***	3/1***
CLUSPRO	1***	2/1***
ULTRAFOLD_SERVER	1	2/1***
HDOCK	1**	2/1**
DFOLDING-REFINE	1**	2/1**

Scorers and Scoring Servers	Top-1	Top-5
Oliva	1***	4/3***/1**
Fernandez-Recio	1***	3***
HDOCK	0	1**
S_Huang	0	1**
Zou	0	1
MDOCKPP	0	1

Table S2 – Prediction results for Target/Interface T209.1

Predictors	Top-1	Top-5
Wei_Zheng	1***	2***
Toshiyuki_Oda-PEZYFoldings	0	2/1***
Suwen_Zhao	0	2/1***
Wallner	1***	1***
Jianyi_Yang	1***	1***
Kihara	1	2/1**
S_Chang	0	1**
YiTing_Chen	0	3
Shan_Chang-CoDock	0	1**
Sieradzan	0	1

Servers	Top-1	Top-5
YANG-MULTIMER	1***	1***
LZERD	1	4/2**
KIHARA_SERVER	1	4/2**

Scorers and Scoring Servers	Top-1	Top-5
MDOCKPP	0	1**
S_Chang	0	1**

Table S2 – Prediction results for Target/Interface T216.1

Predictors	Top-1	Top-5
no acceptable models		

Servers	Top-1	Top-5
no acceptable models		

Scorers and Scoring Servers	Top-1	Top-5
no acceptable models		

Table S2 – Prediction results for Target/Interface T217.1

Predictors	Top-1	Top-5
no acceptable models		

Servers	Top-1	Top-5
no acceptable models		

Scorers and Scoring Servers	Top-1	Top-5
MDOCKPP	0	1

Table S2 – Prediction results for Target/Interface T218.1

**Predictors**	Top-1	Top-5
Junlin_Wang-MUFold_H	1**	5/1***/4**
J_Cheng	1**	5**
Gray	1**	5**
Zhigang_Sun-GinobiFold	1**	5**
Xinqi_Gong-BeijingAIProtein	1**	5**
Wei_Zheng	1**	5**
Suwen_Zhao	1**	5**
Qiwei_Ye-UltraFold	1**	5**
McGuffin	1**	5**
Jianyi_Yang	1**	5**
Elofsson	1**	5**
Anqi_Pang-Coqualia	1**	5**
Wenyi_Zhang	1**	5/4**
AIlab-trComplex	1**	5/4**
Sieradzan	1**	4**
Kihara	1**	4**
Takeda-Shitaka	1**	4**
Ovchinnikov-ColabFold	1**	4**
Xuyang_Liu-Manifold	1**	4/3**
Hongjiang_Miao-TRFold	1**	4/3**
Venclovas	1**	3**
S_Chang	1	5/2**
Pierce	1**	3**
Fernandez-Recio	1**	3**
Shan_Chang-CoDock	1	5/2**
S_Huang	1**	3/2**
Grudinin	1**	3/2**
DelCarpio	1	4
Zou	1**	1**
Kozakov	1**	1**
Wallner	1**	1**
Toshiyuki_Oda-PEZYFoldings	1**	1**
Ruihan_Guo	1**	1**
KozakovVajda	1**	1**
Jie_Hou-FoldEver-Hybrid	1**	1**
David_Jones-DMP	1**	1**
Shen	0	1
Y_Sun	0	1

Table S2 – Prediction results for Target/Interface T218.1

Servers	Top-1	Top-5
MULTICOM	1**	5**
LZERD	1**	5**
YANG-MULTIMER	1**	5**
ULTRAFOLD_SERVER	1**	5**
SHANGHAITECH-TS-SER	1**	5**
RAPTORX-MULTIMER	1**	5**
MULTIFOLD	1**	5**
MULTICOM_QA	1**	5**
MULTICOM_DEEP	1**	5**
MUFOLD	1**	5**
KIHARA_SERVER	1**	5**
GINOBIFOLD	1**	5**
AF2-MULTIMER	1**	5**
DFOLDING-SERVER	1**	3/1***/1**
COLABFOLD	1**	4**
MANIFOLD-E	1**	4/3**
HDOCK	1**	3/2**
DFOLDING-REFINE	1**	3/2**
MDOCKPP	1**	1**
CLUSPRO	1**	1**
FOLDEVER	1**	1**

Scorers and Scoring Servers	Top-1	Top-5
Takeda-Shitaka	1**	5**
J_Cheng	1**	5/4**
Oliva	1**	4**
HDOCK	1**	3/2**
S_Huang	1**	3/2**
Kihara	0	2**
Fernandez-Recio	0	2**
Venclovas	1**	3/1**
S_Chang	1	2/1**
Bonvin	0	1**
Zou	1**	1**
LZERD	0	1

Table S2 – Prediction results for Target/Interface T195.1

**Predictors**	Top-1	Top-5
Venclovas	1**	4**
Allab-trComplex	1**	4/3**
Baker	1**	3**
Sieradzan	1	5/1**
S_Chang	1	5/1**
Toshiyuki_Oda-PEZYFoldings	1**	3/2**
Hongjiang_Miao-TRFold	1	3/2**
Jianyi_Yang	1**	2**
Pierce	1	5
Kozakov	1	5
Wei_Zheng	1	5
Zou	1	3
Junlin_Wang-MUFold_H	0	3
Fang_Bai	1	3
Elofsson	0	2
Shen	1	1
Fernandez-Recio	0	1
McGuffin	0	1

**Servers**	Top-1	Top-5
AF2-MULTIMER	1**	3**
YANG-MULTIMER	1**	2**
CLUSPRO	1	5
DFOLDING-SERVER	1**	3/1**
MDOCKPP	1	3
MULTIFOLD	0	1

**Scorers and Scoring Servers**	Top-1	Top-5
Fernandez-Recio	1**	5**
Kihara	1**	5/3**
LZERD	0	4/3**
Bonvin	1	5/2**
Venclovas	1**	4/2**
Zou	1	5
MULTICOM	1	5
MDOCKPP	1	5
Oliva	1	5
HDOCK	1	5
S_Huang	1	5
Takeda-Shitaka	1	4
S_Chang	1	4

Table S2 – Prediction results for Target/Interface T203.1

**Predictors**	Top-1	Top-5
Zou	1***	5***
S_Huang	1***	5***
S_Chang	1***	5***
Pierce	1***	5***
Kihara	1***	5***
J_Cheng	1***	5***
Gray	1***	5***
Fernandez-Recio	1***	5***
Bates	1***	5***
Wei_Zheng	1***	5***
Grudinin	1***	5***
Fang_Bai	1***	5***
Shen	1***	5/4***/1**
McGuffin	1***	5/4***/1**
Elofsson	1***	5/4***/1**
Zechen_Wang-AIchemy_LIG2	0	4***
Toshiyuki_Oda-PEZYFoldings	1***	4***
Suwen_Zhao	1***	4***
Ovchinnikov-ColabFold	1***	4***
Hongjiang_Miao-TRFold	1***	4***
AIlab-trComplex	1***	4***
Y_Sun	1***	4/3***/1**
Zhigang_Sun-GinobiFold	0	3***
Xinqi_Gong-BeijingAIProtein	1***	3***
Xiao_Jia-AIchemy_LIG3	0	3***
Xianming_Pan	1***	3***
Wallner	0	3***
Junlin_Wang-MUFold_H	1***	3***
Jianyi_Yang	1***	3***
Gwang_So-FTBiot0119	0	3***
Anqi_Pang-Coqualia	1***	3***
Venclovas	1**	4/2***/2**
Jimenez-Garcia	1***	4/2***/2**
Xuyang_Liu-Manifold	0	3/2***/1**
Takeda-Shitaka	0	2***
Shan_Chang-CoDock	1***	2***
Qiwei_Ye-UltraFold	1***	2***
Liangzhen_Zheng-AIchemy_LIG	1***	2***
Baker	0	2***
Sieradzan	1**	5**
Kozakov	1**	5**
Wenyi_Zhang	1**	5**
KozakovVajda	1**	4**
GuijunLab	0	1***
David_Jones-DMP	1***	1***

Table S2 – Prediction results for Target/Interface T203.1

**Servers**	Top-1	Top-5
MULTICOM	1***	5***
MDOCKPP	1***	5***
LZERD	1***	5***
HDOCK	1***	5***
YANG-MULTIMER	1***	5***
YANG-SERVER	1***	4***
ULTRAFOLD_SERVER	1***	4***
SHANGHAITECH-TS-SER	1***	4***
MULTICOM_QA	1***	4***
MULTICOM_DEEP	1***	4***
KIHARA_SERVER	1***	4***
COLABFOLD	1***	4***
AF2-MULTIMER	1***	4***
MUFOLD	1***	3***
GUIJUNLAB-ASSEMBLY	0	3***
DFOLDING-REFINE	0	3***
MANIFOLD-E	0	3/2***/1**
GUIJUNLAB-DEEPDA	1***	2***
GINOBIFOLD	0	2***
CLUSPRO	1**	5**
MULTIFOLD	1***	3/1***/2**

**Scorers and Scoring Servers**	Top-1	Top-5
MULTICOM	1***	5***
Zou	1***	5***
MDOCKPP	1***	5***
LZERD	1***	5***
Kihara	1***	5***
Venclovas	1***	5***
Oliva	1***	5***
Takeda-Shitaka	1***	5***
S_Chang	1***	5/4***/1**
J_Cheng	1***	5/4***
Bonvin	1**	5/3***/2**
Fernandez-Recio	1***	5/2***/3**
Shen	0	4/2***/1**
HDOCK	1**	5/1***/4**
S_Huang	1**	5/1***/4**

Table S2 – Prediction results for Target/Interface T203.2

Predictors	Top-1	Top-5
S_Huang	1	5
Pierce	1	5
J_Cheng	1	5
Gray	1	5
Wenyi_Zhang	1	5
Wei_Zheng	1	4
Toshiyuki_Oda-PEZYFoldings	1	4
Takeda-Shitaka	1	4
Fang_Bai	1	4
S_Chang	1	3
Kozakov	1	3
Kihara	0	3
Xinqi_Gong-BeijingAIProtein	1	3
Qiwei_Ye-UltraFold	1	3
Zou	1	2
Venclovas	1	2
Fernandez-Recio	0	2
Shan_Chang-CoDock	1	2
KozakovVajda	1	2
Hongjiang_Miao-TRFold	0	2
Baker	1	2
AIlab-trComplex	0	2
Sieradzan	0	1
Wallner	1	1
Kazuki_Yamamoto-ddquest	1	1
Elofsson	0	1
David_Jones-DMP	1	1

Servers	Top-1	Top-5
HDOCK	1	5
MULTICOM	1	4
MULTICOM_QA	1	4
MULTICOM_DEEP	1	4
CLUSPRO	1	3
ULTRAFOLD_SERVER	1	3
MDOCKPP	1	2
FOLDEVER	0	2
DFOLDING-REFINE	1	2

Scorers and Scoring Servers	Top-1	Top-5
LZERD	1	5
S_Chang	1	5
Venclovas	1	5
Oliva	1	5
Fernandez-Recio	1	5
MDOCKPP	1	5
Bonvin	1	4
Takeda-Shitaka	1	4
Zou	1	4
Kihara	0	3
HDOCK	0	3
S_Huang	0	3
MULTICOM	0	1
Shen	1	1

Table S2 – Prediction results for Target/Interface T203.3

**Predictors**	Top-1	Top-5
Wenyi_Zhang	1**	5/1**
Fang_Bai	1**	4/1**
S_Huang	1	5
S_Chang	1	5
Pierce	1	5
Kozakov	1	5
Gray	1	5
Venclovas	1	4
Wei_Zheng	1	4
Toshiyuki_Oda-PEZYFoldings	1	4
Takeda-Shitaka	1	4
Shan_Chang-CoDock	1	4
Xinqi_Gong-BeijingAIProtein	1	3
Wallner	1	3
Qiwei_Ye-UltraFold	1	3
KozakovVajda	1	3
Zou	1	2
J_Cheng	1	2
Fernandez-Recio	0	2
Hongjiang_Miao-TRFold	0	2
Baker	1	2
AIlab-trComplex	0	2
McGuffin	1	1
Kazuki_Yamamoto-ddquest	1	1
Jianyi_Yang	0	1
Elofsson	0	1
David_Jones-DMP	1	1

**Servers**	Top-1	Top-5
HDOCK	1	5
CLUSPRO	1	5
MULTICOM	1	4
MULTICOM_QA	1	4
MULTICOM_DEEP	1	4
ULTRAFOLD_SERVER	1	3
MDOCKPP	1	2
YANG-SERVER	0	1
YANG-MULTIMER	0	1
DFOLDING-REFINE	0	1

**Scorers and Scoring Servers**	Top-1	Top-5
LZERD	1	5
Bonvin	1	5
S_Chang	1	5
Venclovas	1	5
Takeda-Shitaka	1	5
Oliva	1	5
Fernandez-Recio	1	5
MDOCKPP	1	5
Zou	1	4
HDOCK	0	4
S_Huang	0	4
Kihara	0	2
MULTICOM	0	1

Table S2 – Prediction results for Target/Interface T203.4

**Predictors**	Top-1	Top-5
S_Chang	1**	5/3**
Shan_Chang-CoDock	1**	4/3**
Venclovas	0	2/1**
Sieradzan	0	3
Kazuki_Yamamoto-ddquest	1**	1**
Baker	1	2
Pierce	0	1
Kihara	0	1
Junlin_Wang-MUFold_H	0	1
Jianyi_Yang	1	1
Fang_Bai	0	1

**Servers**	Top-1	Top-5
YANG-SERVER	1	2
YANG-MULTIMER	1	2
LZERD	0	1
KIHARA_SERVER	0	1

**Scorers and Scoring Servers**	Top-1	Top-5
S_Chang	1**	2**
Kihara	0	2
Takeda-Shitaka	0	1
HDOCK	1	1
LZERD	1	1
S_Huang	1	1
J_Cheng	0	1
Shen	0	1

Table S2 – Prediction results for Target/Interface T204.1

**Predictors**	Top-1	Top-5
Kozakov	1**	5**
Wei_Zheng	1**	5**
Xuyang_Liu-Manifold	1	5/2**
S_Huang	1	5
S_Chang	1	5
Kihara	1	5
Shan_Chang-CoDock	1	5
Gwang_So-FTBiot0119	1	5
Jianyi_Yang	1	3
Venclovas	1	2
Baker	1	2
Toshiyuki_Oda-PEZYFoldings	1	1
Schneidman	1	1
DFolding	1	1

**Servers**	Top-1	Top-5
CLUSPRO	1**	5**
MANIFOLD-E	1	5/2**
HDOCK	1	5
DFOLDING-SERVER	1	5
DFOLDING-REFINE	1	5
YANG-MULTIMER	1	3
KIHARA_SERVER	1	3
LZERD	0	2

**Scorers and Scoring Servers**	Top-1	Top-5
J_Cheng	1	5
S_Chang	1	5
MULTICOM	1	5
Zou	1	5
MDOCKPP	1	5
Kihara	1	5
Venclovas	1	5
Fernandez-Recio	1	5
Takeda-Shitaka	1	5
LZERD	1	5
HDOCK	1	5
S_Huang	1	5
Oliva	1	4
Bonvin	0	1

Table S2 – Prediction results for Target/Interface T204.2

**Predictors**	Top-1	Top-5
Wei_Zheng	1**	4/3**
S_Chang	1	5
Kozakov	1	5
Kihara	1	5
Shan_Chang-CoDock	1	5
Gwang_So-FTBiot0119	1	4
Xuyang_Liu-Manifold	1	3
Jianyi_Yang	1	3
Venclovas	1	1
Schneidman	1	1

**Servers**	Top-1	Top-5
CLUSPRO	1	5
YANG-MULTIMER	1	3
KIHARA_SERVER	1	3
LZERD	0	2

**Scorers and Scoring Servers**	Top-1	Top-5
Zou	1	5
MDOCKPP	1	5
Venclovas	1	5
LZERD	1	5
S_Chang	1	5
J_Cheng	1	5
Fernandez-Recio	1	5
Kihara	1	4
Oliva	1	4
HDOCK	1	4
S_Huang	1	4
Takeda-Shitaka	1	3
Bonvin	0	1

Table S2 – Prediction results for Target/Interface T204.3

Predictors	Top-1	Top-5
S_Huang	1***	5***
S_Chang	1***	5***
Shan_Chang-CoDock	1***	5***
Wei_Zheng	1**	4/1***/3**
Takeda-Shitaka	0	2***
Pierce	1**	5**
Kozakov	1**	5**
Kihara	1**	5**
Suwen_Zhao	1**	5**
Jianyi_Yang	1**	5**
Elofsson	1**	5**
Baker	1**	5**
Sieradzan	1**	5/4**
Venclovas	1**	4**
Xuyang_Liu-Manifold	1**	4**
Junlin_Wang-MUFold_H	1**	4**
Wallner	1**	3**
Schneidman	1***	1***
Fang_Bai	0	1***
DFolding	1***	1***
Grudinin	1**	2**
Bates	1	5
GuijunLab	0	2/1**
Shen	1**	1**
Zhigang_Sun-GinobiFold	1**	1**
Toshiyuki_Oda-PEZYFoldings	0	1**
Y_Sun	1**	1**
Qiwei_Ye-UltraFold	1	3
McGuffin	1**	1**
Anqi_Pang-Coqualia	1**	1**
Sachin_Kadyan-OpenFold-SingleSeq	1	1
Sachin_Kadyan-OpenFold	1	1

Servers	Top-1	Top-5
HDOCK	1***	5***
DFOLDING-SERVER	1***	5***
DFOLDING-REFINE	1***	5***
MULTICOM	1**	5**
CLUSPRO	1**	5**
YANG-MULTIMER	1**	5**
MULTICOM_QA	1**	5**
MULTICOM_DEEP	1**	5**
MUFOLD	1**	4**
KIHARA_SERVER	1**	3**
LZERD	0	2**
AF2-MULTIMER	1**	2**
ULTRAFOLD_SERVER	1	3
MULTIFOLD	1**	1**
GUIJUNLAB-DEEPDA	0	1**

Scorers and Scoring Servers	Top-1	Top-5
MULTICOM	1***	5***
Zou	1***	5***
MDOCKPP	1***	5***
Venclovas	1***	5***
LZERD	1***	5***
Fernandez-Recio	1***	5***
HDOCK	1***	5***
S_Huang	1***	5***
Oliva	1***	5/4***/1**
S_Chang	1***	5/4***/1**
Kihara	1**	4/2***/2**
Bonvin	1**	5/1***/4**
J_Cheng	1**	5**
Takeda-Shitaka	1**	3**
Shen	0	1**

Table S2 – Prediction results for Target/Interface T204.4

**Predictors**	Top-1	Top-5
S_Huang	1***	5***
S_Chang	1***	5***
J_Cheng	1***	5***
Shan_Chang-CoDock	1***	5***
Jianyi_Yang	1***	5/4***/1**
Wei_Zheng	1***	4***
Baker	1**	5/3***/2**
Junlin_Wang-MUFold_H	1***	4/2***/2**
Xuyang_Liu-Manifold	1**	4/1***/3**
Takeda-Shitaka	0	2***
Kihara	1**	5**
Suwen_Zhao	1**	5**
Venclovas	1**	3**
Schneidman	1***	1***
McGuffin	1***	1***
Fang_Bai	0	1***
DFolding	1***	1***
Wallner	1**	2**
Grudinin	1**	2**
Sieradzan	1	3
Zhigang_Sun-GinobiFold	1**	1**
Toshiyuki_Oda-PEZYFoldings	0	1**
Ruihan_Guo	1**	1**
Jie_Hou-FoldEver-Hybrid	1**	1**
GuijunLab	0	1**
Elofsson	0	1**
Anqi_Pang-Coqualia	1**	1**

**Servers**	Top-1	Top-5
MULTICOM	1***	5***
HDOCK	1***	5***
MULTICOM_QA	1***	5***
MULTICOM_DEEP	1***	5***
DFOLDING-SERVER	1***	5***
DFOLDING-REFINE	1***	5***
MUFOLD	1***	4/2***/2**
YANG-MULTIMER	1**	5/1***/4**
MULTIFOLD	1***	1***
AF2-MULTIMER	1**	2**
GUIJUNLAB-DEEPDA	0	1**
FOLDEVER	1**	1**

**Scorers and Scoring Servers**	Top-1	Top-5
MULTICOM	1***	5***
Zou	1***	5***
MDOCKPP	1***	5***
Venclovas	1***	5***
LZERD	1***	5***
Fernandez-Recio	1***	5***
HDOCK	1***	5***
S_Huang	1***	5***
S_Chang	1***	5/4***/1**
Oliva	1***	4***
Kihara	1**	4/2***/2**
Bonvin	0	2/1***/1**
Takeda-Shitaka	1**	3**
Shen	0	1***

Table S2 – Prediction results for Target/Interface T204.5

**Predictors**	Top-1	Top-5
Pierce	1***	5***
Kihara	1***	5***
Baker	1***	5***
J_Cheng	1***	5/4***/1**
Jianyi_Yang	1**	5/4***/1**
Wei_Zheng	1***	4***
S_Chang	1***	5/3***/2**
Shan_Chang-CoDock	1***	5/3***/2**
Venclovas	1***	3***
Grudinin	1***	2***
Junlin_Wang-MUFold_H	1***	3/1***/2**
Xuyang_Liu-Manifold	1**	4**
Wallner	1***	2/1***/1**
Schneidman	1***	1***
GuijunLab	0	1***
Fang_Bai	0	1***
DFolding	1***	1***
Elofsson	1**	2/1**
Toshiyuki_Oda-PEZYFoldings	0	1**
Suwen_Zhao	1**	1**
Ruihan_Guo	1**	1**
Shen	1	1
Y_Sun	1	1

**Servers**	Top-1	Top-5
MULTICOM	1***	5***
MULTICOM_QA	1***	5***
MULTICOM_DEEP	1***	5***
DFOLDING-SERVER	1***	5/4***/1**
DFOLDING-REFINE	1***	5/4***/1**
YANG-MULTIMER	1***	5/3***/2**
AF2-MULTIMER	1***	2***
MUFOLD	1***	3/1***/2**
GUIJUNLAB-DEEPDA	0	1***

**Scorers and Scoring Servers**	Top-1	Top-5
Fernandez-Recio	1***	5***
LZERD	1**	5/4***/1**
S_Chang	1***	5/3***/2**
Kihara	1***	4/3***/1**
Takeda-Shitaka	1***	3***
Zou	1**	5/1***/4**
MDOCKPP	1**	5/1***/4**
Venclovas	1**	5/1***/4**
HDOCK	1**	4/1***/3**
S_Huang	1**	4/1***/3**
Oliva	1**	4**
Bonvin	0	2/1***/1**
Shen	0	1***

Table S2 – Prediction results for Target/Interface T204.6

Predictors	Top-1	Top-5
S_Chang	1**	5**
Shan_Chang-CoDock	1**	5**
Jianyi_Yang	1**	5**
Baker	1**	5**
Xuyang_Liu-Manifold	1**	4**
Venclovas	1	5/2**
S_Huang	1	5
Pierce	1	5
J_Cheng	1	3/1**
Kihara	1	4
Shen	1**	1**
Wei_Zheng	0	1**
Takeda-Shitaka	0	1**
Y_Sun	1**	1**
Schneidman	1**	1**
DFolding	1**	1**
Wallner	0	2
David_Jones-DMP	1	1

Servers	Top-1	Top-5
YANG-MULTIMER	1**	5**
DFOLDING-REFINE	1**	5**
DFOLDING-SERVER	1**	4**
HDOCK	1	5
MULTICOM_DEEP	0	2/1**
MULTICOM	0	2
MULTICOM_QA	0	2

Scorers and Scoring Servers	Top-1	Top-5
Zou	1**	5**
MDOCKPP	1**	5**
Venclovas	1**	5**
Oliva	1**	5**
LZERD	1**	5**
Fernandez-Recio	1**	5**
Bonvin	1**	5/4**
S_Chang	1**	5/4**
HDOCK	1**	5/4**
S_Huang	1**	5/4**
Kihara	1	4/2**
MULTICOM	1	5
Takeda-Shitaka	1	3

Table S2 – Prediction results for Target/Interface T204.7

**Predictors**	Top-1	Top-5
Venclovas	1**	5/1***/4**
S_Chang	1**	5/1***/4**
Xuyang_Liu-Manifold	1**	4/1***/3**
Kozakov	1**	5**
Kihara	1**	5**
Elofsson	1**	3/1***/2**
Baker	1**	5**
Wei_Zheng	1**	2/1***/1**
Sieradzan	1	5/2**
Wallner	1**	3**
DFolding	1***	1***
Pierce	1**	5/1**
Grudinin	1**	3/2**
Bates	1	5
GuijunLab	1	3/1**
Shen	1**	1**
Shan_Chang-CoDock	0	1**
McGuffin	1**	1**

**Servers**	Top-1	Top-5
MULTICOM_DEEP	1***	5/3***/2**
DFOLDING-SERVER	1***	5/3***/2**
DFOLDING-REFINE	1***	5/3***/2**
MULTICOM	1**	5/2***/3**
MULTICOM_QA	1**	5/2***/3**
KIHARA_SERVER	1***	3***
LZERD	0	2***
CLUSPRO	1**	5**
AF2-MULTIMER	1**	3/2**
GUIJUNLAB-DEEPDA	1	3/1**
MULTIFOLD	1**	1**
GUIJUNLAB-ASSEMBLY	0	1

**Scorers and Scoring Servers**	Top-1	Top-5
J_Cheng	1***	5***
S_Chang	1**	5/1***/4**
LZERD	1**	5/1***/4**
Bonvin	1**	5**
Venclovas	1**	5**
Oliva	1**	5**
Zou	1**	5**
MDOCKPP	1**	5**
Fernandez-Recio	1**	5**
Kihara	1**	4**
HDOCK	1**	4**
S_Huang	1**	4**
Takeda-Shitaka	1**	3**
Shen	0	1***

Table S2 – Prediction results for Target/Interface T204.8

**Predictors**	Top-1	Top-5
S_Huang	1***	5***
Pierce	1***	5***
Kihara	1***	5***
Venclovas	1***	5/4***/1**
J_Cheng	1***	5/4***/1**
Kozakov	1***	5/3***/2**
Takeda-Shitaka	1***	3***
Gwang_So-FTBiot0119	1***	3***
S_Chang	1**	5**
Bates	1**	5**
Sieradzan	0	3

**Servers**	Top-1	Top-5
MULTICOM	1***	5***
HDOCK	1***	5***
CLUSPRO	1***	5/3***/2**

**Scorers and Scoring Servers**	Top-1	Top-5
Takeda-Shitaka	1***	3***
Kihara	1***	2***
S_Chang	0	1***
Bonvin	1**	3**
Oliva	0	1**

Table S2 – Prediction results for Target/Interface T219.1

**Predictors**	Top-1	Top-5
Zou	1**	5**
Pierce	1**	5**
Kihara	1**	5**
J_Cheng	1**	5**
Bates	1**	5**
Xinqi_Gong-BeijingAIProtein	1**	5**
Takeda-Shitaka	1**	5**
Qiwei_Ye-UltraFold	1**	5**
Junlin_Wang-MUFold_H	1**	5**
Jianyi_Yang	1**	5**
Fang_Bai	1**	5**
Kozakov	1**	5/4**
Ovchinnikov-ColabFold	1**	5/4**
Elofsson	1**	5/4**
Xuyang_Liu-Manifold	1**	4**
Wei_Zheng	1**	4**
Venclovas	1**	5/3**
Gray	1	5/3**
KozakovVajda	0	4/3**
Toshiyuki_Oda-PEZYFoldings	1**	5/2**
MyongHo_Chae	1**	3**
Junhan_Chang-Manifold-X	1**	3**
Grudinin	1**	3**
S_Chang	1	5/1**
Shan_Chang-CoDock	1	5/1**
GuijunLab	1**	2**
Shen	1	5
Y_Sun	1	5
S_Huang	1**	2/1**
Wallner	1**	2/1**
Baker	1	4
Ness	1**	1**
McGuffin	0	1**
Kazuki_Yamamoto-ddquest	1**	1**
Hongjiang_Miao-TRFold	0	1
AIlab-trComplex	0	1

Table S2 – Prediction results for Target/Interface T219.1

**Servers**	Top-1	Top-5
MULTICOM	1**	5**
MDOCKPP	1**	5**
YANG-SERVER	1**	5**
ULTRAFOLD_SERVER	1**	5**
RAPTORX-MULTIMER	1**	5**
MULTICOM_DEEP	1**	5**
KIHARA_SERVER	1**	5**
AF2-MULTIMER	1**	5**
CLUSPRO	1**	5/4**
COLABFOLD	1**	5/4**
MULTICOM_QA	1**	4**
MUFOLD	1**	4**
MANIFOLD-LC-E	1**	4**
MANIFOLD-E	1**	4**
DFOLDING-SERVER	1**	4**
YANG-MULTIMER	1**	4/3**
DFOLDING-REFINE	0	3**
GUIJUNLAB-DEEPDA	0	2**
HDOCK	1**	2/1**
MULTIFOLD	0	1**
GUIJUNLAB-ASSEMBLY	0	1**

**Scorers and Scoring Servers**	Top-1	Top-5
Venclovas	1**	5**
Takeda-Shitaka	1**	5**
Zou	1**	5/4**
S_Chang	1	5/3**
Bonvin	1**	4/3**
MDOCKPP	1**	4/3**
Kihara	1**	3**
Fernandez-Recio	1	5/2**
HDOCK	1**	4/2**
S_Huang	1**	4/2**
Oliva	1	4/1**
MULTICOM	1**	1**

Table S2 – Prediction results for Target/Interface T219.2

**Predictors**	Top-1	Top-5
Zou	1**	5**
Venclovas	1**	5**
S_Chang	1**	5**
Pierce	1**	5**
Kozakov	1**	5**
Kihara	1**	5**
J_Cheng	1**	5**
Wei_Zheng	1**	5**
Shan_Chang-CoDock	1**	5**
Qiwei_Ye-UltraFold	1**	5**
Ovchinnikov-ColabFold	1**	5**
Junlin_Wang-MUFold_H	1**	5**
Jianyi_Yang	1**	5**
Fang_Bai	1**	5**
Baker	1**	5**
Shen	1	5/4**
Y_Sun	1	5/4**
Xinqi_Gong-BeijingAIProtein	0	4**
McGuffin	0	4**
KozakovVajda	1**	4**
Junhan_Chang-Manifold-X	1**	4**
Hongjiang_Miao-TRFold	1**	4**
Grudinin	1**	4**
Gray	1	5/3**
Toshiyuki_Oda-PEZYFoldings	1**	4/3**
Xuyang_Liu-Manifold	1**	3**
Takeda-Shitaka	0	3**
Elofsson	0	3**
MyongHo_Chae	1**	3/2**
AIlab-trComplex	1**	3/2**
GuijunLab	1**	2**
Bates	1	5
S_Huang	1**	2/1**
Wallner	1**	2/1**
Sieradzan	1	3
Ness	1**	1**
Kazuki_Yamamoto-ddquest	1**	1**

Table S2 – Prediction results for Target/Interface T219.2

**Servers**	Top-1	Top-5
MULTICOM	1**	5**
MDOCKPP	1**	5**
CLUSPRO	1**	5**
YANG-SERVER	1**	5**
ULTRAFOLD_SERVER	1**	5**
MULTICOM_QA	1**	5**
MULTICOM_DEEP	1**	5**
COLABFOLD	1**	5**
RAPTORX-MULTIMER	1**	4**
MULTIFOLD	0	4**
MUFOLD	1**	4**
MANIFOLD-LC-E	1**	4**
MANIFOLD-E	1**	4**
KIHARA_SERVER	1**	4**
DFOLDING-SERVER	1**	4**
AF2-MULTIMER	1**	4**
YANG-MULTIMER	1**	3**
DFOLDING-REFINE	0	3**
GUIJUNLAB-DEEPDA	0	2**
GUIJUNLAB-ASSEMBLY	0	2**
HDOCK	1**	2/1**

**Scorers and Scoring Servers**	Top-1	Top-5
Venclovas	1**	5**
MDOCKPP	1**	5**
Zou	1**	5**
Takeda-Shitaka	1**	5**
S_Chang	1**	5/4**
Fernandez-Recio	1**	5/4**
Bonvin	1**	5/4**
HDOCK	1**	4**
S_Huang	1**	4**
Oliva	1**	4/3**
Kihara	1**	3**
MULTICOM	1**	1**

Table S2 – Prediction results for Target/Interface T219.3

**Predictors**	Top-1	Top-5
Zou	1	5
S_Chang	1	5
Pierce	1	5
Kozakov	1	5
Kihara	1	5
J_Cheng	1	5
Gray	1	5
Bates	1	5
Shan_Chang-CoDock	1	5
Qiwei_Ye-UltraFold	1	5
Ovchinnikov-ColabFold	1	5
KozakovVajda	1	5
Jianyi_Yang	1	5
Fang_Bai	1	5
Elofsson	1	5
Shen	1	4
Xuyang_Liu-Manifold	1	4
Xinqi_Gong-BeijingAIProtein	1	4
Takeda-Shitaka	1	4
Y_Sun	1	4
Junlin_Wang-MUFold_H	0	4
Junhan_Chang-Manifold-X	1	4
Grudinin	1	4
Venclovas	1	3
Wallner	1	3
Toshiyuki_Oda-PEZYFoldings	0	3
MyongHo_Chae	1	3
S_Huang	1	2
Hongjiang_Miao-TRFold	0	2
GuijunLab	1	2
Wei_Zheng	0	1
Kazuki_Yamamoto-ddquest	1	1
AIlab-trComplex	0	1

Table S2 – Prediction results for Target/Interface T219.3

Servers	Top-1	Top-5
MULTICOM	1	5
MDOCKPP	1	5
CLUSPRO	1	5
ULTRAFOLD_SERVER	1	5
RAPTORX-MULTIMER	1	5
MULTICOM_DEEP	1	5
MUFOLD	1	5
AF2-MULTIMER	1	5
YANG-SERVER	1	4
YANG-MULTIMER	1	4
MULTICOM_QA	1	4
MANIFOLD-LC-E	1	4
MANIFOLD-E	1	4
KIHARA_SERVER	1	4
DFOLDING-SERVER	1	4
COLABFOLD	1	4
DFOLDING-REFINE	0	3
HDOCK	1	2
GUIJUNLAB-DEEPDA	0	2
GUIJUNLAB-ASSEMBLY	0	2
MULTIFOLD	0	1

Scorers and Scoring Servers	Top-1	Top-5
Zou	1	5
Venclovas	1	5
S_Chang	1	5
Takeda-Shitaka	1	5
MDOCKPP	1	4
Bonvin	1	4
Oliva	1	4
Fernandez-Recio	0	4
HDOCK	1	4
S_Huang	1	4
Kihara	1	3
MULTICOM	1	1
LZERD	0	1

Table S2 – Prediction results for Target/Interface T220.1

**Predictors**	Top-1	Top-5
Zou	1**	5**
Pierce	1**	5**
Kozakov	1**	5**
Kihara	1**	5**
J_Cheng	1**	5**
Gray	1**	5**
Bates	1**	5**
Takeda-Shitaka	1**	5**
Ovchinnikov-ColabFold	1**	5**
Ness	1**	5**
McGuffin	1**	5**
KozakovVajda	1**	5**
Jianyi_Yang	1**	5**
Fang_Bai	1**	5**
Elofsson	1**	5**
Xuyang_Liu-Manifold	1**	5/4**
Wei_Zheng	1**	5/4**
Junhan_Chang-Manifold-X	1**	5/4**
Zechen_Wang-AIchemy_LIG2	1**	4**
Xiao_Jia-AIchemy_LIG3	1**	4**
Liangzhen_Zheng-AIchemy_LIG	1**	4**
Junlin_Wang-MUFold_H	1**	4**
Venclovas	1	5/2**
S_Chang	1	5/2**
Shan_Chang-CoDock	1	5/2**
MyongHo_Chae	1**	3**
S_Huang	1**	3/2**
Xinqi_Gong-BeijingAIProtein	1**	5/1**
Wenyi_Zhang	1**	5/1**
Qiwei_Ye-UltraFold	1**	5/1**
Baker	1	5/1**
Shen	1	5
Y_Sun	1	5
GuijunLab	1	4
Ziheng_Zou	1**	1**
Wei_Lu	1**	1**
Ruihan_Guo	1**	1**

Table S2 – Prediction results for Target/Interface T220.1

**Servers**	Top-1	Top-5
MULTICOM	1**	5**
LZERD	1**	5**
CLUSPRO	1**	5**
MULTIFOLD	1**	5**
MULTICOM_QA	1**	5**
MULTICOM_DEEP	1**	5**
MUFOLD	1**	5**
KIHARA_SERVER	1**	5**
COLABFOLD	1**	5**
AF2-MULTIMER	1**	5**
MANIFOLD-LC-E	1**	5/4**
MANIFOLD-E	1**	5/4**
YANG-SERVER	1**	4**
RAPTORX-MULTIMER	0	4**
MDOCKPP	1**	5/3**
HDOCK	1**	3/2**
ULTRAFOLD_SERVER	1**	5/1**
GUIJUNLAB-DEEPDA	1	4
DFOLDING-SERVER	0	1**
DFOLDING-REFINE	0	1**
GUIJUNLAB-ASSEMBLY	0	1

**Scorers and Scoring Servers**	Top-1	Top-5
Takeda-Shitaka	1**	5**
Bonvin	1**	5**
Zou	1**	5/4**
MDOCKPP	1**	5/4**
MULTICOM	1**	5/4**
S_Chang	1	5/3**
Kihara	1**	3**
Fernandez-Recio	1**	5/2**
Oliva	1**	3**
LZERD	0	2**
HDOCK	0	2**
S_Huang	0	2**
J_Cheng	1**	3/1**
Venclovas	1	5

Table S2 – Prediction results for Target/Interface T220.2

Predictors	Top-1	Top-5
Zou	1**	5**
Kozakov	1**	5**
Kihara	1**	5**
J_Cheng	1**	5**
Ovchinnikov-ColabFold	1**	5**
KozakovVajda	1**	5**
McGuffin	1**	5/4**
Jianyi_Yang	1**	5/4**
Elofsson	1**	5/4**
Baker	1**	5/4**
Zechen_Wang-AIchemy_LIG2	1**	4**
Xiao_Jia-AIchemy_LIG3	1**	4**
Ness	1**	4**
Liangzhen_Zheng-AIchemy_LIG	1**	4**
Shen	1	5/3**
Wenyi_Zhang	1**	5/3**
Takeda-Shitaka	1**	5/3**
Y_Sun	1	5/3**
Junlin_Wang-MUFold_H	1**	5/3**
Fang_Bai	1	5/3**
Venclovas	1	5/2**
MyongHo_Chae	1**	3**
S_Chang	1	5/1**
Pierce	1	5/1**
Xuyang_Liu-Manifold	1	5/1**
Wei_Zheng	1	5/1**
Shan_Chang-CoDock	1	5/1**
Junhan_Chang-Manifold-X	1	5/1**
S_Huang	1	3/1**
Gray	1	5
Bates	1	5
Xinqi_Gong-BeijingAIProtein	1	5
Qiwei_Ye-UltraFold	1	5
GuijunLab	1	4
Ziheng_Zou	1**	1**
Wei_Lu	1**	1**
Ruihan_Guo	1**	1**

Table S2 – Prediction results for Target/Interface T220.2

**Servers**	Top-1	Top-5
MULTICOM	1**	5**
LZERD	1**	5**
CLUSPRO	1**	5**
RAPTORX-MULTIMER	1**	5**
MULTICOM_QA	1**	5**
MULTICOM_DEEP	1**	5**
KIHARA_SERVER	1**	5**
COLABFOLD	1**	5**
MULTIFOLD	1**	5/4**
AF2-MULTIMER	1**	5/4**
MDOCKPP	1**	5/3**
MUFOLD	1**	5/3**
YANG-SERVER	0	4/3**
MANIFOLD-LC-E	1	5/1**
MANIFOLD-E	1	5/1**
HDOCK	1	3/1**
ULTRAFOLD_SERVER	1	5
GUIJUNLAB-DEEPDA	1	5
GUIJUNLAB-ASSEMBLY	0	2

**Scorers and Scoring Servers**	Top-1	Top-5
Zou	1**	5**
MDOCKPP	1**	5**
Fernandez-Recio	1**	5**
Takeda-Shitaka	1**	5**
Bonvin	1**	5**
MULTICOM	1**	5/3**
J_Cheng	1	4/3**
Kihara	1**	3**
S_Chang	1	5/1**
Oliva	1**	3/2**
LZERD	0	2**
Venclovas	1	5
HDOCK	0	2/1**
S_Huang	0	2/1**

Table S2 – Prediction results for Target/Interface T220.3

**Predictors**	Top-1	Top-5
Zou	1**	5**
S_Chang	1**	5**
Pierce	1**	5**
Kozakov	1**	5**
Kihara	1**	5**
J_Cheng	1**	5**
Gray	1**	5**
Bates	1**	5**
Xuyang_Liu-Manifold	1**	5**
Takeda-Shitaka	1**	5**
Ness	1**	5**
McGuffin	1**	5**
KozakovVajda	1**	5**
Junlin_Wang-MUFold_H	1**	5**
Fang_Bai	1**	5**
Elofsson	1**	5**
Baker	1**	5**
Wenyi_Zhang	1**	5/4**
Qiwei_Ye-UltraFold	1**	5/4**
Zechen_Wang-AIchemy_LIG2	1**	4**
Xinqi_Gong-BeijingAIProtein	1**	4**
Wei_Zheng	1**	4**
Shan_Chang-CoDock	1**	4**
Jianyi_Yang	1**	4**
Venclovas	1	5/2**
Xiao_Jia-AIchemy_LIG3	1**	3**
Shen	1**	5/1**
S_Huang	1**	3/2**
MyongHo_Chae	1**	2**
Liangzhen_Zheng-AIchemy_LIG	1**	2**
Junhan_Chang-Manifold-X	0	2**
GuijunLab	1	4
Ziheng_Zou	1**	1**
Wei_Lu	1**	1**
Y_Sun	0	3
Ruihan_Guo	1**	1**
Ovchinnikov-ColabFold	0	1**

Table S2 – Prediction results for Target/Interface T220.3

Servers	Top-1	Top-5
MULTICOM	1**	5**
MDOCKPP	1**	5**
LZERD	1**	5**
CLUSPRO	1**	5**
YANG-SERVER	1**	5**
RAPTORX-MULTIMER	1**	5**
MULTICOM_QA	1**	5**
MULTICOM_DEEP	1**	5**
MUFOLD	1**	5**
MANIFOLD-LC-E	1**	5**
MANIFOLD-E	1**	5**
KIHARA_SERVER	1**	5**
COLABFOLD	1**	5**
AF2-MULTIMER	1**	5**
ULTRAFOLD_SERVER	1**	5/4**
MULTIFOLD	1**	3**
HDOCK	1**	3/2**
GUIJUNLAB-DEEPDA	1	4
DFOLDING-SERVER	0	1**
DFOLDING-REFINE	0	1**
GUIJUNLAB-ASSEMBLY	0	2

Scorers and Scoring Servers	Top-1	Top-5
Zou	1**	5**
MDOCKPP	1**	5**
Venclovas	1**	5**
Bonvin	1**	5**
S_Chang	1**	5**
Takeda-Shitaka	1**	5**
Fernandez-Recio	1**	5**
MULTICOM	1**	5**
J_Cheng	1**	5/3**
Kihara	1**	3**
Oliva	1**	3**
LZERD	0	2**
HDOCK	0	2**
S_Huang	0	2**

Table S2 – Prediction results for Target/Interface T220.4

**Predictors**	Top-1	Top-5
Zhigang_Sun-GinobiFold	1***	5***
YiTing_Chen	1***	5***
Anqi_Pang-Coqualia	1***	5***
Baker	1***	4***
Wenyi_Zhang	1**	4/2***/2**
Venclovas	1**	3/1***/2**
Kihara	1**	5**
Gray	1**	5**
Kozakov	1**	5/4**
Xuyang_Liu-Manifold	1**	4**
Junhan_Chang-Manifold-X	1**	4**
S_Chang	1**	5/3**
Shen	1**	4/3**
Y_Sun	1**	4/3**
J_Cheng	0	1***
KozakovVajda	1**	3**
Jie_Hou-FoldEver-Hybrid	1***	1***
Shan_Chang-CoDock	1**	4/2**
Zou	1**	3/2**
Takeda-Shitaka	1	4/1**
Pierce	1	5
Bates	1	5
Fang_Bai	1	3/1**
S_Huang	1	4
Zechen_Wang-AIchemy_LIG2	1	3
Xiao_Jia-AIchemy_LIG3	1	3
McGuffin	1	3
Liangzhen_Zheng-AIchemy_LIG	1	3
Elofsson	1	3
Wei_Zheng	0	2
Ziheng_Zou	1	1
Xinqi_Gong-BeijingAIProtein	0	1
Wei_Lu	1	1
Qiwei_Ye-UltraFold	0	1
Junlin_Wang-MUFold_H	0	1
Jianyi_Yang	0	1
GuijunLab	1	1

Table S2 – Prediction results for Target/Interface T220.4

**Servers**	Top-1	Top-5
SHANGHAITECH-TS-SER	1***	5***
GINOBIFOLD	1***	5***
DFOLDING-SERVER	1***	4***
DFOLDING-REFINE	1***	4***
CLUSPRO	1**	5/4**
MANIFOLD-LC-E	1**	4**
MANIFOLD-E	1**	4**
FOLDEVER	1***	1***
HDOCK	1	4
MUFOLD	0	3
AF2-MULTIMER	1	3
MULTICOM	0	1
YANG-SERVER	0	1
ULTRAFOLD_SERVER	0	1
MULTICOM_QA	0	1
MULTICOM_DEEP	0	1

**Scorers and Scoring Servers**	Top-1	Top-5
Fernandez-Recio	1**	5/2***/2**
Kihara	1**	3**
S_Chang	1**	5/2**
Zou	1	5/2**
MDOCKPP	1	5/2**
Bonvin	1**	4/2**
Venclovas	1**	3/2**
Takeda-Shitaka	1	4
Shen	0	2
Oliva	0	1

Table S2 – Prediction results for Target/Interface T221.1

**Predictors**	Top-1	Top-5
Zou	1**	5**
Pierce	1**	5**
Kozakov	1**	5**
Kihara	1**	5**
J_Cheng	1**	5**
Gray	1**	5**
Bates	1**	5**
Xuyang_Liu-Manifold	1**	5**
Wenyi_Zhang	1**	5**
Wei_Zheng	1**	5**
Takeda-Shitaka	1**	5**
Ovchinnikov-ColabFold	1**	5**
Ness	1**	5**
McGuffin	1**	5**
KozakovVajda	1**	5**
Junlin_Wang-MUFold_H	1**	5**
Junhan_Chang-Manifold-X	1**	5**
Fang_Bai	1**	5**
Elofsson	1**	5**
Jianyi_Yang	1**	5/4**
Zechen_Wang-AIchemy_LIG2	1**	4**
Xiao_Jia-AIchemy_LIG3	1**	4**
Liangzhen_Zheng-AIchemy_LIG	1**	4**
Baker	1**	5/3**
S_Chang	1	5/2**
Shan_Chang-CoDock	1	5/2**
S_Huang	1**	3/2**
Xinqi_Gong-BeijingAIProtein	1**	5/1**
Qiwei_Ye-UltraFold	1**	5/1**
Shen	1	3/1**
Venclovas	1	5
Y_Sun	1	3/1**
GuijunLab	1	5
Wei_Lu	1**	1**
Ruihan_Guo	1**	1**

Table S2 – Prediction results for Target/Interface T221.1

**Servers**	Top-1	Top-5
MULTICOM	1**	5**
LZERD	1**	5**
CLUSPRO	1**	5**
RAPTORX-MULTIMER	1**	5**
MULTIFOLD	1**	5**
MULTICOM_QA	1**	5**
MULTICOM_DEEP	1**	5**
MUFOLD	1**	5**
MANIFOLD-LC-E	1**	5**
MANIFOLD-E	1**	5**
KIHARA_SERVER	1**	5**
COLABFOLD	1**	5**
AF2-MULTIMER	1**	5**
YANG-SERVER	1**	5/4**
MDOCKPP	1**	5/3**
HDOCK	1**	3/2**
ULTRAFOLD_SERVER	1**	5/1**
GUIJUNLAB-DEEPDA	1	5
DFOLDING-SERVER	0	1**
DFOLDING-REFINE	0	1**

**Scorers and Scoring Servers**	Top-1	Top-5
Takeda-Shitaka	1**	5**
Bonvin	1**	5**
Zou	1**	5/4**
MDOCKPP	1**	5/4**
Fernandez-Recio	1**	5/3**
S_Chang	1	5/2**
Kihara	1**	3**
HDOCK	1**	3**
S_Huang	1**	3**
Venclovas	1	5/1**
MULTICOM	1**	2**
Oliva	1	2

Table S2 – Prediction results for Target/Interface T221.2

Predictors	Top-1	Top-5
Zou	1**	5**
Kozakov	1**	5/4**
Kihara	1	5/4**
KozakovVajda	1**	5/4**
Shen	1	5/3**
Y_Sun	1	5/3**
Venclovas	1	5
S_Huang	1	3/1**
S_Chang	1	5
Pierce	1	5
J_Cheng	1	5
Gray	1	5
Bates	1	5
Xuyang_Liu-Manifold	1	5
Xinqi_Gong-BeijingAIProtein	1	5
Wenyi_Zhang	1	5
Wei_Zheng	1	5
Takeda-Shitaka	1	5
Shan_Chang-CoDock	1	5
Qiwei_Ye-UltraFold	1	5
Ovchinnikov-ColabFold	1	5
Ness	1	5
McGuffin	1	5
Junlin_Wang-MUFold_H	1	5
Junhan_Chang-Manifold-X	1	5
Jianyi_Yang	1	5
GuijunLab	1	5
Fang_Bai	1	5
Elofsson	1	5
Baker	1	5
Zechen_Wang-AIchemy_LIG2	1	4
Xiao_Jia-AIchemy_LIG3	1	4
Liangzhen_Zheng-AIchemy_LIG	1	4
Ruihan_Guo	1**	1**
Wei_Lu	1	1

Table S2 – Prediction results for Target/Interface T221.2

**Servers**	Top-1	Top-5
MULTIFOLD	1**	5**
CLUSPRO	1**	5/4**
MDOCKPP	1**	5/3**
LZERD	1	5/2**
KIHARA_SERVER	1	5/2**
MULTICOM	1	5
HDOCK	1	3/1**
YANG-SERVER	1	5
ULTRAFOLD_SERVER	1	5
RAPTORX-MULTIMER	1	5
MULTICOM_QA	1	5
MULTICOM_DEEP	1	5
MUFOLD	1	5
MANIFOLD-LC-E	1	5
MANIFOLD-E	1	5
GUIJUNLAB-DEEPDA	1	5
COLABFOLD	1	5
AF2-MULTIMER	1	5
DFOLDING-SERVER	0	1**
DFOLDING-REFINE	0	1**

**Scorers and Scoring Servers**	Top-1	Top-5
Bonvin	1**	5**
Zou	1**	5/4**
MDOCKPP	1**	5/4**
S_Chang	1	5/1**
Kihara	1	3/2**
Venclovas	1	5/1**
HDOCK	1	3/2**
S_Huang	1	3/2**
Fernandez-Recio	1	5
Takeda-Shitaka	1	5
MULTICOM	1	2
Oliva	1	2

Table S2 – Prediction results for Target/Interface T221.3

**Predictors**	Top-1	Top-5
Wenyi_Zhang	1	5/3**
Zou	1	5
Shen	1	5
Venclovas	1	5
S_Chang	1	5
Pierce	1	5
Kozakov	1	5
Kihara	1	5
J_Cheng	1	5
Gray	1	5
Bates	1	5
Xuyang_Liu-Manifold	1	5
Xinqi_Gong-BeijingAIProtein	1	5
Wei_Zheng	1	5
Takeda-Shitaka	1	5
Y_Sun	1	5
Shan_Chang-CoDock	1	5
Qiwei_Ye-UltraFold	1	5
Ovchinnikov-ColabFold	1	5
Ness	1	5
McGuffin	1	5
KozakovVajda	1	5
Junlin_Wang-MUFold_H	1	5
Junhan_Chang-Manifold-X	1	5
Jianyi_Yang	1	5
GuijunLab	1	5
Fang_Bai	1	5
Elofsson	1	5
Baker	1	5
Zechen_Wang-Alchemy_LIG2	1	4
Xiao_Jia-Alchemy_LIG3	1	4
Liangzhen_Zheng-Alchemy_LIG	1	4
S_Huang	1	3
Wei_Lu	1	1
Ruihan_Guo	1	1

Table S2 – Prediction results for Target/Interface T221.3

Servers	Top-1	Top-5
MDOCKPP	1	5/2**
MULTICOM	1	5
LZERD	1	5
CLUSPRO	1	5
YANG-SERVER	1	5
ULTRAFOLD_SERVER	1	5
RAPTORX-MULTIMER	1	5
MULTICOM_QA	1	5
MULTICOM_DEEP	1	5
MUFOLD	1	5
MANIFOLD-LC-E	1	5
MANIFOLD-E	1	5
KIHARA_SERVER	1	5
GUIJUNLAB-DEEPDA	1	5
COLABFOLD	1	5
AF2-MULTIMER	1	5
MULTIFOLD	0	4
HDOCK	1	3
DFOLDING-SERVER	0	1
DFOLDING-REFINE	0	1

Scorers and Scoring Servers	Top-1	Top-5
Zou	1	5
MDOCKPP	1	5
Venclovas	1	5
Bonvin	1	5
S_Chang	1	5
Fernandez-Recio	1	5
Takeda-Shitaka	1	5
Kihara	1	3
HDOCK	1	3
S_Huang	1	3
MULTICOM	1	2
Oliva	1	2

Table S2 – Prediction results for Target/Interface T221.4

**Predictors**	Top-1	Top-5
Zhigang_Sun-GinobiFold	1***	5***
YiTing_Chen	1***	5***
Anqi_Pang-Coqualia	1***	5***
Baker	1***	4***
Venclovas	1***	5/3***/2**
Wenyi_Zhang	1**	4/2***/2**
Xuyang_Liu-Manifold	1***	4/2***/1**
Junhan_Chang-Manifold-X	1***	4/2***/1**
Shen	1**	5**
S_Chang	1**	5**
Kozakov	1**	5**
Kihara	1**	5**
Y_Sun	1**	4**
Shan_Chang-CoDock	1**	4**
KozakovVajda	1**	4**
Zechen_Wang-AIchemy_LIG2	1**	3**
Xiao_Jia-AIchemy_LIG3	1**	3**
Liangzhen_Zheng-AIchemy_LIG	1**	3**
Jie_Hou-FoldEver-Hybrid	1***	1***
Fang_Bai	1**	3**
Zou	1**	3/2**
S_Huang	1**	5/1**
Pierce	1	5/1**
Takeda-Shitaka	1	4/1**
Bates	1	5
Elofsson	1**	3/1**
McGuffin	1**	2/1**
J_Cheng	1	3
Gray	1**	1**
Wei_Zheng	1	3
Wei_Lu	1**	1**
Jianyi_Yang	1	2
Xinqi_Gong-BeijingAIProtein	1	1
Qiwei_Ye-UltraFold	1	1

Table S2 – Prediction results for Target/Interface T221.4

**Servers**	Top-1	Top-5
SHANGHAITECH-TS-SER	1***	5***
GINOBIFOLD	1***	5***
DFOLDING-SERVER	1***	4***
DFOLDING-REFINE	1***	4***
MANIFOLD-LC-E	1***	4/2***/1**
MANIFOLD-E	1***	4/2***/1**
CLUSPRO	1**	5**
FOLDEVER	1***	1***
HDOCK	1**	5/1**
MULTICOM_DEEP	0	3/1**
MUFOLD	1	3/1**
AF2-MULTIMER	1**	3/1**
MULTICOM	0	3
YANG-SERVER	1	2
MULTICOM_QA	0	2
ULTRAFOLD_SERVER	1	1
MULTIFOLD	1	1

**Scorers and Scoring Servers**	Top-1	Top-5
Venclovas	1***	5/2***/3**
Fernandez-Recio	1**	5**
Bonvin	1**	4/1***/1**
S_Chang	1**	5/4**
Takeda-Shitaka	1	5/4**
Zou	1**	4/3**
MDOCKPP	1	4/3**
Kihara	1**	3**
Oliva	1**	3/1**
HDOCK	1**	2/1**
S_Huang	1**	2/1**
Shen	1	2

Table S2 – Prediction results for Target/Interface T230.1

**Predictors**	Top-1	Top-5
Venclovas	1***	5***
Kozakov	1***	5***
Kihara	1***	5***
J_Cheng	1***	5***
Wei_Zheng	1***	5***
Wallner	1***	5***
Takeda-Shitaka	1***	5***
KozakovVajda	1***	5***
Elofsson	1***	5***
Baker	1***	5***
Zou	1***	5/4***/1**
Toshiyuki_Oda-PEZYFoldings	1***	4***
Jianyi_Yang	1***	4***
S_Huang	1***	3***
McGuffin	1**	5/1***/4**
Junlin_Wang-MUFold_H	1***	2***
Hongjiang_Miao-TRFold	1***	2***
DFolding	1***	2***
S_Chang	1**	5**
Shan_Chang-CoDock	1**	5**
Pierce	0	4**
Fernandez-Recio	1***	4/1***
Gray	1***	1***
Grudinin	1***	1***
Fang_Bai	1***	1***
David_Jones-DMP	1***	1***
Shen	0	2

**Servers**	Top-1	Top-5
MULTICOM	1***	5***
MDOCKPP	1***	5***
LZERD	1***	5***
CLUSPRO	1***	5***
YANG-SERVER	1***	5***
YANG-MULTIMER	1***	5***
MULTICOM_QA	1***	5***
MULTICOM_DEEP	1***	5***
DFOLDING-REFINE	1***	5***
MULTIFOLD	1***	5/4***/1**
AF2-MULTIMER	1***	4***
HDOCK	1***	3***
KIHARA_SERVER	0	3***
COLABFOLD	1***	3***
MUFOLD	0	2***
DFOLDING-SERVER	1***	2***
RAPTORX-MULTIMER	1**	3**
GUIJUNLAB-ASSEMBLY	0	1***

**Scorers and Scoring Servers**	Top-1	Top-5
Venclovas	1***	5***
Takeda-Shitaka	1***	5***
Kihara	1**	5/3***/2**
Zou	1**	5/3***/2**
Fernandez-Recio	1**	5/3***/2**
MDOCKPP	1**	5/2***/3**
S_Chang	1**	5/1***/4**
HDOCK	1**	5/1***/4**
S_Huang	1**	5/1***/4**
Bonvin	1**	5**
Oliva	1**	5**
Shen	1**	4**
MULTICOM	1**	4/3**
LZERD	1**	2**

Table S3 – Assessment Unit performance for AU203.1

Predictors	T203 1	2	3	average total
Fang_Bai	***	*	**	**
Zou	***	*	*	*
Xinqi_Gong-BeijingAIProtein	***	*	*	*
Wenyi_Zhang	**	*	**	*
Wei_Zheng	***	*	*	*
Wallner	***	*	*	*
Venclovas	***	*	*	*
Toshiyuki_Oda-PEZYFoldings	***	*	*	*
Takeda-Shitaka	***	*	*	*
S_Huang	***	*	*	*
Shan_Chang-CoDock	***	*	*	*
S_Chang	***	*	*	*
Qiwei_Ye-UltraFold	***	*	*	*
Pierce	***	*	*	*
J_Cheng	***	*	*	*
Hongjiang_Miao-TRFold	***	*	*	*
Gray	***	*	*	*
Fernandez-Recio	***	*	*	*
Elofsson	***	*	*	*
David_Jones-DMP	***	*	*	*
Baker	***	*	*	*
AIlab-trComplex	***	*	*	*
McGuffin	***	0	*	*
KozakovVajda	**	*	*	*
Kozakov	**	*	*	*
Kihara	***	*	0	*
Jianyi_Yang	***	0	*	*
Zhigang_Sun-GinobiFold	***	0	0	*
Zechen_Wang-Alchemy_LIG2	***	0	0	*
Y_Sun	***	0	0	*
Xuyang_Liu-Manifold	***	0	0	*
Xiao_Jia-Alchemy_LIG3	***	0	0	*
Xianming_Pan	***	0	0	*
Suwen_Zhao	***	0	0	*
Sieradzan	**	*	0	*
Shen	***	0	0	*
Ovchinnikov-ColabFold	***	0	0	*
Liangzhen_Zheng-Alchemy_LIG	***	0	0	*
Junlin_Wang-MUFold_H	***	0	0	*
Jimenez-Garcia	***	0	0	*
Gwang_So-FTBiot0119	***	0	0	*
GuijunLab	***	0	0	*
Grudinin	***	0	0	*
Bates	***	0	0	*
Anqi_Pang-Coqualia	***	0	0	*
Kazuki_Yamamoto-ddquest	0	*	*	0

Table S3 – Assessment Unit performance for AU203.1

Servers	T203 1	2	3	average total
ULTRAFOLD_SERVER	***	*	*	*
MULTICOM_QA	***	*	*	*
MULTICOM_DEEP	***	*	*	*
MULTICOM	***	*	*	*
MDOCKPP	***	*	*	*
HDOCK	***	*	*	*
DFOLDING-REFINE	***	*	*	*
YANG-SERVER	***	0	*	*
YANG-MULTIMER	***	0	*	*
CLUSPRO	**	*	*	*
SHANGHAITECH-TS-SER	***	0	0	*
MULTIFOLD	***	0	0	*
MUFOLD	***	0	0	*
MANIFOLD-E	***	0	0	*
LZERD	***	0	0	*
KIHARA_SERVER	***	0	0	*
GUIJUNLAB-DEEPDA	***	0	0	*
GUIJUNLAB-ASSEMBLY	***	0	0	*
GINOBIFOLD	***	0	0	*
COLABFOLD	***	0	0	*
AF2-MULTIMER	***	0	0	*
FOLDEVER	0	*	0	0

Scorers and Scoring Servers	T203 1	2	3	average total
Zou	***	*	*	*
Venclovas	***	*	*	*
Takeda-Shitaka	***	*	*	*
S_Huang	***	*	*	*
S_Chang	***	*	*	*
Oliva	***	*	*	*
MULTICOM	***	*	*	*
MDOCKPP	***	*	*	*
LZERD	***	*	*	*
Kihara	***	*	*	*
HDOCK	***	*	*	*
Fernandez-Recio	***	*	*	*
Bonvin	***	*	*	*
Shen	***	*	0	*
J_Cheng	***	0	0	*

Table S3 – Assessment Unit performance for AU204.2

Please see Table S2 – Prediction results for Target/Interface T203.4

Table S3 – Assessment Unit performance for AU204.1

Predictors	T204 1	2	best total
Wei_Zheng	**	**	**
Xuyang_Liu-Manifold	**	*	**
Kozakov	**	*	**
Venclovas	*	*	*
Shan_Chang-CoDock	*	*	*
Schneidman	*	*	*
S_Chang	*	*	*
Kihara	*	*	*
Jianyi_Yang	*	*	*
Gwang_So-FTBiot0119	*	*	*
Toshiyuki_Oda-PEZYFoldings	*	0	*
S_Huang	*	0	*
DFolding	*	0	*
Baker	*	0	*

Servers	T204 1	2	best total
CLUSPRO	**	*	**
YANG-MULTIMER	*	*	*
MANIFOLD-E	**	0	**
LZERD	*	*	*
KIHARA_SERVER	*	*	*
HDOCK	*	0	*
DFOLDING-SERVER	*	0	*
DFOLDING-REFINE	*	0	*

Scorers and Scoring Servers	T204 1	2	best total
S_Chang	**	*	**
J_Cheng	**	*	**
Zou	*	*	*
Venclovas	*	*	*
Takeda-Shitaka	*	*	*
S_Huang	*	*	*
Oliva	*	*	*
MDOCKPP	*	*	*
LZERD	*	*	*
Kihara	*	*	*
HDOCK	*	*	*
Fernandez-Recio	*	*	*
Bonvin	*	*	*
MULTICOM	*	0	*

Table S3 – Assessment Unit performance for AU204.2

Predictors	T204<br>3	4	5	6	7	8	average<br>total
J_Cheng	***	***	***	**	***	***	**
S_Chang	***	***	***	**	***	**	**
Venclovas	**	**	***	**	***	***	**
Wei_Zheng	***	***	***	**	***	0	**
DFolding	***	***	***	**	***	0	**
Shan_Chang-CoDock	***	***	***	**	**	0	**
Kihara	**	**	***	*	**	***	**
Xuyang_Liu-Manifold	**	***	**	**	***	0	**
Baker	**	***	***	**	**	0	**
Takeda-Shitaka	***	***	0	**	0	***	*
Schneidman	***	***	***	**	0	0	*
Pierce	**	0	***	*	**	***	*
Wallner	**	**	***	*	**	0	*
Jianyi_Yang	**	***	***	**	0	0	*
S_Huang	***	***		*	0	***	*
GuijunLab	**	**	***	0	**	0	*
Grudinin	**	**	***	0	**	0	*
Fang_Bai	***	***	***	0	0	0	*
Elofsson	**	**	**	0	***	0	*
Junlin_Wang-MUFold_H	**	***	***	0	0	0	*
Y_Sun	**	0	*	**	0	0	0
McGuffin	**	***	0	0	**	0	*
Kozakov	**	0	0	0	**	***	*
Toshiyuki_Oda-PEZYFoldings	**	**	**	0	0	0	*
Suwen_Zhao	**	**	**	0	0	0	*
Sieradzan	**	*	0	0	**	*	*
Shen	**	0	*	**	**	0	*
Zhigang_Sun-GinobiFold	**	**	0	0	0	0	0
Ruihan_Guo	0	**	**	0	0	0	0
Bates	*	0	0	0	*	**	0
Anqi_Pang-Coqualia	**	**	0	0	0	0	0
Gwang_So-FTBiot0119	0	0	0	0	0	***	0
Jie_Hou-FoldEver-Hybrid	0	**	0	0	0	0	0
Sachin_Kadyan-OpenFold-SingleSeq	*	0	0	0	0	0	0
Sachin_Kadyan-OpenFold	*	0	0	0	0	0	0
Qiwei_Ye-UltraFold	*	0	0	0	0	0	0
David_Jones-DMP	0	0	0	*	0	0	0

Table S3 – Assessment Unit performance for AU204.2

Servers	T204 3	4	5	6	7	8	average total
MULTICOM	***	***	***	**	***	***	**
DFOLDING-SERVER	***	***	***	**	***	0	**
DFOLDING-REFINE	***	***	***	**	***	0	**
MULTICOM_DEEP	**	***	***	**	***	0	**
MULTICOM_QA	**	***	***	*	***	0	**
YANG-MULTIMER	**	***	***	**	0	0	*
HDOCK	***	***		*	0	***	*
GUIJUNLAB-DEEPDA	**	**	***	0	**	0	*
AF2-MULTIMER	**	**	***	0	**	0	*
MUFOLD	**	***	***	0	0	0	*
MULTIFOLD	**	***	0	0	**	0	*
CLUSPRO	**	0	0	0	**	***	*
LZERD	**	0	0	0	***	0	0
KIHARA_SERVER	**	0	0	0	***	0	0
FOLDEVER	0	**	0	0	0	0	0
ULTRAFOLD_SERVER	*	0	0	0	0	0	0
GUIJUNLAB-ASSEMBLY	0	0	0	0	*	0	0

Scorers and Scoring Servers	T204 3	4	5	6	7	8	average total
S_Chang	***	***	***	**	***	***	**
LZERD	***	***	***	**	***	***	**
Kihara	***	***	***	**	***	***	**
Bonvin	***	***	***	**	***	**	**
Venclovas	***	***	***	**	***	0	**
Oliva	***	***	**	**	**	**	**
J_Cheng	**	**	***	*	***	***	**
Zou	***	***	***	**	**	0	**
Takeda-Shitaka	**	**	***	*	**	***	**
S_Huang	***	***	***	**	**	0	**
MDOCKPP	***	***	***	**	**	0	**
HDOCK	***	***	***	**	**	0	**
Fernandez-Recio	***	***	***	**	**	0	**
Shen	**	***	***	0	***	0	*
MULTICOM	***	***		*	0	0	*

Table S3 – Assessment Unit performance for AU219

	T219			T220			T221			best
**Predictors**	1	2	3	1	2	3	1	2	3	total
Zou	**	**	*	**	**	**	**	**	*	**
S_Huang	**	**	*	**	**	**	**	**	*	**
KozakovVajda	**	**	*	**	**	**	**	**	*	**
Kozakov	**	**	*	**	**	**	**	**	*	**
Kihara	**	**	*	**	**	**	**	**	*	**
Xuyang_Liu-Manifold	**	**	*	**	**	**	**	*	*	**
Wei_Zheng	**	**	*	**	**	**	**	*	*	**
Takeda-Shitaka	**	**	*	**	**	**	**	*	*	**
Shan_Chang-CoDock	**	**	*	**	**	**	**	*	*	**
S_Chang	**	**	*	**	**	**	**	*	*	**
Pierce	**	**	*	**	**	**	**	*	*	**
Ovchinnikov-ColabFold	**	**	*	**	**	**	**	*	*	**
Junlin_Wang-MUFold_H	**	**	*	**	**	**	**	*	*	**
Junhan_Chang-Manifold-X	**	**	*	**	**	**	**	*	*	**
Jianyi_Yang	**	**	*	**	**	**	**	*	*	**
J_Cheng	**	**	*	**	**	**	**	*	*	**
Fang_Bai	**	**	*	**	**	**	**	*	*	**
Elofsson	**	**	*	**	**	**	**	*	*	**
Y_Sun	*	**	*	*	**	*	**	**	*	**
Xinqi_Gong-BeijingAIProtein	**	**	*	**	*	**	**	*	*	**
Venclovas	**	**	*	**	**	**	*	*	*	**
Qiwei_Ye-UltraFold	**	**	*	**	*	**	**	*	*	**
Ness	**	**	0	**	**	**	**	*	*	**
McGuffin	**	**	0	**	**	**	**	*	*	**
Gray	**	**	*	**	*	**	**	*	*	**
Shen	*	**	*	*	**	**	**	**	*	**
Bates	**	*	*	**	*	**	**	*	*	**
Baker	*	**	0	**	**	**	**	*	*	**
Wenyi_Zhang	0	0	0	**	**	**	**	*	**	**
Ruihan_Guo	0	0	0	**	**	**	**	**	*	**
MyongHo_Chae	**	**	*	**	**	**	0	0	0	**
GuijunLab	**	**	*	*	*	*	*	*	*	**
Zechen_Wang-AIchemy_LIG2	0	0	0	**	**	**	**	*	*	**
Xiao_Jia-AIchemy_LIG3	0	0	0	**	**	**	**	*	*	**
Wei_Lu	0	0	0	**	**	**	**	*	*	**
Liangzhen_Zheng-AIchemy_LIG	0	0	0	**	**	**	**	*	*	**
Ziheng_Zou	0	0	0	**	**	**	0	0	0	**
Wallner	**	**	*	0	0	0	0	0	0	**
Toshiyuki_Oda-PEZYFoldings	**	**	*	0	0	0	0	0	0	**
Kazuki_Yamamoto-ddquest	**	**	*	0	0	0	0	0	0	**
Grudinin	**	**	*	0	0	0	0	0	0	**
Hongjiang_Miao-TRFold	*	**	*	0	0	0	0	0	0	**
AIlab-trComplex	*	**	*	0	0	0	0	0	0	**
Sieradzan	0	*	0	0	0	0	0	0	0	*

Table S3 – Assessment Unit performance for AU219

	T219			T220			T221			best
**Servers**	1	2	3	1	2	3	1	2	3	total
MDOCKPP	**	**	*	**	**	**	**	**	**	**
MULTIFOLD	**	**	*	**	**	**	**	**	*	**
KIHARA_SERVER	**	**	*	**	**	**	**	**	*	**
HDOCK	**	**	*	**	**	**	**	**	*	**
CLUSPRO	**	**	*	**	**	**	**	**	*	**
YANG-SERVER	**	**	*	**	**	**	**	*	*	**
RAPTORX-MULTIMER	**	**	*	**	**	**	**	*	*	**
MULTICOM_QA	**	**	*	**	**	**	**	*	*	**
MULTICOM_DEEP	**	**	*	**	**	**	**	*	*	**
MULTICOM	**	**	*	**	**	**	**	*	*	**
MUFOLD	**	**	*	**	**	**	**	*	*	**
MANIFOLD-LC-E	**	**	*	**	**	**	**	*	*	**
MANIFOLD-E	**	**	*	**	**	**	**	*	*	**
COLABFOLD	**	**	*	**	**	**	**	*	*	**
AF2-MULTIMER	**	**	*	**	**	**	**	*	*	**
ULTRAFOLD_SERVER	**	**	*	**	*	**	**	*	*	**
DFOLDING-SERVER	**	**	*	**	0	**	**	**	*	**
DFOLDING-REFINE	**	**	*	**	0	**	**	**	*	**
LZERD	0	0	0	**	**	**	**	**	*	**
GUIJUNLAB-DEEPDA	**	**	*	*	*	*	*	*	*	**
GUIJUNLAB-ASSEMBLY	**	**	*	*	*	*	0	0	0	**
YANG-MULTIMER	**	**	*	0	0	0	0	0	0	**

	T219			T220			T221			best
**Scorers and Scoring Servers**	1	2	3	1	2	3	1	2	3	total
Zou	**	**	*	**	**	**	**	**	**	**
MDOCKPP	**	**	*	**	**	**	**	**	**	**
S_Huang	**	**	*	**	**	**	**	**	*	**
S_Chang	**	**	*	**	**	**	**	**	*	**
Kihara	**	**	*	**	**	**	**	**	*	**
HDOCK	**	**	*	**	**	**	**	**	*	**
Bonvin	**	**	*	**	**	**	**	**	*	**
Takeda-Shitaka	**	**	*	**	**	**	**	*	*	**
MULTICOM	**	**	*	**	**	**	**	*	*	**
Fernandez-Recio	**	**	*	**	**	**	**	*	*	**
Venclovas	**	**	*	*	*	**	**	**	*	**
Oliva	**	**	*	**	**	**	*	*	*	**
LZERD	0	0	*	**	**	**	**	**	*	**
J_Cheng	0	0	0	**	**	**	0	0	0	**

Table S3 – Assessment Unit performance for AU220

Predictors	T2204	T2214	best total
Zhigang_Sun-GinobiFold	***	***	***
YiTing_Chen	***	***	***
Wenyi_Zhang	***	***	***
Venclovas	***	***	***
Jie_Hou-FoldEver-Hybrid	***	***	***
Baker	***	***	***
Anqi_Pang-Coqualia	***	***	***
Xuyang_Liu-Manifold	**	***	***
Junhan_Chang-Manifold-X	**	***	***
Zou	**	**	**
Y_Sun	**	**	**
Takeda-Shitaka	**	**	**
Shen	**	**	**
Shan_Chang-CoDock	**	**	**
S_Chang	**	**	**
KozakovVajda	**	**	**
Kozakov	**	**	**
Kihara	**	**	**
J_Cheng	***	*	***
Gray	**	**	**
Fang_Bai	**	**	**
Zechen_Wang-AIchemy_LIG2	*	**	**
Xiao_Jia-AIchemy_LIG3	*	**	**
Wei_Lu	*	**	**
S_Huang	*	**	**
Pierce	*	**	**
McGuffin	*	**	**
Liangzhen_Zheng-AIchemy_LIG	*	**	**
Elofsson	*	**	**
Xinqi_Gong-BeijingAIProtein	*	*	*
Wei_Zheng	*	*	*
Qiwei_Ye-UltraFold	*	*	*
Jianyi_Yang	*	*	*
Bates	*	*	*
Ziheng_Zou	*	0	*
Junlin_Wang-MUFold_H	*	0	*
GuijunLab	*	0	*

Table S3 – Assessment Unit performance for AU220

Servers	T220<br>4	T221<br>4	best<br>total
SHANGHAITECH-TS-SER	***	***	***
GINOBIFOLD	***	***	***
FOLDEVER	***	***	***
DFOLDING-SERVER	***	***	***
DFOLDING-REFINE	***	***	***
MULTICOM	***	**	***
MANIFOLD-LC-E	**	***	***
MANIFOLD-E	**	***	***
CLUSPRO	**	**	**
MULTICOM_DEEP	*	**	**
MUFOLD	*	**	**
HDOCK	*	**	**
AF2-MULTIMER	*	**	**
YANG-SERVER	*	*	*
ULTRAFOLD_SERVER	*	*	*
MULTICOM_QA	*	*	*
MULTIFOLD	0	*	*

Scorers and Scoring Servers	T220<br>4	T221<br>4	best<br>total
Venclovas	**	***	***
Fernandez-Recio	***	**	***
Bonvin	**	***	***
Zou	**	**	**
S_Chang	**	**	**
MDOCKPP	**	**	**
Kihara	**	**	**
Takeda-Shitaka	*	**	**
Oliva	*	**	**
Shen	*	*	*
S_Huang	0	**	**
HDOCK	0	**	**
LZERD	*	0	*

Table S4 – Complete participant performance

						Dockq			
Rank	**Predictors**	Participation	Top-1	Top-5	Score	Sum	Rank	ΣZ^+	Rank
1	Venclovas	38	26/12***/10**	29/14***/11**	68	22.94	1	52.80	3
	Wallner	35	25/16***/6**	28/17***/6**	68	21.76	4	57.82	1
3	Wei_Zheng	38	26/11***/11**	28/13***/11**	65	22.21	2	43.89	4
	Toshiyuki_Oda-PEZYFoldings	37	21/11***/8**	29/13***/10**	65	21.83	3	55.93	2
5	Jianyi_Yang	36	27/12***/8**	27/13***/8**	61	20.63	5	38.17	9
6	S_Chang	38	23/6***/9**	27/8***/15**	58	20.55	6	40.59	6
7	Kihara	38	25/6***/12**	28/8***/12**	56	19.21	9	41.13	5
	J_Cheng	38	22/8***/10**	24/12***/8**	56	19.93	8	29.77	12
	Shan_Chang-CoDock	37	22/6***/8**	26/8***/14**	56	20.09	7	39.74	8
10	Pierce	38	22/7***/8**	27/9***/10**	55	18.93	11	32.56	11
11	S_Huang	38	23/8***/9**	26/8***/12**	54	19.07	10	40.52	7
12	Xuyang_Liu-Manifold	38	18/9***/5**	24/10***/9**	53	18.90	12	33.25	10
	Junlin_Wang-MUFold_H	38	20/9***/9**	24/11***/7**	53	17.61	19	18.84	25
14	Kozakov	38	22/7***/10**	24/8***/11**	51	17.77	17	26.70	14
	Elofsson	38	22/9***/9**	23/10***/8**	51	18.26	14	19.95	22
16	Takeda-Shitaka	38	19/9***/7**	22/10***/8**	50	18.62	13	22.32	17
	Baker	38	22/5***/11**	25/7***/11**	50	18.12	15	27.64	13
18	McGuffin	38	19/6***/10**	22/9***/9**	49	17.99	16	19.79	23
19	Zou	38	20/6***/10**	23/8***/9**	48	16.92	21	20.02	21
	Xinqi_Gong-BeijingAIProtein	36	20/4***/10**	22/9***/8**	48	17.12	20	25.32	15
	Qiwei_Ye-UltraFold	38	21/5***/10**	22/9***/8**	48	17.75	18	25.31	16
22	Ovchinnikov-ColabFold	33	17/8***/8**	19/10***/6**	45	14.56	29	18.98	24
23	Hongjiang_Miao-TRFold	36	14/6***/5**	20/8***/8**	44	16.62	22	20.80	18
24	Shen	38	18/3***/9**	23/5***/9**	42	15.40	26	18.27	27
	Fang_Bai	38	17/2***/11**	24/3***/12**	42	15.58	25	20.68	19
26	Grudinin	35	16/7***/6**	20/8***/5**	41	15.93	23	18.04	28
	AIlab-trComplex	36	12/4***/6**	19/7***/8**	41	15.63	24	18.64	26
28	Y_Sun	36	16/3***/8**	20/5***/9**	39	14.84	27	17.81	30
	KozakovVajda	26	16/5***/8**	18/6***/9**	39	13.14	31	20.66	20
	GuijunLab	36	14/5***/4**	18/8***/5**	39	14.82	28	14.38	34
31	Gray	33	16/5***/9**	17/5***/10**	37	13.32	30	11.01	37
32	Sieradzan	36	11/4***/4**	18/5***/4**	32	12.04	35	8.57	43
	Fernandez-Recio	35	12/6***/4**	16/6***/4**	32	11.61	36	11.18	36
34	David_Jones-DMP	28	13/6***/5**	13/6***/5**	30	12.43	34	14.90	32
35	Bates	38	12/2***/4**	14/4***/5**	27	10.50	38	8.58	42
36	Suwen_Zhao	20	8/2***/4**	11/5***/3**	24	9.23	39	17.97	29
	Anqi_Pang-Coqualia	20	9/5***/4**	10/5***/4**	24	8.65	41	10.04	39
38	DelCarpio	33	9/3***/3**	11/3***/6**	23	9.04	40	10.30	38
39	Zhigang_Sun-GinobiFold	20	7/4***/3**	9/5***/3**	22	8.32	42	9.75	41
40	Jie_Hou-FoldEver-Hybrid	32	9/3***/4**	9/4***/4**	21	12.74	33	9.98	40
41	DFolding	13	8/2***/4**	10/2***/5**	19	7.22	43	11.19	35
42	Negi	6	6/4***/2**	6/5***/1**	17	5.25	46	4.08	48
	Wenyi_Zhang	17	9/6**	9/1***/6**	17	7.04	44	7.08	44
44	Ruihan_Guo	17	6/2***/4**	6/2***/4**	14	5.39	45	4.49	47
45	Junhan_Chang-Manifold-X	6	5/3***/1**	5/3***/1**	12	3.61	50	4.03	49
46	YiTing_Chen	9	3/2***/1**	5/2***/2**	11	3.77	49	5.66	46
	Sachin_Kadyan-OpenFold-SingleSeq	24	2***	5/2***/2**	11	4.52	47	3.75	50
	Sachin_Kadyan-OpenFold	24	2***	5/2***/2**	11	4.52	48	3.75	51
49	Zechen_Wang-AIchemy_LIG2	7	3/1***/2**	4/1***/2**	8	3.15	51	1.72	54
	Xiao_Jia-AIchemy_LIG3	7	3/1***/2**	4/1***/2**	8	3.15	52	1.72	55
	Liangzhen_Zheng-AIchemy_LIG	7	4/1***/2**	4/1***/2**	8	3.15	53	1.72	56
	Kazuki_Yamamoto-ddquest	4	4/1***/2**	4/1***/2**	8	2.42	55	2.66	53
53	Ness	4	3/1***/2**	3/1***/2**	7	2.29	56	2.89	52
54	Gwang_So-FTBiot0119	38	2/1***	4/1***	6	13.14	32	14.74	33
55	Wei_Lu	2	2**	2**	4	1.05	59	0.57	59
56	Ziheng_Zou	2	2/1**	2/1**	3	0.98	60	0.36	60
57	Schneidman	6	2	2	2	2.18	57	6.69	45
	MyongHo_Chae	34	1**	1**	2	11.29	37	15.24	31
59	Jimenez-Garcia	5	1	1	1	0.61	63	0.00	64
	Xianming_Pan	27	1	1	1	2.68	54	0.00	61
61	L_Yang	16	0	0	0	1.50	58	0.00	63
	Rives	13	0	0	0	0.65	62	1.57	58
	Qi_Wu	1	0	0	0	0.01	65	0.00	62
	Grudinin-KORP-PL	1	0	0	0	0.25	64	0.00	65
	Gong	7	0	0	0	0.66	61	1.60	57

Table S4 – Complete participant performance

						Dockq			
Rank	**Servers**	Participation	Top-1	Top-5	Score	Sum	Rank	ΣZ⁺	Rank
1	YANG-MULTIMER	37	26/10***/9**	27/12***/8**	59	20.51	1	34.40	3
2	MULTICOM	38	21/7***/10**	24/12***/9**	57	19.91	2	30.64	4
3	MULTICOM_QA	38	21/8***/10**	24/11***/9**	55	19.50	4	26.10	9
	MULTICOM_DEEP	38	20/7***/10**	24/10***/11**	55	19.70	3	28.10	6
	AF2-MULTIMER	38	22/10***/10**	24/11***/9**	55	17.92	7	18.67	15
6	HDOCK	38	23/8***/9**	26/8***/12**	54	19.07	5	40.52	1
7	CLUSPRO	38	22/7***/10**	24/8***/11**	51	17.77	9	26.70	8
	MANIFOLD-E	37	20/9***/6**	23/10***/8**	51	18.66	6	30.35	5
	DFOLDING-SERVER	31	21/8***/11**	23/9***/10**	51	17.45	11	35.04	2
10	MUFOLD	38	17/8***/6**	22/10***/8**	50	17.36	12	16.29	18
11	ULTRAFOLD_SERVER	38	21/5***/10**	22/9***/8**	48	17.71	10	25.38	10
	MULTIFOLD	38	20/8***/9**	22/9***/8**	48	16.66	13	18.75	14
	DFOLDING-REFINE	35	21/2***/15**	25/3***/17**	48	17.83	8	26.84	7
	COLABFOLD	34	17/8***/8**	20/11***/6**	48	15.45	18	19.68	12
15	KIHARA_SERVER	37	17/4***/8**	22/6***/10**	44	15.76	17	21.47	11
16	GUIJUNLAB-ASSEMBLY	38	13/7***/5**	18/10***/5**	43	15.90	16	17.54	17
17	GUIJUNLAB-DEEPDA	38	14/4***/8**	18/9***/5**	41	15.94	15	14.25	19
18	LZERD	33	16/3***/9**	20/4***/10**	38	14.01	19	19.61	13
19	MDOCKPP	38	18/3***/10**	18/5***/8**	36	13.76	20	14.18	20
	RAPTORX-MULTIMER	37	13/5***/8**	15/6***/9**	36	16.28	14	17.73	16
21	YANG-SERVER	17	14/4***/5**	14/6***/3**	29	9.89	22	13.66	21
22	FOLDEVER	25	10/3***/5**	10/5***/4**	24	9.91	21	9.35	24
23	SHANGHAITECH-TS-SER	17	8/4***/4**	10/5***/3**	23	6.96	24	9.87	23
24	GINOBIFOLD	17	7/4***/3**	9/5***/3**	22	7.16	23	10.04	22
25	MANIFOLD-LC-E	5	4/2***/1**	4/2***/1**	9	2.80	25	2.78	26
26	XRC_VU	11	2/1**	2/1***/1**	5	2.14	26	1.67	27
27	TS317	1	1***	1***	3	0.70	28	4.44	25
	GUIJUNLAB-META	1	1**	1***	3	0.85	27	1.12	29
29	FALCON2	11	0	0	0	0.11	30	0.00	30
	FALCON0	11	0	0	0	0.11	31	0.00	31
	CEREBRA	8	0	0	0	0.69	29	1.60	28

						Dockq			
Rank	**Scorers and Scoring Servers**	Participation	Top-1	Top-5	Score	Sum	Rank	ΣZ⁺	Rank
1	Venclovas	37	24/8***/12**	26/11***/11**	59	19.99	1	45.18	1
2	S_Huang	37	18/3***/12**	26/9***/11**	55	19.52	2	40.08	2
3	Kihara	37	22/3***/12**	25/9***/11**	54	19.23	3	37.48	5
4	HDOCK	37	17/3***/11**	26/8***/11**	53	19.14	4	39.49	4
5	S_Chang	37	22/5***/10**	25/7***/12**	51	18.59	6	39.90	3
6	Zou	37	19/3***/13**	24/8***/10**	50	18.54	7	35.69	6
7	Oliva	37	20/3***/12**	26/5***/13**	49	18.75	5	32.63	7
	Fernandez-Recio	37	20/4***/10**	23/8***/10**	49	17.30	10	22.46	12
9	MDOCKPP	37	17/1***/12**	24/7***/10**	48	17.35	9	32.27	8
	Bonvin	37	15/3***/8**	23/7***/11**	48	17.47	8	29.15	9
11	Takeda-Shitaka	37	20/4***/10**	21/6***/10**	43	15.44	11	22.63	11
12	LZERD	37	15/2***/6**	20/5***/9**	39	15.17	12	25.85	10
13	MULTICOM	37	17/3***/6**	18/4***/7**	33	13.69	13	17.73	13
14	Shen	36	6/1***/4**	16/3***/6**	28	12.80	14	13.22	14
15	J_Cheng	37	4/2**	9/2***/4**	17	7.25	15	5.98	15

CAPRI ID	CASP ID	AU	Stoichiometry	Interfaces	Kingdom	Organism	PDB	Source and Resolution	UniProt ID	Protein name	Residues	BSA	AF (PDB) rms	Best top-1 DockQ	Best DockQ Avg +StdDev	#groups	Top-1 performance	Top-5 performance	#scorer groups	S Top-1 performance	S Top-5 performance	S Top-10 performance	# scorer models	scorer quality content	# uploaded models	uploader quality content
	**Targets with one interface (A2) and no intertwining**																									
T198	T1123	T198	A2	1	Viruses	Astrovirus M	7UZT	X; 1.86	B6UYJ1	Capsid polyprote	266	1570	6.48	**	0.285 +0.206	60	21/14**	30/19**	15	5/4**	6/5**	7/6**	135	29/22**	1110	36/11**
T201	T1132	T201	A2	1	Bacteria	Pseudomon		X; 2.00	A0A072ZNL3	Antibiotic biosynt	102	1125	1.12 (2OMO)	***	0.940 +0.072	58	42/36***/4**	54/52***/1**	15	9/4***/4**	14/11***/2**	15/13***	110	100/65***/28**	992	538/417***/109**
T211	T1153	T211	A2	1	Eukaryotes	Homo sapie		X; 2.00	Q7L9B9	Endonuclease/ex	299	550	0.38	***	0.666 +0.285	58	39/12***/27**	48/25***/21**	15	3**	9/2***/5**	9/3***/5**	135	22/3***/14**	1111	138/30***/61**
T225	T1178	T225	A2	1	Viruses	Human astr		X; 2.73	unannotated		306	3715	0.78	***	0.611 +0.179	66	51/9***/40**	57/9***/45**	15	12/1***/10**	13/4***/9**	14/4***/10**	130	107/5***/97**	1106	330/2***/249**
T226	T1179	T226	A2	1	Viruses	Murine astr		X; 1.75	unannotated		261	1830	1.08	***	0.377 +0.205	66	29/2***/5**	48/2***/6**	15	6	12	12/3**	132	40/3**	1070	133/1**
T229	T1187	T229	A2	1	Eukaryotes	Nicotiana		X; 2.0	Q94EW1	Nictaba	166	935	0.57	***	0.252 +0.356	71	7/3***/4**	16/14***/1**	15	0	1	1	135	1	1151	57/1***/2**
	**Targets with one interface (A2/A3) and intertwining**																									
T192	T1109	T192	A2	1	Bacteria	Ralstonia sc		X; 1.00	Q8XYF6:D180A	Putative transcrip	227	2100	1.02 (3MGK)	***	0.758 +0.174	60	53/38***/14**	54/45***/9**	15	14/12***/2**	15/13***/2**	15/13***/2**	135	114/70***/37**	1009	544/160***/274**
T193	T1110	T193	A2	1	Bacteria	Ralstonia sc		X; 0.74	Q8XYF6	Putative transcrip	227	2265	1.36 (3MGK)	***	0.919 +0.066	60	56/51***/4**	56/53***/3**	15	14/9***/3**	15***	15***	135	118/90***/23**	1007	572/307***/243**
T194	T1113	T194	A2	1	Viruses	Pseudomon		X; 2.63	A0A4D6BFJ2	Uncharacterized	193	2750	8.9	**	0.692 +0.110	62	55/46**	57/1***/54**	15	13/12**	13**	13**	135	111/90**	995	243/274**
T197	T1121	T197	A2	1	Bacteria	Pseudomon	7TIL	EM; 3.7	A0A0H2ZM47	DUF3322 and DU	381	1420	11.79	*	0.197 +0.092	58	3	7	15	0	1	3	135	9	1040	39
T199	T1127	T199	A2	1	Eukaryotes	Arabidopsis		X; 1.35	Q9ZV05	L-ornithing N5-ac	211	3355	1.08 (2FE7)	***	0.882 +0.069	69	65/54***/9**	65/57***/8**	15	13/7***/4**	14/12***/2**	15/13***/2**	135	106/34***/49**	1088	410/152***/198**
T213	T1160	T213	A2	1				X; 1.35	designed		48	1080	Not available	0	0.199 +0.121	64	0	3**	15	0	0	0	135	0	1023	2**
T214	T1161	T214	A2	1				X; 1.30	designed		48	1845	Not available	**	0.340 +0.176	64	2/1**	10/4***/3**	15	2/1**	5/1***/3**	6/1***/4**	135	10/1***/6**	1024	25/2***/4**
T222	T1173	T222	A3	1	Bacteria	Bdellovibrio		X; 2.40	Q6MNC5	Cell wall surface	204	2015	3.49	***	0.603 +0.244	71	42/17***/19**	56/21***/25**	15	9/1***/3**	13/6***/6**	13/7***/5**	132	93/22***/49**	1028	162/10***/63**
T223	T1174	T223	A3	1	Bacteria	Bdellovibrio		X; 2.50	Q6ML84	Uncharacterized	338	5715	2.55	**	0.425 +0.096	69	30/2**	48/2**	15	4	11	11	132	34	786	50
T224	T1176	T224	A2	1	Bacteria	Clostridioide		X; 2.00	unannotated	Hypothetical prot	170	5700	0.81	0	0.029 +0.021	63	0	0	15	0	0	0	133	0	969	0
T227	T1181	T227	A3	1	Viruses	Gamaleyavi		X; 2.30	G0XNW6	Tail fiber protein	688	4940	1.14	***	0.476 + 0.223	70	45/2***/29**	53/3***/31**	15	9**	13/11**	14/1***/11**	126	80/1***/70**	967	159/1***/83**
	**Hetero-targets with one interface (A1B1)**																									
T191	H1106	T191	AB	1	Bacteria	Yersinia ent	7QII	X; 1.92	P0C2N4, P61417	Yop proteins tran	122, 114	1440	8.74, 0.56	***	0.735 +0.227	79	68/34***/34**	70/48***/21**	No scoring						984	359/94***/238**
T200	H1129	T200	AB	1	Viruses	Escherichia	8A8C	EM; 3.1	P06971, P23207	Ferrichrome oute	747, 640	2040	0.35 (4CU4), 1.83	***	0.184 +0.171	71	10/4***	20/5***	14	4/1***/1**	10/7***	12/8***/1**	130	33/8***/3**	1189	81/1***/1**
T202	H1134	T202	AB	1	Bacteria	Enterobacte	7UBZ	X; 1.75	A0A0H3CKN4, A0A0M7ENE2	Ankyrin repeat do	230, 313	2000	3.93 (6MOK), 5.37	***	0.608 +0.246	76	60/11***/46**	63/22***/39**	15	12/1***/1**	14/5***/9**	14/7***/7**	135	103/8***/90**	1059	307/4***/265**
T210	H1151	T210	AB	1	Bacteria	Mycobacteri		X; 1.80	P9WGI1, P9WF37	RNA polymerase	112, 116	740	1.18 (7KUG)	***	0.816 +0.235	77	66/57***/6**	68/63***/5**	15	11/4***/6**	12/8***/4**	14/9***/4**	135	84/30***/41**	1105	394/212***/112**
T212	H1157	T212	AB	1	Eukaryotes	Thermochae		EM; 3.3+	G0SCX7, G0SGS2	Alpha-1,2-mannc	1029, 495	2250	0.89, 7.93	**	0.562 +0.157	73	59/53**	63/61**	15	12**	14**	14**	135	100/78**	1089	413/306**
	**Targets with one interface (A1B1 or A:HL): nanobodies and antibodies**																									
T205	H1140	T205	AB	1	Eukaryotes	Mus muscul		X; 2.75	P16330	2',3'-cyclic-nucle	219, 132	775	0.54 (5AE0)	**	0.093 +0.137	80	6/2**	7/2**	15	0	1**	1**	135	1**	1379	24/4**
T206	H1141	T206	AB	1	Eukaryotes	Mus muscul		X; 2.50	P16330	2',3'-cyclic-nucle	219, 127	925	0.54 (5AE0)	***	0.175 +0.242	78	8/3***/5**	12/4***/7**	15	1***	1***	1***	135	2/1***	1332	27/1***/12**
T207	H1142	T207	AB	1	Eukaryotes	Mus muscul		X; 1.73	P16330	2',3'-cyclic-nucle	219, 128	585	0.54 (5AE0)	0	0.035 +0.056	77	0	1	15	0	0	0	135	0	1270	2
T208	H1143	T208	AB	1	Eukaryotes	Mus muscul		X; 2.55	P16330	2',3'-cyclic-nucle	219, 131	770	0.54 (5AE0)	***	0.662 +0.321	76	52/35***/9**	57/47***/6**	15	2***	6/2***/2**	11/3***/4**	135	19/9***/5**	1072	231/70***/106**
T209	H1144	T209	AB	1	Eukaryotes	Mus muscul		X; 1.50	P16330	2',3'-cyclic-nucle	219, 122	895	0.54 (5AE0)	***	0.167 +0.238	77	7/4***	14/6***/5**	15	0	2**	4**	135	5**	1185	23/5**
T216	H1166	T216	AHL	1	Eukaryotes / Viruses	Homo sapie	7SUE	X; 2.90	P0DTC9	S24-188 Fab; Nu	216, 231, 130	1690	0.6 (7N0R)	0	0.101 + 0.045	72	0	0	15	0	0	0	135	0	1228	0
T217	H1167	T217	AHL	1	Eukaryotes / Viruses	Homo sapie	7STS	X; 2.16	P0DTC9	S24-188 Fab; Nu	212, 218, 130	1600	0.6 (7N0R)	0	0.066 +0.023	76	0	0	15	0	1	1	135	1	1247	2
T218	H1168	T218	AHL	1	Eukaryotes / Viruses	Homo sapie	7STR	X; 1.50	P0DTC9	S24-188 Fab; Nu	215, 222, 130	1820	0.6 (7N0R)	***	0.559 +0.229	76	58/55**	60/2***/55**	15	8/7**	12/11**	13/11**	135	53/39**	1254	201/134**
	**Large assemblies**																									
T195	T1115	T195	A16	1	Eukaryotes	Homo sapie		EM; 2.80	P27105	Stomatin	288	3350	7.98	**	0.345 +0.246	54	19/8**	25/12**	15	12/3**	13/5**	13/8**	134	110/31**	799	266/50**
T203	H1135		A9B3	4	Eukaryotes	Homo sapie		X; unknown	O94901, Q12912	SUN domain-con	195, 25															
		AU203.1	A3B1	3					O94901, Q12912		195, 25	1100, 850, 750	3.93 (6R2I)	***	0.437 +0.164	75	53/10***/1**	67/12***/1**	15	12	15	15	111	103	156	137
		AU203.2	A2	1					O94901		195	500		**	0.351 +0.086	71	7/3**	16/4**	15	4/1**	8/1**	10/2**	126	16/6**	175	28/1**
T204	H1137		ABCDEFG2IJ	8				EM; 3.10	unannotated		409, 343, 524, 547, 390, 518, 653, 266, 289		Not available													
		AU204.1	ABCDEF	2								6500, 2750		**	0.361 +0.200	62	21/3**	22/5**	15	13	14	14/2**	132	127/10**	434	159/24**
		AU204.2	A'6G2IJ	6								1500, 1500, 750, 2250, 750, 1000		**	0.375 +0.205	61	32/8**	38/14**	15	13/11**	14/12**	15/13**	118	117/94**	406	183/156**
T219	T1170		A6	3	Bacteria	Streptococc	7PBR	EM; 3.3	Q5M2B1	RuvB	318	1900														
T220	H1171		A6B1	4	Bacteria	Streptococc	7PBL	EM; 3.3	Q5M2B1, P66746	RuvB, RuvA	318, 48	1900, 680														
T221	H1172		A6B2	4	Bacteria	Streptococc	7PBP	EM; 3.3	Q5M2B1, P66746	RuvB, RuvA	318, 48	1900, 640														
		AU219	A2	1					Q5M2B1		318	1900	1.30 (3PFI)	**	0.608 +0.112	75	65/61**	67/65**	15	13**	14**	14**	135	100/88**	882	307/190**
		AU220	AB	1					Q5M2B1, P66746		318, 48	650	0.82	***	0.546 +0.231	63	51/15***/21**	54/18***/24**	15	12/1***/8**	12/3***/8**	13/3***/8**	90	50/2***/22**	850	370/190**
T230	T1192	T230	A10	1	Eukaryotes	Homo sapie		EM; 2.3	P43351	DNA repair prote	418	2225	0.78	***	0.753 +0.266	58	40/36***/4**	45/40***/4**	15	14/2***/12**	14/9***/5**	15/12***/3**	126	111/45***/64**	662	342/100***/218**
													rms to best template (AF or PDB)		colors following thresholds in dockq paper	CAPRI and CASP without overlap						boldface if all groups had acceptable+				
	**Table S5 - CASP15/CAPRI Target Master Table**																									

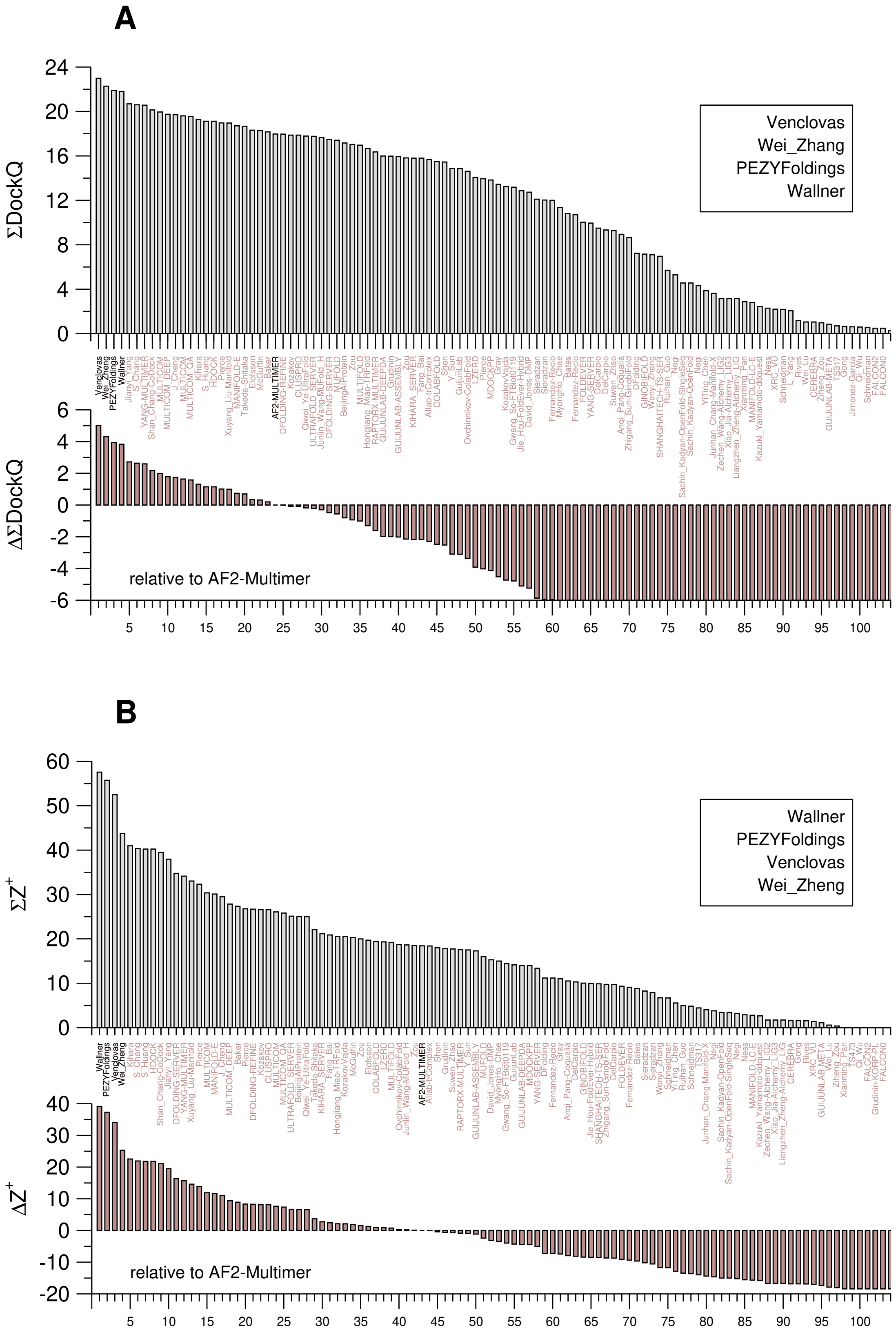

Figure S1 – Alternative ranking using the sum of DockQ (**A**) or sum of DockQ Z-scores (**B**). Only the best model of each Predictor is taken into account for the DockQ scores. For the Z-score ranking a normal distribution is assumed and only positive Z-scores are retained. Values correspond to the DockQ Sum and ΣZ^+ columns of Supplementary Table S4. The bottom panel of both figures show the ranking relative to the off-the-bench AF2-Multimer submission provided by the Elofsson group.

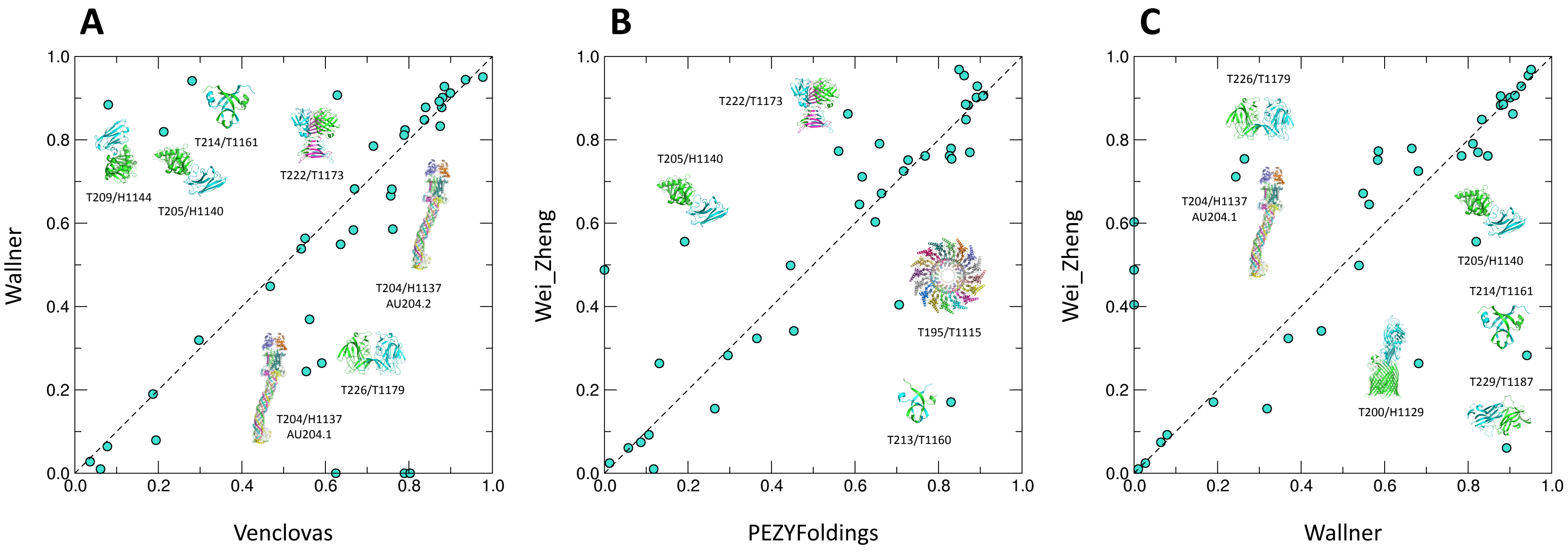

Figure S2 – Relative performance of the four top-performing groups.
Shown are the target cq. assessment unit pair-wise DockQ values for the two predictor groups indicated; a point in the lower triangle indicating better performance for the predictor group listed on the horizontal axis and likewise the vertical axis for the upper triangle. Selected off-diagonal targets are indicated by target picture and CAPRI and CASP target IDs. A. Wallner vs. Venclovas; B. Zheng vs. PEZYFoldings; C. Zheng vs. Wallner.

Protein complex assembly by employing programs AlphaFold2 and SwarmDock

Paul A. Bates

Biomolecular Modelling Laboratory, The Francis Crick Institute, 1 Midland Road, London NW1 1AT, UK
paul.bates@crick.ac.uk

The construction, optimization and docking of protein models remains challenging. All require extensive sampling of high-dimensional conformational space, which is intractable with methods based on exhaustive enumeration of all possible solutions. However, modern machine learning methods that incorporate information derived from the coevolution of residues in the modelling process, within and between protein chains [1, 2], have proven ability to limit the search space. Here a method is employed that takes account of coevolution for the construction of single protein chains to be docked, constructed *via* AlphaFold2, but not in the docking process itself, as could have been employed by the program AlphaFold-Multimer [2]. The idea being to assess the contribution coevolution plays in the docking of protein chains.

Methods

The methodology employed for single protein chain construction and subsequent docking of the chains can be described as follows:

i) Single chain construction using AlphaFold2

Single chain components for docking were modelled using AlphaFold2 [1]. Only the default settings, along with the recommended default sequence databases, were employed. No templates were used to build structures. The top ranked single chain models, as ranked by AlpaFold2, were used as input to our docking program, SwarmDock [3]. If time permitted, multiple runs of SwarmDock were performed using the lower ranked AlphaFold2 models; however, no more than the 5 models, returned by a single default setting run of AlphaFold2, were considered.

ii) Docking using SwarmDock

For the modelling of all protein complexes, from single chain models described above, a modification to our binary protein-docking algorithm SwarmDock [3] was employed. Our method uses the principles of Particle Swarm Optimization (PSO) [4] to search the parameter docking space. The Innovation added to our published method is, for homo-oligomers, to treat each particle within the swarm as an instance of a packed homo-oligomer, constrained by the appropriate symmetry operators. The objective is to optimize the particle space to find the most energetically favorable homo-oligomer. Particles move through a multi-parameter space by the optimization of two sets of parameters: orientations and translations of each monomeric unit relative to the imposed symmetry and linear combinations of normal modes that adjust the conformation of each monomer, in the presence of the other monomers, in this simultaneous docking process. For hetero-oligomeric structures we employed our standard SwarmDock protocol [5]. This docking methodology isn't template based. Moreover, additional information, such as potential sequence conservation at the protein-protein interface, was not considered. The ranking of docked poses was obtained using our 'democratic' scoring system, as previously described [6]. To an extent, we considered both the principle of 'conformational selection' and 'induced fit' in our docking procedure. Conformational

selection, by using a variety of starting protein conformations, obtained here by using alternative AlphaFold2 single chain models. Induced fit, is considered too since small adjustments are made in both the backbones and side-chains of the interacting protein chains upon docking *via* the employment of our PSO procedure, which smoothly adjusts the weights between normal modes of conformational space each single chain displays.

Results

For this round of CASP-CAPRI our results are quite modest. Only for targets classified as ‘easy’ (T191, T192, T193, T199, T200, T201, T203, T219, T220, T221, T225, T226 and T229), and two ‘difficult’ (T194 and T202), did the above method show some utility. However, for one target, T229 (Tobacco lectin Nictaba homodimer, PDB code 8AD2), the method did provide a notable model. This target although classified as ‘easy’, did prove to be problematic for most of the predictors with only 5 ‘high’, 1 ‘medium’ and 2 ‘acceptable’ submitted by the whole CAPRI community. The method described here produced one of these ‘high’ quality models, ranked second by the approach. The below figure shows the AlphaFold2 model used by SwarmDock to obtain the homodimer complex. Two flexible loops in the model are indicated in red (labelled Region I, residues V(88)AEGAGI and Region II, residues K(104)KKLPGELGR), two regions required to undergo subtle conformational adjustment to form the native interface of the homodimer. The program SwarmDock, with its ability to smoothly combine normal modes of motion during the docking process, is especially tuned to the fine conformational adjustments required.

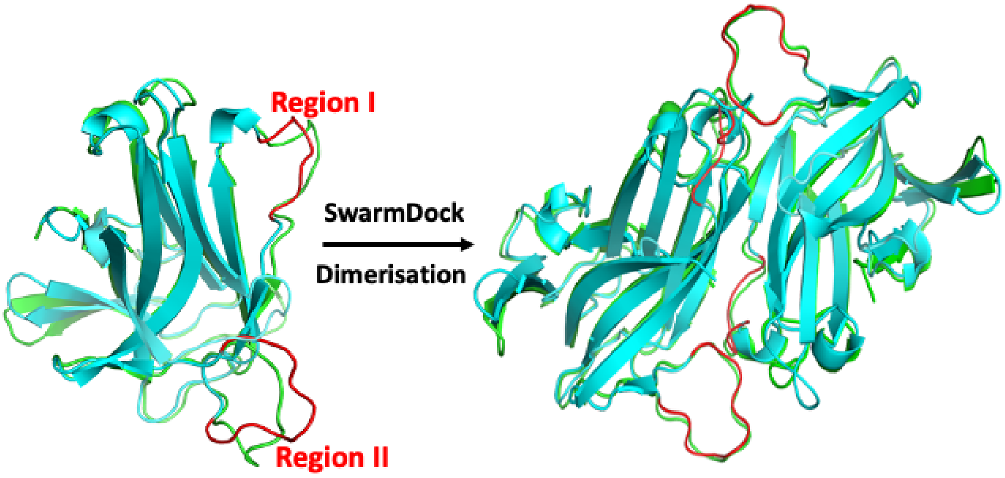

Figure: Model building for Target T229

Left-hand side cartoon shows superposition of the AlphaFold2 model (cyan) used for docking on the X-ray structure for one monomer of the dimeric X-ray structure (PDB code 8AD2, green). Two regions on the AlphaFold2 model are colored in red. To obtain the correct homodimer docking, these regions need to adjust their conformation towards the equivalent regions of the X-ray structure. The right-hand side cartoon shows superposition of the SwarmDock model (cyan) on the X-ray structure of the complexed dimer (green). The two previously described red regions, for which there are two copies in the dimer, marked on the SwarmDock model, can be seen to be in nearly identical conformation to their equivalent regions in the X-ray structure, regions that interact across the dimer interface forming several stabilizing backbone and side-chain hydrogen bonds.

Conclusion

As might be expected, to not take account of the principle of residue coevolution between complexed protein chains, *via* a program such as AlphaFold-Multimer, isn't competitive with methods that do. Nevertheless, the described method performs reasonably well for the easier targets. For the more difficult targets, the approach generally fails, underlining the need to take account of coevolution. However, it is perhaps easier to breakdown and understand the subtleties of conformational changes that are required upon complex formation for certain cases, such as for Target T229, by employing a two-phase docking strategy as described, rather than ubiquitously employing an end-to-end machine learning approach such as AlphaFold-Multimer, albeit the latter being generally a more successful methodology.

Funding

This work was supported by the Francis Crick Institute, which receives its core funding from Cancer Research UK (FC0001003), the UK Medical Research Council (FC001003), and the Wellcome Trust (FC001003).

References

1. Jumper, J., et al., *Highly accurate protein structure prediction with AlphaFold*. Nature, 2021.
2. Evans, R., et al., *Protein complex prediction with AlphaFold-Multimer*. bioRxiv, 2022: p. 2021.10.04.463034.
3. Moal, I.H. and P.A. Bates, *SwarmDock and the use of normal modes in protein-protein docking*. Int J Mol Sci, 2010. **11**(10): p. 3623-48.
4. Kennedy, J. and A.C. Eberhardt, *Particle swarm optimization*. IEEE Int Conf Neural Networks, 1995. **4**: p. 1942-1948.
5. Moal, I.H., et al., *A Guide for Protein-Protein Docking Using SwarmDock*. Methods Mol Biol, 2020. **2165**: p. 199-216.
6. Moal, I.H., et al., *IRaPPA: information retrieval based integration of biophysical models for protein assembly selection*. Bioinformatics, 2017.

HADDOCK scoring of CAPRI Round54 models.

Marco Giulini, Rodrigo V. Honorato, Charlotte van Noort, João M.C. Teixeira and A.M.J.J. Bonvin

Bijvoet Centre for Biomolecular Research, Faculty of Science - Chemistry, Utrecht University, Utrecht, the Netherlands
mgiulini@uu.nl, r.vargashonorato@uu.nl, c.w.vannoort@uu.nl, joaomcteixeira@gmail.com, a.m.j.j.bonvin@uu.nl

The HADDOCK team participated as a scorer group in the CAPRI 54 round. The scoring protocol was entirely based on the energetics of the complex, with no information coming from other sources such as deep learning methods or bioinformatic predictions. The scoring module of the new modular version of HADDOCK, HADDOCK3[1] (https://github.com/haddocking/haddock3/), was used throughout the entire round (in its beta version).

Methods

The HADDOCK scoring workflow consists of consecutive modules, namely: *Preprocessing* (including topology generation), *energy minimisation (EM) and scoring*, and *Fraction of Common Contacts (FCC) clustering*. In the *preprocessing* stage the input ensemble is parsed and potentially problematic models are identified and corrected. Topologies are created for each model and missing atoms are added. The protonation state of histidine residues and the presence of disulfide bonds are automatically handled. The *EM and scoring* step follows, which consists of a short energy minimisation (50 steepest descent EM steps) carried out with the OPLS[2] force field. The minimized models are then ranked based on their HADDOCK score (HS)[3]:

$$HS = 1.0\ E_{vdw} + 0.2\ E_{elec} + 1.0\ E_{desolv}$$

where E_{vdw} and E_{elec} correspond to the intermolecular van der Waals and electrostatic energies, respectively, and E_{desolv} is a solvent accessible surface area-dependent empirical desolvation energy term[4]. In the final *clustering* step, the structures are clustered using the FCC clustering algorithm[5]. This procedure lumps together models that share a consistent part of their interfacial contacts (≥60%). A minimum of four models is required to define a cluster, and the input structures that fail to satisfy this criterion are labeled as “unclustered”.

The model selection is based on the HADDOCK score of each cluster, calculated as the average score of the best four models. A short visual inspection is carried out to exclude biologically implausible complexes, whose excellent scores might be due to unphysical interactions. For submission, we typically select one model for each of the top five clusters as top 1-5 predictions. Positions 6-10 are usually filled with additional models coming either from the top clusters or from other clusters, depending on the case and number of clusters. Occasionally, unclustered structures with a particularly good HADDOCK score are also considered. Figure 1 shows an example of cluster-based HADDOCK3 output.

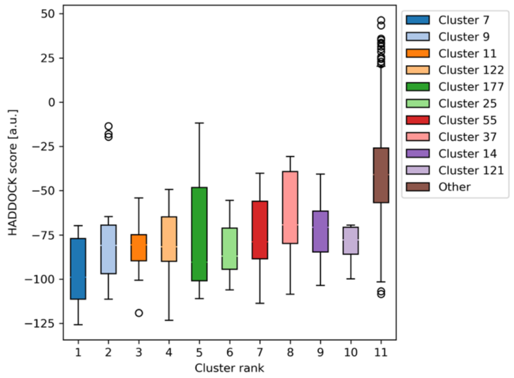

Figure 1: Example of HADDOCK3 cluster-based output. Here the clusters are ordered according to the average HADDOCK score of the top four ranked models.

Results and discussion

The HADDOCK team ranked in position 10 among the CAPRI scorers, with a total of 48 points and 23 acceptable targets. Among those, 7 are labelled as high quality, 11 as medium quality, and 5 as acceptable quality models. Table 1 provides a summary of the achieved performances for each target. Notably, the HADDOCK team was the only scorer to identify an acceptable model for target 229. The corresponding model (T229_S12.M03) possesses the fifth lowest value of HS over the whole scoring ensemble, with a particularly good electrostatic component E_{elec} (the fourth lowest). The four top models for T229 were all part of the same structural cluster, while T229_S12.M03 is labeled as "unclustered" and placed in third position in our re-scored submission ensemble.
As for the complexes for which no acceptable models were selected, HADDOCK3 performed poorly over almost all the targets containing nanobody-antigen complexes. Although the overall success rate over such targets has been quite low, a good number of acceptable models have been identified by other groups, especially for target 208. This type of complexes might require a reweighting of our scoring function.
19 out of the 268 interfaces submitted by the team have been disqualified because they shared low sequence identity with the target (16 cases) or because they present a high number of clashes (3 cases). For the former, this can be explained by the fact we did not check the sequence identity but trusted the provided models. The latter is however rather surprising as our HADDOCK will typically penalize models with clashes. The energy minimization can however generate hydrogen bonds with heavy atom distances below the 3Å cutoff used by CAPRI to detect clashes (note that hydrogen bonds can be shorter than 3Å).

Table 1: Target-specific performances of the HADDOCK team over the 38 assessed interfaces, expressed as number of acceptable (#acc), medium (#med), and high (#high) quality models among the top 5 submitted structures. The text between squared brackets specifies the ranking of the first model belonging to each category. * indicates assessment units in which more than one interface is involved: For T203-AU1 and T204-AU1 we calculate the score as the average of the available, interface-specific scores, while for T203-AU1, T219-220-221-AU1, and T219-220-221-AU2 the numbers refer to all the existing interfaces (consistently with the assessment criteria).

Target/Interface	# acc	# med	#high	points
T192	0	1 [5th]	4 [1st]	3
T193	1 [4th]	1 [5th]	3 [1st]	3
T194	0	2 [1st]	0	2
T195	3 [1st]	2 [2nd]	0	2
T197	0	0	0	0
T198	0	0	0	0
T199	1 [5th]	0	1 [1st]	3
T200	0	0	1 [3rd]	3
T201	0	0	4 [2nd]	3
T202	0	4 [1st]	1 [2nd]	3
T203-AU1*	5 [1st]	0	0	1
T203-AU2	0	0	0	0
T204-AU1*	2	0	0	1
T204-AU2*	1 [3rd]	4 [1st]	0	2
T205	0	0	0	0
T206	0	0	0	0
T207	0	0	0	0
T208	0	0	0	0
T209	0	0	0	0
T210	1 [2nd]	2 [1st]	0	2
T211	0	0	0	0
T212	1 [5th]	1 [1st]	0	2
T213	0	0	0	0
T214	0	0	0	0
T216	0	0	0	0
T217	0	0	0	0
T218	0	1 [3rd]	0	2
T219-220-221-AU1*	11	32	0	2
T219-220-221-AU2*	4	3	1	3
T222	2 [1st]	1 [2nd]	0	2
T223	1 [5th]	0	0	1
T224	0	0	0	0
T225	1 [5th]	1 [2nd]	0	2
T226	1 [5th]	0	0	1
T227	1 [5th]	3 [1st]	0	2
T229	1 [3rd]	0	0	1
T230	0	5 [1st]	0	2

Funding

Financial support from the European Union Horizon 2020, project BioExcel (823830) and from the Netherlands e-Science Center, project IVRESSE (027.020.G13) is acknowledged.

References

1. Dominguez C, Boelens R, Bonvin AMJJ. HADDOCK: A Protein−Protein Docking Approach Based on Biochemical or Biophysical Information. *J Am Chem Soc*. 2003;125(7):1731-1737.
2. Jorgensen WL, Tirado-Rives J. The OPLS [optimized potentials for liquid simulations] potential functions for proteins, energy minimizations for crystals of cyclic peptides and crambin. *J Am Chem Soc*. 1988;110(6):1657-1666
3. Vangone A, Rodrigues JPGLM, Xue LC, et al. Sense and simplicity in HADDOCK scoring: Lessons from CASP-CAPRI round 1. *Proteins Struct Funct Bioinforma*. 2017;85(3):417-423
4. Fernández-Recio J, Totrov M, Abagyan R. Identification of protein-protein interaction sites from docking energy landscapes. *J Mol Biol*. 2004;335(3):843-865
5. Rodrigues J.P.G.L.M., Trellet M., Schmitz C., Kastritis P.L., Karaca E., Melquiond A.S.J. and Bonvin A.M.J.J. Clustering biomolecular complexes by residue contacts similarity. *Proteins: Struc. Funct. & Bioinformatic*. 2021:80:1810-1817

Summary, Chang group, CAPRI Round 54 (CASP15-CAPRI)

Ren Kong, Hang Shi, Xufeng Lu, Shan Chang*
Institute of Bioinformatics and Medical Engineering, School of Electrical and Information Engineering, Jiangsu University of Technology, Changzhou 213001, China. E-mail: schang@jsut.edu.cn
Funding: National Natural Science Foundation of China (22003020)

In CASP15-CAPRI, our group combined the AlphaFold-Multimer(AFM) [1], and our hybrid docking strategy called CoDockPP [2, 3] for the docking and scoring experiments. For each target, we queried Protein Data Bank (PDB) for structures of protein homologs that can be used as template for modeling the homo and hetero protein complexes. The templates for each target are listed in Table 1. If no proper template was available, we used the AFM to build the complex, and use CoDock to perform re-docking, such as T194 and T196. For T195, T204 and T230, these targets have more than 4000 residues, so AFM is not suitable to modeling the whole complex directly. For these 3 targets, we modeled the partial structures by using AFM, and aligned the partial structures to produce the whole complex according to the template. Our group failed mainly in the 10 targets of T205, T206, T207, T213, T214, T216, T217, T223, T224 and T226. For T216 and T224, no group in CAPRI obtained acceptable models. The 5 targets of T205, T206, T207, T216 and T217 were antigen-antibody interactions, which were difficult for AFM and molecular docking. Although the template of T213 and T214 were found, it was also difficult to predict the acceptable conformations due to the different crystallization conditions. For targets of T223 and T226, AFM was used for modeling, but no acceptable model was obtained.

For scoring function, the knowledge-based scoring function included much more information of near-native structures in the observed pair distribution function, enabling it more robust for conformational changes.

Table 1. List of templates, docking methods and interface performance of our group (P19 and S19) for each target

CAPRI ID (CASP ID)	Template and method	Stocihiometry	Residue number	Prediction quality (Top-5)	
				Prediction	Scoring
T191(H1106)	7NTF	A1B1	236	1. 5/4***/1** [a]	No data
T192(T1109)	7L9Z	A2	454	1. 5/4***/1**	1. 5/4***/1**
T193(T1110)	7L9Z	A2	454	1. 5/5***	1. 5/5***
T194(T1113)	AFM [b]	A2	386	1. 5/4**	1. 5/4**
T195(T1115)	7VHP	A16	4608	1. 5/1**	1. 4
T196(T1119)	AFM	A3	459	No data	No data
T197(T1121)	AFM	A2	762	1. 2	1. 2
T198(T1123)	AFM	A2	532	1. 0	1. 2
T199(T1127)	2FE7	A2	422	1. 5/5***	1. 5/3***/2**
T200(H1129)	AFM	A1B1	1348	1. 4	1. 2
T201(T1132)	1X7V	A6	612	1. 5/3***/2**	1. 5/3***/2**
T202(H1134)	AFM	A1B1	543	1. 4/4**	1. 5/1***/4**
T203(H1135)	6R16	A9B3	1830	1. 5/5***<br>2. 3<br>3. 5<br>4. 5/3**	1. 5/4***/1**<br>2. 5<br>3. 5<br>4. 2/2**
T204(H1137)	7CGE	A1B1C1D1E1F 1G2H1I1	4592	1. 5<br>2. 5<br>3. 5/5***<br>4. 5/5***<br>5. 5/3***/2**<br>6. 5/5**	1. 5<br>2. 5<br>3. 5/4***/1**<br>4. 5/4***/1**<br>5. 5/3***/2**<br>6. 5/4**

				7. 5/1***/4**<br>8. 5/5**	7. 5/1***/4**<br>8. 5/1***/4**
T205(H1140) [c]	AFM	A1B1	351	1. 0	1. 0
T206(H1141)	AFM	A1B1	346	1. 0	1. 0
T207(H1142)	AFM	A1B1	347	1. 0	-- [d]
T208(H1143)	AFM	A1B1	350	1. 2/1***/1**	1. 0
T209(H1144)	AFM	A1B1	341	1. 1/1**	1. 1/1**
T210(H1151)	7KUG, 6ONO	A1B1	228	1. 5/3***/2**	1. 5/1***/3**
T211(T1153)	3DNI, 7KIU,<br>3MPR	A2	598	1. 2/2**	1. 1/1**
T212(H1157)	AFM	A1B1	1524	1. 5/5**	1. 4/4**
T213(T1160)	7DXS, 7DXR	A2	96	1. 0	--
T214(T1161)	7DXR	A2	96	1. 0	1. 0
T216(H1166)	7N3D	A1B1C1	577	--	--
T217(H1167)	AFM	A1B1C1	560	--	1. 0
T218(H1168)	7N3C	A1B1C1	567	1. 5/2**	1. 2/1**
T219(T1170)	3PFI, 1IXR, 1IXS	A6	1908	1. 5/1**<br>2. 5/5**<br>3. 5	1. 5/3**<br>2. 5/4**<br>3. 5
T220(H1171)	1IXR, 1IXS	A6B1	1956	1. 5/2**<br>2. 5/1**<br>3. 5/5**<br>4. 5/3**	1. 5/3**<br>2. 5/1**<br>3. 5/5**<br>4. 5/2**
T221(H1172)	1IXR, 1IXS	A6B2	2004	1. 5/2**<br>2. 5<br>3. 5<br>4. 5/5**	1. 5/2**<br>2. 5/1**<br>3. 5<br>4. 5/4**
T222(T1173)	AFM	A3	612	1. 5/2**	1. 5/3**
T223(T1174)	AFM	A3	1014	1. 0	1. 0
T224(T1176)	2BJM, 2GIX,<br>2DYC	A8	1360	--	--
T225(T1178)	AFM	A2	612	1. 5/5**	1. 5/5**
T226(T1179)	AFM	A2	522	1. 0	1. 0
T227(T1181)	4OJP, 6NW9	A3	2064	1. 1/1**	1. 3/3**
T228(T1184)	AFM	A3	513	No data	No data
T229(T1187)	AFM	A2	332	1. 1/1***	1. 0
T230(T1192)	5XRZ, 5XS0	A10	4180	1. 5/5**	1. 5/1***/4**

[a] First part was the serial number of interface. Second part was the number of targets for which an acceptable model or better was submitted by our group, followed by the number of targets with high (***) or medium (**) quality.

[b] AFM means the AlphaFold-Multimer is used for modeling the complex.

[c] In these 10 targets, our group didn't obtain acceptable or better models in prediction or scoring experiments.

[d] '--' represents no groups in CAPRI obtained acceptable models.

References:

[1] Evans R, O'Neill M, Pritzel A, Antropova N, Senior A, Green T, Žídek A, Bates R, et al. Protein complex prediction with AlphaFold-Multimer. *bioRxiv*. 2022; 2021.10.04.463034.

[2] Kong R, Wang F, Zhang J, Xu X J, Chang S. CoDockPP: a multistage approach for global and site-specific protein-protein docking. *J Chem Inf Model*. 2019; 59(8): 3556-3564.

[3] Kong R, Liu R R, Xu X M, Zhang D W, Xu X S, Shi H, Chang S. Template-based modeling and *ab-initio* docking using CoDock in CAPRI. *Proteins*. 2020; 88(8): 1100-1109.

Improving Multimer Structure Prediction by Sensitive Alignment Sampling, Template Identification, Model Ranking, Iterative Refinement in CASP15-CAPRI

Jian Liu, Zhiye Guo, Xiao Chen, Alex Morehead, Raj S. Roy, Tianqi Wu, Nabin Giri, Farhan Quadir, Chen Chen, Jianlin Cheng

Department of Electrical Engineering and Computer Science, University of Missouri, Columbia, MO 65211, USA

Emails: JL: jl4mc@mail.missouri.edu; ZG: zggc9@mail.missouri.edu; XC: xiaochen@mail.missouri.edu; AM: acmwhb@mail.missouri.edu; RR: rsr3gt@mail.missouri.edu; TW: tianqiwu@mail.missouri.edu; NG: ngzvh@missouri.edu; FQ: fqg7h@mail.missouri.edu; CC: chen.chen@mail.missouri.edu; JC: chengji@missouri.edu

For the 2022 CAPRI experiment, we developed a protein multimer prediction system on top of AlphaFold2 and AlphaFold2-multimer by improving the input fed to AlphaFold2 and AlphaFold2-Multimer and evaluating and refining their outputs to enhance the multimer structure prediction. The system was tested as one server predictor (MULTICOM_multimer) and one human predictor (MULTICOM_complex, Group ID: 3) in the assembly (multimer) structure prediction in the CASP15-CAPRI experiment.

Methods

Given a multimer target, our system first generates multiple sequence alignments and predict tertiary structures for each unit (chain) of the target (see Section 1 below), which are then use as the input to predict the quaternary structure of the multimer (see Section 2 below).

1. Monomer structure prediction for units of multimers

Monomer multiple sequence alignment (MSA) sampling. For each unique unit (chain) of a multimer target, a monomer alignment generation pipeline is applied to generate various kinds of MSAs by using HHblits[3,4], JackHMMER[5] and MMseqs2[6] to search the sequence databases, including UniRef30, UniRef90[7], BFD[8,9], MGnify clusters[10], UniProt[7], and the ColabFold DB[6]. Moreover, a DeepMSA-like alignment tool is executed in the background to search the huge Integrated Microbial Genomes (IMG) database to generate alternative alignments for hard targets having few homologous sequences. **Monomer Template identification**. In addition to using the templates identified by the default AlphaFold2, the MSA generated from UniRef90 is used to search our inhouse template database curated from Protein Data Bank[11] (PDB) to identify alternative templates. **Monomer structural model generation**. A customized version of AlphaFold2 is used to generate models using the MSAs and templates generated from the previous steps. Each combination of a MSA and a set of templates is used to generate five models. Multiple combinations of MSAs and templates lead to about 50 models generated for each target. If the depth of the MSA generated by the default AlphaFold2 is less than 200, the MSA generated from the IMG database is also used to generate more models. **Monomer Model ranking**. The APOLLO[12] model ranking score (the average pairwise structural similarity between models) and the global pLDDT score generated by AlphaFold2 are used to rank the structural models. The average of the two is also used to rank them.

2. Multimer structure prediction

Multimer MSA sampling. The MSAs of the subunits of a multimer target are concatenated using potential protein-protein interactions extracted from multiple sources such as the species information, UniProt accession IDs, the protein-protein interactions in the STRING[13] database, and the protein complexes in the PDB, resulting in a series of MSAs for the multimer. The predicted tertiary structures of the units of the multimer target are also searched against an inhouse complex template database built from PDB and against the single-chain

models in the AlphaFoldDB (the version released before March 2022) by FoldSeek[14] to identity similar structural units in a template complex or similar non-overlapped domains of an AlphaFoldDB model, whose sequences are concatenated to generate MSAs.

Multimer template identification. The sequence of the multimer is searched against PDB70 and an inhouse complex template database built from PDB by HHsearch[4] to identify the structural templates. The templates for each subunit are concatenated together if they share the same PDB code. Moreover, the predicted tertiary structural model of each unit of the multimer is searched against the inhouse structure template database by FoldSeek to identify more templates, which are concatenated as multimer templates.

Multimer structural model generation. Each combination of the concatenated MSAs and templates is fed for a customized AlphaFold2-Multimer to generate multimer structures. Usually, more than 100 models are generated for a target.

Multimer model ranking. MultimerEva, an inhouse tool of evaluating the quality of multimer models based on the average pairwise structural similarity score between models of a target, is used to rank the generated models. The structural similarity score is calculated by MM-align. The confidence score generated by AlphaFold2-Multimer is also used to rank the models. Finally, the average of the two is applied to rank the models as well.

Multimer model refinement. An initial target structural model is used as input for FoldSeek[14] to search for similar structures in the template database curated from the PDB and AlphaFoldDB (the version released before March 2022). The output of the FoldSeek includes the e-value of the similar structural hits as well as the structural alignments between the target model and the hits, which are converted into the sequence alignments between them. The MSAs of the subunits generated from the FoldSeek search are concatenated if they are from the same PDB complex structure or the non-overlapped regions of the same single-chain AlphaFoldDB model. The sequence alignments are added into the original MSA to generate a deeper MSA. The new MSA and the top-ranked structural hits found by FoldSeek are used as inputs for the customized AlphaFold2-Multimer to generate refined models. If the highest confidence score of the newly refined models is higher than the input model, the refinement process is repeated with the refined model as input until the number of the refinement iterations reaches 5.

CAPRI Predictors: Both the monomer and multimer prediction methods of our *in-house* system above were executed to generate the models for the multimer targets. Our server multimer predictor (MULTICOM_multimer) used the AlphaFold2-Multimer confidence score to rank multimer models. Due to the three-day time constraint, the refinement was only applied to some smaller multimer targets in the server prediction. For the human prediction (MULTICOM_complex; Group ID: 3), more multimer models were generated from more diverse MSAs thanks to a much longer timeline. The FoldSeek-based iterative model refinement was applied to most targets. MULTICOM_complex used MultimerEva score to rank and select models for final submission.

Results

On the 33 CAPRI targets whose experimental structures are available to us, the models predicted by MULTICOM_multimer and MULTICOM_complex have better quality than the models predicted by the standard version (v2.2.0) of AlphaFold-Multimer with default parameters (**Table 1** and **Figure 1**).

Table 1. Average per-target TM-score of top 1 ranked model or best of top five ranked models of MULTICOM_multimer, MULTICOM_complex, and AlphaFold-multimer on 33 CAPRI targets.

Predictor	Average TM-score of best of top 5 models	Average TM-score of top 1 model
MULTICOM_multimer	0.7992	0.7719
MULTICOM_complex	0.8003	0.7743
AlphaFold-Multimer	0.7639	0.7454

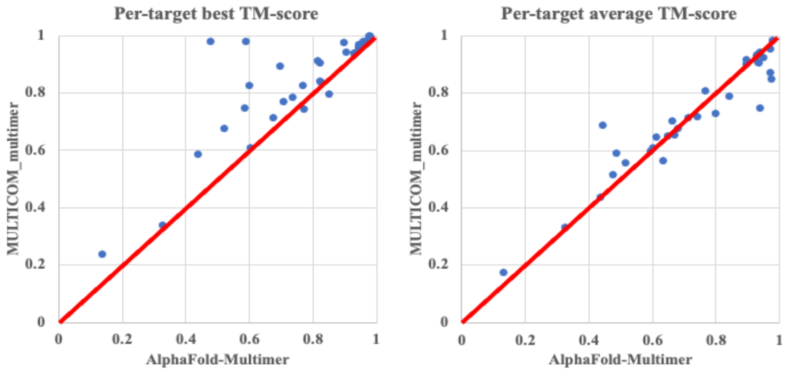

Figure 1. The per-target comparison of multimer models predicted by MULTICOM_multimer and AlphaFold-Multimer for 33 CAPRI targets. **Left**: the plot of the highest TM-score of all the models of each target generated by MULTICOM_multimer against that by AlphaFold-Multimer; **Right**: the plot of the average TM-score of all the models of each target generated by MULTICOM_multimer against that by AlphaFold-Multimer.

Funding
This work was partially supported by US National Science Foundation (grant #: DBI1759934 and IIS1763246), and US National Institutes of Health (grant #.: R01GM093123 and R01GM146340).

References

1. Jumper J, Evans R, Pritzel A, et al. Highly accurate protein structure prediction with AlphaFold. *Nature* 2021;596:583-589.
2. Evans R, O'Neill M, Pritzel A, et al. Protein complex prediction with AlphaFold-Multimer. *BioRxiv* 2021.
3. Remmert M, Biegert A, Hauser A, et al. HHblits: lightning-fast iterative protein sequence searching by HMM-HMM alignment. *Nature methods* 2012;9:173-175.
4. Steinegger M, Meier M, Mirdita M, et al. HH-suite3 for fast remote homology detection and deep protein annotation. *BMC bioinformatics* 2019;20:1-15.
5. Johnson LS, Eddy SR, Portugaly E. Hidden Markov model speed heuristic and iterative HMM search procedure. *BMC bioinformatics* 2010;11:1-8.
6. Mirdita M, Schütze K, Moriwaki Y, et al. ColabFold: making protein folding accessible to all. *Nature Methods* 2022:1-4.
7. Consortium U. UniProt: a worldwide hub of protein knowledge. *Nucleic acids research* 2019;47:D506-D515.
8. Steinegger M, Mirdita M, Söding J. Protein-level assembly increases protein sequence recovery from metagenomic samples manyfold. *Nature methods* 2019;16:603-606.
9. Steinegger M, Söding J. Clustering huge protein sequence sets in linear time. *Nature communications* 2018;9:1-8.
10. Mitchell AL, Almeida A, Beracochea M, et al. MGnify: the microbiome analysis resource in 2020. *Nucleic acids research* 2020;48:D570-D578.
11. Sussman JL, Lin D, Jiang J, et al. Protein Data Bank (PDB): database of three-dimensional structural information of biological macromolecules. *Acta Crystallographica Section D: Biological Crystallography* 1998;54:1078-1084.
12. Wang Z, Eickholt J, Cheng J. APOLLO: a quality assessment service for single and multiple protein models. *Bioinformatics* 2011;27:1715-1716.

13. Mering Cv, Huynen M, Jaeggi D, et al. STRING: a database of predicted functional associations between proteins. *Nucleic acids research* 2003;31:258-261.
14. van Kempen M, Kim S, Tumescheit C, et al. Foldseek: fast and accurate protein structure search. *bioRxiv* 2022.

Multimer Model Scoring Based on Gated-Graph Transformer and Steerable Equivariant Graph Neural Networks in CASP15-CAPRI

Xiao Chen, Alex Morehead, Jian Liu, Zhiye Guo, Raj S. Roy, Tianqi Wu, Nabin Giri, Farhan Quadir, Chen Chen, Jianlin Cheng

Department of Electrical Engineering and Computer Science, University of Missouri, Columbia, MO 65211, USA

Emails: XC: xiaochen@mail.missouri.edu; AM: acmwhb@mail.missouri.edu; JL: jl4mc@mail.missouri.edu; ZG: zggc9@mail.missouri.edu; RR: rsr3gt@mail.missouri.edu; TW: tianqiwu@mail.missouri.edu; NG: ngzvh@missouri.edu; FQ: fqg7h@mail.missouri.edu; CC: chen.chen@mail.missouri.edu; JC: chengji@missouri.edu

In the CASP15-CAPRI experiment, we deployed two different methods for scoring predicted multimer models. Our server scorer MULTICOM_multimer is a *single-model* deep learning method using our Gated neighborhood-modulating Graph Transformer (GGT) architecture to predict the global quality score of a multimer model. Our human scorer MULTICOM_complex is a *single-model* deep learning method that uses a Steerable Equivariant Graph Neural Network (SEGNN) to predict both the per-residue local distance difference test (lDDT) scores and the global quality score of a multimer model.

Methods

MULTICOM_multimer (server)

MULTICOM_multimer is built on top of our in-house *single-model* quality assessment method – DProQ [1] that takes a multimer model as input and represents it as a 3D graph to predict the DockQ [2] score of the model. The node features include one-hot amino acid encoding, secondary structure type, relative accessible surface area, phi angle, psi angle, and graph Laplacian positional encoding of residues. The edge features include Ca-Ca distance, Cb-Cb distance, N-O distance, inter-chain contact encoding, permutation-invariant chain encoding, and edgewise positional encoding. DProQ uses the GGT architecture to update node and edge embeddings during graph message passing to predict the DockQ score of the multimer model. It does so by performing message passing on protein graph topologies induced by a 3D k-nearest neighbours definition of edges between amino acid residues (i.e., nodes). Importantly, the GGT introduces a novel approach to regulating information flow between nodes: incorporating learnable node feature gates and edge feature gates into the graph attention-based network architecture. Our ablations of such network components reveal that they enhance the ability of the GGT to assess within DProQ the quality of input protein complex structures.

MULTICOM_complex (human group ID: 3)

Our human scoring method is based on our in-house *single-model* quality assessment method – DeepRefine [3]. It represents a multimer model as a 3D graph, where the nodes of the graph correspond to the amino acid residues in the model and the edges are defined according to each residue's 20 nearest neighbors in 3D space. For this 3D graph, DeepRefine generates features for each of its nodes and edges such as cosinusoidal and sinusoidal encodings of the residue's backbone dihedral angles and distances between residues. On such graphs, we then apply the SEGNN [4] model to predict the input structure's quality (i.e., nativeness). Specifically, DeepRefine predicts the lDDT score corresponding to the nativeness of the 3D position of each node (residue). The average of the per-residue lDDT scores is used as the predicted global quality of the multimer model. Notably, we predict such lDDT scores by performing 3D graph message passing using scalar and vector-valued node and edge features derived from the input protein complex's 3D structure such as spherical

harmonics embeddings of the relative distance and direction between two nodes in 3D space. We then average such edge-focused embeddings to provide us with additional node-focused geometric vector features on which we apply SEGNN message passing to predict per-node lDDT scores, allowing our model to learn from protein complex structures directly from a geometric perspective.

Results

MULTICOM_multimer and MULTICOM_complex were blindly tested in the CASP15-CAPRI experiment (CAPRI round 54). Both methods evaluated the models of all 40 CAPRI targets. There were usually over 1000 predicted decoys for each target. Here we analyse MULTICOM_multimer's results on Target 224 (CASP target ID: T1176) as an example. Target 224 is a homo-multimer consisting of 8 chains.

We calculated the loss of ranking the models of each target in terms of TM_score [5]. MULTICOM_mulitmer's ranking loss for Target 224 is 0.0167. MULTICOM_mulitmer's top selected model is ranked among top 10% in the model pool.

Availability

DProQA (MULTICOM_multimer) is available at: DPROQA.

Funding
This work was partially supported by US National Science Foundation (grant #: DBI1759934 and IIS1763246), and US National Institutes of Health (grant #.: R01GM093123 and R01GM146340).

References

1. Chen, X., Morehead, A., Liu, J., & Cheng, J. (2022). DProQ: A Gated-Graph Transformer for Protein Complex Structure Assessment. *bioRxiv*.

2. Basu, S., & Wallner, B. (2016). DockQ: a quality measure for protein-protein docking models. *PloS one*, *11*(8), e0161879.

3. Morehead, A., Chen, X., Wu, T., Liu, J., & Cheng, J. (2022). EGR: Equivariant Graph Refinement and Assessment of 3D Protein Complex Structures. *arXiv preprint arXiv:2205.10390.*

4. Brandstetter, Johannes, et al. (2021). "Geometric and physical quantities improve E(3) equivariant message passing." *arXiv preprint arXiv:2110.02905*.

5. J. Xu, Y. Zhang, *How significant is a protein structure similarity with TM-score=0.5?* Bioinformatics, 26, 889-895 (2010)

EXPLORING THE SYNERGY OF DEEP LEARNING AND MIAX FOR PROTEIN-PROTEIN INTERACTION PREDICTION IN CAPRI ROUND 54

Carlos A. Del Carpio Muñoz[1], Eichiro Ichiishi[2]

[1] *Choju-Medical Institute, Fukushimuta Hospital. Noyori-cho, Yamanaka 19-14. Toyohashi-City, Aichi-ken 441-8124, Japan*

[2]*International University of Health and Welfare (IUHV Hospital) 537 Iguchi, Nasushiobara-City, Japan*

Abstract

The computational platform used by our group to predict protein complex configurations in the CAPRI round 54 (5th joint CASP-CAPRI) assembly prediction challenge consists in combining the strengths of our original MIAX (Macromolecular Interaction Assessment Computational System)[1,2,3] and the revolutionary protein 3D prediction capabilities of the AlphaFold system[2]. The results have been highly satisfactory, leading us to conclude that our protein interation (PPI) prediction system MIAX is highly efficient.

Methodology

The accuracy of the 3D protein structures predicted with our original homology based modules is improved by comparing them to those generated by the AlphaFold system. The optimal interacting structures are then input to MIAX which firstly identifies crucial regions on the protein surfaces that are highly likely to be involved in protein-protein interactions(PPI's). The algorithm based on clustering amino acids of the surface of the interacting proteins according to a particular amino acid physicochemical property using self organizing maps (SOM) and fourier transforms has been described elsewhere[1,3]. After the docking module in MIAX is executed the selection of potential complexes is achieved through a comparison of the interaction interfaces of the decoys with the interaction clusters predicted by MIAX.

The selection of candidate complex configurations is achieved by comparing the set of amino acids in the interaction surfaces of the series of MIAX output decoys with those in the MIAX-predicted interaction clusters. As depicted in the upper part of Fig. 1 (A), the interaction surface in the best decoy (shown in red) for taget T1173 has a significant overlap with the interaction cluster predicted by MIAX (in blue).

Results

Fig. 1.B lists the values plotted in Fig. 1.C to illustrate the correlation between the CASP-evaluated interface root mean square(RMSD) values for 28 assembly targets in CAPRI 54 and the composition of the surface of the receptor and ligand subunits, as inferred by the MIAX analytical module. A strong relationship between the CASP interface RMSD values and the hydrophobic regions on the MIAX hydrophobic clusters is clearly visible, i.e. for small rmsd values the overlap of the MIAX clusters with the interface of the putative complex is generally high. In conclusion, the results of our analysis in CAPRI 54 demonstrate the effectiveness of our system for PPI prediction, which is a combination of the MIAX system and the AlphaFold program. The MIAX system accurately identifies key hydrophobic regions on the protein surfaces that are likely to be involved in interactions, while the AlphaFold system assists in

optimizing the predicted 3D protein structures. The correlation between the CASP-evaluated interface RMSD values and the hydrophobic regions on the MIAX provides further evidence of the validity and robustness of our system. Overall, our findings highlight the synergy between the MIAX system and AlphaFold program, making it a valuable tool for predicting PPI's.

(A)

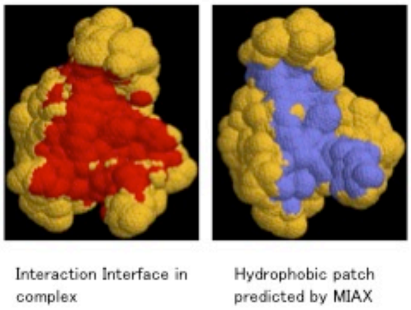

(B)

Target	CASP RMSD (interf)	Hits/Interf REC (%)	(MIAX)	LIG (%)	Hits/Interf (MIAX)
H1106TS447_3	1.34	17/19	(89.47)	5/17	(29.41)
T1109TS447_1	20.59	16/24	(66.67)	16/24	(66.67)
T1110TS447_1	0.72	19/22	(86.36)	19/22	(86.36)
T1113TS447_5	1.96	20/33	(60.61)	20/33	(60.61)
T1121TS447_1	3.52	9/27	(33.33)	7/17	(41.18)
T1123TS447_2	10.72	20/33	(60.61)	20/32	(62.50)
T1124TS447_1	2.32	30/44	(68.18)	23/53	(45.10)
T1127TS447_2	4.56	22/26	(84.65)	23/26	(88.46)
H1129TS447_3	23.03	11/17	(64.71)	6/16	(37.50)
T1132TS447_4	0.72	23/30	(76.67)	25/39	(83.33)
H1134TS447_4	19.02	8/21	(38.10)	11/25	(44.00)
H1140TS447_4	13.26	5/13	(38.47)	5/16	(31.25)
H1141TS447_2	16.12	2/6	(33.33)	7/12	(58.30)
H1142TS447_5	11.92	9/12	(75.0)	2/13	(15.35)
H1143TS447_2	10.61	2/12	(16.67)	3/7	(42.86)
H1144TS447_2	9.37	12/23	(52.17)	12/23	(52.17)
H1151TS447_5	2.31	0/14	(0.00)	4/11	(36.36)
T1153TS447_2	7.64		0		0
H1157TS447_1	25.97	11/23	(47.83)	15/42	(5.71)
H1166TS447_3	2.02	6/11	(44.55)	4/9	(44.44)
H1168TS447_1	2.12	4/7	(57.143)	4/9	(44.44)
T1170TS447_5	4.9	8/9	(88.89)	7/7	(100.0)
T1173TS447_2	1.06	22/43	(51.12)	18/45	(40.00)
T1178TS447_1	5.66	22/37	(59.46)	22/35	(62.86)
T1179TS447_1	18.12	7/22	(31.82)	10/18	(55.56)
T1181TS447_1	18.37	15/61	(24.59)	25/61	(40.98)
H1185TS447_2	1.21	36/40	(90.00)	24/37	(64.86)
T1187TS447_1	2.62	4/8	(50.00)	1/7	(14.28)

(C)

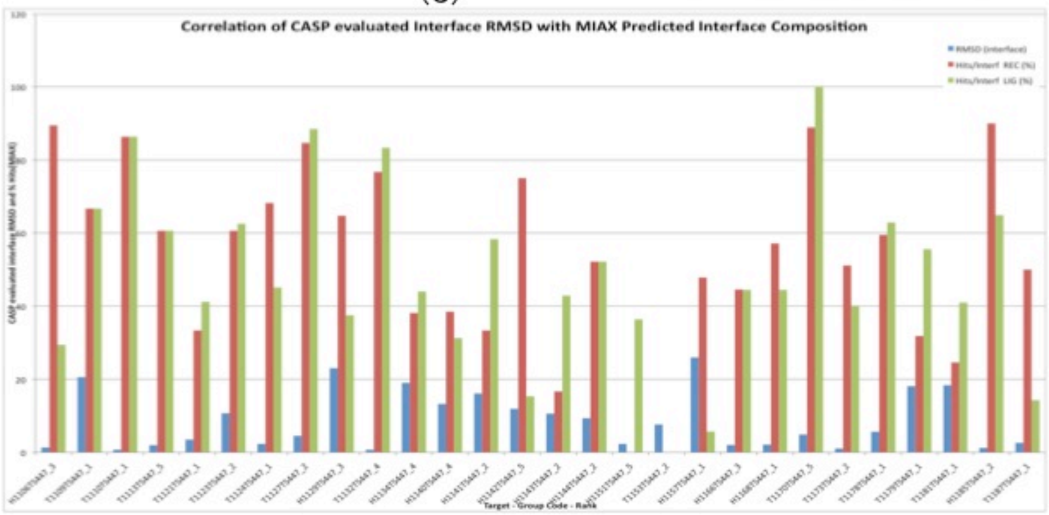

Fig.1 (A) Interaction Interface in complex (red), Hydrophobic pathch predicted by MIAX(blue). (B) Table of CASP evaluated interface RMSD and amino acid hits in MIAX predicted interaction regions and the interface.

References.

1. *Del Carpio C.A., Ichiishi E., Yoshimori A., Yoshikawa T.; "MIAX: A New Paradigm to Model Bio-Molecular Interaction and Complex Formation in Condensed Phases",PROTEINS: Structure, Function and Genetics, 48(4) (2002), 696-732*

2. *Jumper, J., Evans, R., Pritzel, A. et al*. "Highly accurate protein structure prediction with AlphaFold". Nature 596,583-589(2021).

3. *Del Carpio, C. A., and Eiichiro I.* "Inference of Structure and Dynamic Order of Formation of Multimeric Protein Complexes." Current Advances in Chemistry and Biochemistry Vol. 9 (2021): 93-112.

Fernandez-Recio's group performance in CASP15 / CAPRI round 54

Luis A. Rodríguez-Lumbreras,[1,2] and Juan Fernandez-Recio[1,2]

[1] Instituto de Ciencias de la Vida y del Vino (ICVV), CSIC - Universidad de La Rioja - Gobierno de La Rioja, Logroño, Spain
[2] Barcelona Supercomputing Center (BSC), Barcelona, Spain

e-mails:
luisangel.rodriguez@icvv.es (Luis A. Rodriguez-Lumbreras)
juan.fernandezrecio@icvv.es (Juan Fernandez-Recio)

Funding:
Spanish Ministry of Science (grant PID2019-110167RB-I00 / AEI /10.13039/501100011033)

We have participated as human predictors and scorers in all of the proposed targets (except T204 as predictors). We explored here a mostly automatic strategy for the modeling of protein assemblies based on docking and energy scoring with pyDock [1], using models of the interacting subunits generated by AlphaFold [2], and a minimal input from AF-Multimer [3] to either confirm or complement the energy-based ranking of assembly models.

Methods

For each assembly, the coordinates of the individual subunits were taken from the AlphaFold2 models available at CASP site by Elofsson group (except for some subunits in targets T203 and T210, which had available structure). The AF2 models were further processed to keep only reliable residues, based on pLDDT values, initially using a cutoff value of pLDDT > 60, and then pLDDT >70 (in some cases we tolerated smaller pLDDT values to avoid removing >30% of the protein sequence to pass CAPRI server verification).

As predictors, we applied our pyDock [1] docking pipeline to the individual subunits, in order to build the binary interactions in each assembly, using FTDock (electrostatics on; 0.7 Å grid resolution) to generate 10,000 rigid-body docking poses. In homo-oligomers, we assume symmetry and removed docking poses not satisfying the expected symmetry (e.g. cyclic C_2 symmetry for homo-dimers; C_3 for homo-trimers) within a given tolerance [4]. In target T203, the homo-trimer (A3) is directly taken from available x-ray structure (PDB 6R15), hetero-tetrameric interfaces (A3B1) were built based by superimposition on an available template (PDB code 6WME), and these models were used as input for *ab initio* docking to build the final assembly as a trimer of hetero-tetramers (A9B3). The protein-peptide interfaces of targets T220 and T221 (considered as 1 assessment unit) were built by docking with restraints from template PDB 1IXR (using pyDockRST [5]). In target T230, the individual subunits were taken from the AF-Multimer assembly models available at CASP site by Elofsson group, then docked with pyDock, and the resulting docking models were selected using restraints (with pyDockRST) from an available template (PDB code 1KN0).

All docking models were scored and sorted by pyDock energy-based function. After removing redundant models (within 4 Å ligand RMSD), we selected automatically the 100 best-ranked models (per rules of CAPRI) from pyDock scoring. In all cases, except for those based on available templates, i.e. T203 (2 assessment units), T230, or T220-221 (1 assessment unit), we compared the models generated by pyDock with the rank #1 assembly model generated by AF-Multimer by Elofsson group. Then we followed different submission strategies. In nine cases, the 10 best-ranked models from pyDock scoring contained docking poses that were similar (within 10 Å ligand RMSD) to the AF-Multimer assembly models. In these cases, given the consistency between the energy-based pyDock and the AF-Multimer predictions, we directly submitted the docking set automatically generated with pyDock. In the rest of the cases, to build a more reliable submission set, we sorted the entire set of

FTDock docking models by similarity to the AF-Multimer predictions, and inserted (in alternative order) the docking models as sorted by pyDock scoring. In a few cases (T222, T223, T224, T227) no model with ligand RMSD < 20 Å from AF-Multimer predictions was found in the entire FTDock set, so we submitted the docking models sorted by pyDock. Before submission, all models were minimized with AMBER to remove clashes and improve geometries.

In the scorers experiment, we first removed models with more than 25 clashes (i.e., intermolecular pairs of atoms closer than 3 Å) per interface. Then, we applied pyDock scoring and used the same criteria to rank the docking models as in predictors (i.e. filter by symmetry, check available templates, compare to AF-Multimer, and minimize clashes).

Results

As predictors, we were successful in 16 of the 38 assessed interfaces (42% success rate). This performance is slightly better than the one we obtained in last CASP14 (36% success rate).

The majority of the successful cases (10 out of 16) were obtained by our standard pyDock scoring (applied to either *ab initio* docking or template-based models). In 5 of these cases, the successful models turned out to be similar to AF-Multimer assembly predictions provided by Eloffson group. In 3 cases, the successful models were not considered to be similar to AF-Multimer predictions based on ligand RMSD. In the remaining 2 cases, models were built based on templates and were not compared to AF-Multimer predictions. We later checked that AF-Multimer (Eloffson group) was also successful in these 10 cases.

The rest of our successful cases (6 out of 16) came from docking poses that were added because of their similarity to the AF-Multimer predictions. We checked that AF-Multimer was successful in 5 of these cases. Interestingly, in one case (T200) AF-Multimer submissions were not correct while a docking model similar to AF-Multimer predictions was acceptable.

Discussion

On the one side, we found that 56% of the cases where pyDock scoring found similar models to AF-Multimer predictions were successful. Some of the unsuccessful cases could have been easily corrected with a more careful strategy. For instance, in T198 the correct AF-Multimer model was actually rank #2, but we only used rank #1 to compare with our models (perhaps in the future we should check more AF models, not just the first one). In T219 we had docking models similar to AF-Multimer but they were removed due to clashes when building the assembly. In T229, the docking model similar to AF-Multimer was ranked 10, but only the top 5 were evaluated. Finally, in T197, the AF-Multimer models were incorrect, but this is more difficult to solve.

On the other side, from the cases where we selected the most similar docking models to AF-Multimer predictions, only 37% were successful. In a few cases, despite AF-Multimer showing correct predictions, our models were unsuccessful perhaps because of limited docking sampling, or because the rank #1 AF-Multimer model was not correct, like in T226. But in the majority of the unsuccessful cases, the reason of failure is that AF-Multimer predictions were not correct. More than half of these cases were antibody-antigen complexes, which are still highly challenging.

Finally, from the cases where our entire docking set did not (apparently) contain any model similar to AF-Multimer predictions, we were successful in only one case (T222), where we got an acceptable model based solely on docking. This model was mistakenly considered dissimilar to the AF-Multimer prediction. We checked later that ligand RMSD alone was not suitable for comparison in this and other cases, perhaps because the conformation of unbound AF models and bound AF-Multimer subunits were very different (Figure 1). We could improve comparison of energy-based and AF-Multimer models by using interface RMSD and fraction of native contacts in addition to ligand RMSD. And a deeper sampling strategy could also help to generate orientations sufficiently similar to AF-Multimer models.

Among the targets with available templates, we failed in T220, where we obtained a correct model with rank 6, probably because we used docking and template-based restraints instead of just superimposing the unbound subunits on the template.

Regarding scorers, we were successful in 23 of the 37 assessed interfaces (62% success rate), similar to the performance of AF-Multimer in predictors.

In summary, our main goal here was to explore the use of *ab initio* docking and scoring with pyDock, with input from available AF-based models. We have used template-based modeling only for a few cases, due to limited time and resources. When analyzing the results, we found that the AF models of the unbound subunits have worked well in docking, comparable to the use of x-ray unbound structures. Regarding the quality of the models, a central strategy in this CASP15 edition has been the comparison of the docking models with the AF-Multimer predictions. Based on the results, this is indeed a significant step towards reliable modeling of protein interactions, but we need to further explore other possibilities for more accurate and automatic integration of energy- and AI-based docking approaches.

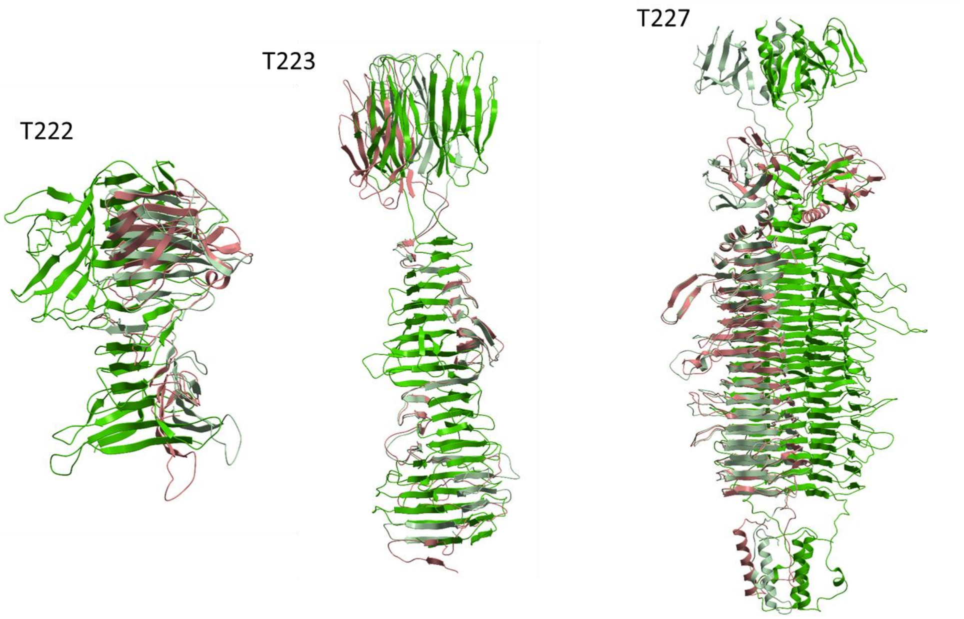

Figure 1. Targets where we failed to find docking models similar to the correct AF-Multimer predictions (in green). The AF2 unbound models used for docking (in pink) clashed with the other subunits in the AF-Multimer model (dark green).

[1] Cheng, T.M.-K., Blundell. T.L. & Fernandez-Recio, J. (2007) pyDock: electrostatics and desolvation for effective rigid-body protein-protein docking. *Proteins* ***68***, 503-515.

[2] Jumper, J., Evans, R., Pritzel, A., et al. (2021) Highly accurate protein structure prediction with AlphaFold. *Nature* ***596***, 583-589.

[3] Evans, R., O'Neill, M., Pritzel, A., et al. (2022) Protein complex prediction with AlphaFold-Multimer. bioRxiv. https://doi.org/10.1101/2021.10.04.463034

[4] Rosell, M., Rodríguez-Lumbreras, L.A., Romero-Durana, M., et al. (2020) Integrative modeling of protein-protein interactions with pyDock for the new docking challenges. *Proteins* ***88***, 999-1008.

[5] Chelliah, V., Blundell, T.L., Fernández-Recio, J. (2006) Efficient restraints for protein-protein docking by comparison of observed amino acid substitution patterns with those predicted from local environment. *J.Mol.Biol.* ***357***, 1669-1682.

Rosetta-based docking strategies for flexible protein-protein docking in the CASP15-CAPRI experiment

Ameya Harmalkar[a], Lee-Shin Chu[a], Sam Canner[b], Rituparna Samanta[a], Jeffrey J. Gray[a,b]

[a]*Department of Chemical and Biomolecular Engineering, Johns Hopkins University, Baltimore, MD, USA*

[b]*Program in Molecular Biophysics, Johns Hopkins University, Baltimore, MD, USA*

Correspondence*: jgray@jhu.edu*

Introduction

Prior CAPRI (Critical Assessment of Protein Interaction) challenges revealed that binding-induced conformational flexibility still confounds protein docking algorithms and hampers the accuracy of docking predictions[1]. In CASP14, AlphaFold[2,3] made a breakthrough in the field of protein sequence-to-structure prediction by predicting over two-thirds of CASP14 target proteins with global distance test scores above 90 out of 100[4]. This CASP15-CAPRI challenge is the first to blindly assess docking where participants have access to AlphaFold and other deep learning (DL) algorithms[5]. In this edition of CASP15-CAPRI, we expanded our docking methods in Rosetta and incorporated available monomer and complex predictions from AlphaFold, to tackle the binding-induced conformational flexibility in protein-protein docking. To model the targets in this round, we employed our recent progress in induced-fit docking with ReplicaDock 2.0[6], and our prior conformer-selection approaches, RosettaDock4[7] and SymDock2[8]. ReplicaDock 2.0[6] is a replica exchange Monte Carlo (MC) protocol that mimics induced-fit binding by capturing backbone motions over interfacial residues on-the-fly while docking. RosettaDock 4.0[7] on the other hand, uses conformer-selection by first pre-generating backbone ensembles and then docking proteins with backbone swaps from the ensemble. SymDock[9] is a symmetric docking protocol to dock monomeric subunits with user-specified symmetry to generate homomeric structures. Further, we also employed our structure prediction tools, i.e., IgFold[10], for generating antibody structures for antibody-antigen complexes.

Model Curation

The availability of predicted monomer and complex structures from AlphaFold and RoseTTAFold simplified the model curation for this round of CASP15-CAPRI. In a few cases, we relied on conventional ab initio structure prediction tools when the structures obtained had disordered regions or domains with low pLDDT (predicted Local Distance Difference Test) scores (under 40). Further, irrespective of the pLDDT scores from the DL methods, for all the targets, we used the BLAST program to identify homologous proteins and search for templates structures to compare with AlphaFold[2,3] and RoseTTAFold[11] predicted structures. We observed that for protein complex structure predictions from AlphaFold, the confidence was low at potential interfaces, particularly for loops. Especially for antibody-antigen complexes, the low confidence at interface regions (often comprising the CDR loops of the antibody) resulted in false positive binding poses. This highlights the need for docking algorithms to capture binding-induced conformational changes.

Docking Methods:

To obtain putative binding decoys, we modeled the complex structures using AlphaFold-multimer. We classified the targets based on their stoichiometry, i.e., heterodimers, homomers and multimers. Further, antibody- and nanobody-antigen complexes are categorized separately. From

the assessment results, we observed that for most targets, AlphaFold-multimer predictions served as good models to initiate local docking with more backbone flexibility. However, for antibody-antigen complexes and a few homomer targets, using AlphaFold models often skewed the results to false positive interfaces resulting in poor predictions. Here we discuss the docking methods we used in the CASP15-CAPRI round for each of the categories.

Heterodimers:

To dock heterodimeric complexes, we used a conformer-selection method, RosettaDock 4.0[7], and an induced-fit method, ReplicaDock 2.0[6]. For conformer-selection, we obtain the binding pose from AlphaFold-multimer predictions and dock the protein partners after generating an ensemble of structures. We diversify the backbone conformations in the ensemble by generating structures using Rosetta Relax, Rosetta Backrub, and normal modes. For induced-fit, we initiate docking from the same binding pose as defined prior, however, we allow on-the-fly backbone motions on the putative interface while docking. Each ReplicaDock local docking simulation spans over three temperature replicas, with inverse temperatures set to β, of 1.5^{-1} $kcal^{-1}$.mol, 3^{-1} $kcal^{-1}$.mol and 5^{-1} $kcal^{-1}$.mol. Replica exchange swaps are attempted every 1000 MC (Monte Carlo) steps generating 6,000 decoys at a local binding site. Our predictions perform well (refer Table 1, Heterodimers) for heterodimeric targets with models for four out of five targets scoring in medium/high category. The exception was target 200, a membrane protein and phage protein complex with disordered regions on interface.

Homodimers:

For homodimers and symmetric complexes, we utilize the symmetry framework in our docking protocol, SymDock2[8]. In CASP15-CAPRI round, we modelled 12 homomeric targets with A2 symmetry. Monomer predictions and homodimer models were obtained from AlphaFold and AlphaFold-multimer, respectively. In cases with disordered regions on the N- and C-termini regions, we either truncated the residues or built the monomer using ab-initio modelling on Robetta.[12,13] Additionally, as for heterodimeric targets, we generated an ensemble of structures. SymDock2 incorporates backbone ensembles and relaxation in high-resolution stage that enables sampling of tighter, complementary interfaces with better packing for symmetric complexes. Figure 1 demonstrates our performance for model T194 in comparison with an AlphaFold model. Our docking protocols refine and model better interface resulting in a medium CAPRI-quality target (DockQ = 0.77 vs 0.62 for AF2-multimer). Although the AF2 generated structure is in the right binding region, our docking strategy packs a tighter interface as indicated by the shift in orientation of one of the protein partners.

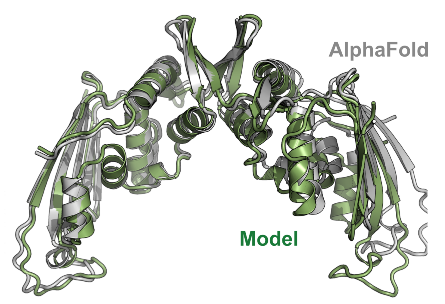

Figure 1: Prediction for Target T194 (homodimer with A2 symmetry). Our model (in green) highlighted over AlphaFold model (in gray).

Despite this robust strategy, our approach could not sample high accuracy decoys for most homodimers. One of the barriers was not performing a global docking search and relying only on AlphaFold-multimer generated templates for initiating local docking. So, when the AF-multimer template is at a wrong interface, our local docking runs would exclusively sample near such an interface yielding incorrect predictions. Although this strategy worked

well for heterodimers, performing a fast, rigid, global docking search with the monomers (using ClusPro[14] or ReplicaDock2 rigid docking[6]) before local docking would have been useful. The global docking would serve as validation of the AF-multimer generated models, and in case different binding poses are predicted by global docking, we could sample alternative binding sites eventually improving our model prediction quality.

Multimers:

Multimeric targets comprised of either larger symmetric assemblies or heteromeric assemblies. For these targets, we combined our approaches for heterodimers and homomers to better predict multimer assemblies. First, for larger symmetric assemblies, such as A3 (targets 222 and 224), A6 (targets 201 and 219), A10 (target 230) and A16 symmetries (target 195), we used SymDock2 protocol to dock the monomer unit across a symmetry axis. The monomer prediction was obtained by AlphaFold and often refined using ab-initio modeling with Robetta. In this case, we did not use the AlphaFold-multimer template to initiate local docking runs but instead performed exhaustive SymDock2 searches. As a result, we were able to sample more CAPRI-acceptable or higher quality decoys for most of the targets. Figure 2B shows the A10 symmetry multimer modeled using SymDock2 (right). As evident in the figure, the AlphaFold prediction (left) contained highly disordered regions and ab-initio modeling helped to obtain a better monomeric subunit resulting in a high CAPRI-quality target.

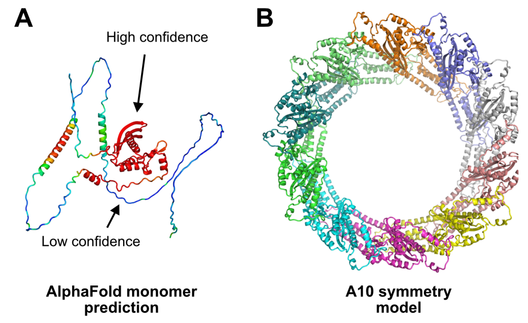

Figure 2: Target T230 (A10 symmetry). (A) AlphaFold prediction for the monomer highlights a high confidence core, with low confidence termini regions. We re-model the monomer using Robetta for docking predictions. (B) Our high CAPRI-quality docking prediction for T230.

For multimeric targets with heteromeric assemblies i.e., target 203 (A9B3), 220(A6B1), and 221 (A6B2), we first modelled the subunits that later associate to form the overall assembly. For target 203, we modelled A3B1 subunit and then obtained the overall assembly with SymDock2. Similar strategies were employed for the other targets resulting in better predictions for multimers. Multibody docking is still a challenging problem and developing tools exclusively to tackle multibody interactions, both homomers and heteromers, is a potential research avenue.

Antibody-Antigen targets:

This CASP15-CAPRI round incorporated 3 antibody-antigen targets and 5 nanobody-antigen targets. All the targets incorporating these interactions were in the difficult category owing to lower accuracy of AlphaFold and RoseTTAFold to predict antibody structures. Unlike the conventional heterodimeric docking, for these targets we employed our antibody structure prediction tools in conjunction with the binding poses from AlphaFold-multimer. Specifically, for the antibody/nanobody sequences, we predicted structures with IgFold, a multi-track deep learning model[10]. Next, we obtained the complex structures from AlphaFold-multimer, and replaced the antibody/nanobody in the AF2-generated complexes with our IgFold antibody/nanobody structures. These updated complexes, preserving the binding region from AlphaFold, but with antibody/nanobody models from IgFold, were then docked using RosettaDock4 and ReplicaDock2. Further, to focus sampling on CDR loops of the antibody/nanobody while docking,

we employed the directed induced-fit in ReplicaDock2 as described in Harmalkar et al.[6] This approach allows backbone sampling on known flexible regions in protein complexes to narrow the conformational search.

As with homodimers, an incorrect AF2 template adversely affected the performance. For targets 208 and 218, where the AF2 template led to a closer binding region with respect to the native, our docking methods yielded medium- and high-quality structures, respectively. However, for all other targets, our performance was underwhelming, often binding in the wrong epitopic region, and resulting in incorrect starting structures. Comparison with the top-performing group[15] for these targets (i.e., antigen with antibodies and nanobodies) indicates that excessive global sampling was crucial to identify better binding poses to refine and capture high-quality structures.

Successes and Failures

A summary of our performance is shown in Table 1. We participated in 44 targets of CASP15-CAPRI and achieved 6 highly accurate, 15 medium, and 7 acceptable quality targets (28 targets successful out of 44). Our performance for CASP15-CAPRI round highlights our improved performance in docking heterodimers and multimeric complexes. We consistently predicted medium and high-quality targets for those categories by incorporating backbone flexibility and refinement on AF2-generated templates. However, for antibody-antigen interactions and homodimers, where incorrect templates from AF2 were quite frequent, we had significantly worse predictions. By relying entirely on AF2-templates to initiate local docking, we limited our sampling search. From the results, it is evident that a global, rigid search can serve to either validate a binding pose predicted by AF2 or filter it as a false-positive interface. As with prior CASP-CAPRI challenges, diversifying our local docking binding poses remains a robust strategy to sample the diverse conformational landscapes of proteins and obtain a high-quality prediction.

Funding: This work was supported by National Institute of Health grant R35-GM141881. Computational resources were provided by the Advanced Research Computing at Hopkins (ARCH) core facility, supported by the National Science Foundation (NSF) grant number OAC1920103.

Conflict of Interest: JJG is an inventor of the IgFold technology mentioned in this report. Johns Hopkins University and JJG may be entitled to a portion of revenue received on commercial licensing of IgFold. JJG is an unpaid board member of the Rosetta Commons. Under institutional participation agreements between the University of Washington, acting on behalf of the Rosetta Commons, Johns Hopkins University may be entitled to a portion of revenue received on commercial licensing of Rosetta software including some programs described in this review. JJG has a financial interest in Cyrus Biotechnology. Cyrus Biotechnology distributes the Rosetta software, which may include methods described in this review. These arrangements have been reviewed and approved by the Johns Hopkins University in accordance with its conflict-of-interest policies.

References

1. Harmalkar, A. & Gray, J. J. Advances to tackle backbone flexibility in protein docking. *Curr. Opin. Struct. Biol.* **67**, 178–186 (2020).

2. Jumper, J., Evans, R., Pritzel, A., Green, T., Figurnov, M., Ronneberger, O.,

Tunyasuvunakool, K., Bates, R., Žídek, A., Potapenko, A., Bridgland, A., Meyer, C., Kohl, S. A. A., Ballard, A. J., Cowie, A., Romera-Paredes, B., Nikolov, S., Jain, R., Adler, J., *et al.* Highly accurate protein structure prediction with AlphaFold. *Nature* (2021) doi:10.1038/s41586-021-03819-2.

3. Evans, R., Neill, M., Pritzel, A., Antropova, N., Senior, A., Green, T., Ž\'\idek, A., Bates, R., Blackwell, S., Yim, J., Ronneberger, O., Bodenstein, S., Zielinski, M., Bridgland, A., Potapenko, A., Cowie, A., Tunyasuvunakool, K., Jain, R., Clancy, E., *et al.* Protein complex prediction with AlphaFold-Multimer. *bioRxiv* (2021) doi:10.1101/2021.10.04.463034.

4. Kryshtafovych, A., Schwede, T., Topf, M., Fidelis, K. & Moult, J. Critical assessment of methods of protein structure prediction (CASP)—Round XIV. *Proteins Struct. Funct. Bioinforma.* **89**, 1607–1617 (2021).

5. Lensink, M. F., Brysbaert, G., Mauri, T., Nadzirin, N., Velankar, S., Chaleil, R. A. G., Clarence, T., Bates, P. A., Kong, R., Liu, B., Yang, G., Liu, M., Shi, H., Lu, X., Chang, S., Roy, R. S., Quadir, F., Liu, J., Cheng, J., *et al.* Prediction of protein assemblies, the next frontier: The CASP14-CAPRI experiment . *Proteins Struct. Funct. Bioinforma.* 1–24 (2021) doi:10.1002/prot.26222.

6. Harmalkar, A., Mahajan, S. P. & Gray, J. J. Induced fit with replica exchange improves protein complex structure prediction. *PLOS Comput. Biol.* **18**, 1–21 (2022).

7. Marze, N. A., Roy Burman, S. S., Sheffler, W. & Gray, J. J. Efficient flexible backbone protein-protein docking for challenging targets. *Bioinformatics* **34**, 3461–3469 (2018).

8. Roy Burman, S. S., Yovanno, R. A. & Gray, J. J. Flexible Backbone Assembly and Refinement of Symmetrical Homomeric Complexes. *Structure* **27**, 1041-1051.e8 (2019).

9. Roy Burman, S. S., Yovanno, R. R. A. & Gray, J. J. Flexible Backbone Assembly and Refinement of Symmetrical Homomeric Complexes. *Structure* **27**, 1041--1051.e8 (2019).

10. Ruffolo, J. A. & Gray, J. J. Fast, accurate antibody structure prediction from deep learning on massive set of natural antibodies. *Biophys. J.* **121**, 155a-156a (2022).

11. Baek, M., Dimaio, F., Anishchenko, I., Dauparas, J., Ovchinnikov, S., Lee, G. R., Wang, J., Cong, Q., Kinch, L. N., Schaeffer, R. D., Millán, C., Park, H., Adams, C., Glassman, C. R., Degiovanni, A., Pereira, J. H., Rodrigues, A. V, Van Dijk, A. A., Ebrecht, A. C., *et al.* Accurate prediction of protein structures and interactions using a 3-track network. *bioRxiv* 2021.06.14.448402 (2021).

12. Song, Y., Dimaio, F., Wang, R. Y. R., Kim, D., Miles, C., Brunette, T., Thompson, J. & Baker, D. High-resolution comparative modeling with RosettaCM. *Structure* **21**, 1735–1742 (2013).

13. Raman, S., Vernon, R., Thompson, J., Tyka, M., Sadreyev, R., Pei, J., Kim, D., Kellogg, E., DiMaio, F., Lange, O., Kinch, L., Sheffler, W., Kim, B.-H., Das, R., Grishin, N. V & Baker, D. Structure prediction for CASP8 with all-atom refinement using Rosetta. *Proteins* **77 Suppl 9**, 89–99 (2009).

14. Bohnuud, T., Luo, L., Wodak, S. J., Bonvin, A. M. J. J., Weng, Z., Vajda, S., Schueler-Furman, O. & Kozakov, D. A benchmark testing ground for integrating homology modeling and protein docking. *Proteins Struct. Funct. Bioinforma.* **85**, 10–16 (2017).

15. Wallner, B. AFsample: Improving Multimer Prediction with AlphaFold using Aggressive Sampling. *bioRxiv* (2022) doi:10.1101/2022.12.20.521205.

Target	Target Difficulty	Stoichiometry	Classification	Interface	DockQ	Model Quality	CAPRI Rank
205	Difficult	A1B1	Antibody	1	0.05	incorrect	0
206	Difficult	A1B1	Antibody	1	0.08	incorrect	0
207	Difficult	A1B1	Antibody	1	0.01	incorrect	0
208	Difficult	A1B1	Antibody	1	0.83	high	***
216	Difficult	A1B1C1	Antibody	1	0.03	incorrect	0
217	Difficult	A1B1C1	Antibody	1	0.07	incorrect	0
218	Difficult	A1B1C1	Antibody	1	0.66	medium	**
191	Easy	A1B1	Heterodimer	1	0.61	medium	**
200	Easy	A1B1	Heterodimer	1	0.13	incorrect	0
210	Easy	A1B1	Heterodimer	1	0.88	high	***
202	Difficult	A1B1	Heterodimer	1	0.53	medium	**
212	Difficult	A1B1	Heterodimer	1	0.59	medium	**
192	Easy	A2	Homodimer	1	0.54	medium	**
193	Easy	A2	Homodimer	1	0.81	medium	**
198	Easy	A2	Homodimer	1	0.26	acceptable	*
199	Easy	A2	Homodimer	1	0.89	high	***
213	Easy	A2	Homodimer	1	0.16	incorrect	0
214	Easy	A2	Homodimer	1	0.27	incorrect	0
225	Easy	A2	Homodimer	1	0.53	medium	2
226	Easy	A2	Homodimer	1	0.03	incorrect	0
229	Easy	A2	Homodimer	1	0.04	incorrect	0
194	Difficult	A2	Homodimer	1	0.77	medium	**
197	Difficult	A2	Homodimer	1	0.24	incorrect	0
211	Difficult	A2	Homodimer	1	0.02	incorrect	0
201	Easy	A6	Multimer	1	0.91	high	***
203	Easy	A9B3	Multimer	1	0.85	high	***
203	Easy	A9B3	Multimer	2	0.44	acceptable	*
203	Easy	A9B3	Multimer	3	0.44	acceptable	*
203	Easy	A9B3	Multimer	4	0.40	incorrect	0

219	Easy	A6	Multimer	1	0.51	medium	**
219	Easy	A6	Multimer	2	0.70	medium	**
219	Easy	A6	Multimer	3	0.49	acceptable	*
220	Easy	A6B1	Multimer	1	0.51	medium	**
220	Easy	A6B1	Multimer	2	0.53	acceptable	*
220	Easy	A6B1	Multimer	3	0.58	medium	**
220	Easy	A6B1	Multimer	4	0.60	medium	**
221	Easy	A6B2	Multimer	1	0.50	medium	**
221	Easy	A6B2	Multimer	2	0.52	acceptable	*
221	Easy	A6B2	Multimer	3	0.51	acceptable	*
221	Easy	A6B2	Multimer	4	0.47	medium	**
230	Easy	A10	Multimer	1	0.84	high	***
195	Difficult	A16	Multimer	1	0.07	incorrect	0
222	Difficult	A3	Multimer	1	0.19	incorrect	0
224	Difficult	A3	Multimer	1	0.02	incorrect	0

Table 1: Gray Lab performance on CASP15-CAPRI targets. Targets are classified based on 4 categories: Antibody-antigen targets, heterodimers, homodimers and multimers.

Huang

Integrating AlphaFold-Multimer and HDOCK for CASP15-CAPRI

Hao Li[1], Peicong Lin[1], Jiahua He[1], Huanyu Tao[1], and Sheng-You Huang[1*]

[1]*School of Physics, Huazhong University of Science and Technology, Wuhan, China 430074*

*Email: huangsy@hust.edu.cn

Specifically, for most targets, we used AlphaFold-Multimer (v2.2.0) to construct the complex structures [1], and the individual subunits were separated from the complex structures. After separating individual subunits from the 'ranked 0.pdb' built by AlphaFold-Multimer, we performed ab initio docking using HDOCK (for asymmetric complexes) and HSYMDOCK (for symmetric complexes) based on these subunits [2, 3]. Based on the confidence of the models produced by AlphaFold-Multimer, we submitted the 'ranked 0.pdb' of AlphaFold-Multimer and the top 99 docking models to the CAPRI for most targets. Since most of the targets are separated and re-docked based on the 'ranked_0.pdb' of AlphaFold-Multimer, most of our results are also consistent with AF2-Multimer (the off-the-bench AlphaFold-Multimer modeling as submitted by the Elofsson group). As shown in Figure 1, when considering a model of acceptable quality as a success, for all 37 targets, 22 targets (59.5%) were successfully predicted by our group and AF2-Multiemr, while both our group and AF2-Multiemr failed on the other 9 targets (24.3%). From the interface level, for all 55 interfaces, 34 interfaces (61.8%) were successfully predicted by our group and AF2-Multiemr, while both our group and AF2-Multiemr failed on the other 10 interfaces (18.2%).

Next, we focus on the targets whose results differ between AF2-Multimer and our group. AF2-Multimer succeeded on T195 and T198, but our group failed. The 16-mer T195 has a total of 4608 residues. Due to the limitation of computing resources, we have no way to use AlphaFold-Multimer to directly build complex structures. Instead, we used AlphaFold2 to build a monomer structure, and then provide it to HSYMDOCK for C16 symmetry docking. For T198, although the 'ranked_0.pdb' of AF2-Multimer failed, the 'ranked_1.pdb' succeeded.

For T203, due to the poor quality of the 12-mer models (A9B3) directly built by AlphaFold-Multimer, we first used AlphaFold-Multimer to build a tetramer model (A3B1), and then imported this tetramer into HSYMDOCK for C3 symmetry docking. Thanks to the above operations, we succeeded in 2 more interfaces on T203 than AF2-Multimer that directly built 12-mer models. The decamer T204 has a total of 4592 residues. Due to the limitation of computing resources, we have no way to use AlphaFold-Multimer to directly build the decamer models. As an alternative, we first used AlphaFold-Multimer to build two pentamers ABCDE and FGHIJ. After analyzing the structures of these two pentamers, we have the following three speculations: (1) ABCDEF forms a helical hexamer. (2) IJ forms a dimer and is located above the ABCDEF hexamer. (3) GH forms a dimer and locates above the IJ dimer. According to the above speculations, we used AlphaFold-Multimer to build the ABCDEFIJ octamer and the GHIJ tetramer, and then superimposed the GHIJ tetramer on the octamer according to IJ to obtain the decamer. Limited by computational resources, we deleted some residues at the ends of some chains when building the ABCDEFIJ octamer. Due to the above operations, we succeeded in three interfaces on T204 where AF2-Multimer failed. For three dimers (T206, T213 and T214) that AF2-Multimer failed, we all obtained medium quality models thanks to the re-docking of HDOCK.

In the scoring challenge stage, the knowledge-based scoring function ITScorePP [4] was used to evaluate the models submitted by CAPRI participants. There are more than two subunits in many targets, which leads to multiple interaction interfaces. We scored all subunit pairs using ITScorePP and then added up the individual scores to obtain the overall scores for the protein complexes. Next, we ranked all models according to the total scores of the complexes. For the top-ranked models, those structures were manually inspected. After removing the unreasonable structures, we submitted the 'ranked 0.pdb' of AlphaFold-Multimer and the top 4 scoring models to the CAPRI for the scoring experiment.

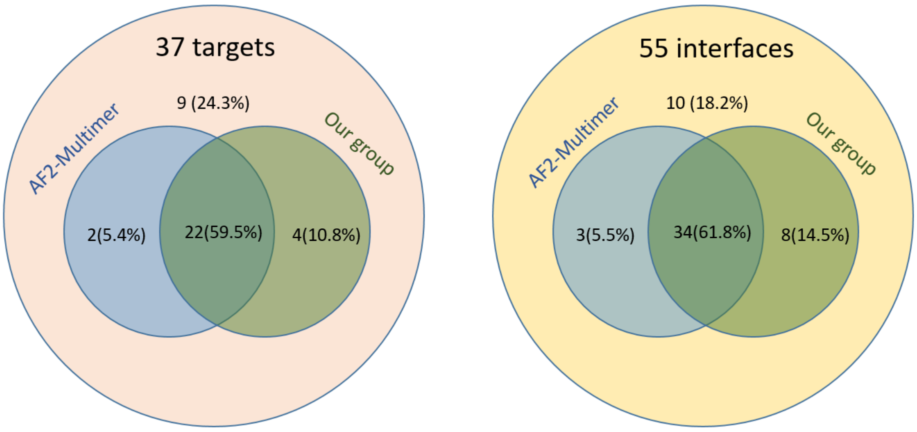

Figure 1: The successful predictions by AF2-Multimer, our docking approach, and both methods for the 37 assemble targets and their 55 protein-protein interfaces.

References:

1. Evans R, O'Neill M, Pritzel A, et al. Protein complex prediction with AlphaFold-Multimer. BioRxiv, 2022: 2021.10.04.463034.
2. Yan Y, Tao H, He J, Huang SY. The HDOCK server for integrated protein-protein docking. Nat Protoc. 2020; 15(5):1829-1852.
3. Yan Y, Tao H, Huang S-Y. HSYMDOCK: a docking web server for predicting the structure of protein homo-oligomers with Cn or Dn symmetry. Nucleic Acids Res. 2018;46(W1):W423-W431.
4. Huang S-Y, Zou X. An iterative knowledge-based scoring function for protein-protein recognition. Proteins. 2008; 72:557-579.

Funding:

This work was supported by the National Natural Science Foundation of China to S.H. (Grant No: 62072199 and 32161133002).

The adventures of LightDock in the CASP15/CAPRI contest

Jorge Roel-Touris[1], Brian Jiménez-García[2,*]

1 Protein Design and Modeling Lab, Department of Structural Biology, Molecular Biology Institute of Barcelona (IBMB-CSIC), Baldiri Reixac 15, 08028 Barcelona, Spain
2 Zymvol Biomodeling, Pau Claris 94 3B, 08010, Barcelona, Spain
* Corresponding author <bjimenez@zymvol.com>

Abstract

The LightDock team participated in the human predictors category, and contributed models for the last joint CASP15/CAPRI challenge. Specifically, we submitted predictions for the T196 and T203 targets (symmetric oligomers), T200 (membrane-associated assembly), and T205 and T206 (nanobody-mediated interactions); covering exclusively protein-protein complexes. Taking advantage of the tremendous advances in computational methods, we employed a plethora of different techniques to generate, diversify and select the models, with LightDock [1] for the modeling of interactions. LightDock is an artificial intelligence-powered software that integrates the *glowworm swarm optimization* algorithm as an optimization engine for the modeling of protein-protein (including peptides) and protein-nucleic acids.

For T196 and T203 targets, we expanded the LightDock software to account for cyclic symmetries (https://github.com/lightdock/lightdock/tree/sym). This new version of the software optimizes the general position and orientation of the monomers while also being capable of modeling backbone flexibility via anisotropic network model (ANM). For both targets, the selected symmetric oligomers were relaxed in cycles of decreasing temperature with the FastRelax Mover included in Rosetta [2,3] and implemented in PyRosetta [4]. While for T196 submitted models were selected according to DFIRE [5] score, for T203 we used a composite ranking encompassing DFIRE and Rosetta (ref_2015) [3] scores, followed by manual inspection.

As T200 involved a membrane protein, we embedded the AlphaFold2 [6] generated receptor into an artificial bead-based membrane using the limited information available at UNIPROT. Then, we performed the dedicated and automatic membrane modeling mode of the LightDock Server [7] to generate the top predicted and minimized with OpenMM [8] models. In this case, selection was made purely based on DFIRE scores ranking provided by the LightDock-Server.

Targets T205 and T206 involved the interactions of two nanobodies with the CNPase. For T205, we used AlphaFold2 with multiple sequence alignments of varying depth to diversify the conformation of the H3 loop. In the case of target T206, we used RoseTTAFold [9] to model the nanobody structure. In both cases, we used LightDock and the H3 sequence as residue

restraints to drive the simulation. After a short minimization with OpenMM, models were selected according to their score.

Funding
J.R-T. acknowledges funding from the European Molecular Biology Organization (EMBO) [ALTF 145-2021]; BJ-G. is employed by Zymvol Biomodeling on a project which received funding from the European Union’s Horizon 2020 research and innovation programme under Marie Skłodowska-Curie grant agreement No. 801342 (Tecniospring INDUSTRY) and the Government of Catalonia’s Agency for Business Competitiveness (ACCIÓ).

References
[1] Jiménez-García, B., Roel-Touris, J., Romero-Durana, M., Vidal, M., Jiménez-González, D., & Fernández-Recio, J. (2018). LightDock: a new multi-scale approach to protein–protein docking. Bioinformatics, 34(1), 49-55.
[2] Das, R., & Baker, D. (2008). Macromolecular modeling with rosetta. Annu. Rev. Biochem., 77, 363-382.
[3] Alford, R. F., Leaver-Fay, A., Jeliazkov, J. R., O’Meara, M. J., DiMaio, F. P., Park, H., ... & Gray, J. J. (2017). The Rosetta all-atom energy function for macromolecular modeling and design. Journal of chemical theory and computation, 13(6), 3031-3048.
[4] Chaudhury, S., Lyskov, S., & Gray, J. J. (2010). PyRosetta: a script-based interface for implementing molecular modeling algorithms using Rosetta. Bioinformatics, 26(5), 689-691.
[5] Zhou, H., & Zhou, Y. (2002). Distance‐scaled, finite ideal‐gas reference state improves structure‐derived potentials of mean force for structure selection and stability prediction. Protein science, 11(11), 2714-2726.
[6] Jumper, J., Evans, R., Pritzel, A., Green, T., Figurnov, M., Ronneberger, O., ... & Hassabis, D. (2021). Highly accurate protein structure prediction with AlphaFold. Nature, 596(7873), 583-589.
[7] Jiménez-García, B., Roel-Touris, J., & Barradas Bautista, D. (2023). The LightDock Server: Artificial Intelligence-powered modeling of macromolecular interactions.
[8] Eastman, P., Swails, J., Chodera, J. D., McGibbon, R. T., Zhao, Y., Beauchamp, K. A., ... & Pande, V. S. (2017). OpenMM 7: Rapid development of high performance algorithms for molecular dynamics. PLoS computational biology, 13(7), e1005659.
[9] Baek, M., DiMaio, F., Anishchenko, I., Dauparas, J., Ovchinnikov, S., Lee, G. R., ... & Baker, D. (2021). Accurate prediction of protein structures and interactions using a three-track neural network. Science, 373(6557), 871-876.

Kiharalab

Integrated Modeling Protocol for Human and Server Prediction of Macromolecular Assemblies

C.W. Christoffer[1], A.J. Jain[2], Y. Kagaya[2], H. Kannan[2,3], T. Nakamura[2], G. Terashi[2], J.C. Verburgt[2], Y. Zhang[1], Z. Zhang[1], Hayato Fujita[4], Masakazu Sekijima[4], and D. Kihara[1,2]

[1] - Department of Computer Science, Purdue University, West Lafayette, IN, USA, [2] - Department of Biological Sciences, Purdue University, West Lafayette, IN, USA, [3] - Department of Biotechnology, Bhupat and Jyoti Mehta School of Biosciences, Indian Institute of Technology Madras, Chennai 600036, India, [4] Department of Computer Science, Tokyo Institute of Technology, Yokohama, 226-8501, Japan

Correspondence: dkihara@purdue.edu

Key: *Auto:N; CASP_serv:Y; Templ:Y; MSA:Y.MetaG; Fragm:N; Cont:N; Dist:Y; Tors:Y; DeepL:Y; EMA:Y; MD:Y*

Our group participated in both the prediction and scoring stages for complex targets.

Methods

Single-chain subunit protein structure prediction: We generated MSAs for the deep learning network inputs using the following two-step strategy. For a query sequence, we first performed 3 iterations of search on UniRef30 (2021_03) and UniRef_90[1] (2022_01) with hhblits[2] and jackhmmer[3], respectively, to obtain two MSAs. These two MSAs were combined and then used as input profiles for the second step. In the second step, the MGnify[4] (2019_05), Metaclust_nr[5] (2018_06), and BFD (Latest) databases were used. The search was performed with only a single iteration with jackhmmer and hhblits. In addition, the MSAs used in ColabFold[6] were computed independently using mmseqs2[7] and their corresponding database. All resulting MSAs from both first and second step were simply concatenated and then used as input for the network.

In addition to predictions with the AlphaFold2[8] parameters that were made available by DeepMind, we trained a separate in-house structure module for single-chain prediction. The inputs for this structure module were the pair and single representations generated from the regular AlphaFold inference pipeline. The training dataset contained around 30k sequences with a 25% sequence similarity cutoff. Single chain targets with 25% sequence similarity to the training set were used as a validation set. We used all losses along with their corresponding weight hyperparameters as claimed in the AlphaFold paper, except for the MSA and distogram losses, as they were not applicable to the structure module. Using both original AlphaFold2 and the model we trained ourselves, we had 10 predictions available in total for each unique sequence entity/chain of each target. Predicted structures were ranked based on their mean predicted pLDDT values. Literature information was used for model selection and modifying models when available. When necessary, manual modification of input MSAs as well as of the resulting structure models were also performed.

Protein complex assembly prediction: For whole protein complexes, MSAs generated as above were used to generate AlphaFold models. Models were generated using AlphaFold-Multimer and other existing server implementations. Models from different sources were ranked by calibrated model quality estimates. For large prediction targets where reasonable models could not be generated in a single inference, models were broken down, either by reducing stoichiometry or separating plausible domains, and combined using minimization by phenix.geometry_minimization[9]. For higher-order

homomeric targets where AlphaFold-Multimer still failed to generate reasonable models, we used SAM[10] for symmetrical docking. We first ran AlphaFold-Multimer on repeated target sequences and obtained subunit structures, which were input to SAM to produce complexes with various symmetries. We essentially ranked those structures using the score output by SAM. Manual modifications of models and input MSAs were performed when necessary.

Automatic complex assembly prediction server: We used an automatic protein structure prediction pipeline to submit full complexes as LZerD[12]. The sequence search method described above was used to generate input to AlphaFold-Multimer. The top 10 models by predicted pLDDT were then submitted. In cases where GPU memory was exhausted before models were output, the size or stoichiometry of the input was reduced until models were generated.

Automatic complex assembly scoring server: We used an automatic protein complex scoring pipeline to submit scoring models as LZerD. All available scoring models were clustered with a cutoff of 4 Å root-mean-square deviation (RMSD) to produce a less-redundant pool of models. These models were then ranked according their ranksum score[11], calculated from multiple knowledge-based scoring functions[13-15]. The top 10 models were then submitted.

Modeling successes and failures: For T226, a complex from murine astrovirus, our group submitted a higher-quality top-1 model than both the top-ranked Venclovas group and the AF2-MULTIMER baseline. Here, we used our AF2-derived pipeline with an MSA generated from an NCBI database of virus proteins[16] and generated an acceptable top-1 model with an I-RMSD of 2.2 Å, an L-RMSD of 7.1, and an f_{na} of 0.28. For T205, a CNPase-nanobody complex where top-1 models from the baseline and top group were both incorrect, our manual modeling yielded an acceptable top-1 model with an I-RMSD of 2.5 Å, an L-RMSD of 9.9, and an f_{na} of 0.36. On the other hand, there were three targets where we failed to submit a top-1 model of at least acceptable quality, although the top-1 models from the baseline or the top group were of at least acceptable quality. T195, a high-order stomatin complex with 4608 residues, was not handled well by our available GPU capacity, exhausting the GPU memory. Manual modeling of this complex was unable to acceptably reproduce the interface. T206 and T208, both CNPase-nanobody targets, were not compatible with the coevolution information approach in our deep learning pipeline, and manual intervention did not yield properly placed nanobodies.

We note that despite this, our group was one of only four groups to overall produce top-1 models with significantly better (Wilcoxon test, $p < 0.05$, no discarding of negative Z-scores) DockQ scores than the baseline AF2-MULTIMER, alongside Venclovas, Wei_Zheng, and Jianyi_Yang. When considering significance against our models, only Wei_Zheng produced significantly higher-DockQ top-1 models than ours, which suggest that our group is the second next to Wei_Zheng or among the second ranked teams following Wen_Zheng.

Acknowledgements

We are grateful for ITaP Research Computing at Purdue University for providing us additional computational resources for this project. This work is partially supported by the National Institutes of Health (R01GM133840, R01GM123055), the National Science Foundation (CMMI1825941, MCB1925643, DBI2146026, IIS2211598, and DBI2003635). C.C. and J.V. were supported by a National Institute of General Medical Sciences- funded predoctoral fellowship to C.C. and J.V. (T32 GM132024).

References

1. Suzek, B. E., Wang, Y., Huang, H., McGarvey, P. B., and Wu, C. H. (2015). UniRef clusters: A comprehensive and scalable alternative for improving sequence similarity searches. Bioinformatics 31, 926–932. doi: 10.1093/bioinformatics/btu739.
2. Mistry, J., Finn, R. D., Eddy, S. R., Bateman, A., and Punta, M. (2013). Challenges in homology search: HMMER3 and convergent evolution of coiled-coil regions. Nucleic Acids Res 41, e121–e121. doi: 10.1093/NAR/GKT263.
3. Steinegger, M., Meier, M., Mirdita, M., Vöhringer, H., Haunsberger, S. J., and Söding, J. (2019). HH-suite3 for fast remote homology detection and deep protein annotation. BMC Bioinformatics 20, 1–15. doi: 10.1186/s12859-019-3019-7.
4. Mitchell, A. L., Almeida, A., Beracochea, M., Boland, M., Burgin, J., Cochrane, G., et al. (2020). MGnify: the microbiome analysis resource in 2020. Nucleic Acids Res 48, D570–D578. doi: 10.1093/NAR/GKZ1035.
5. Steinegger, M., and Söding, J. (2018). Clustering huge protein sequence sets in linear time. Nature Communications 2018 9:1 9, 1–8. doi: 10.1038/s41467-018-04964-5.
6. Mirdita, M., Schütze, K., Moriwaki, Y., Heo, L., Ovchinnikov, S., and Steinegger, M. (2022). ColabFold: making protein folding accessible to all. Nature Methods 2022 19:6 19, 679–682. doi: 10.1038/s41592-022-01488-1.
7. Steinegger, M., and Söding, J. (2017). MMseqs2 enables sensitive protein sequence searching for the analysis of massive data sets. Nature Biotechnology 2017 35:11 35, 1026–1028. doi: 10.1038/nbt.3988.
8. Jumper,J. et al. (2021) Highly accurate protein structure prediction with AlphaFold. Nature, 596, 583–589.
9. Liebschner,D. et al. (2019) Macromolecular structure determination using X-rays, neutrons and electrons: recent developments in Phenix. Acta Cryst Sect D Struct Biol, 75, 861–877.
10. D.W. Ritchie and S. Grudinin (2016). Spherical Polar Fourier Assembly of Protein Complexes with Arbitrary Point Group Symmetry. Journal of Applied Crystallography, 49(1), 158-167.
11. Christoffer,C. et al. (2019) Performance and enhancement of the LZerD protein assembly pipeline in CAPRI 38- 46. Proteins, 88, 948–961.
12. Christoffer,C. et al. (2021) LZerD Protein-Protein Docking Webserver Enhanced With de novo Structure Prediction. Front. Mol. Biosci., 8.
13. Zhou,H., Zhou,Y. (2002) Distance-scaled, finite ideal-gas reference state improves structure-derived potentials of mean force for structure selection and stability prediction. Protein Sci., 11(11):2714-2726.
14. Zhou,H., Skolnick,J. (2011) GOAP: a generalized orientation-dependent, all-atom statistical potential for protein structure prediction. Biophys J., 101(8):2043-2052.
15. Huang,S.Y., Zou,X. (2011) Statistical mechanics-based method to extract atomic distance-dependent potentials from protein structures. Proteins, 79(9):2648-2661.
16. Hatcher,E.L., Zhdanov,S.A., Bao,Y., Blinkova,O., Nawrocki,E.P., Ostapchuck,Y., Schaffer,A.A., Brister,J.R. (2017) Virus Variation Resource – improved response to emergent viral outbreaks. Nucleic Acids Res., 45(D1):D482-D490.

ClusPro and Kozakov/Vajda

Prediction of protein assemblies and ligand binding modes using a combination of ClusPro, Alphafold, and template-based modeling

Omeir Khan[1], Sergei Kotelnikov[2], Usman Ghani[1], Dzmitry Padhorny[2], Dmitri Beglov[1], Sandor Vajda[1], Dima Kozakov[1,2]
1 - Boston University, 2 - Stony Brook University
midas@laufercenter.org; vajda@bu.edu

In the latest CASP-CAPRI round, our group generated models of protein assemblies using a combination of Alphafold-Multimer (AFM), Alphafold2 (AF2), and docking using the ClusPro webserver.[1-3] Here we describe the methods used for both generating and ranking ensembles of protein—protein complexes.

Methods:

Model Generation:

Our group used a two-stage methodology, first generating an ensemble of initial models that were subsequently provided to Alphafold-Multimer (AFM) as templates for obtaining "refined" structures of the target complex. The protocols used for initial model generation are described below.

Docking Alphafold2 Models with Cluspro (AF2+ClusPro): The structure of each chain in the assembly was independently predicted using the pTM parameter set of AF2. These single-chain predictions were then ranked by the predicted LDDT (pLDDT). The top ranked model for each chain was selected, and low confidence residues (pLDDT < 0.50) were cut from the termini. The trimmed models were docked using the ClusPro web server.[3] All models generated using the "Electrostatic-favored" coefficient set were downloaded and retained for further processing. For antibody and nanobody targets, ClusPro was run in antibody mode.[4] For homomeric complexes additional models were generated using ClusPro's multimer docking mode.

Multimer Prediction with Templates (AFM-Temp): An unmodified version of AFM was used to generate 25 models of the target complex. For template searching, the maximum template release date was set to May 14th, 2022.

Multimer Prediction without Templates (AFM-NoTemp): The MMseqs2 API was used to generate multiple sequence alignments (MSAs) for each subunit of the complex[5]. The pTM parameter set was then used to generate 5 models of the target assembly. No templates were used in the generation of these models.

Template-based modeling with ClusPro-TBM (TBM): The ClusPro template-based modeling functionality was used to select templates for a given target complex. The sequences and stoichiometry of the assembly were given as inputs. Templates were found using a local installation of HHPred, from which the HHblits and HHsearch commands were used to search the uniclust30 and pdb100 databases.[6-8] Search results were selected with > 20% probability and > 20% query sequence coverage. If the stoichiometry of a template matched that of the target, the template was retained and used for model generation in the next step.

Model Refinement:

In the refinement step models generated using the aforementioned approaches were provided as template structures to Alphafold-Multimer. The refinement stage was dual purpose, as it was used both for improving the quality of template models and producing a confidence score for each model, which was then used for ranking. For refinement, MSAs were prepared for each subunit using the AFMMseqs2 API.[5]

Results:

Performance Highlights:

Two successes highlighted here are targets T1161/T214 and H1166/T216. For target T214, our most accurate models were produced by the *AFM-NoTemp* and *TBM* protocols. For target T216, top ranked predictions were generated with the *AF2+ClusPro* and *TBM* approaches, resulting in a model with an "Acceptable" DockQ score. The highlighted results underscore the potential advantages of generating models by a variety of methods, since refining models produced by docking or template-based modeling with AFM can produce higher confidence models than those generated with Alphafold alone.

Encountered difficulties:

The generation of accurate antibody–antigen complexes remains a challenge in computational protein modeling. We note that although predicted antibody binding modes were inaccurate, the protocol was capable of correctly modelling the epitopes in targets H1166/T216 and H1168/T218. For these targets, inaccuracies stemmed from discrepancies in CDR loop conformations that ultimately resulted in lower quality models. Therefore, future efforts directed towards developing more robust protocols for accurately modelling CDR loops may bolster the capabilities of prediction algorithms for antibody–antigen complexes.

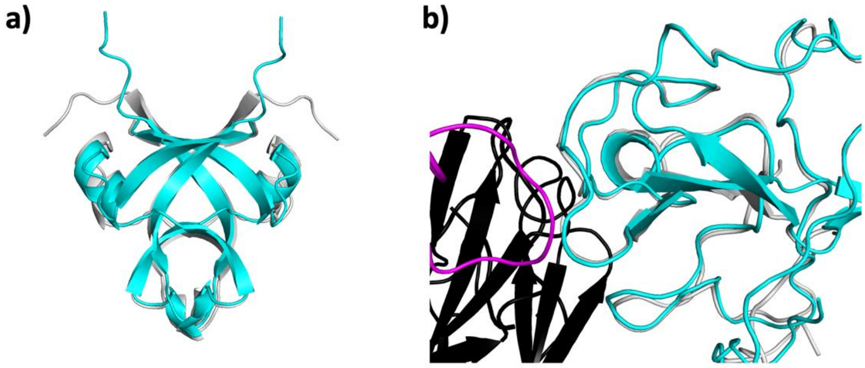

Figure 1. Models for antibody targets H1161/T214 and H1166/T216. a) The most accurate model (cyan) for T214 aligned to the native structure (grey) b.) A view of the epitope with the antigen model (cyan) aligned to the native antigen (grey). The predicted antibody (magenta) is shown to align poorly with the native antibody (black) despite interacting at the correct interface.

Availability:
ClusPro is available as a webserver that is free for academic and governmental use.

References

1. Evans,R., O'Neill,M., Pritzel,A., Antropova,N., Senior,A., Green,T., Zidek,A., Bates,R., Blackwell,S., Yim,J., Ronnenberger,O., Bodenstein,S., Zielinski,M., Bridgland,A., Potapenko,A., Cowie,A., Tunyasuvunakool,K., Jain,R., Clancy,E., Kohli,P., Jumper,J. & Hassabis,D. (2022). Protein complex predition with Alphafold-Multimer. Preprint at *Biorxiv*.
2. Jumper, J., Evans,R., Pritzel,A., Green,T., Figurnov,M., Ronnenberger,O., Tunyasuvunakool,K., Bates,R., Zidek,A., Potapenko,A., Bridgland,A., Meyer,C., Kohl,S.A.A., Ballard,A.J., Cowie,A., Romera-Paredes,B., Nikolov,S., Jain,R., Adler,J., Back,T., Petersen,S., Reiman,D., Clancy,E., Zielinski,M., Steinegger,M., Pacholska,M., Berghammer,T., Bodenstein,S., Silver,D., Vinyals,O., Senior,A.W., Kavukcuoglu,K., Kogli,P. & Hassabis,D. (2021). Highly accurate protein structure prediction with AlphaFold. *Nature.* **596**, 583-589
3. Kozakov,D., Hall,D.R., Xia,B., Porter,K.A., Padhorny,D., Yueh,C., Beglov,D. & Vajda,S (2017). The ClusPro web server for protein-protein docking. *Nat. Protoc.* **12,** 255-278
4. Brenke,R., Hall,D.R., Chuang,G-Y., Comeau,S.R., Bohnuud,T., Beglov,D., Schuler-Furman,O., Vajda,S., & Kozakov,D. (2012). Application of asymmetric statistical potentials to antibody-protein docking. *Bioinformatics*. **28**, 2608-2614
5. Steinegger,M. & Soding,J. (2017). MMseqs2 enabled sensitive protein sequence searching for the analysis of massive data sets. *Nat Biotechnol*. **35**, 1026-1028
6. Mirdita,M.,von den Driesch,L., Galiez,C., Martin,M.J., Soding,J. & Steinegger,M. (2016) Uniclust databases of clustered and deeply anntotated protein sequences and alignments. *Nucleic Acids Res.* **45**, D170-D176
7. Hildebrand,A., Remmert,M., Biegert,A. & Soding,J., F (2009). Fast and accurate automatic structure prediction with Hhpred. *Proteins*. **77**, 128-132
8. Padhorny,D., Porter,K.A., Ignatov,M., Alekseenko,A., Beglov,D., Kotelnikov,S., Ashizawa,R., Desta,I., Alam,N., Sun,Z., Brini,E., Dill,K., Schueler-Furman,O., Vajda, S. & Kozakov, D. (2020) Cluspro in rounds 38-45 of CAPRI: Toward combining template-based methods with free docking. *Proteins*. **88**, 1082-1090

Surendra S. Negi
Sealy Center for Structural Biology and Molecular Biophysics
University of Texas Medical Branch
310 University Boulevard, Galveston, Texas 77555-0857

Abstract:

Rapid advancement in the development of new artificial intelligence methods provides a new opportunity to understand how proteins fold and form large molecular assemblies. In the current CASP15/CAPRI competition, we have used LocalColabFold (https://github.com/YoshitakaMo/localcolabfold) (1), a fork of AlphaFold (2) running locally on a Linux computer to generate initial set of docked protein complexes. We found that LocalColabFold provides faster results because the sequence alignments are generated using the fast MMseqs2 method (3). Additionally, we used the traditional homology modeling approach to model the protein structures and compared them with structures obtained from LocalColabFold method. Major differences were observed in the loop region, where AlphaFold model the structures with a high pLDDT score (4). For all of our model structures, we used templates (to generate a model structure from latest available templates) and amber (energy minimized structure) option in the LocalColabFold before submitting the final structure to CASP15/CAPRI prediction.

References:

1. Mirdita, M., Schütze, K., Moriwaki, Y., Heo, L., Ovchinnikov, S., and Steinegger, M. 2022. ColabFold: making protein folding accessible to all. *Nature Methods* 19:679-682.
2. Jumper, J., Evans, R., Pritzel, A., Green, T., Figurnov, M., Ronneberger, O., Tunyasuvunakool, K., Bates, R., Žídek, A., Potapenko, A., et al. 2021. Highly accurate protein structure prediction with AlphaFold. *Nature* 596:583-589.
3. Steinegger, M., and Söding, J. 2017. MMseqs2 enables sensitive protein sequence searching for the analysis of massive data sets. *Nature Biotechnology* 35:1026-1028.
4. Mariani, V., Biasini, M., Barbato, A., and Schwede, T. 2013. lDDT: a local superposition-free score for comparing protein structures and models using distance difference tests. *Bioinformatics* 29:2722-2728.

17 Oliva

Models ranking by CONSRANK and Clust-CONSRANK

Tiziana Ricciardelli[1], Didier Barradas-Bautista[1], Zhen Cao[1], Mohit Chawla[1], Luigi Cavallo[1] and Romina Oliva[2*]

[1] King Abdullah University of Science and Technology, Saudi Arabia
[2] University of Naples "Parthenope", Italy

E-mail:
tiziana.ricciardelli@kaust.edu.sa
didier.barradasbautista@kaust.edu.sa
zhen.cao@kaust.edu.sa
mohit.chawla@kaust.edu.sa
luigi.cavallo@kaust.edu.sa
romina.oliva@uniparthenope.it*

Scoring function/scheme used

We submitted scoring predictions for all the 36 targets (and 55 interfaces) assessed in the scoring experiment. As a preliminary step, possible templates for modeling the complexes were searched by HHPRED [1], to start having an idea about the prediction difficulty of each target. All the targets (with the exception of T204, see below) were then scored with our tools CONSRANK (CONSensus RANKing) and Clust-CONSRANK. CONSRANK is a pure *consensus* method for the ranking of docking decoys we introduced in the field years ago [2-3]. It ranks models based on their ability to match the most frequent inter-residue contacts in the ensemble of decoys they belong to, and is also available as a user-friendly web server [4]. Clust-CONSRANK is a CONSRANK implementation we developed to introduce a contact-based clustering of the models as a preliminary step of the scoring process [5]. For the clustering, as in previous CAPRI rounds, a threshold was set on the number of clusters (and not on the models similarity), as 1/10 of the total number of models per target.

For each target, the ≈25-30 models with the highest CONSRANK score and the 2-3 top ranked models for the 15 most populated clusters from the Clust-CONSRANK output were compared and further analyzed with CoCoMaps [6], a web tool we specifically developed for the analysis of the interface in macromolecular complexes.

Differently from previous CAPRI rounds, we observed, as a trend, a quite clear *consensus* emerging from the decoy ensembles for most of the targets. As a consequence, the binding solutions resulting from the clustering analyses were often observed to be similar. Therefore, in several cases we selected for submission solutions from more clusters, which featured specific details regarding either the interface or the predicted structure for interacting proteins, although not representing substantially alternative binding modes. CASP15-CAPRI targets were thus a useful benchmark for us to test the capability of our contact-based clustering approach of identifying higher quality decoys in sets of similar docking solutions. Models showing a large number of clashes were removed from the selection. Ranking of the selected models from the top-1 to the top-10 position was mainly based on results of the interface analysis. Models exhibiting a more extended contact network and interface area were generally top ranked.

1

As CONSRANK could not be applied to the 10-mer (stoichiometry: A1B1C1D1E1F1G2I1J1) target T204, a completely different approach, based on molecular dynamics simulations carried out with GROMACS [7], was used for it.

Short description of successes and failures

In the top-5 ranking, we had correct models for 26 out of the total 36 targets, on a par with the best performing scoring group in this CAPRI round, and for 45 out of the total 55 interfaces, i.e. one more than them. Staying on the targets, we had at least a solution of high or medium quality in the top-5 positions for 20 of them (7 and 13, respectively), and at least an acceptable solution for the remaining 6 ones. Our performance in the top-1 ranking was also satisfactory, as we had a correct model ranked 1st for the majority of targets (19 over 36), for 16 of them being of high or medium quality (5 and 11, respectively).

Overall, we had correct solutions for all the large assembly targets and our successful targets included two nanobody complexes (T205 and T208). The ten targets on which we failed were homo/heterodimers either classified as difficult from the beginning by CAPRI assessors (T197, T206, T207, T209, T216, T217, T224) or initially classified as easy and then found to be "surprisingly difficult" (T213, T214 and T229). It is worth noticing that especially for the targets T197, T213 and T214, we observed a clear consensus in terms of inter-molecular contacts emerging from the scoring ensembles. The maximum CONSRANK score for them indeed exceeded the value of 0.3, reflecting a common contacts pattern for a significant fraction of models to be scored. While such values were, in our previous experience in CAPRI, high enough for us to confidently top rank the models representative of the overall ensembles, they led to failure here. For T213 and T214, experimental structures have not been released yet, for us to check. As for T197, our post-analyses based on the now available experimental structure of the complex (PDB ID: 7TIL) show that the high consensus we observed corresponded to roughly 40 mutual contacts between ≈30 residues (211 to 239) of the protein C-terminal domain, which were correctly present in over 20% of all the models to be scored. However, the rest of the interface, involving other regions of the protein (which forms an intertwined homodimer) was less conserved and not correctly predicted in the models we selected. A near-native model was actually present in our submission (from Clust-CONSRANK cluster 2) but at the ranking position 9, not assessed in the CASP/CAPRI experiment.

Funding

This research was funded by the KAUST baseline research funding (to LC) and by MIUR-FFABR "Fondo per il Finanziamento Attività Base di Ricerca" (to RO).

1. Söding J, Biegert A, Lupas AN. (2005) *Nucleic Acids Res.* **33**:W244-8.
2. Oliva R, Vangone A, Cavallo L. (2013) *Proteins* **81**, 1571-1584.
3. Vangone A, Oliva R, Cavallo L. (2013) *Proteins* **81**, 2210-2220.
4. Chermak E, Petta A, Serra L, Vangone A, Scarano V, Cavallo L, Oliva R. (2015) *Bioinformatics* **31**, 1481-3.
5. Chermak, E, De Donato, R, Lensink MF, Petta, A, Serra, L, Scarano, V, Cavallo L, Oliva R. (2016) *PLoS One* **11**:e0166460.
6. Vangone A, Spinelli R, Scarano V, Cavallo L, Oliva R. (2011) *Bioinformatics* **27**:2915.
7. Van Der Spoel D, Lindahl E, Hess B, Groenhof G, Mark AE, Berendsen HJ. (2005) *J Comput Chem.* **26**:1701.

Integrative protein complex modeling using deep learning, ZDOCK, and Rosetta

Rui Yin[1,2], Melyssa Cheung[1,3], Johnathan D. Guest[1,2], Jessica Lee[1,2], and Brian G. Pierce[1,2]

[1]University of Maryland Institute for Bioscience and Biotechnology Research, Rockville, MD 20850, USA
[2]Department of Cell Biology and Molecular Genetics, University of Maryland, College Park, MD 20742, USA
[3]Department of Chemistry and Biochemistry, University of Maryland, College Park, MD 20742, USA

A combination of artificial intelligence and physics-based approaches was utilized to generate structural models for this CASP-CAPRI round. For targets with AlphaFold-Multimer[1] models generated by the Elofsson Lab (http://duffman.it.liu.se/casp15/), we applied an ipTM score threshold of 0.75, which was noted previously to discriminate accurate complex structure predictions[2], to screen for high confidence models. When high confidence models were absent or when pre-generated AlphaFold-Multimer target predictions were incomplete (missing chains), which was the case for targets with larger assemblies (e.g. T195, T204, T224, T230) we used AlphaFold[3], ColabFold[4] and RoseTTAFold[5] to generate subunit structures, followed by docking using ZDOCK 3.0.2[6] and RosettaDock[7], or SymDock[8] and M-ZDOCK[9] for symmetric targets. Rosetta's FastRelax protocol[10] was used to refine top-ranked models from ZDOCK or M-ZDOCK, in order to alleviate possible interface clashes due to rigid-body docking. Docking models were pooled together with all 25 AlphaFold-Multimer models (if available), scored and ranked by ZRANK2[11] (which was used for most targets) or Rosetta interface energy score[12]. Top-ranked structures were manually inspected prior to submission. For a subset of targets, information from the literature, if available, and as well as structures of complexes from sequence-based searches against the Protein Data Bank (PDB)[13], or structural searches against the PDB using modeled monomer subunits with DALI[14] was utilized during the docking (through residue blocking in ZDOCK or M-ZDOCK) or during the model selection stage.

Out of 55 interfaces from 37 targets, we successfully generated Medium or higher accuracy models for 55% of interfaces (**Figure 1a**). Additionally, we were generally able to rank the highest accuracy prediction correctly within the five submitted models. For 87% of interfaces, our top-ranked prediction represented the highest accuracy prediction among the five models. Medium or higher accuracy predictions were generated for 61% of the interfaces from "Easy" targets (N=31), and 63% of non-antibody "Difficult" targets (N=16), and 25% of antibody "Difficult" targets (N=8) (target difficulty classifications assigned by CAPRI). Therefore, for general protein-protein complexes (non-antibody/nanobody) target difficulty class did not impact success in achieving moderate/high quality (Medium/High CAPRI accuracy) models.

For T203, which has an A9B3 assembly mode, we found that separate modeling of the A3B1 complex between homotrimeric protein and peptide led to a high confidence model in AlphaFold-Multimer, which we then assembled into the full A9B3 complex through trimerization using M-ZDOCK, relaxed with Rosetta's FastRelax protocol and ranked by Rosetta total energy score. **Figure 1b** shows our rank 3 model for that target, for which the CAPRI accuracy was High (one interface) and Acceptable (three interfaces).

For nanobody-antigen (T205-209) and antibody-antigen (T216-218) complex targets, which are classes of interfaces that are challenging for AlphaFold and AlphaFold-Multimer[1,2], we adopted a multi-stage strategy which started with the use of AlphaFold, AlphaFold-Multimer[1] (for heavy-light chain antibody structures), and ColabFold to generate unbound nanobody and antibody models (antigen structures were available from experimentally determined structures). The unbound structures were docked by ZDOCK 3.0.2, RosettaDock (T205-209), and SnugDock[15] (T216-218). Models were refined with Rosetta FastRelax (ZDOCK models only), pooled along with AlphaFold-Multimer complex models, and scored with ZRANK2 and Rosetta. With these hybrid approaches, we successfully generated Acceptable accuracy models for two nanobody-antigen targets, Medium accuracy models for one

antibody-antigen target, and High accuracy models for one nanobody-antigen target. The challenges and decreased success rate in modeling antibody-antigen and certain other targets highlights the need to expand and improve our modeling strategy for antibody-antigen and complex assemblies where AlphaFold-Multimer fails. Regardless, our overall success in this round indicates that our integrative modeling approach effectively combines AlphaFold-Multimer with previously developed docking and scoring approaches to generate and select accurate models.

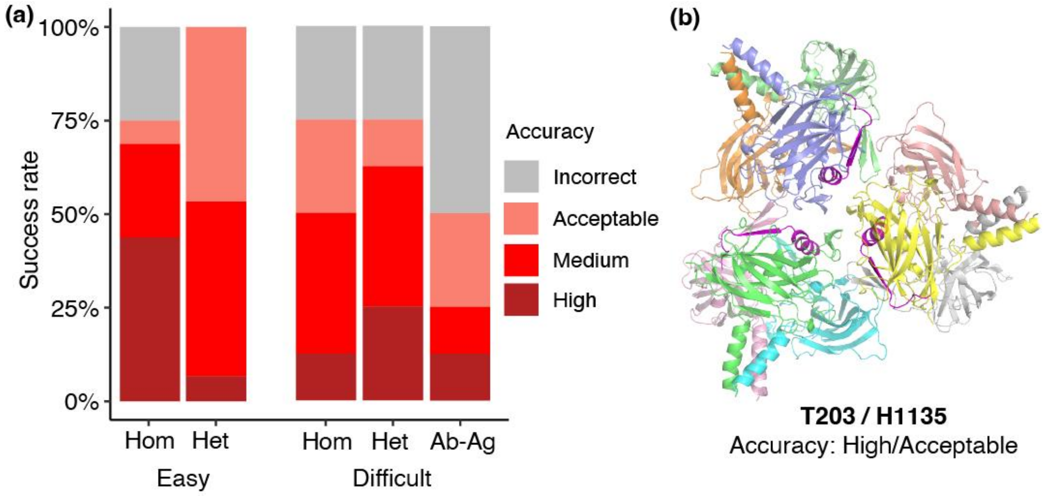

Figure 1. Round 54 CASP15-CAPRI complex prediction success by Pierce group. (a) A total of 55 interfaces of protein-protein complexes were assessed for CAPRI accuracy among the five models submitted by our team. The interfaces were grouped by the prediction difficulty assigned by CAPRI, and interface type. Based on the stoichiometry and complex type of the protein complex, each interface is classified as Homomer ("Hom", denoting an interface formed by single type of subunits), Heteromer ("Het", denoting an interface formed by different types of subunits), or Antibody-Antigen ("Ab-Ag", denoting antibody-antigen or nanobody-antigen complexes). There are 31 "Easy" interfaces, and 24 "Difficult" interfaces. Within each category, the total number of interfaces are 16 (Homomer, Easy), 15 (Heteromer, Easy), 8 (Homomer, Difficult), 8 (Heteromer, Difficult) and 8 (Antibody-Antigen, Difficult). (b) T203 third ranking model by the Pierce group, which was assessed as High, Acceptable, Acceptable, and Acceptable for the four interfaces. The modeled peptide chains are shown in magenta.

Funding information: This work was supported by NIH R35 GM144083 (to BGP).

References

1. Evans R, O'Neill M, Pritzel A, et al. Protein complex prediction with AlphaFold-Multimer. *bioRxiv*. 2021.
2. Yin R, Feng BY, Varshney A, Pierce BG. Benchmarking AlphaFold for protein complex modeling reveals accuracy determinants. *Protein Sci*. 2022;31(8):e4379.
3. Jumper J, Evans R, Pritzel A, et al. Highly accurate protein structure prediction with AlphaFold. *Nature*. 2021;596(7873):583-589.
4. Mirdita M, Schutze K, Moriwaki Y, Heo L, Ovchinnikov S, Steinegger M. ColabFold: making protein folding accessible to all. *Nat Methods*. 2022;19(6):679-682.
5. Baek M, DiMaio F, Anishchenko I, et al. Accurate prediction of protein structures and interactions using a three-track neural network. *Science*. 2021;373(6557):871-876.

6. Pierce BG, Hourai Y, Weng Z. Accelerating protein docking in ZDOCK using an advanced 3D convolution library. *PLoS One*. 2011;6(9):e24657.
7. Gray JJ, Moughon S, Wang C, et al. Protein-protein docking with simultaneous optimization of rigid-body displacement and side-chain conformations. *J Mol Biol*. 2003;331(1):281-299.
8. Andre I, Bradley P, Wang C, Baker D. Prediction of the structure of symmetrical protein assemblies. *Proc Natl Acad Sci U S A*. 2007;104(45):17656-17661.
9. Pierce B, Tong W, Weng Z. M-ZDOCK: a grid-based approach for Cn symmetric multimer docking. *Bioinformatics*. 2005;21(8):1472-1478.
10. Khatib F, Cooper S, Tyka MD, et al. Algorithm discovery by protein folding game players. *Proc Natl Acad Sci U S A*. 2011;108(47):18949-18953.
11. Pierce B, Weng Z. A combination of rescoring and refinement significantly improves protein docking performance. *Proteins*. 2008;72(1):270-279.
12. Alford RF, Leaver-Fay A, Jeliazkov JR, et al. The Rosetta All-Atom Energy Function for Macromolecular Modeling and Design. *J Chem Theory Comput*. 2017;13(6):3031-3048.
13. Berman HM, Westbrook J, Feng Z, et al. The Protein Data Bank. *Nucleic Acids Res*. 2000;28(1):235-242.
14. Holm L. Dali server: structural unification of protein families. *Nucleic Acids Res*. 2022.
15. Weitzner BD, Jeliazkov JR, Lyskov S, et al. Modeling and docking of antibody structures with Rosetta. *Nat Protoc*. 2017;12(2):401-416.

Modelling large macromolecular complexes and antibody-antigen interactions

Ben Shor[1], Tomer Cohen[1], Matan Halfon[1], Dina Schneidman-Duhovny[1]
School of Computer Science and Engineering, The Hebrew University of Jerusalem, Israel

We have focused on modelling two groups of targets that we found as most challenging. The first group is antibody-antigen and nanobody-antigen targets that cannot be predicted using co-evolutionary information from multiple sequence alignment (MSA) that is required for application of deep learning models. The second group is large assemblies, which due to resource limitations cannot be predicted as a whole using AlphaFold2[1,2] on a standard GPU.

We have modelled the antibodies and the nanobodies using NanoNet[3] and AlphaFold2[1]. The models were docked to the antigen using PatchDock[4] and scored by a statistical potential (SOAP)[5] and a deep learning-based scoring function, ContactNet[6]. ContactNet is an attention-based GNN model, trained for classifying antibody-antigen complex models obtained from docking algorithms into accurate and incorrect ones. Inspired by end-to-end structure modelling, during CASP, we have developed a fold&dock model[7] that given an antigen structure and antibody sequence produces accurate complex models that include side-chains. The network simultaneously folds the antibody (light and heavy chains) and docks it to the antigen. An accurate model is detected for 30% and 60% of the test set among the Top-5 and Top-50 predictions, respectively. Overall, the modeling of these CASP targets was not successful because the methods were still in the development phase.

The large assemblies were modelled through hierarchical application of AlphaFold-multimer-v2 (AFM)[2]. We applied AFM to calculate models for pairs (or larger subsets) of subunits (domains or chains) that were further assembled into larger models using alignment or docking. For targets with cyclic symmetry, where AFM pairwise models were not fully compatible with cyclic symmetry, we relied on SymmDock[8] to generate symmetric models and select those with a pairwise interaction interface most similar to the one produced by AFM.

For the more complicated case (H1137) we run AFM for all pairs of chains (Fig. 1A). These results enabled us to identify domains and domain-domain interactions, such as helical domains, roughly the same length in six different chains that were folding into a tube-like structure. Interactions between domains that received a low PAE score (<10.0) were used to generate an interaction graph between the domains, enabling us to group the interacting domains into four groups. The domain fragments we used had overlapping residues to enable us to connect consecutive domains by structural alignment of these residues. In certain cases, the overlap was of tens of residues, and in other cases it was of entire chains (Table 1). AFM was used to calculate models for each of the four groups (Fig. 1B) and the overlapping residues were used to connect the models of the four groups and obtain the final model (Fig. 1C). We have submitted a single model which had the highest Contact Agreement Score (QS) of 0.896 among all submissions for this target with TM-score of 0.869.

Chains	Domain 1	Domain 2	Domain 3
A	1-175	155-320	280-409
B	1-154	134-302	182-263
C	1-155	135-300	280-384
D	1-167	147-315	295-487
E	1-174	154-318	298-410
F	1-157	137-300	280-423
H	whole chain		
I	whole chain		

Table 1. Subdivision of H1137 chains into a set of overlapping domains.

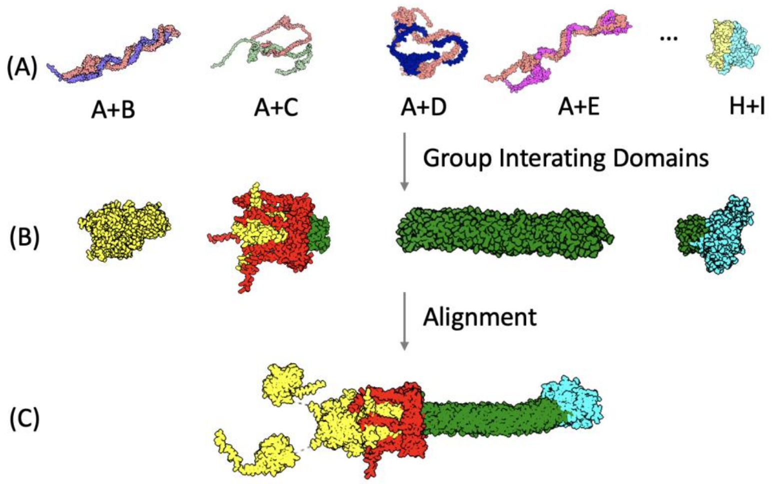

Figure 1. Hierarchical assembly of the H1137 target. **(A)** pairwise domain-domain interactions **(B)** AFM models of the four subgroups **(C)** the final model.

References

1. Jumper J, Evans R, Pritzel A, et al. Highly accurate protein structure prediction with AlphaFold. *Nature.* 2021.
2. Evans R, O'Neill M, Pritzel A, et al. Protein complex prediction with AlphaFold-Multimer. *bioRxiv.* 2021:2021.2010.2004.463034.
3. Cohen T, Halfon M, Schneidman-Duhovny D. NanoNet: Rapid and accurate end-to-end nanobody modeling by deep learning. *Frontiers in immunology.* 2022;13:958584.
4. Duhovny D, Nussinov R, Wolfson HJ. Efficient Unbound Docking of Rigid Molecules. Paper presented at: Second International Workshop, WABI 20022002; Rome, Italy.
5. Dong GQ, Fan H, Schneidman-Duhovny D, Webb B, Sali A. Optimized atomic statistical potentials: assessment of protein interfaces and loops. *Bioinformatics.* 2013;29(24):3158-3166.

6. Halfon M, Cohen T, Fattal R, Schneidman-Duhovny D. ContactNet: attention-based deep learning model for predicting antibody-antigen interactions. . *NeurIPS MLSB* 2022.
7. Cohen T, Schneidman-Duhovny D. End-to-end accurate and high-throughput modeling of antibody-antigen complexes. *NeurIPS MLSB.* 2022.
8. Schneidman-Duhovny D, Inbar Y, Nussinov R, Wolfson HJ. PatchDock and SymmDock: servers for rigid and symmetric docking. *Nucleic Acids Res.* 2005;33(Web Server issue):W363-367.

Shen

Shaowen Zhu[1], Rujie Yin[1], Yuanfei Sun[1], and Yang Shen[123]*

[1] Department of Electrical and Computer Engineering, Texas A&M University, College Station, Texas 77843, United States
[2] Department of Computer Science and Engineering, Texas A&M University, College Station, Texas 77843, United States
[3] Institute of Biosciences and Technology and Department of Translational Medical Sciences, Texas A&M University, Houston, Texas 77030, United States

* E-mail: yshen@tamu.edu

Methods

Docking. We used AlphaFold-Multimer [1], SAM [2], ClusPro [3] and homology models to build initial structure models, our own BAL (Bayesian Active Learning) [4] to refine them, and a suite of scoring functions including our own BAL and EGCN (Energy-based Graph Convolution Networks) [5] to select final submissions. More details are provided as follows.

Initial Structure Predictions. For most targets, especially dimers, we started with AlphaFold-Multimer (AF-M in short) predictions (usually 25) kindly provided by the Elofsson group [6] at http://duffman.it.liu.se/casp15/. For some targets we had to generate more initial models with other protocols when the interface pTM (ipTM) scores of all AF-M models were too low (below 0.23). With few exceptions, those cases demanding other protocols for initial models often involved oligomers with orders higher than dimers or antigen-antibody complexes. In such cases, we searched for homology templates using HHpred (searching with monomer sequences) and PPI3D (searching with two sequences for hetero-oligomers); and, if no templates were identified, we used the following protocols (hereinafter we use CAPRI/CASP indices [and stoichiometry, if applicable] to identify each target):

Homo-oligomers exceptions

- T195/T1115 (A16). We clustered monomer structures from the provided A8 oligomer predictions using TM scores and selected five representatives (a8_middle_monomer_0; a8_middle_monomer_5; a8_middle_monomer_14; a8_middle_monomer_18; a1_ranked_0). We used SAM to generate ten C16 (cyclic symmetry) models for each monomer and then selected three for each based on the similarity to a template (PDB ID: 7VHP), the diversity and the number of clashes.
- T196/T1119 (A3). We identified templates 6N9H & 5J0J (C3 symmetric helical bundle).
- T198/T1123 (A2). Identified templates (3QSQ & 5EWO) did not include the supposed interfaces and were not prioritized.
- T201/T1132 (A6). Potential templates for A2 and A3 were identified (3KKF and 1TZ0, respectively). We used monomer predictions from AF2 to manually build trimer of dimers and dimer of trimers based on the A2/A3 templates.
- T224/T1176 (A8). Only a dimer template (1WMX) was identified. So we used SAM to dock A1 structure models (AF2) using C8 and D4 symmetries and dock A2 structure models using C4 and D2 symmetries.
- T230/T1192 (A10). We again used SAM for A10 assemblies using A1 structure models.

Hetero-oligomer exceptions

- T203/H1135 (A9B3). We adopted an incremental assembly approach. We first used the first A3 prediction from AF-M to build A9 assemblies by SAM. We also docked B1 (peptide sequence input) to A3 (structure model from AF-M) in the hypothetical inter-chain site by CABSdock. We then align the predicted A3B1 models to various subunits of the predicted A9 models to generate initial perditions.
- T204/H1137 (A1B1C1D1E1F1G2I1J1). We identified a template (7CH9) for the architecture but monomer conformations had to be adjusted to remove clashes.
- T219-221/H1170-1172 (A6, A6B1, and A6B2). Again an incremental assembly approach was used. AF-M predictions for A6 were of high ipTM and matched the template 6H7X. Then template 1IXS (A1B1) was used for T220 and T221.

For antigen-antibody complexes (T205-209/H1140-1144 nanobodies and T216-218/H1166-1168 antibodies) and few dimers (T200-H1129 (A1B1) and T229/T1187 (A2)), we used ClusPro to dock the monomer models from AlphaFold2 and retained the top 10 for each set of energy function. For antigen-antibodies we used both the default and the antibody modes of ClusPro.

Uncertainty-Aware Flexible Refinement. We used BAL (Bayesian Active Learning) [4] that (1) refines initial predictions in the space spanned by encounter complex-based normal modes [7]; (2) actively learns the posterior distribution of the native structures under uncertainty; and (3) estimates the absolute quality (iRMSD) and the relative quality (ranking) of the final predictions.

Multi-Criteria Selection. By default, after clustering BAL-refined predictions using FCC, we used the top two based on AF-M's weighted sum of ipTM and pTM (before BAL refinement), top 1 among the rest based on ipTM alone, top 1 among the rest based on the probability of each structure model estimated by BAL, and top 1 among the rest based on EGCN (Energy-based Graph Convolutional Networks) score again after BAL. When AF-M had low confidence, we relied more on EGCN especially when no reliable templates were identified.

Scoring. We used FCC to cluster given decoys and EGCN to re-rank the cluster heads.

Acknowledgement

The study was in part supported by NIH/NIGMS (R35GM124952 to YS) and NSF (CCF-1943008 to YS). Portions of this research were conducted with the advanced computing resources provided by Texas A&M High Performance Research Computing.

References

[1] R. Evans, M. O'Neill, A. Pritzel, N. Antropova, A. Senior, T. Green, A. Žídek et al. "Protein complex prediction with AlphaFold-Multimer." BioRxiv (2022): 2021-10.
[2] D.W. Ritchie and S. Grudinin. Spherical polar Fourier assembly of protein complexes with arbitrary point group symmetry. Journal of Applied Crystallography (2016): 49(1), pp.158-167.
[3] D. Kozakov, D.R. Hall, B. Xia, K.A. Porter, D. Padhorny, C. Yueh, D. Beglov and S. Vajda. The ClusPro web server for protein-protein docking. Nature protocols (2017), 12(2), pp.255-278.
[4] Y. Cao and Y. Shen. Bayesian active learning for optimization and uncertainty quantification in protein docking. Journal of Chemical Theory and Computation (2020), 16(8), pp.5334-5347.
[5] Y. Cao and Y. Shen. Energy-based graph convolutional networks for scoring protein docking models. Proteins (2020), 88(8), pp.1091-1099.
[6] P. Bryant, P., G. Pozzati, and A. Elofsson. Improved prediction of protein-protein interactions using AlphaFold2. Nature communications (2022), 13(1), pp.1-11.
[7] T. Oliwa and Y. Shen. cNMA: a framework of encounter complex-based normal mode analysis to model conformational changes in protein interactions. Bioinformatics (2015), 31(12): pp. i151-i160.

M. Maszota-Zieleniak[1], K.K. Bojarski[2], E.A. Lubecka[2], M. Marcisz[1], A. Danielson[1], Ł. Dziadek[1], M. Gaardløs[1], A. Gieldon[1], A. Liwo[1], S.A. Samsonov[1], R. Slusarz[1], K. Zieba[1], A.K. Sieradzan[1,*], C. Czaplewski[1]

1. University of Gdańsk, Wita Stwosza 63, Gdańsk
2. Technical University of Gdańsk, ul. G. Narutowicza 11/12, Gdańsk

We participated in CAPRI Round 54 as UNRES group all of which used the latest version of the coarse-grained UNRES force field [1]. The UNRES group used distance- and local-structure restraints extracted from server model, as described previously [2]. The restraints were imposed only on monomers. The UNRES software was modified to handle large oligomeric targets.

The protocol for oligomer-structure prediction. Initial multimeric models were built from monomer structures. The monomers were, in turn, modeled, within the CASP15 experiment, by using our hierarchical protocol, in which restrained MREMD (Multiplexed Replica Exchange Molecular Dynamics) [3] simulations with the coarse-grained UNRES force field [1] were carried out. Simulations of monomers were started from multiple server models.

When oligomeric server predictions were available we used them as starting points for simulations. Otherwise we used an oligomeric template from HHpred [4] when available, while in other cases the monomers were assembled into the initial oligomer structures by using the random-oligomer-positioning algorithm of UNRES-dock [5]. For each target, multiple oligomer structures were constructed. The starting structures were subjected to restrained MREMD simulations with the UNRES force field [1], as described previously [2]. Subsequently, simulation results were processed by using the Weighted Histogram Analysis Method (WHAM) [6] and cluster analysis to extract 10 families of conformations [4]. The families are ranked based on their free energies (each computed over the entire conformational sub-ensemble constituting a family [1]). Coarse-grained structures which were closest to the cluster centers were subsequently converted to all-atom representation by using the PULCHRA [7] and SCWRL [8] knowledge-based algorithms, and subjected to final refinement at the all-atom level with the AMBER ff14SB force field [9].

Acknowledgments

Supported by National Science Center of Poland (Narodowe Centrum Nauki) (NCN), grants UMO-2017/25/B/ST4/01026, UMO-2017/26/M/ST4/00044, and UMO-2017/27/B/ST4/00926. Computational resources were provided by (a) the Interdisciplinary Centre of Mathematical and Computational Modelling (ICM) at the University of Warsaw (b) the Centre of Informatics – Tricity Academic Superkomputer & NetworK (CI TASK) in Gdańsk, (c) the Polish Grid Infrastructure (PL-GRID), and (d) our Beowulf cluster at the Faculty of Chemistry, University of Gdańsk.

[1] A. Liwo, A.K. Sieradzan, A.G. Lipska, C. Czaplewski, I. Joung, W. Żmudzińska, A. Hałabis,A., S. Ołdziej. J. Chem. Phys., 150 (2019) 155104.

[2] A.S. Karczyńska, K. Zięba, U. Uciechowska, M.A. Mozolewska, P. Krupa, E.A. Lubecka, A.G. Lipska, C. Sikorska, S.A. Samsonov, A.K. Sieradzan, A. Giełdoń, A. Liwo, R. Ślusarz, M. Ślusarz, J. Lee, K. Joo, C. Czaplewski. J. Chem. Inf. Model.,60 (2020) 1844-1864.

[3] Y.M. Rhee, V.S. Pande, Biophys. J. 84 (2003) 775-786.

[4] Zimmermann L, Stephens A, Nam SZ, Rau D, Kübler J, Lozajic M, Gabler F, Söding J, Lupas AN, Alva V. J Mol Biol. 430 (2018) 2237-2243.

[5] P. Krupa, A.S. Karczyńska, M.A. Mozolewska, A. Liwo, C. Czaplewski. Bioinformatics, (2021) btaa897, https://doi.org/10.1093/bioinformatics/btaa897

[6] S. Kumar, D. Bouzida, R.H. Swendsen, P.A. Kollman, J.M. Rosenberg. J. Comput. Chem. 13 (1992) 1011-1021.

[7] P. Rotkiewicz, J. Skolnick. J. Comput. Chem. 29 (2008) 1460-1465.

[8] Q. Wang, A.A. Canutescu, R.L. Dunbrack. Nat. Protoc. 3 (2008) 1832-1847.

[9] J.A. Maier, C. Martinez, K. Kasavajhala, L. Wickstrom, K.E. Hauser and C. Simmerling. J. Chem. Theory Comput. 11, (2015) 3696-3713.

Structure Prediction in CASP15-CAPRI Round 54

Shinpei Kobayashi, Yuta Miyakawa, Yasuomi Kiyota and Mayuko Takeda-Shitaka

School of Pharmacy, Kitasato University, 5-9-1 Shirokane, Minato-ku, Tokyo 108-8641, Japan

E-mail addresses
Shinpei Kobayashi : dl21101@st.kitasato-u.ac.jp
Yuta Miyakawa : miyakawa.yuta@st.kitasato-u.ac.jp
Yasuomi Kiyota : kiyotay@pharm.kitasato-u.ac.jp
Mayuko Takeda-Shitaka : shitakam@pharm.kitasato-u.ac.jp

Introduction

We participated in the assembly category of CASP15-CAPRI54 experiment. Basically, we used AlphaFold-Multimer[1] for construction of multimeric protein structure models and used our model quality assessment method for ranking. Here, we briefly describe our methods.

Methods

Human Predictor

Model construction

We constructed multimeric protein structure models (25 models per target) using AlphaFold-Multimer for assembly targets. We predicted both homo- and hetero- oligomeric protein structures according to the oligomeric state in the target list of CASP15. We also used AlphaFold-Multimer models (25 models per target) generated by the Elofsson group (http://duffman.it.liu.se/casp15/).

Template Search

To obtain monomeric and multimeric templates, we carried out two-step template search[2]. Firstly, templates were searched by HHblits[3] against UniRef30 and PDB70. Secondly, to search templates more widely, we ran PSI-BLAST[4] on PDBaa using HHblits hits obtained in the first step as inputs.

Further model construction

We visually inspected model structures and compared them with template structures obtained by two-step template search. When models had unreliable or unfolded regions, we divided target sequences into domains or regions and constructed models using AlphaFold-Multimer. For large targets that had many subunits, we constructed partial structure models. Finally, we applied docking of models to obtain whole structure models. Human intervention was made if necessary.

Assessment of model quality

For dimeric targets, we used our original quality assessment method[5]. This method was developed

to predict the ranking of dimeric models by the DockQ score[6], using the learning to rank approach with LambdaMART[7]. As features, we used SOAP-PP score[8], VoroMQA interface score[9], and Rosetta scores[10] (Total Rosetta score and 10 energy terms). The training set was the protein-protein docking benchmark 4.0[11], containing 174 bound structures. For other multimeric targets, we used the model confidence score from AlphaFold-Multimer for ranking. Models with many clashes at the interface were manually removed.

Scorer

For dimeric targets, we used the same quality assessment method for out human prediction. For other multimeric targets, models were selected based on structure similarity to the best model of our human prediction, using US-align[12]. Models with many clashes at the interface were manually removed. Based on the results, 10 models were selected.

Calculations in this study were performed using the computer systems lasc and olive of School of Pharmacy, Kitasato University.

Performance

For hetero-dimer T202 classified as difficult by the assessors, our group achieved a high-quality model in human prediction. We were able to select a high-quality model from models that have two different conformations in an α-helix region (shown in magenta and orange in Figure 1(a)), using our original quality assessment method (Figure 1).

For hetero-10mer T204 classified as difficult and assessed as AU1 (T204.1-2) and AU2 (T204.3-8) by the assessors, our group achieved medium-quality in AU1 and high-quality in several interfaces of AU2 in human prediction. We performed further model construction using our method described above taking into account structural information of an oligomeric template (PDB 7ch8) obtained by two-step template search.

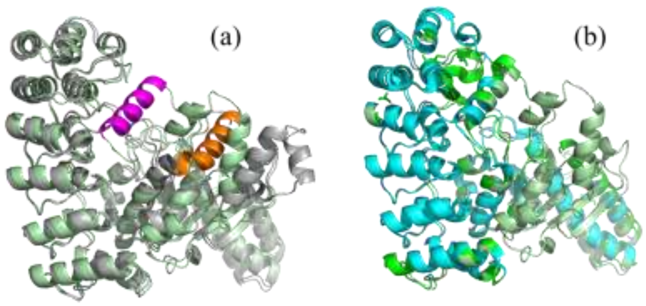

Figure 1. (a) Structural alignment of our high-quality model of T202 and another model by US-align.

Our high-quality model is shown in palegreen (α-helix is colored magenta) and another model is shown in gray (α-helix is colored orange). (b) Structural alignment of our high-quality model and the experimental structure (PDB 7ubz) by US-align. Our high-quality model is shown in palegreen and the experimental structure is shown in green (correctly predicted regions where distance between corresponding Cα atoms is ≦ 1Å are colored cyan in both structures).

References

1. Evans R, et al. Protein complex prediction with alphafold-multimer. *bioRxiv*. 2021.
2. Kiyota Y, Kobayashi S, Harada Y, Takeda-Shitaka M. CASP14 abstract book. https://predictioncenter.org/casp14/doc/CASP14_Abstracts.pdf. 266-267.
3. Steinegger M, et al. HH-suite3 for fast remote homology detection and deep protein annotation. *BMC Bioinformatics*. 2019;20(1):473.
4. Altschul SF, et al. Gapped BLAST and PSI-BLAST: a new generation of protein database search programs. *Nucleic Acids Res*. 1997;25(17):3389-3402.
5. Harada Y. Kitasato University, thesis, 2022.
6. Basu S, Wallner B. DockQ: A Quality Measure for Protein-Protein Docking Models. *PLoS One*. 2016;11(8):e0161879.
7. Burges C. From RankNet to LambdaRank to LambdaMART: an overview. *Technical report*, Microsoft Research. 2010.
8. Dong GQ, et al. Optimized atomic statistical potentials: assessment of protein interfaces and loops. *Bioinformatics*. 2013;29(24):3158-3166.
9. Olechnovič K, Venclovas Č. VoroMQA: Assessment of protein structure quality using interatomic contact areas. *Proteins*. 2017;85(6):1131-1145.
10. Alford RF, et al. The Rosetta All-Atom Energy Function for Macromolecular Modeling and Design. *J Chem Theory Comput*. 2017;13(6):3031-3048.
11. Hwang H, et al. Protein-protein docking benchmark version 4.0. *Proteins*. 2010;78(15):3111-3114.
12. Zhang C, et al. US-align: universal structure alignments of proteins, nucleic acids, and macromolecular complexes. *Nat Methods*. 2022;19(9):1109-1115.

Modeling and Scoring Protein Assemblies in CAPRI Round 54 (CASP15-CAPRI)

Kliment Olechnovič, Lukas Valančauskas, Justas Dapkūnas and Česlovas Venclovas
Institute of Biotechnology, Life Sciences Center, Vilnius University
kliment.olechnovic@bti.vu.lt, lukas.valancauskas@gmc.vu.lt, justas.dapkunas@bti.vu.lt, ceslovas.venclovas@bti.vu.lt

Methods

The general workflow for 3D structure prediction of protein assemblies consisted of two major stages: (1) construction of an ensemble of multiple diverse structural models and (2) selection of the best models using a newly developed accuracy estimation protocol. Accuracy estimation procedures were based on both new and previously developed contact-area based scoring functions (see Fig. 1).

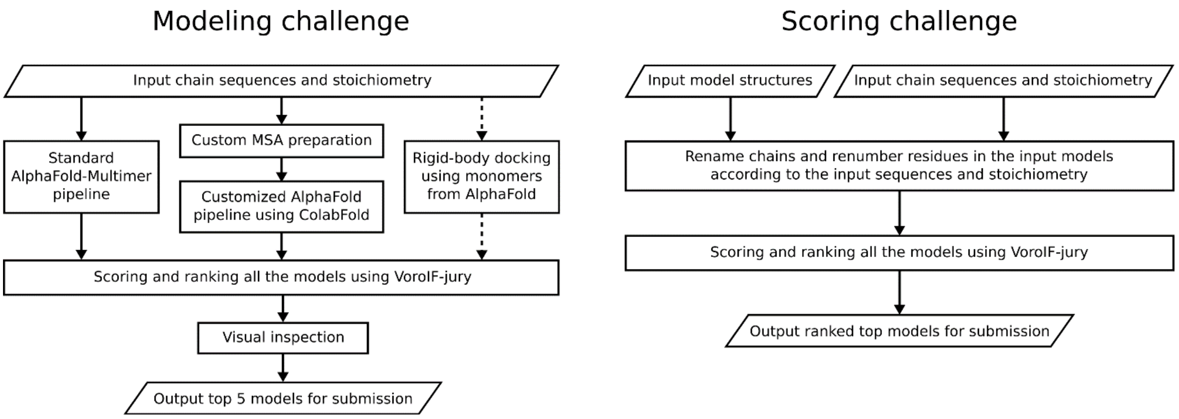

Figure 1. Schematic illustration of the workflow for modeling (left) and scoring (right) challenges

Construction of 3D models. Initial ensembles of protein complexes were constructed using AlphaFold-Multimer-v2[1,2] available either as the ColabFold[3] or the original DeepMind's implementation. The DeepMind's AlphaFold modeling pipeline included both full and reduced database presets for multiple sequence alignment (MSA) generation and PDB templates. The ColabFold-based AlphaFold modeling pipeline employed a variety of different parameters and conditions so as to achieve extensive structure sampling. These variations included the choice of sequence databases, the construction and pairing of MSAs as well as varying the number of AlphaFold recycles and using the AlphaFold-pTM version for multimer modeling. If AlphaFold failed to generate the complex (assembly was too large to handle or subunits did not form a complex), structural models were obtained using docking. Docking models were also added if the resulting AlphaFold models had poor self-estimated accuracy (pLDDT, pTM, ipTM) or did not show structural consensus. Rigid-body docking was performed with FTDOCK[4] and HEX[5] for generating heterodimers, whereas SAM[6] was used for generating symmetric homomers. In several cases, when closely related PDB templates were identified by PPI3D[7], homology models[8] were also constructed and added to the model set.

Model ranking and selection. Model ranking and selection for both protein assembly prediction and scoring challenge was done using a newly developed VoroIF-jury (Voronoi-based InterFace jury) procedure. VoroIF-jury resembles EMA-jury, developed previously for assessing the models in a recent CASP-commons experiment focused on SARS-CoV-2[9]. Given a set of models, VoroIF-jury (a) computes multiple rankings using different interface-focused scores (most of them based on the VoroMQA interface energy[10]); (b) pools the top 1, top 2, ... , top N models selected by

each EMA ranking into N corresponding supersets; (c) for every model in each superset calculates the VoroIF-jury interface consensus score, that is an average of the interface CAD-score[11] values derived by comparing a given model with other models in the superset; (d) ranks models by the best achieved VoroIF-jury score; (e) removes redundant models from the final ranking using the interface CAD-score-based clustering. For docking models VoroIF-jury was applied in two stages: (1) selecting top 300 from all the docking models, often exceeding 100 000; (2) after relaxing those 300 models using OpenMM[12] to remove clashes, selecting the final top five models. VoroIF-jury included two newly developed interface scoring methods. The first one, a generic interatomic contact area-based energy potential, applicable for scoring of not only protein-protein, but also protein-nucleic acids interfaces, was derived from the protein-protein VoroMQA potential[10]. The second one, VoroIF-GNN, a graph neural network-based method, was developed for predicting the residue-level interface accuracy in models of protein-protein complexes. VoroIF-GNN is based on a graph attention network (GAT) that accepts a Voronoi tessellation-derived graph of inter-chain interface contacts. The network was trained using heterodimeric models produced by rigid-body redocking of complexes from PDB. The ground truth interface quality scores were calculated by comparing the models with the corresponding experimental structures using interface CAD-score[11].

Analysis of the available structural data. Available additional structural information (e.g., PDB templates, stoichiometry) related to the target protein assemblies or their homologs was also considered for manual model selection and re-ranking for some challenging targets.

Results

According to CAPRI criteria, our top-ranked models were of high accuracy for 12 out of 37 targets, medium accuracy for 9, and acceptable accuracy for 5 targets. For 11 targets our top models were incorrect. Subsequent analysis suggested that the sampling using both AlphaFold and docking was not sufficient for these failed targets, especially for the antibody-antigen complexes. Notably, AlphaFold was unable to produce full models for large protein complexes (CAPRI T195/CASP T1115 (A_{16}), T203/H1135 (A_9B_3) and T204/H1137 ($ABCDEFG_2HI$)). In these cases, we successfully modeled full structures from partial AlphaFold models by either docking or combining models of overlapping subcomplexes. The VoroIF-jury scoring method, which did not use any of AlphaFold-specific scores, was not only instrumental in our modeling pipeline consisting of different structure prediction methods, but also performed successfully in selecting accurate models from the diverse CAPRI scoring sets.

Availability

The methods developed in our laboratory are available at https://bioinformatics.lt/software/.

Funding: Research Council of Lithuania (grant S-MIP-21-25).

References

1. Jumper,J., et al (2021) Highly accurate protein structure prediction with AlphaFold. *Nature* **596**, 583–589.
2. Evans,R., et al (2022) Protein complex prediction with AlphaFold-Multimer. *bioRxiv* 2021.10.04.463034 doi:10.1101/2021.10.04.463034.
3. Mirdita,M., Schütze,K., Moriwaki,Y., Heo,L., Ovchinnikov,S. & Steinegger,M. (2022) ColabFold: making protein folding accessible to all. *Nat Methods* **19**, 679–682.
4. Gabb,H.A., Jackson,R.M. & Sternberg,M.J. (1997) Modelling protein docking using shape complementarity, electrostatics and biochemical information. *J Mol Biol* **272**, 106–120.

5. Ritchie,D.W. & Kemp,G.J. (2000) Protein docking using spherical polar Fourier correlations. *Proteins* **39**, 178–194.
6. Ritchie,D.W. & Grudinin,S. (2016) Spherical polar Fourier assembly of protein complexes with arbitrary point group symmetry. *J Appl Cryst* **49**, 158–167.
7. Dapkūnas,J., Timinskas,A., Olechnovič,K., Margelevičius,M., Dičiūnas,R. & Venclovas,Č. (2017) The PPI3D web server for searching, analyzing and modeling protein-protein interactions in the context of 3D structures. *Bioinformatics* **33**, 935–937.
8. Dapkūnas,J., Olechnovič,K. & Venclovas,Č. (2021) Modeling of protein complexes in CASP14 with emphasis on the interaction interface prediction. *Proteins* **89**, 1834–1843.
9. Kryshtafovych,A., et al (2021) Modeling SARS-CoV-2 proteins in the CASP-commons experiment. *Proteins* **89**, 1987–1996.
10. Olechnovič,K. & Venclovas,Č. (2017) VoroMQA: Assessment of protein structure quality using interatomic contact areas. *Proteins* **85**, 1131–1145.
11. Olechnovič,K. & Venclovas,Č. (2020) Contact Area-Based Structural Analysis of Proteins and Their Complexes Using CAD-Score. *Methods Mol Biol* **2112**, 75–90.
12. Eastman,P., et al (2017) OpenMM 7: Rapid development of high performance algorithms for molecular dynamics. *PLoS Comput. Biol.* **13**, e1005659.

Improved Multimer Prediction using Aggressive Sampling with AlphaFold

Björn Wallner
Bioinformatics Division, Department of Physics, Chemistry, and Biology, Linköping University
bjorn.wallner@liu.se

In this CAPRI, we used a modified version of AlphaFold[1] that has improved sampling capabilities and has shown good performance in modeling peptide-protein structures[2]. The method is fully automated but was run as a manual server to allow for more computational time. To achieve improved sampling, we activated the dropout layers during inference and generated a minimum of 1,000 models using six different settings. These settings involved utilizing templates, omitting templates, and employing an increased number of recycles for both multimer version 1 (v1) and version 2 (v2) weights. In total, we generated 6,000 models for each target. To facilitate a direct comparison with the baseline AlphaFold version, we used multiple sequence alignments created using the default setting in AlphaFold with databases updated before the start of CASP15 and provided by the CASP community at http://bioinfo.ifm.liu.se/casp15/. Generating this many models was time-consuming for some large targets but not an issue for most targets. Furthermore, if the best ranking_confidence reported by AlphaFold among the 6,000 models was less than 0.70, we generated additional models. For certain targets, we generated up to 30,000 models. The model with the highest score was consistently submitted as rank 1. But to avoid submitting five identical models, we implemented a filter that selected the model with the highest score but with a TMscore from MMalign[3] lower than 0.8 compared to a higher-ranked model. The method is available here: http://wallnerlab.org/AFsample/.

Funding

Wallenberg AI, Autonomous System and Software Program (WASP) and Berzelius from Knut and Alice Wallenberg Foundation (KAW), Swedish Research Council, 2020-03352, The Swedish e-Science Research Center, and Carl Tryggers stiftelse för Vetenskaplig Forskning, 20:453.

1. Jumper, J. *et al.* (2021). Highly accurate protein structure prediction with AlphaFold. *Nature* 1–11 doi:10.1038/s41586-021-03819-2.
2. Johansson-Åkhe, I. & Wallner, B. (2022). Improving Peptide-Protein Docking with AlphaFold-Multimer using Forced Sampling. *Front. Bioinform.* doi: 10.3389/fbinf.2022.959160
3. Mukherjee, S. & Zhang, Y. (2009). MM-align: a quick algorithm for aligning multiple-chain protein complex structures using iterative dynamic programming. *Nucleic Acids Res* **37**, e83–e83.

Spatial Layouts of Low-Entropy Hydration Shells can be used for prediction of structures of Protein complexes

Lin Yang [a,b], Chengyu Hou[c], Xiaodong He[a,d,*], Shuai Guo [a], Shenda Jiang[a], Xiaolang Ma

[a] *National Key Laboratory of Science and Technology on Advanced Composites in Special Environments, Center for Composite Materials and Structures, Harbin Institute of Technology, Harbin 150080, China*

[b] *School of Aerospace, Mechanical and Mechatronic Engineering, The University of Sydney, NSW 2006, Australia*

[c] *School of Electronics and Information Engineering, Harbin Institute of Technology, Harbin 150080, China*

[d]*Shenzhen STRONG Advanced Materials Research Institute Co., Ltd, Shenzhen 518035, P. R. China.*

Methodology:

Our prediction method includes three steps. First, we identify low-entropy regions of hydration shells of proteins by screening off pseudo hydrophilic groups on protein surfaces and revealing that large low-entropy regions of the hydration shells typically cover the binding sites of individual proteins. In our method, all the binding sites of protein pairs should be covered by the shape-matched large low-entropy regions of the hydration shells, enable the largest low-entropy regions fully collapse at the protein-protein interfaces during the docking processes. Second, we identify two shape-matched low-entropy regions of the hydration shell on each protein pairs as the bind sites. Third, we dock the two protein subunits and make the two low-entropy regions fully shield with each other. Then, we get the prediction results.

To ensure that submitted models strictly adhere to the standard format and residue numbering, the submission PDB format uses a template that be provided for each target by the CAPRI system. All the three-dimensional (3D) structure data of individual protein subunits are resourced from the CASP prediction results generated by the Elofsson group and Baker group. By the way, some protein subunits structures were manually bended to facilitate the docking prediction.

Funding
The authors acknowledge the financial support from the Science Foundation of the National Key Laboratory of Science and Technology on Advanced Composites in Special Environments, the Fundamental Research Funds for the Central Universities of China.

References:

1.Yang L, Li J, Guo S, et al. SARS-CoV-2 variants, RBD mutations, binding affinity, and antibody escape[J]. International journal of molecular sciences, 2021, 22(22): 12114.
2.Li J, Hou C, Ma X, et al. Entropy-enthalpy compensations fold proteins in precise ways[J]. International journal of molecular sciences, 2021, 22(17): 9653.
3. Li J, Ma X, Guo S, et al. A Hydrophobic‐ Interaction‐ Based Mechanism Triggers Docking between the SARS‐ CoV‐ 2 Spike and Angiotensin‐ Converting Enzyme 2[J]. Global Challenges, 2020, 4(12): 2000067.
4. Lin Y, Shuai G, Xiao-Liang M, et al. Universal initial thermodynamic metastable state of unfolded proteins[J]. Progress in biochemistry and biophysics, 2019, 46(10): 993-1001.
5. Yang, L., Guo, S., Liao, C., Hou, C., Jiang, S., Li, J., Ma, X., Shi, L., Ye, L. and He, X. (2023), Spatial Layouts of Low-Entropy Hydration Shells Guide Protein Binding. Global Challenges 2300022. https://doi.org/10.1002/gch2.202300022

Lin Yang email:linyang@hit.edu.cn
Xiaodong He email:hexd@hit.edu.cn
Chengyu Hou email:houcy@hit.edu.cn
Shuai Guo email:20B918032@stu.hit.edu.cn
Shenda Jiang email:22B918022@stu.hit.edu.cn
Xiaoliang Ma email:mxlprc@163.com

Summary of the methods used by the Zou Group and the MDockPP server

Rui Duan[1], Liming Qiu[1], Xianjin Xu[1], Xiaoqin Zou[1,2,3,4]

1. Dalton Cardiovascular Research Center, University of Missouri, Columbia, Missouri, USA.
2. Department of Physics and Astronomy, University of Missouri, Columbia, Missouri, USA.
3. Department of Biochemistry, University of Missouri, Columbia, Missouri, USA.
4. Institute for Data Science and Informatics, University of Missouri, Columbia, Missouri, USA.

We participated in the joint CASP15-CAPRI experiment in both the predicting and scoring categories for all the announced targets. Different from our approaches used in the previous CASP14-CAPRI experiment[1], AlphaFold v2.2.0[2,3] was applied to generate monomeric structures for docking. In addition, molecular dynamics (MD) simulations with explicit water were performed to refine the complex structures.

In the prediction challenge, AlphaFold combined with the docking-based method was applied for the given targets. Specifically, the monomeric structures used for docking were generated by AlphaFold via two approaches, 1) directly generated by the AlphaFold-Monomer model, and 2) extracted from the multimeric structures built by the AlphaFold-Multimer model. Both AlphaFold monomer and multimer modeling were run on a single NVIDIA Tesla V100 GPU with default parameters using the latest date as the maximum template release date. The monomers were clustered by the root-mean-square deviation (RMSD) of heavy atoms and the clustered monomers were thereafter used as the input for docking. Docking was performed on our in-house developed GPU version of the MDockPP server[4,5], using a fast Fourier transform (FFT)-based rigid docking algorithm[6] to generate putative binding modes. The docking was performed on a single NVIDIA GEFORCE RTX 2080 Ti GPU. The biological information searched by the in-house Rebipp server[1,4] was also used as one of the inputs for the MDockPP server. The generated binding poses were optimized and ranked with our latest ITScorePP scoring function[5], which is an atomic-level, statistical potential-based scoring function for protein-protein interactions. Next, the ranked binding modes were clustered according to their heavy atom RMSDs. The best model from each of the top 10-ranked clusters was selected and submitted to CAPRI as the MDOCKPP server prediction.

Differently, in the human prediction, the clustered multimeric structures generated by the AlphaFold-Multimer model were used as the initial structure for MD simulations. The complexes were solvated in a periodic box of water using the Leap module, with counter ions added to neutralize the system. The complexes and water were represented by ff14SB[7] and TIP3P[8] models, respectively. The solvated complexes were then minimized and heated from 0 to 300 K in 100 ps, followed by 100 ns (or 10 ns) of NPT simulation with a time step of 2 fs. All the MD simulations were performed using AMBER16 package[9]. The frames of the simulations were clustered based on the heavy atom RMSD. All the clustered frames together with the docking-generated binding modes were ranked with ITScorePP and clustered according to their RMSDs. Up to 100 clustered binding modes were manually inspected, and ten complexes were selected for CAPRI submission.

In the scoring experiment, the putative binding modes collected from the groups participating in the prediction challenge and redistributed by CAPRI were filtered if biological information is available. Thereafter, the filtered binding modes were ranked using ITScorePP and clustered according to the RMSDs. For the MOCKPP server scoring challenge, the best binding mode from each of the top 10-ranked clusters was selected and submitted to CAPRI. For the human scoring challenge, up to 100 clustered binding modes were manually inspected, and ten complexes were selected for submission.

Success and Failures

In CASP15-CAPRI Round 54, our human and server group predicted 26 and 20, respectively, acceptable or better results in top 5 models out of the 41 assessment units (AUs) in 37 targets. Our success rates, either of human or server prediction, are comparable to that of AlphaFold-Multimer, which is 25 out of 41 AUs. The similarity is largely due to our strategy of using the models generated by AlphaFold-Multimer as a biased reference. However, AlphaFold-Multimer performed better than us in generating high-accuracy models. AlphaFold-Multimer produced high-accurate results for 11 AUs, as compared to 8 by our human approach. Specifically, for T191, T202 and AU1 of T203, the top 5 models submitted by our human approach were of lower accuracy than those produced by AlphaFold-Multimer. Nonetheless, AlphaFold-Multimer failed to produce correct models for T200 using the default settings, while we produced acceptable-accuracy model in the top 1 model. The evaluation results show the urgency for us to develop a new approach to leverage the much-improved success of AlphaFold-Multimer for better results, such as using alternative settings to construct diverse structural models and applying an improved protocol to rank these models.

References

1. Lensink MF, Brysbaert G, Mauri T, Nadzirin N, Velankar S, Chaleil RAG, Clarence T, Bates PA, Kong R, Liu B, Yang G, Liu M, Shi H, Lu X, Chang S, Roy RS, Quadir F, Liu J, Cheng J, Antoniak A, Czaplewski C, Giełdoń A, Kogut M, Lipska AG, Liwo A, Lubecka EA, Maszota-Zieleniak M, Sieradzan AK, Ślusarz R, Wesołowski PA, Zięba K, Del Carpio Muñoz CA, Ichiishi E, Harmalkar A, Gray JJ, Bonvin AMJJ, Ambrosetti F, Vargas Honorato R, Jandova Z, Jiménez-García B, Koukos PI, Van Keulen S, Van Noort CW, Réau M, Roel-Touris J, Kotelnikov S, Padhorny D, Porter KA, Alekseenko A, Ignatov M, Desta I, Ashizawa R, Sun Z, Ghani U, Hashemi N, Vajda S, Kozakov D, Rosell M, Rodríguez-Lumbreras LA, Fernandez-Recio J, Karczynska A, Grudinin S, Yan Y, Li H, Lin P, Huang SY, Christoffer C, Terashi G, Verburgt J, Sarkar D, Aderinwale T, Wang X, Kihara D, Nakamura T, Hanazono Y, Gowthaman R, Guest JD, Yin R, Taherzadeh G, Pierce BG, Barradas-Bautista D, Cao Z, Cavallo L, Oliva R, Sun Y, Zhu S, Shen Y, Park T, Woo H, Yang J, Kwon S, Won J, Seok C, Kiyota Y, Kobayashi S, Harada Y, Takeda-Shitaka M, Kundrotas PJ, et al. Prediction of protein assemblies, the next frontier: The CASP14-CAPRI experiment. *Proteins Struct Funct Bioinforma*. 2021;89(12):1800-1823. doi:10.1002/PROT.26222
2. Jumper J, Evans R, Pritzel A, Green T, Figurnov M, Ronneberger O, Tunyasuvunakool K, Bates R, Žídek A, Potapenko A, Bridgland A, Meyer C, Kohl SAA, Ballard AJ, Cowie A, Romera-Paredes B, Nikolov S, Jain R, Adler J, Back T, Petersen S, Reiman D, Clancy E, Zielinski M, Steinegger M, Pacholska M, Berghammer T, Bodenstein S, Silver D, Vinyals O, Senior AW, Kavukcuoglu K, Kohli P, Hassabis D. Highly accurate protein structure prediction with AlphaFold. *Nature*. 2021;596(7873):583-589. doi:10.1038/s41586-021-03819-2
3. Evans R, O'Neill M, Pritzel A, Antropova N, Senior A, Green T, Žídek A, Bates R, Blackwell S, Yim J, Ronneberger O, Bodenstein S, Zielinski M, Bridgland A, Potapenko A, Cowie A, Tunyasuvunakool K, Jain R, Clancy E, Kohli P, Jumper J, Hassabis D. Protein complex prediction with AlphaFold-Multimer. *bioRxiv*. Published online March 10, 2022:2021.10.04.463034. doi:10.1101/2021.10.04.463034
4. Duan R, Qiu L, Xu X, Ma Z, Merideth BR, Shyu CR, Zou X. Performance of human and server prediction in CAPRI rounds 38-45. *Proteins Struct Funct Bioinforma*. 2020;88(8):1110-1120. doi:10.1002/prot.25956
5. Qiu L, Bhutoria S, Kalpana GV, Zou X. Computational modelling of IN-CTD/TAR complex to elucidate additional strategies to inhibit HIV-1 replication. Invited book chapter in *Methods in*

Molecular Biology, Virus-Hosts Interactions. Marilena Aquino de Muro (ed.), Chapter 7, Springer Science + Business Media, New York, in press.

6. Chen R, Li L, Weng Z. ZDOCK: An initial-stage protein-docking algorithm. *Proteins Struct Funct Genet*. 2003;52(1):80-87. doi:10.1002/prot.10389
7. Maier JA, Martinez C, Kasavajhala K, Wickstrom L, Hauser KE, Simmerling C. ff14SB: Improving the Accuracy of Protein Side Chain and Backbone Parameters from ff99SB. *J Chem Theory Comput*. 2015; 11(8): 3696-3713. doi:10.1021/ACS.JCTC.5B00255/SUPPL_FILE/CT5B00255_SI_001.PDF
8. Jorgensen WL, Chandrasekhar J, Madura JD, Impey RW, Klein ML. Comparison of simple potential functions for simulating liquid water. *J Chem Phys*. 1983;79(2):926-935. doi:10.1063/1.445869
9. Case DA, Betz RM, Cerutti DS, Cheatham III, T.E., Darden TA, Duke RE, Giese TJ, Gohlke H, Goetz AW, Homeyer N, Izadi S, Janowski P, Kaus J, Kovalenko A, Lee TS, LeGrand S, Li P, Lin C, Luchko T, Luo R, Madej B, Mermelstein D, Merz KM, Monard G, Nguyen HT, Nguyen HT, Omelyan I, Onufriev A, Roe DR, Roitberg A, Sagui C, Simmerling CL, Botello-Smith WM, Swails J, Walker RC, Wang J, Wolf RM, Wu X, Xiao L, Kollman PA. Amber 2016. University of California, San Francisco, 2016.

Grant acknowledgement

This work is supported by NIH/NIGMS R35GM136409 to XZ.

Emails:
Rui Duan: duanr@missouri.edu
Liming Qiu: qiul@missouri.edu
Xianjin Xu: xuxian@missouri.edu
Xiaoqin Zou: zoux@missouri.edu

A Prediction Result (Zou)

Target		T191	T192	T193	T194	T195	T197	T198	T199	T200	T201	T202	T203 AU1	T203 AU2	T204 AU1	T204 AU2	T205	T206	T207	T208	T209	T210	T211	T212	T213	T214	T216	T217	T218	T219 AU1	T220 AU1	T220 AU2	T221 AU1	T221 AU2	T222	T223	T224	T225	T226	T227	T229	T230
Top1	H	0	1	1	0	0	0	0	1	0	0	0	0	0	0	0	0	0	0	0	0	1	1	0	0	0	0	0	0	0	0	0	0	0	0	0	0	0	0	0	0	1
	M	1	0	0	1	0	0	0	0	0	1	1	0	0	0	0	0	0	0	0	0	0	0	1	0	0	0	0	1	1	1	1	1	1	1	0	0	1	0	0	0	0
	A	0	0	0	0	1	0	0	0	1	0	0	1	0	0	0	0	0	0	0	0	0	0	0	0	0	0	0	0	0	0	0	0	0	0	0	0	0	0	1	0	0
Top5	H	0	3	3	0	0	0	0	2	0	1	0	0	0	0	0	0	0	0	1	0	4	1	0	0	0	0	0	0	0	0	0	0	0	0	0	0	0	0	0	0	4
	M	5	1	2	2	0	0	0	3	0	4	5	0	0	0	0	0	0	0	0	0	1	0	2	0	0	0	0	1	5	5	2	5	2	3	0	0	5	0	0	0	1
	A	0	1	0	1	3	0	0	0	1	0	0	2	0	0	0	0	0	0	0	0	0	1	2	0	0	0	0	0	0	0	1	0	1	0	3	0	0	3	3	0	0

B Prediction Results (MDOCKPP)

Target		T191	T192	T193	T194	T195	T197	T198	T199	T200	T201	T202	T203 AU1	T203 AU2	T204 AU1	T204 AU2	T205	T206	T207	T208	T209	T210	T211	T212	T213	T214	T216	T217	T218	T219 AU1	T220 AU1	T220 AU2	T221 AU1	T221 AU2	T222	T223	T224	T225	T226	T227	T229	T230
Top1	H	0	0	1	0	0	0	0	0	0	0	0	0	0	0	0	0	0	0	0	0	1	0	0	0	0	0	0	0	0	0	0	0	0	0	0	0	0	0	0	0	1
	M	1	1	0	0	0	0	0	1	0	1	1	0	0	0	0	0	0	0	0	0	0	0	1	0	0	0	0	1	1	1	0	1	0	1	0	0	1	0	0	0	0
	A	0	0	0	1	1	0	0	0	0	0	0	1	0	0	0	0	0	0	0	0	0	0	0	0	0	0	0	0	0	0	0	0	0	0	1	0	0	1	0	0	0
Top5	H	0	0	2	0	0	0	0	1	0	0	0	0	0	0	0	0	0	0	0	0	3	0	0	0	0	0	0	0	0	0	0	0	0	1	0	0	0	0	0	0	5
	M	2	4	3	0	0	0	0	4	0	1	2	0	0	0	0	0	0	0	0	0	1	0	1	0	0	0	0	1	5	5	0	5	0	2	0	0	3	0	0	0	0
	A	0	1	0	1	3	0	0	0	0	0	3	2	0	0	0	0	0	0	0	0	0	0	0	0	0	0	0	0	0	0	0	0	0	1	2	0	1	1	0	0	0

C Scoring Results (Zou)

Target		T191	T192	T193	T194	T195	T197	T198	T199	T200	T201	T202	T203 AU1	T203 AU2	T204 AU1	T204 AU2	T205	T206	T207	T208	T209	T210	T211	T212	T213	T214	T216	T217	T218	T219 AU1	T220 AU1	T220 AU2	T221 AU1	T221 AU2	T222	T223	T224	T225	T226	T227	T229	T230
Top1	H	-	1	1	0	0	0	0	0	0	0	0	0	0	0	0	0	0	0	0	0	1	0	0	0	0	0	0	0	0	0	0	0	0	0	0	0	0	0	0	0	0
	M	-	0	0	1	0	0	0	1	0	1	1	0	0	0	1	0	0	0	0	0	0	0	1	0	0	0	0	1	1	1	0	1	1	1	0	0	1	0	1	0	1
	A	-	0	0	0	1	0	0	0	0	0	0	1	0	1	0	0	0	0	0	0	0	0	0	0	0	0	0	0	0	0	1	0	0	0	0	0	0	0	0	0	0
Top5	H	-	5	5	0	0	0	0	1	1	0	1	0	0	0	0	0	0	0	0	0	3	1	0	0	0	0	0	0	0	0	0	0	0	0	0	0	0	0	0	0	3
	M	-	0	0	1	0	0	0	4	0	3	4	0	0	0	5	0	0	0	0	0	2	0	3	0	0	0	0	1	5	5	2	4	3	3	0	0	5	0	4	0	2
	A	-	0	0	1	5	0	0	0	0	1	0	4	0	5	0	0	0	0	1	0	0	1	2	0	0	0	0	0	0	0	3	1	1	1	1	0	0	2	0	0	0

D Scoring Results (MDOCKPP)

Target		T191	T192	T193	T194	T195	T197	T198	T199	T200	T201	T202	T203 AU1	T203 AU2	T204 AU1	T204 AU2	T205	T206	T207	T208	T209	T210	T211	T212	T213	T214	T216	T217	T218	T219 AU1	T220 AU1	T220 AU2	T221 AU1	T221 AU2	T222	T223	T224	T225	T226	T227	T229	T230
Top1	H	-	1	0	0	0	0	0	0	0	0	0	0	0	0	0	0	0	0	0	0	0	0	0	0	0	0	0	0	0	0	0	0	0	0	0	0	0	0	0	0	0
	M	-	0	1	1	0	0	0	1	0	1	1	0	0	0	1	0	0	0	0	0	1	0	1	0	0	0	0	0	1	1	0	1	0	0	0	0	1	0	1	0	1
	A	-	0	0	0	1	0	0	0	0	0	0	1	0	1	0	0	0	0	0	0	0	0	0	0	0	0	0	0	0	0	1	0	1	0	0	0	0	0	0	0	0
Top5	H	-	4	4	0	0	0	0	1	1	0	1	0	0	0	0	0	0	0	0	0	1	0	0	0	0	0	0	0	0	0	0	0	0	0	0	0	0	0	0	0	2
	M	-	1	1	3	0	0	0	4	0	3	4	0	0	0	5	0	0	0	0	1	4	0	3	0	0	0	0	0	5	5	2	4	3	2	0	0	5	0	4	0	3
	A	-	0	0	2	5	0	0	0	0	1	0	5	0	5	0	0	0	0	1	0	0	0	2	0	0	0	1	0	0	0	3	1		1	1	0	0	1	0	0	0

Figure Prediction (A, B) and Scoring (C, D) results for each target from the human (Zou) and server (MDOCKPP) submissions, respectively. H, M and A stands for high, medium and acceptable accuracy, respectively, and the number in each cell (colored red, green and light blue, respectively, if it is not zero) is the number of models of high, medium and acceptable quality, respectively, in the top-N submission. Target 191 was canceled for Scoring by CASP/CAPRI. The assessment of Target 203 is based on the average accuracy of interfaces 1-3 (AU1) and the accuracy of interface 4 (AU2). The assessment of Target 204 is based on the best accuracy of interfaces 1-2 (AU1) and the average accuracy of interface 3-8 (AU2). The assessment of Target 219-221 is based on the best accuracy of interfaces 1-3 (AU1) and the best accuracy of interface 4 (AU2). (The above summary is based on the assessment results from CAPRI. https://www.capri-docking.org/assessment/)
